# Elucidating direct kinase targets of compound Danshen dropping pills employing archived data and prediction models

**DOI:** 10.1038/s41598-021-89035-4

**Published:** 2021-05-05

**Authors:** Tongxing Wang, Lu Liang, Chunlai Zhao, Jia Sun, Hairong Wang, Wenjia Wang, Jianping Lin, Yunhui Hu

**Affiliations:** 1 GeneNet Pharmaceuticals Co. Ltd., No. 1, Tingjiang West Road, Beichen District, Tianjin, 300410 China; 2 College of Pharmacy, Nankai University, Haihe Education Park, 38 Tongyan Road, Jinnan District, Tianjin, 300353 China

**Keywords:** Cheminformatics, Computational chemistry, Drug discovery and development, Pharmacology, Target identification, Target validation

## Abstract

Research on direct targets of traditional Chinese medicine (TCM) is the key to study the mechanism and material basis of it, but there is still no effective methods at present. We took Compound Danshen dropping pills (CDDP) as a study case to establish a strategy to identify significant direct targets of TCM. As a result, thirty potential active kinase targets of CDDP were identified. Nine of them had potential dose-dependent effects. In addition, the direct inhibitory effect of CDDP on three kinases, AURKB, MET and PIM1 were observed both on biochemical level and cellular level, which could not only shed light on the mechanisms of action involved in CDDP, but also suggesting the potency of drug repositioning of CDDP. Our results indicated that the research strategy including both in silico models and experimental validation that we built, were relatively efficient and reliable for direct targets identification for TCM prescription, which will help elucidating the mechanisms of TCM and promoting the modernization of TCM.

## Introduction

Traditional Chinese medicine (TCM) prescriptions are the characteristics of Chinese medicine. They have been practiced for thousands of years and have been proved to be effective in modern clinical practice. These prescriptions embody the dialectical thought of Chinese medicine and the medication holistic view. In recent years, the reductionist research model has accumulated a lot of data, and also provided illuminating research results, such as the discovery of artemisinin^1^. It was discovered by Youyou Tu, a Chinese traditional medicine scientist, which is an effective and quick acting antimalarial drug. However, there is still a lack of effective approaches to systematically study its mechanism. The research of reductionism is not capable of answering the essential question of the overall efficacy of TCM. It may lead to deviate from the system theory of TCM, so it needs to be combined with the system theory. In recent years, a variety of "omics" techniques based on system theory have been widely used in TCM research^2–5^, for elucidating the pharmacological characteristics of TCM better^6–9^, but still cannot fully reveal the nature of it. Comprehensively understanding the mechanism of synergism among the effective components, drug targets and metabolic pathways remains highly demanded. One key to break this dilemma is to carry out the research on the direct target of TCM. It can not only clarify the pharmacological mechanism of TCM from the origin and scientifically interpret its traditional efficacy, but also unveil novel disease-related mechanism^10^ and provide reasonable estimation for TCM repositioning. At present, technical methods to screen and determine the direct targets for TCM efficiently and accurately are still poorly developed, which hinders elucidating the mechanisms of TCM essentially.

CDDP consists of *Radix Salviae* (Danshen), *Panax Notoginseng (Burk.) F. H. Chen Ex C. Chow* (Sanqi), *Borneolum Syntheticum* (Bingpian). It is widely used in treating coronary artery disease (CAD) and angina pectoris including acute stage and preventive treatment. Although many research articles about CDDP have been published already, the research on its mechanism of action is still not in-depth^11–14^. Most studies focused on the genes or proteins regulated by CDDP treatment, most of which can be referred as indirect targets, so far there is no report focusing on the direct targets of CDDP. It is of great significance to obtain the direct targets of TCM.

Kinases is an important class of drug targets. Among them, protein kinases family is the largest group of kinases, which act on specific proteins and modulate their activities. These kinases play a wide range of roles in cell signaling and complex life activities, and their dysfunction plays an important causal role in many human diseases, including cancer, inflammatory diseases, central nervous system diseases, cardiovascular diseases and so on^15^.

**Figure 1:**
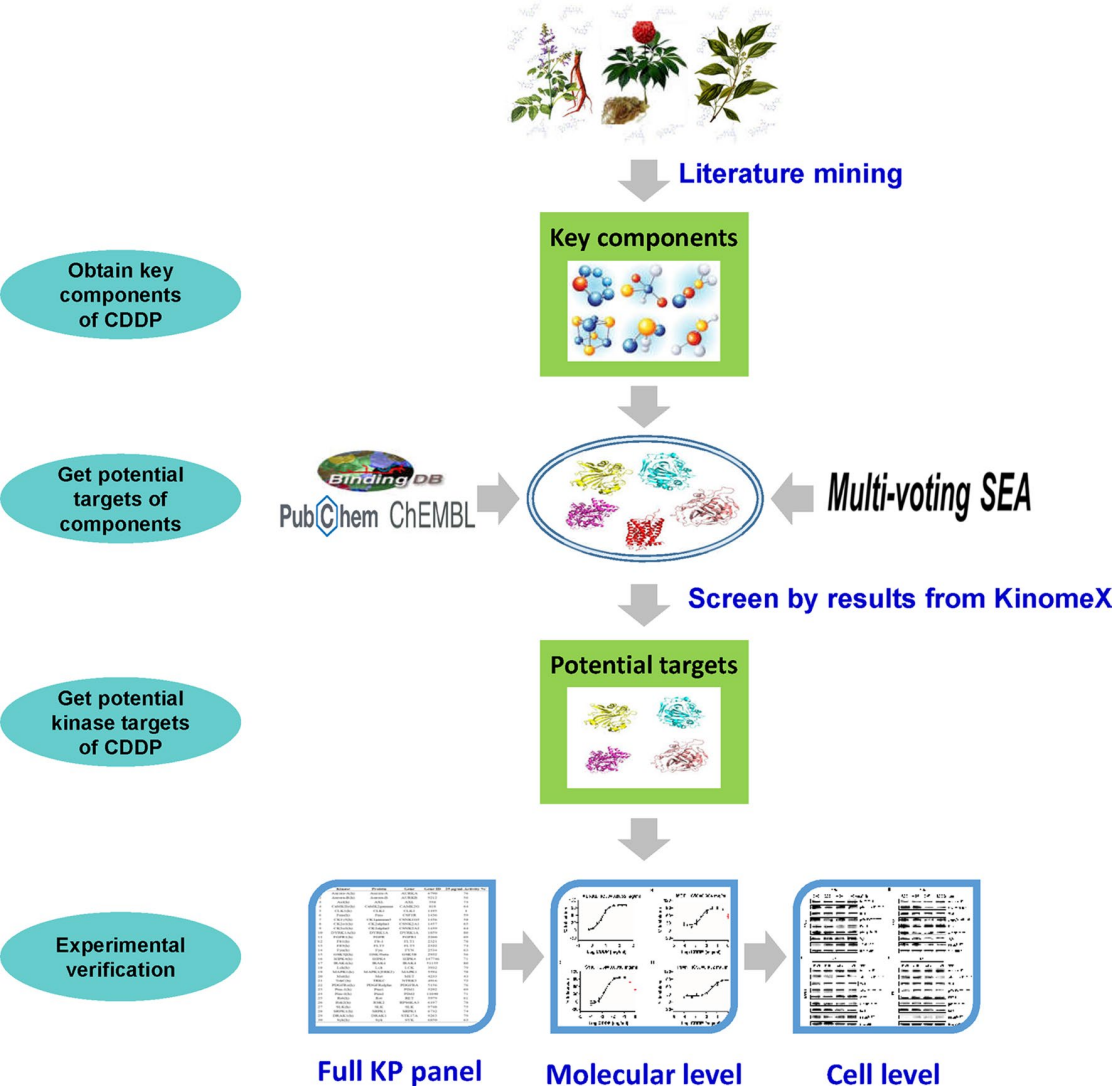
The flowchart of research strategy to obtain the direct kinase targets of CDDP.

To obtain the direct targets of the whole prescription, we proposed a hypothesis that the potential direct targets of important components are more likely to be direct targets of the whole prescription. Therefore, the potential targets of components were utilized to speculate the potential direct targets of the whole prescription. More importantly, we adopt a strategy of integrating multi-source data to improve the success rate of validation results. In this study, we took CDDP as a study case to obtain its direct kinase targets. Firstly, the literature database of CDDP was constructed by literature retrieval, and the important components contained in CDDP were extracted. Secondly, the potential targets of important components were obtained through public database querying and Multi-voting chemical similarity ensemble approach (SEA) algorithm predicting. Then, the kinase targets got from KinomeX system was used to filter the potential kinase targets of CDDP. Finally, 30 active targets were obtained and some of them were further validated by a series of experiments (Fig. 1). In silico methods and experimental verification combination strategy has been demonstrated effective and efficient in deciphering direct targets of TCM.

## Results

### Important components in CDDP

3719 Chinese-language literatures and 59 English-language literatures were obtained through retrieving the customized terms of the CDDP (time to April 15, 2020). Through literature reading manually, the components information of CDDP was extracted. According to the screening criteria of important ingredients, a total of 39 ingredients were collected. In addition, quercetin, a potential important component of CDDP was also included for subsequent analysis. All 40 important components of CDDP are shown in Table 1.

**Table 1:** 40 important components of CDDP from literatures and databases.

#	Component name	PubChem CID
CDDP 01	Danshensu	11600642
CDDP 02	Tanshinone I	114917
CDDP 03	Cryptotanshinone	160254
CDDP 04	Tanshinone IIA	164676
CDDP 05	Dihydrotanshinone I	11425923
CDDP 06	Salvianolic acid A	5281793
CDDP 07	Salvianolic acid B	11629084
CDDP 08	Salvianolic acid B	124518070
CDDP 09	Salvianolic acid D	75412558
CDDP 10	Salvianolic acid G	11683160
CDDP 11	Protocatechuic aldehyde	8768
CDDP 12	Rosmarinic acid	5281792
CDDP 13	Lithospermic acid	6441498
CDDP 14	Ginsenoside-Rg1	441923
CDDP 15	Ginsenoside-Rb1	9898279
CDDP 16	Ginsenoside-Rh1	12855920
CDDP 17	Ginsenoside-Rd	11679800
CDDP 18	Notoginsenoside R1	441934
CDDP 19	Ginsenoside Re	441921
CDDP 20	Borneol	1201518
CDDP 21	Isoborneol	6973640
CDDP 22	Caffeic acid	689043
CDDP 23	Tanshinone IIB	9926694
CDDP 24	Methylenetanshinquinone	105118
CDDP 25	Salvianolic acid C	13991590
CDDP 26	Ginsenoside-Rf	441922
CDDP 27	Ginsenoside-F2	9918692
CDDP 28	Ginsenoside-F1	9809542
CDDP 29	Ginsenoside-Rb2	6917976
CDDP 30	Ginsenoside-Rb3	12912363
CDDP 31	Ginsenoside-Rg2	21599924
CDDP 32	Notoginsenoside-R2	21599925
CDDP 33	20(S)-Ginsenoside Rg3	9918693
CDDP 34	20(R)-Ginsenoside Rg3	46887680
CDDP 35	Miltirone	160142
CDDP 36	Protocatechuic acid	72
CDDP 37	Catechol	289
CDDP 38	Vanillic acid	8468
CDDP 39	4-Hydroxy-3-methyloxyphenyl lactic acid	160637
CDDP 40	Quercetin	5280343

### Potential kinase targets of CDDP

Through querying the three public databases, 262 known targets including 55 kinase targets were obtained, and 377 predicted targets including 121 kinase targets were predicted based on the Multi-voting SEA algorithm. By integrating the above two parts of targets, a total of 479 potential direct targets were obtained, including 148 kinase targets (Table 2 and Supplementary Table S2).

**Table 2:** Number of potential direct kinase targets of CDDP.

Target sources	All targets	Kinase targets	Targets screened by KinomeX
Known targets	262	55	37
Predicted targets	377	121	92
Common targets	160	28	20
Total targets	479	148	109

Known targets of CDDP indicate the targets of 40 compounds obtained by retrieving authoritative public databases ChEMBL, PubChem and BingdingDB; Predicted targets of CDDP indicate the targets of 40 compounds predicted by Multi-voting SEA algorithm; Common targets are the intersection of known targets and predicted targets.

Through KinomeX system, we got 288 kinase targets (see Supplementary Table S3) for 40 important components. To obtain the kinase target set of CDDP with high reliability, we took this result to filter the 148 kinase targets from above. As a result, 37 and 92 kinase targets were screened out from the retrieval results of public database and prediction results of Multi-voting SEA, respectively. These two parts shared 20 kinase targets. In total, 109 kinase targets were treated as the potential kinase targets of CDDP for subsequent experimental verification (Table 2 and Supplementary Table S4).

### Kinase targets of CDDP verified by Full KP panel

Among the above 109 kinase targets, 106 kinase targets were contained in Full KP panel developed by Eurofins Company. We test the activity of them at 25 µg/mL concentration of CDDP. The active targets results were screened according to the threshold described in the method (Table 3). In total, 30 active targets were obtained.

**Table 3:** Kinase targets verified by activity test at 25 µg/mL concentration of CDDP.

#	Kinase	Protein	Gene	Gene ID	Activity (%*)
1	Aurora-A(h)	Aurora-A	AURKA	6790	76
2	Aurora-B(h)	Aurora-B	AURKB	9212	56
3	Axl(h)	AXL	AXL	558	73
4	CaMKIIγ(h)	CaMK2gamma	CAMK2G	818	64
5	CLK1(h)	CLK1	CLK1	1195	4
6	Fms(h)	Fms	CSF1R	1436	59
7	CK1γ3(h)	CK1gamma3	CSNK1G3	1456	58
8	CK2α1(h)	CK2alpha1	CSNK2A1	1457	65
9	CK2α2(h)	CK2alpha2	CSNK2A2	1459	64
10	DYRK1A(h)	DYRK1A	DYRK1A	1859	80
11	FGFR1(h)	FGFR	FGFR1	2260	69
12	Flt1(h)	Flt-1	FLT1	2321	78
13	Flt3(h)	FLT3	FLT3	2322	74
14	Fyn(h)	Fyn	FYN	2534	63
15	GSK3β(h)	GSK3beta	GSK3B	2932	56
16	HIPK4(h)	HIPK4	HIPK4	147746	71
17	IRAK4(h)	IRAK4	IRAK4	51135	80
18	Lck(h)	Lck	LCK	3932	79
19	MAPK1(h)	MAPK1(ERK2)	MAPK1	5594	78
20	Met(h)	Met	MET	4233	43
21	TrkC(h)	TRKC	NTRK3	4916	72
22	PDGFRα(h)	PDGFRalpha	PDGFRA	5156	76
23	Pim-1(h)	Pim1	PIM1	5292	69
24	Pim-2(h)	Pim2	PIM2	11040	71
25	Ret(h)	Ret	RET	5979	61
26	Rsk2(h)	RSK2	RPS6KA3	6197	78
27	SLK(h)	SLK	SLK	9748	75
28	SRPK1(h)	SRPK1	SRPK1	6732	74
29	DRAK1(h)	DRAK1	STK17A	9263	79
30	Syk(h)	Syk	SYK	6850	63

*The kinase activity inhibition rate of the sample compared to the blank group. The kinase activity of the blank was 100%. Generally speaking, the residual enzyme activity below 30% is strong inhibition, 30–70% is moderate inhibition. Considering the characteristics of TCM, we take 80% as the screening threshold. The lower the value is, the stronger the kinase activity is inhibited.

The overall accuracy was about 28.3% (Table 4). As for the known targets from public databases, 15 out of 37 were verified, and the accuracy is about 40.5%. 26 out of 89 kinase targets predicted by Multi-voting SEA got active value, bringing the accuracy up to 29.2%. 11 from common 20 kinase targets were verified, achieving 55% accuracy.

**Table 4:** Validation accuracy of potential direct kinase targets CDDP.

Target sources	Kinase targets screened by KinomeX	In full KP panel	Active targets	Accuracy (%)
Known targets	37	37	15	40.5
Predicted targets	92	89	26	29.2
Common targets	20	20	11	55
Total targets	109	106	30	28.3

Among them, 14 targets with active value lower than 70 were retested at the concentration of 250 µg/mL, and 9 targets with potential dose-dependent effect were found (Table 5).

**Table 5:** Activity data for 14 kinase targets tested at different concentration of CDDP.

#	Kinase	Gene	Gene ID	Activity data 1 (%^a^)	Activity data 2 (%^a^)
1	Aurora-B(h)^b^	AURKB	9212	56	28
2	CaMKIIγ(h)	CAMK2G	818	64	86
3	CK1γ3(h)^b^	CSNK1G3	1456	58	23
4	CK2α1(h)^b^	CSNK2A1	1457	65	47
5	CK2α2(h)^b^	CSNK2A2	1459	64	43
6	CLK1(h)	CLK1	1195	4	4
7	FGFR1(h)^b^	FGFR1	2260	69	43
8	Fms(h)	CSF1R	1436	59	58
9	Fyn(h)	FYN	2534	63	63
10	GSK3β(h)^b^	GSK3B	2932	56	39
11	Met(h)^b^	MET	4233	43	8
12	Pim-1(h)^b^	PIM1	5292	69	18
13	Ret(h)	RET	5979	61	70
14	Syk(h)^b^	SYK	6850	63	2

^a^The lower the value, the stronger the binding activity.

^b^The kinase with potential dose-dependent effect. Activity data 1 and 2 is the activity data of kinase targets tested at 25 µg/mL and 250 µg/mL concentration of CDDP, respectively.

### Kinase assays showed that CDDP could inhibit AURKB, MET, PIM1, and SYK with dose-dependent effect

To further validate the targets with dose-dependent effect, we chose AURKB, MET, PIM1 and SYK targets to carry out the kinase assays. The mean half-maximal inhibitory concentration (IC_50_) value and its standard deviation were obtained (Table 6, Fig. 2). Of four kinase targets, three yielded IC_50_ values lower (better) than 10 μg/mL derived from the concentration–response curves, and the remainder had an IC_50_ value better than 35 μg/mL (Fig. 2). The inhibition curves of positive control compound (Danusertib, Cabozantinib, AZD1208 and Cerdulatinib), testing on AURKB, MET, PIM1 and SYK respectively can be seen in Supplementary Fig. S1.

**Table 6:** IC_50_ values of CDDP testing on AURKB, MET, PIM1 and SYK.

#	Drug name	Target gene name	IC_50_ (mg/mL): mean ± SD
1	CDDP	AURKB	0.0053 ± 0.0011
2	CDDP	MET	0.0039 ± 0.0001
3	CDDP	PIM1	0.0358 ± 0.0138
4	CDDP	SYK	0.0019 ± 0.0007

**Figure 2:**
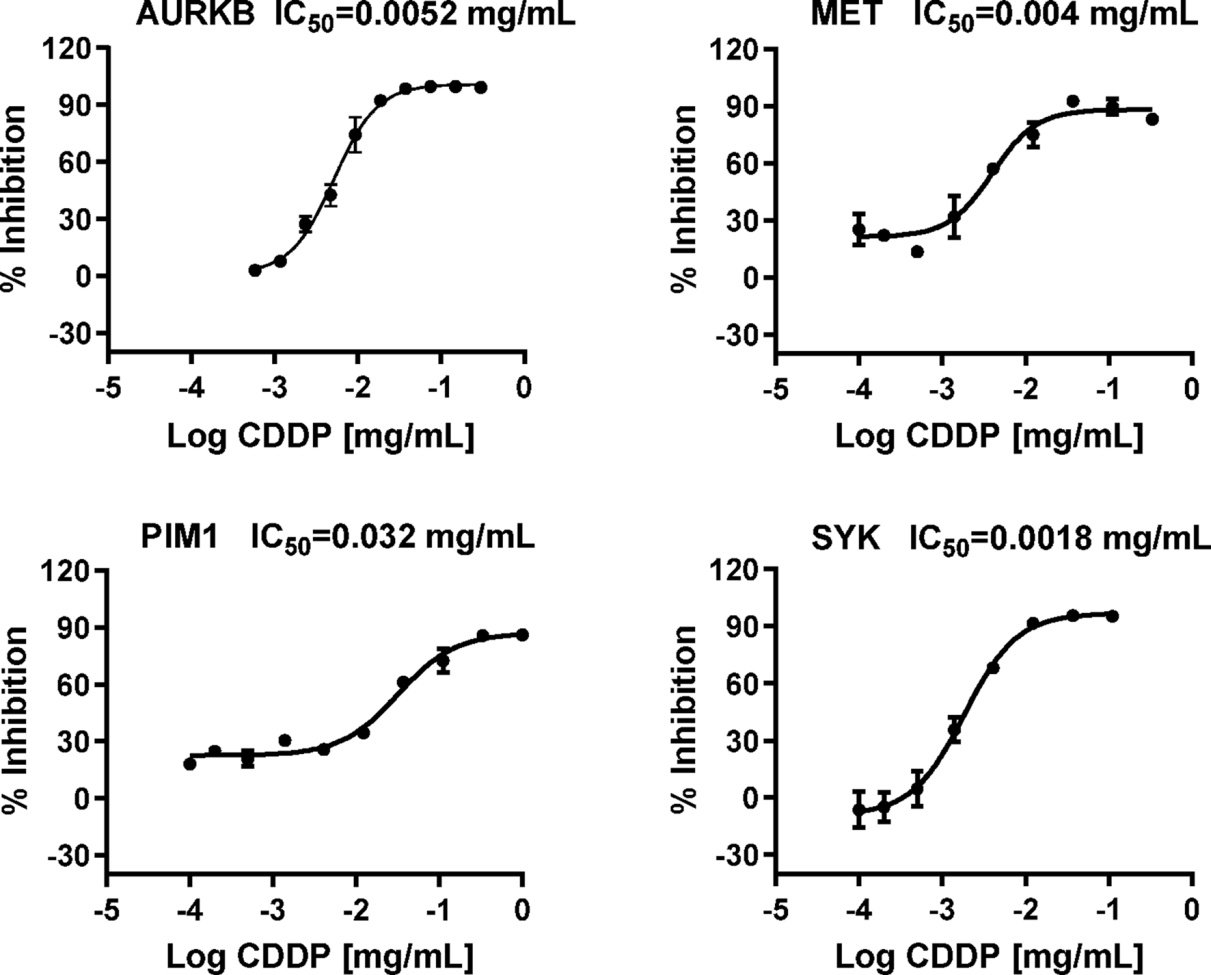
Inhibition curves of CDDP testing on AURKB, MET, PIM1 and SYK. Ten concentration points were obtained by 3 dilution fold.

### Changes in the expression of AURKB, MET, PIM1, SYK and their phosphorylated proteins level after treatment by CDDP in four cancer cell lines

**Figure 3:**
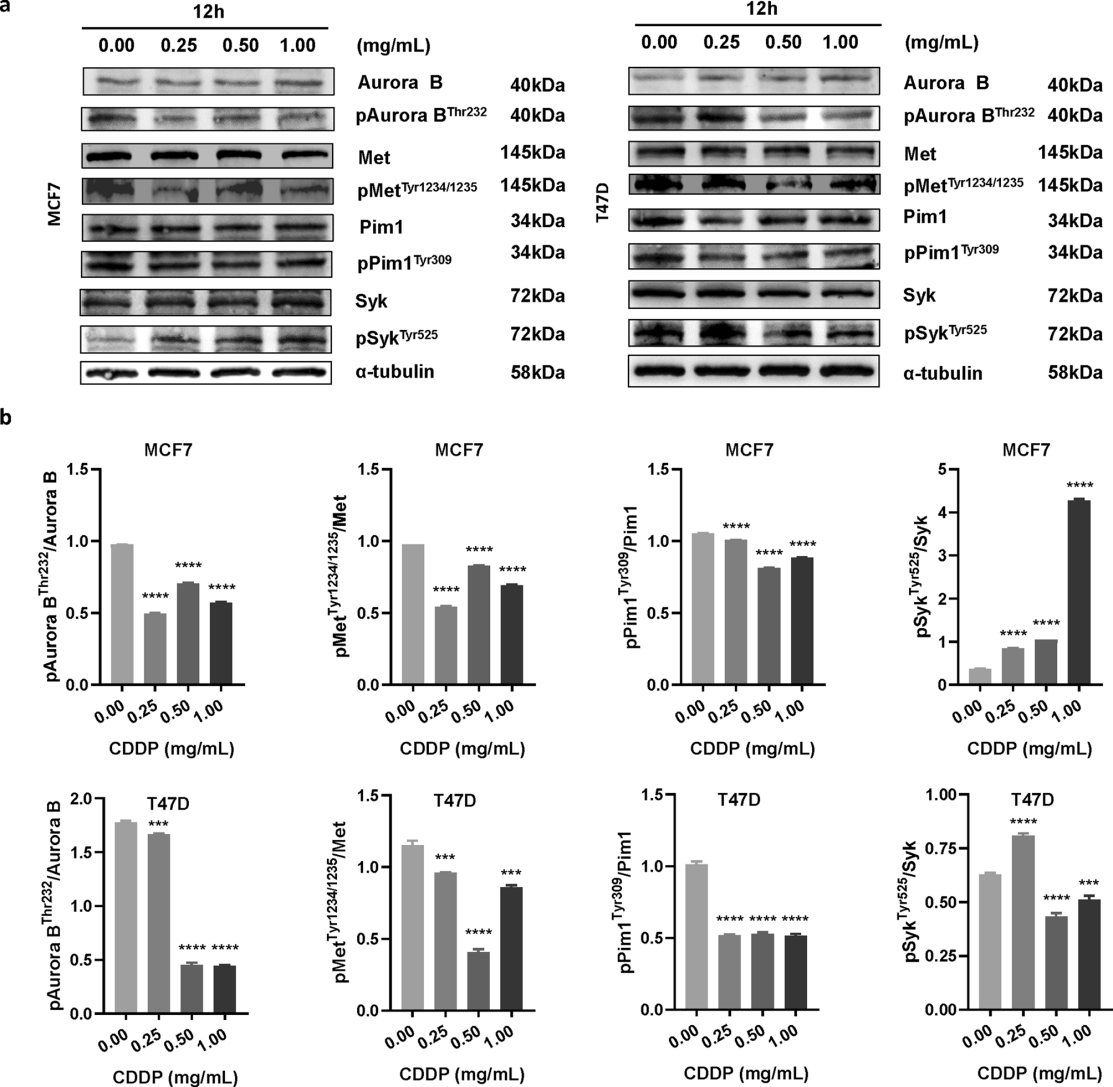
Effects of CDDP on the expression of AURKB, MET, PIM1, SYK and their corresponding phosphorylated proteins level in breast cancer cell lines MCF7 and T47D. (**a**) Western blots indicating protein levels of AURKB, MET, PIM1, SYK and their corresponding phosphorylated proteins in MCF7 and T47D cells. α-tubulin was used as a loading control. One representative image is shown out of three independent experiments. (**b**) Effect of CDDP on the activity of AURKB, MET, PIM1, and SYK in MCF7 and T47D cells. Each cell lines were divided into four groups as follows: control group, CDDP group (0.25 mg/mL), CDDP group (0.5 mg/mL), and CDDP group (1.0 mg/mL). The treatment time was 12 h for MCF7 and T47D cells. The samples derive from the same experiment and that gels/blots were processed in parallel. Statistical significance was determined by a two-tailed, unpaired Student t-test (*P < 0.05, **P < 0.01, ***P < 0.001, ****P < 0.0001 vs control). CDDP, Compound Danshen dropping pills. Full-length blots/gels are presented in Supplementary Fig. S2–3.

**Figure 4:**
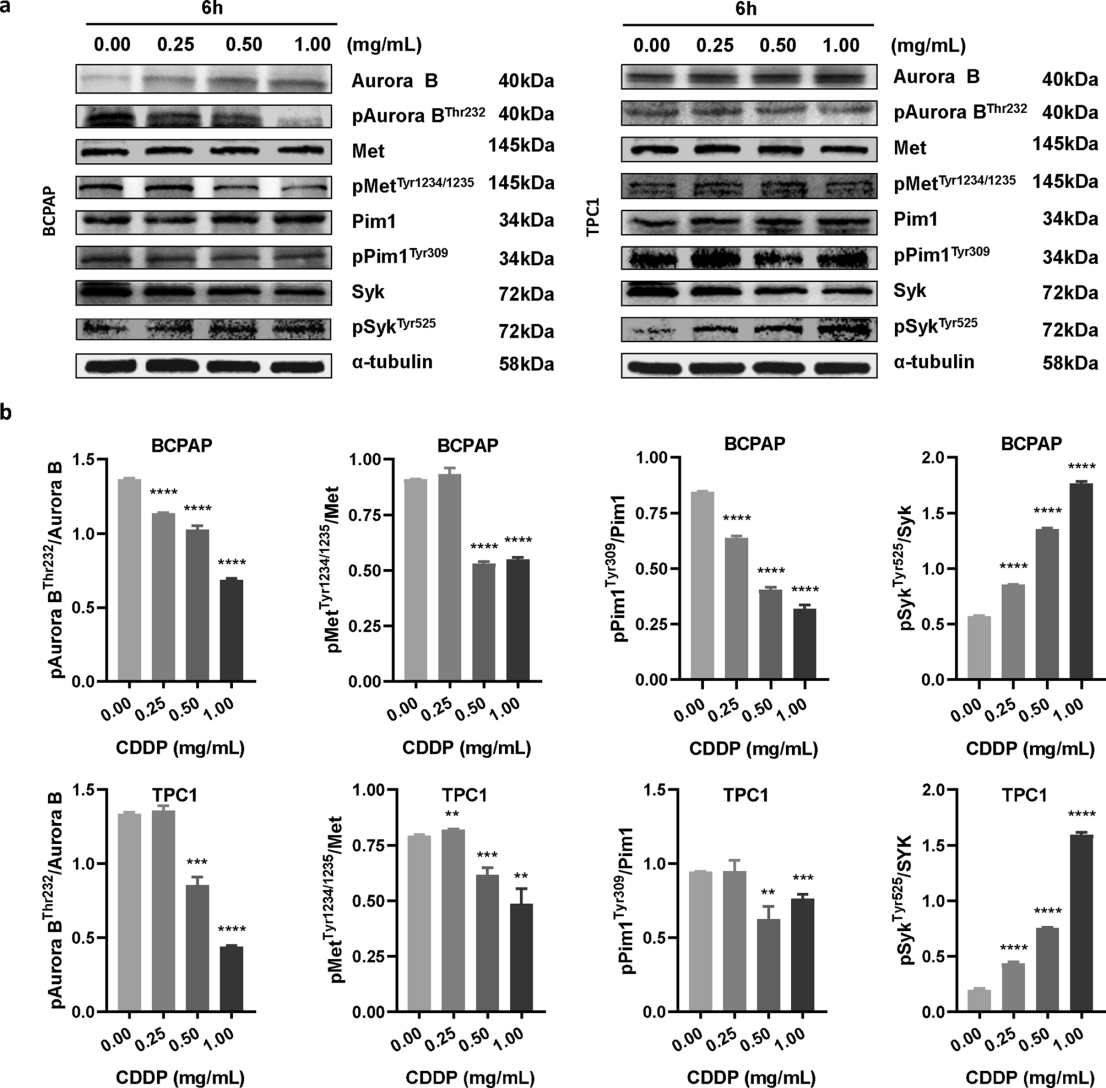
Effects of CDDP on the expression of AURKB, MET, PIM1, SYK and their corresponding phosphorylated proteins level in thyroid cancer cell lines TPC1 and BCPAP. (**a**) Western blots indicating protein levels of AURKB, MET, PIM1, SYK and their corresponding phosphorylated proteins in TPC1 and BCPAP cells. α-tubulin was used as a loading control. One representative image is shown out of three independent experiments. (**b**) Effect of CDDP on the activity of AURKB, MET, PIM1, and SYK in TPC1 and BCPAP cells. Each cell lines were divided into four groups as follows: Control group, CDDP group (0.25 mg/mL), CDDP group (0.5 mg/mL), and CDDP group (1.0 mg/mL). The treatment time was 6 h for TPC1 and BCPAP cells. The samples derive from the same experiment and that gels/blots were processed in parallel. Statistical significance was determined by a two-tailed, unpaired Student t-test (*P < 0.05, **P < 0.01, ***P < 0.001, ****P < 0.0001 vs control). CDDP, Compound Danshen dropping pills. Full-length blots/gels are presented in Supplementary Figs. S4–5.

Human breast cancer cell lines MCF7, T47D and thyroid cancer cell lines TPC1, BCPAP were used to conduct the western blot analysis (Figs. 3, 4, and see Supplementary Table S5). The full-length gels and blots are included in a Supplementary Figs. S2–5. After CDDP treatment, the expression level of MET in four cell lines, AURKB and PIM1 in three cell lines (MCF7, T47D and BCPAP), SYK in three cell lines (T47D, BCPAP and TPC1) was significantly decreased (P < 0.05). In addition, CDDP reduced the levels of phosphorylated AURKB, MET and PIM1 (pAURKB, pMET and pPIM1) in four cell lines (P < 0.05). However, the level of phosphorylated SYK (pSYK) in three cell lines (MCF7, BCPAP and TPC1) was significantly increased (P < 0.05).

## Discussion

TCM prescriptions are the characteristics of Chinese medicine, which embody the dialectical thought of Chinese medicine and the medication holistic view. As accumulating evidences have proved that the ingredients entering the blood, main metabolites, bioequivalence components compared to the prescription, and active components reported in literatures contribute more to the effects and mechanisms of TCM^16–18^, we raised the hypothesis that the potential targets of all the important components mentioned above should be more likely to become the direct targets of the whole prescriptions. In addition, we included another component reported the most in single herbs, quercetin as important component to finalize the list. This method improves the credibility of the data, which is different from most used network pharmacology research flowchart^19–21^. In addition, in order to obtain the potential target data for the important components in a more accurate way, we integrated the recorded data and predicted data. On one hand, the existing research results from open data have been fully utilized. On the other hand, algorithm models were used to predict potential targets to avoid missing some important targets. Moreover, kinase targets predicted by KinomeX platform were used to filter the kinase targets obtained by the above two methods, which can further improve the success rate of further verification.

Most research focused on active components extracted from Chinese herbals and other natural products at present. Among them, small molecule affinity chromatography and activity-based protein profiling (ABPP) are the most widely used target identification technologies for active ingredients, such as target fishing technology^22–27^. Using this strategy, a series of targets for active components of TCM have been successfully identified, including the targets identification of sumitone^28^ and chrysanthema lactone^29^. However, target fishing technology suits more for further in-depth analysis as low-through put experiment due to its excessive cost, which is not an efficient method to obtain direct targets broadly, especially for a whole prescription.

In this study, 106 potential kinase targets of CDDP were tested, and finally 30 active targets were obtained, with an accuracy of 28.3%. As expected, the success rate of the known kinase targets is higher than that of the predicted targets (40.5% vs 29.2%, shown in Table 4). The filter by KinomeX predictive results enables a higher success rate, which suggested that the strategy we built may serve as an efficient direct target predicting system for the other TCM prescriptions. However, in this study, only the algorithm based on structural similarity is used to predict the component target relationship. This method cannot distinguish the molecules with very similar structure, and the prediction results are often the same. However, the potency of a pair of molecules with similar structure will vary greatly^30,31^. In further study, a variety of state-of-art algorithms based on different principles should be utilized to predict component-target relationship for improving the accuracy of predicting results^32–36^, such as in silico models based on network topology parameters^33^, drug and target structure similarity^34^, clustering multi-dimensional drug target data^35^, deep learning and heterogeneous network^36^ etc. In addition, molecular docking technology can also be used to gain more reliable targets for further experimental verification^37,38^.

Among the 14 targets retested at the concentration of 250 µg/mL, the inhibitory activity of four kinases (CAMK2G, CSF1R, FYN and RET) did not decrease but increased at high concentration. The possible reasons may as follow: firstly, the components with high molecular weight in TCM form great stereo-hindrance effect when the concentration increases, which may hinder the combination between active molecules and targets. The second possibility is potential positive effectors involved in CDDP. The synergy weakens the affinity and internal effectiveness of the ligand on the receptors^39–43^. For example, both CDDP 12 (Rosmarinic acid) and CDDP 37 (Catechol) contained in CDDP could act on the common target FYN. However, the binding site for the two components may be different, which may bring the allosteric effect, weakening the inhibition effect under the condition of high concentration. These components may not directly bind to protein active sites, but to the allosteric sites, outside the active sites of the protein, causing the conformational change of proteins and their activity.

Three kinase targets (AURKB, MET and PIM1) of CDDP, that have been finally validated on cellular level, could provide basis for further elucidating the mechanism of CDDP in treating cardiovascular diseases^44,45^. For example, AURKB positively correlates with platelet aggregation and acute myocardial infarction (MI)^46^. MET shows repair function in cardiomyocytes and blood vessels through pro-angiogenesis, anti-inflammation and preventing fibrosis^47^. PIM1 plays a role in vascular smooth muscle cells (VSMCs) proliferation, which is closely related to the pathogenesis of atherosclerosis^48^. Besides, these targets are closely related to some other diseases^49–55^, indicating the potential function of CDDP against other indications, especially cancers. Actually, it has been reported the anti-tumor activity of several significant components of CDDP, including Danshensu^56^, Tanshinone I^57^, Cryptotanshinone^58^, Tanshinone IIA^59^, Rosmarinic acid^60^, and Ginsenoside Rg1^61^, suggesting the potential anti-tumor effect of CDDP.

Comparing with the above three kinases, it is worthy of note that CDDP promoted SYK activity in several cell lines (Figs. 3, 4), which showing an opposite trend with the kinase assays result (Fig. 2). One possible reason of such inconsistency could be the complexity of TCM prescriptions when treating with cells. When some components with weak affinity/activating effect on SYK entering the cells preferentially, while those with strong affinity/inhibitory effect on SYK being obstructed by cell membrane, CDDP exerted activating effect on SYK as a whole prescription on cellular level. All the kinase targets obtained in this study need to be verified in a variety of disease models in the follow-up studies, which can help to explain the mechanism of CDDP on the existing main indications, or expand the new indications of CDDP.

In conclusion, 30 direct targets of CDDP were obtained in this study by the strategy we built, which is independent of any specific disease model and can provide a series of potential direct targets of TCM efficiently. Moreover, this strategy takes TCM as a whole research object, which is in line with the holistic view and systematic theory of TCM, conforming to the guiding principles of pharmacology theory of TCM. The direct targets not only provide the theoretical basis for elucidating the mechanism of action and the material basis, but also indicating rationales for the research of drug repositioning, which is of great significance for promoting TCM modernization.

## Methods

### Construction of important component set for CDDP

In order to review the literatures related to CDDP as comprehensively as possible, we used "Danshen Dropping Pills" as the keyword to obtain the Chinese-language literatures through CNKI (https://www.cnki.net/). Similarly, through PubMed (https://pubmed.ncbi.nlm.nih.gov/), "Compound Danshen drilling pills", "Fufang Danshen Diwan", "T89", "dantonic" and "Cardiotonic Pills" were used to get the English-language literatures (time to April 15, 2020). Then, the components contained in CDDP were extracted manually and standardized through PubChem database (https://pubchem.ncbi.nlm.nih.gov/)^62^. Besides the ingredients entering the blood, main metabolites, bioequivalence components compared to the prescription, active components of CDDP reported in literatures, in order to avoid missing critical components included in CDDP, we selected the most extensively studied component in the three single herbs but still unconfirmed in the whole prescription, through retrieving TCM related databases, such as TcmSP^63^, TCMID^64^, TCM-ID^65^, ETCM^66^, and YaTCM^67^.

### Prediction of potential direct kinases targets of CDDP

Based on the hypothesis and research strategy, we followed the steps below to obtain the potential direct kinase targets of CDDP, as described in the flowchart (Fig. 1).

#### Targets of 40 compounds obtained by retrieving public databases

The known activity data of 40 important components in CDDP were obtained from three authoritative public databases, namely, ChEMBL^68^, PubChem^62^, BindingDB^69^. The targets with definite activity information were standardized by annotating the basic information, such as Gene Symbol, Entrez Gene Name, Location, and Type(s) through ingenuity knowledge base in Ingenuity Pathway Analysis (IPA) and subsequently the kinase targets were screened. It is a professional database of functional annotation and biological interaction, which collects millions of detailed annotation information about proteins, genes, compounds, cells, tissues, drugs and diseases, as well as their interaction information. All information was collected from the original literatures and reviewed by hundreds of doctoral experts to ensure its accuracy.

#### Targets of 40 compounds predicted by multi-voting SEA algorithm

Avoiding missing some important targets, multi-voting SEA algorithm^70^ was utilized to predict potential targets of important components. In this algorithm, prediction models, namely Topological SEA, Morgan SEA, MACCS SEA, Atom Pair SEA and Pharmacophore SEA, were integrated to calculate potential targets of components, which could take advantages of different models and improve the robustness and the success rates of the models. By combining the five models, a flexible forecasting scheme was obtained with precision range from 71 to 90.6%, F_0.5_-Measure range from 0.663 to 0.684 and F_0.25_-Measure range from 0.696 to 0.817. Finally, all potential targets of each component were normalized by IPA and the kinase targets were selected.

#### Kinase targets by predicted by KinomeX

KinomeX system (https://kinome.dddc.ac.cn/en/)^71^ is a prediction and analysis platform of single compound regulated kinase spectrum. It enables users to predict its potential kinase targets based on the structure of a given molecule with the average 0.75 area under the receiver operating characteristic curve (auROC), which is significantly higher than other prediction methods^72–78^. Therefore, we used the KinomeX to predict the potential protein kinase targets of 40 important components in CDDP.

### Potential direct kinase targets of CDDP

To obtain the kinase target set of CDDP with high reliability, the prediction results from KinomeX were used to screen the targets obtained from public databases and Multi-voting SEA algorithm mentioned above. The screened kinase targets were regarded as potential targets of CDDP and subsequently to conduct following experimental verification.

### Experimental validation in a high throughput way by Full KP panel

Full KP panel [km ATP], a kinase profiler, was developed by Eurofins company. In this study, we used this panel to carry out experimental verification for direct kinase targets of CDDP. CDDP was supplied by Tasly Modern Chinese Medicine Resources Co. Ltd. (Tianjin, China). Firstly, the filter-binding radioactive kinase activity assays were performed at a concentration of 25 µg/mL of CDDP. The kinase activity inhibition rate of the sample was expressed as the percentage of the result of sample compared to the blank group. The kinase activity of the blank was 100%. Generally speaking, if the residual enzyme activity is less than 30%, it is considered to be strongly inhibited. And if the residual enzyme activity is between 30 and 70%, it is considered as moderate inhibition. Considering the weak interaction superposition characteristic and synergistic effect of TCM ingredients^79,80^, the threshold value in this study was defined as 80%. In order to get the dose-dependent kinase targets, the kinase targets with activity value less than 70 were retested at a concentration of 250 µg/mL of CDDP.

### Kinase assays for AURKB, MET, PIM1, SYK Kinase analysis

To further obtain a mean IC_50_ value and its standard deviation, we chose four targets showing obvious inhibitory action at the concentration of 250 µg/mL to carry out the kinase assay. Pharmaron (Beijing) was commissioned to perform in vitro kinase assays for AURKB, MET, PIM1, SYK. The detailed information about the assays, such as the reagents, instruments, assay procedure, data analysis, and calculation of IC_50_, for AURKB, MET, PIM1, SYK can be referred in the attachment (see Supplementary Table S1). Ten concentration points were obtained by 3 dilution fold.

### Cell experiments in vitro for AURKB, MET, PIM1, SYK in four cell lines

#### Cell lines and treatments

The human breast cancer cell lines MCF7, T47D and thyroid cancer cell lines BCPAP, TPC1 used in this study were purchased from American Type Culture Collection (Manassas, VA, USA). These four cell lines were maintained in Dulbecco's modified Eagle medium (1640) (HyClone, UT, USA) supplemented with 10% fetal bovine serum (FBS) (Gibco, Gaithersburg, MD). All of them were placed in a 5% CO_2_ and humidified atmosphere at 37 °C. For treatments, each cell lines were divided into four groups as follows: Control group, CDDP group (0.25 mg/mL), CDDP group (0.5 mg/mL), and CDDP group (1.0 mg/mL). Cells at a density of 2 × 10^5^ cells/well in 6-well plates were treated with CDDP according to the groupings. The treatment time was 12 h for MCF7, T47D and 6 h for TPC1, BCPAP.

#### Western blot analysis

Cells were lysed in Triton-X 100 lysis buffer containing 1% (v/v) Triton-X 100, 150 mM NaCl, 50 mM Tris–HCl with protease and phosphatase inhibitors (10 µg/mL aprotinin, 10 µg/mL leupeptin, 1 µg/mL pepstatin, 5 mM sodium orthovanadate, 50 mM NaF, 50 mM Na-pyrophosphate, 150 µM phenylmethylsulfonyl fluoride). The cell lysates were separated by sodium dodecyl sulfate–polyacrylamide gel electrophoresis and then transferred to a polyvinyl difluoride membrane (Millipore, Bedford, MA, USA). The membranes were then blocked with 5% nonfat milk and incubated overnight with rabbit anti-Aurora B (1:1000, Cell signaling, #3094), rabbit anti-pAurora B (1:1000, Cell Signaling, #2914S, Thr232), mouse anti-MET (1:1000, Cell Signaling, #3127), rabbit anti-pMET (1:1000, Cell Signaling, #3077S, Tyr1234/1235), rabbit anti-Pim1 (1:1000, Cell Signaling, #3247S), rabbit anti-pPim1 (1:2000, Immunoway, #YP0331, Tyr309), rabbit anti-Syk (1:2000, Immunoway, #YT6110), and rabbit anti-pSyk (1:2000, Immunoway, #YP0615, Tyr525), respectively. Peroxidase-conjugated anti-mouse or anti-rabbit IgG (1:2000, Vector Laboratories, Burlingame, CA, USA) was used as the secondary antibody. Immunoblotting signals were detected using the ECL reagent (Millipore, Bedford, MA, USA). All membranes were re-probed with mouse anti-α-tubulin antibody (1:2000, Immunoway, #YM3035), which served as a loading control. Gray analysis of western blot image was performed using image analysis software (Image J 1.51j8; National Institutes of Health, Bethesda, MD, USA). Tanon 4600 SF and GelView 6000 Plus full-automatic digital gelatin/chemiluminescence imaging analysis system software is used for protein gel electrophoresis image acquisition.

### Statistical analysis

Statistical significance was determined by a two-tailed, unpaired Student t-test in GraphPad Prism 8. P-value < 0.05 was considered statistically significant.

## Supplementary Information

Supplementary Information.

## Data Availability

All data generated or analyzed during this study are included in this published article (and its Supplementary Information).

## Abbreviations

CDDP: Compound Danshen dropping pills
TCM: Traditional Chinese medicine
CAD: Coronary artery disease
SEA: Similarity ensemble approach
IC_50_: Half-maximal inhibitory concentration
ABPP: Activity-based protein profiling
IMPDH2: Inosine monophosphate dehydrogenase 2
HSP70: Heat shock protein 70
VSMCs: Vascular smooth muscle cells
IPA: Ingenuity pathway analysis
CHD: Coronary heart disease
auROC: Area under the receiver operating characteristic curve
