# Supplementary material for: Elucidating direct kinase targets of compound Danshen dropping pills employing archived data and prediction models
Source: Sci Rep. 2021 May 5;11:9541. doi: 10.1038/s41598-021-89035-4 (PMC8100098; doi:10.1038/s41598-021-89035-4)
Supplement: Supplementary file 1 — Supplementary Information. [file 41598_2021_89035_MOESM1_ESM.docx]

**Elucidating Direct Kinase Targets of Compound Danshen Dropping Pills Employing Archived Data and Prediction Models**

Tongxing Wang^1^, Lu Liang^2^, Chunlai Zhao^1^, Jia Sun^1^, Hairong Wang^1^, Wenjia Wang^1^, Jianping Lin^2^, Yunhui Hu^1*^

1. GeneNet Pharmaceuticals Co. Ltd., No.1, Tingjiang West Road, Beichen District, Tianjin 300410, China.

2. College of Pharmacy, Nankai University, 38 Tongyan Road, Haihe Education Park, Jinnan District, Tianjin 300353, China.

*** Correspondence:**

Yunhui Hu

tsl-huyunhui@tasly.com

**Supplementary information:**

**Supplementary information**

Figure S1. Inhibition curves of positive control compound (Danusertib, Cabozantinib, AZD1208 and Cerdulatinib), testing on AURKB, MET, PIM1 and SYK respectively

Figure S2. Full-length gels and blots of human breast cancer cell lines MCF7

Figure S3. Full-length gels and blots of human breast cancer cell lines T47D

Figure S4. Full-length gels and blots of human thyroid cancer cell lines BCPAP

Figure S5. Full-length gels and blots of human thyroid cancer cell lines TPC1

Table S1. Kinase assay procedure, reagents, and consumables

Table S2. Known and predicted targets of 40 important components and their relationships

Table S3. Predicted kinase targets of 40 important components by KinomeX and their relationships

Table S4. Potential kinase targets of CDDP

Table S5. Expression of pAURKB, pMET, pPIM1, pSYK, AURKB, MET, PIM1, and SYK protein levels in four cell lines


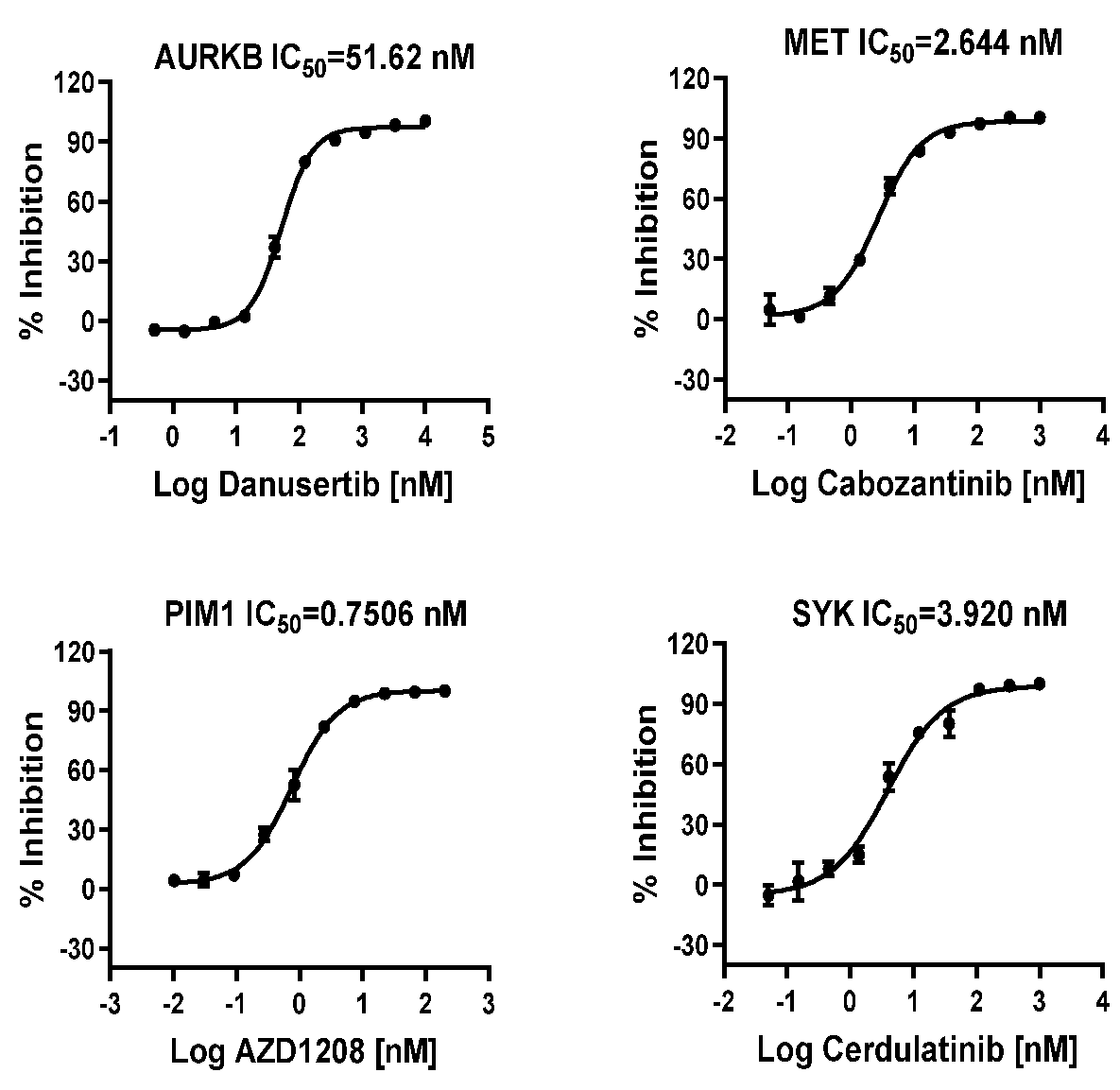


**Figure S1. Inhibition curves of positive control compound (Danusertib, Cabozantinib, AZD1208 and Cerdulatinib), testing on AURKB, MET, PIM1 and SYK respectively**


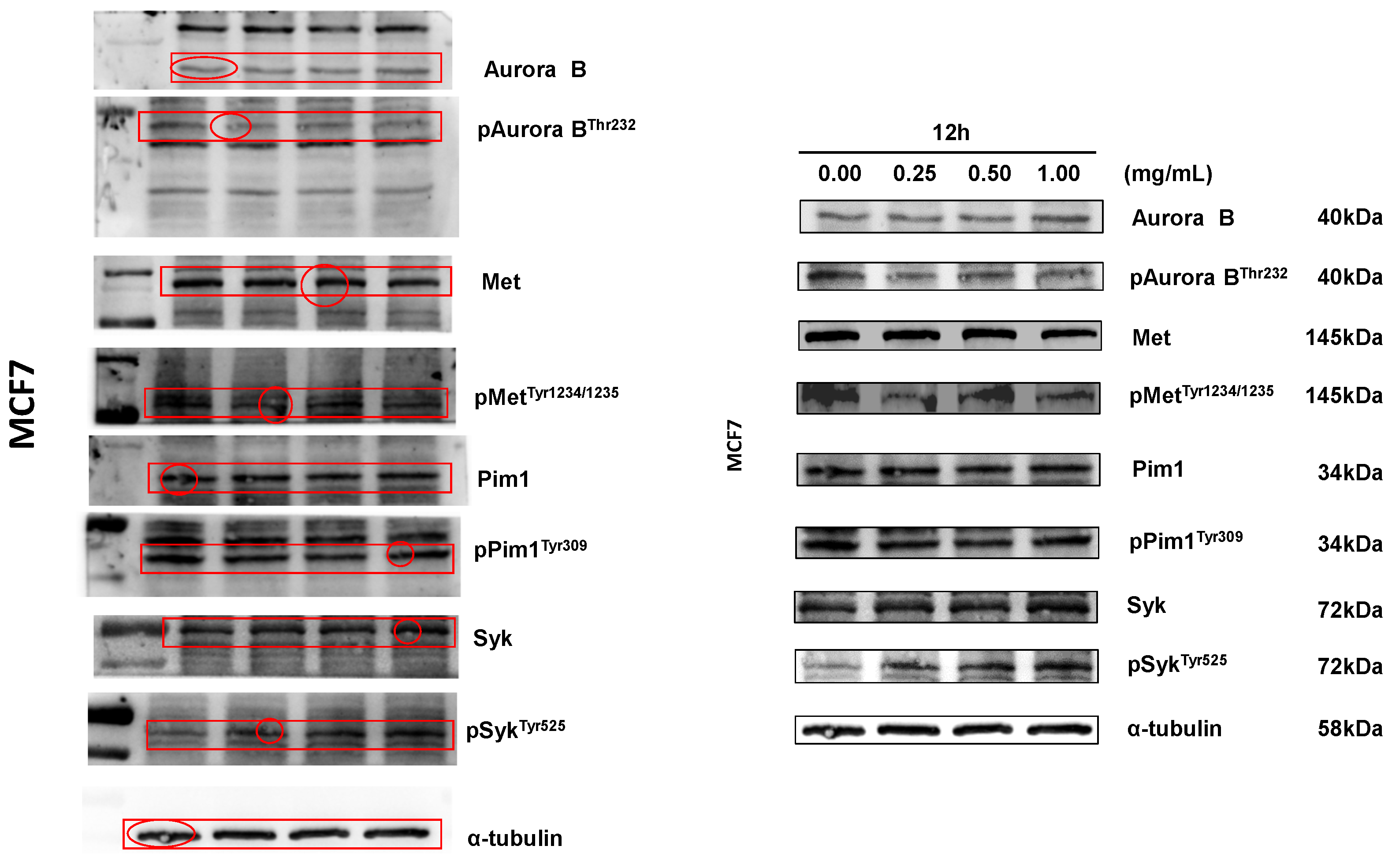


**Figure S2. Full-length gels and blots of human breast cancer cell lines MCF7**


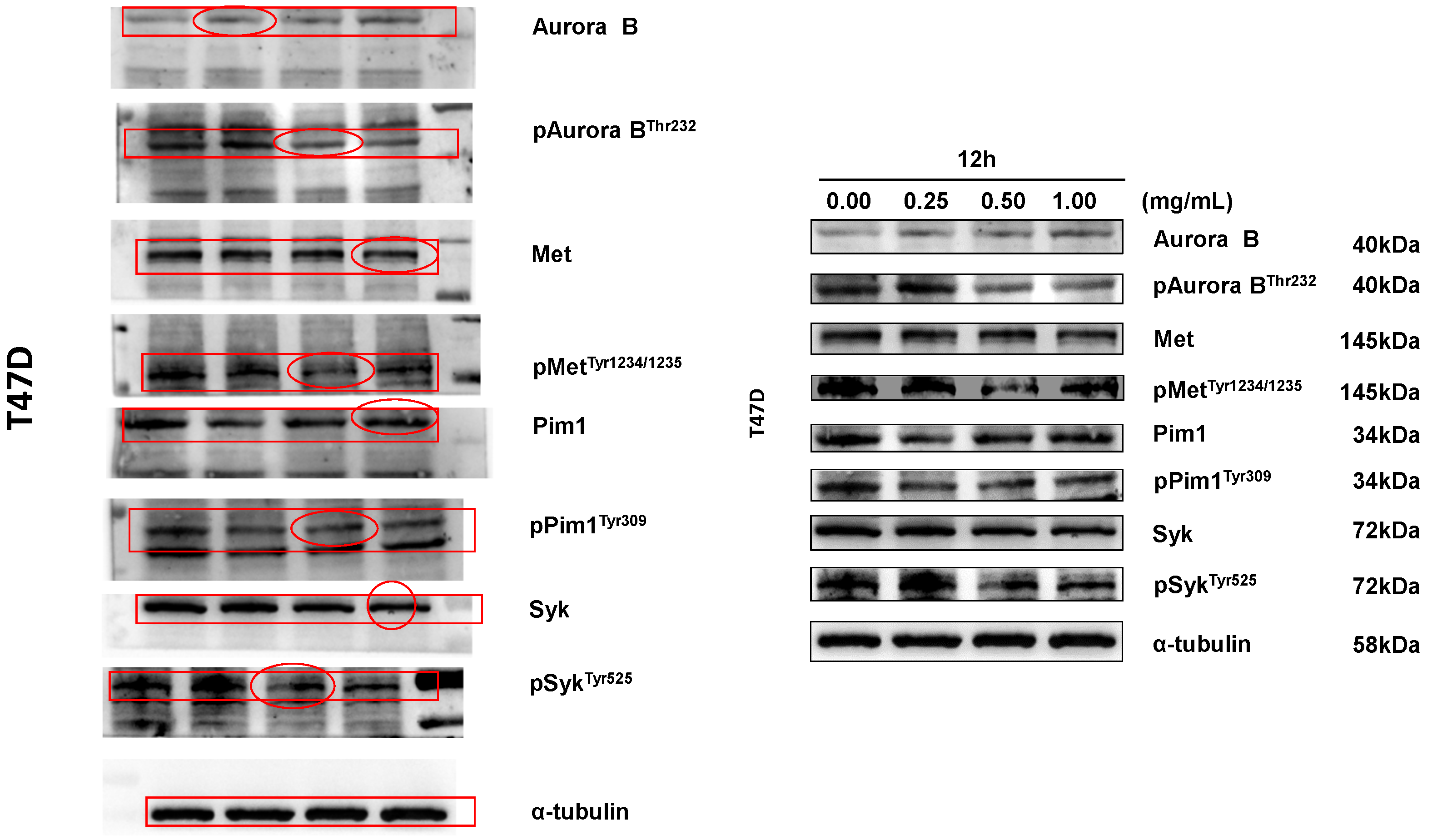


**Figure S3. Full-length gels and blots of human breast cancer cell lines T47D**


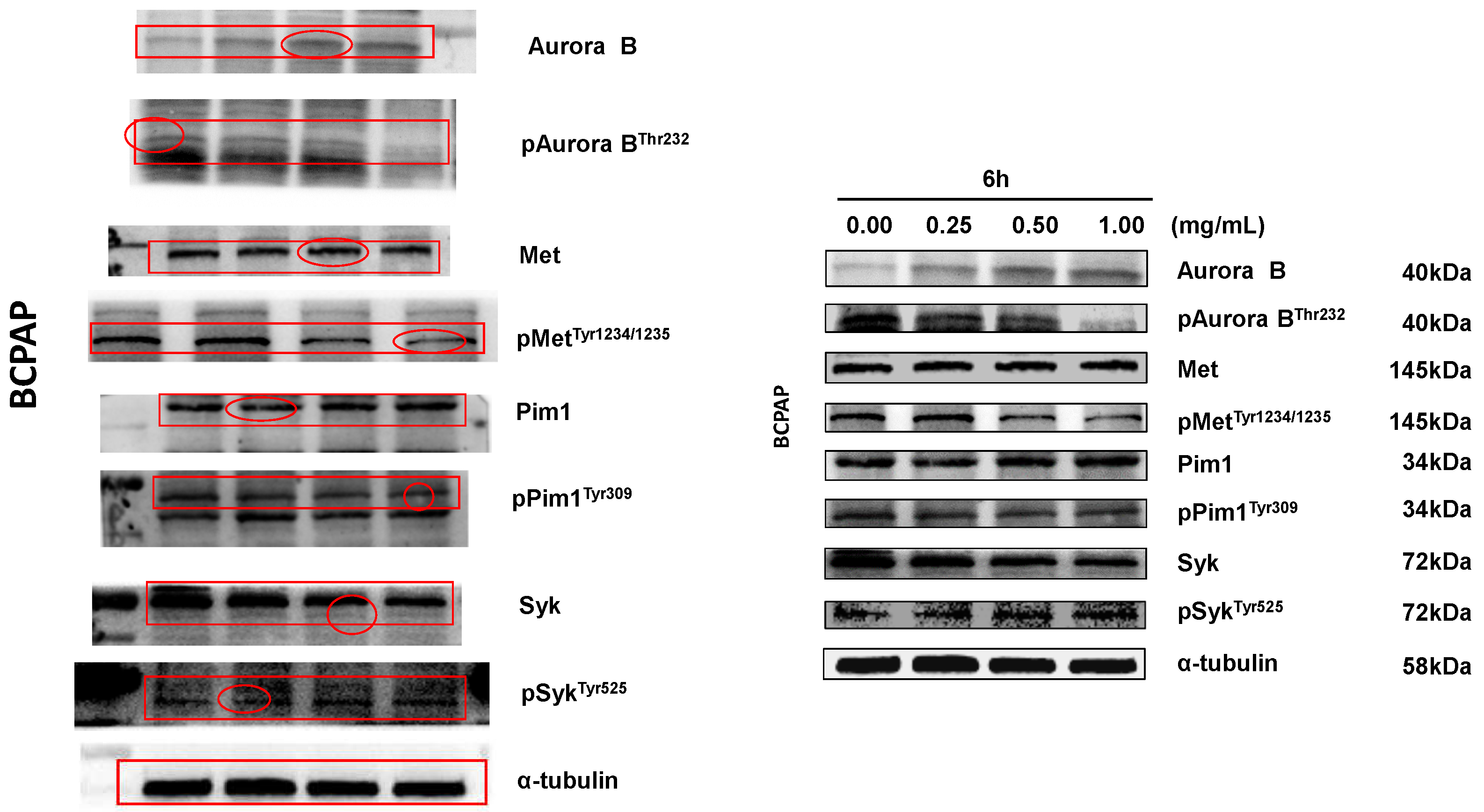


**Figure S4. Full-length gels and blots of human thyroid cancer cell lines BCPAP**


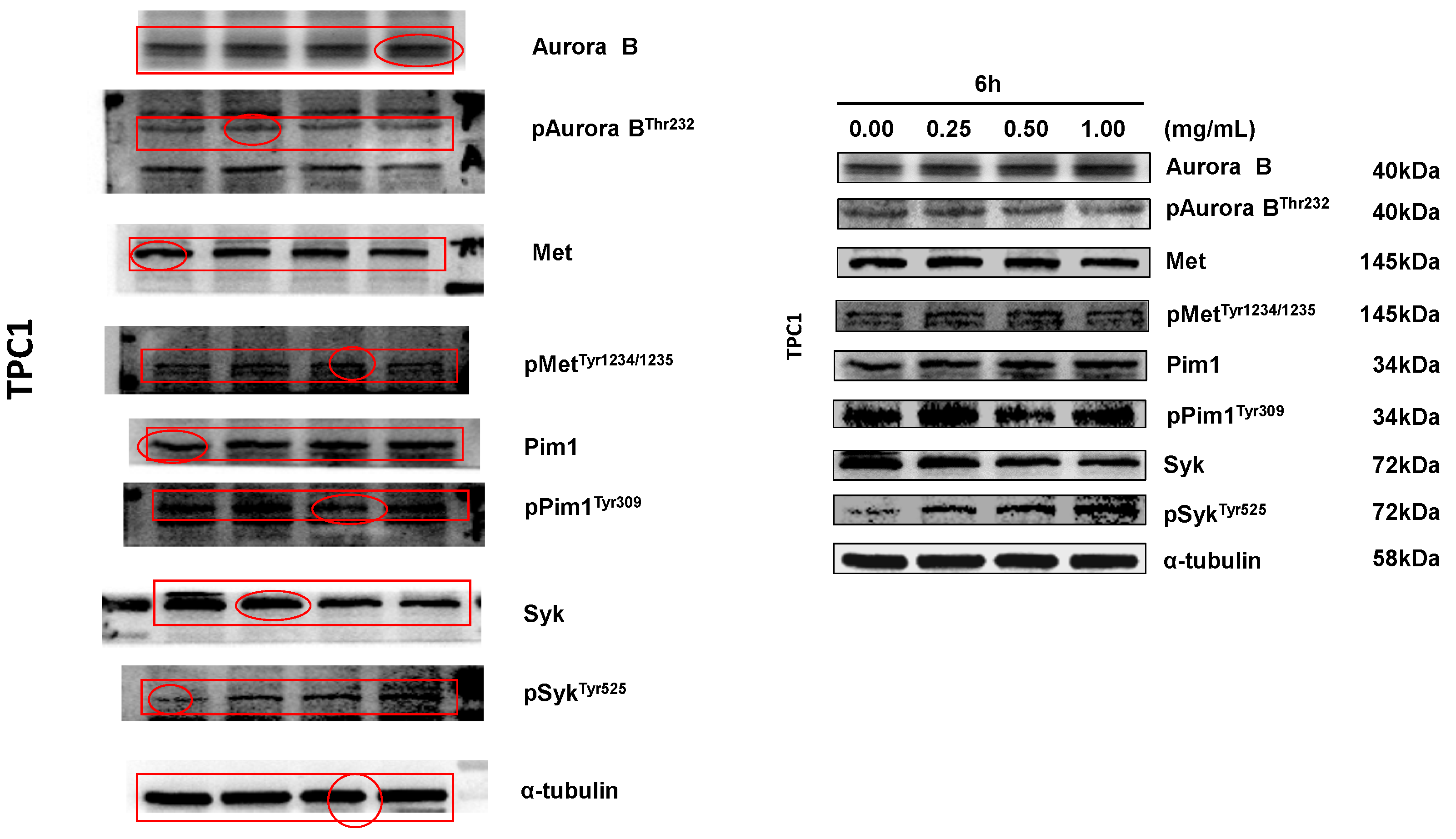


**Figure S5. Full-length gels and blots of human thyroid cancer cell lines TPC1**

**Table S1. Kinase assay procedure, reagents, and consumables**

**Kinase assay for AURKB**

1. Compound’s information

| **Entry** | **Cpd No.** | **Stock conc.** | **Solvent** | **Start Conc.** |
| --- | --- | --- | --- | --- |
| **1** | CDDP | 60 mg/ml | DMSO | 300 ug/ml |
| **Ref** | Danusertib |  |  | 10000 nM |

Notes: Danusertib was used as a positive control. All test samples were prepared in dimethyl sulfoxide (DMSO). The start concentration for Danusertib and CDDP was 10000 nM and 300 μg/ml, respectively. Ten concentration points were obtained by 3 dilution fold.

2. Assay Procedure

2.1 Preparation of 1x kinase Buffer: 1 volume of enzymatic buffer 5X with 4 volumes of distilled water; 5mM MgCl2; 1mM DTT.

2.2 Compound screening: a) Transfer compound dilutions into each well of assay plates (784075, Greiner) using Echo 550; b) Seal the assay plate, centrifuge compound plates at 1000g for 1min. c) Prepare 2X Aurora-B in 1x kinase buffer. d) Add 5μl of 2X Aurora-B into 384-well assay plate (784075, Greiner). e) Centrifuge plates at 1000g for 30s, RT for 10 min. f) Prepare 2x STK2-substrate-biotin and ATP mixture in 1X kinase buffer. g) Start the reaction by adding 5μl STK2-substrate-biotin and ATP (prepared at step g). h) Centrifuge plates at 1000g for 30s. Seal the assay plate, RT for 30min. i) Prepare 4X Sa-XL 665 in HTRF detection buffer. j) Add 5μl Sa-XL 665 and 5μl STK2-antibody-Cryptate (prepared at step i) into each well of the assay plate. k) Centrifuge plate at 1000g for 30s, RT for 1h. l) Read fluorescence signal at 615 nm (Cryptate) and 665 nm (XL665) on Envision 2104 plate reader.

3. Data analysis

3.1 A Signal (Ratio 665/615nm) is calculated for each well.

3.2 % Inhibition is calculated as follow: % inhibition =100-(Signalcompound-SignalAve_PC)/ (SignalAve_VC-SignalAve_PC) × 100.

3.3 Calculate IC50 and Plot effect-dose curve of compounds: Calculate IC50 by fitting % Inhibition values and log of compound concentrations to nonlinear regression (dose response – variable slope) with GraphPad 6.0.

Y=Bottom + (Top-Bottom)/(1+10^((LogIC50-X) * HillSlope))

X: log of inhibitor concentration; Y: % Inhibition.

| **Materials and Reagents** | **Vendor** | **Cat#** |
| --- | --- | --- |
| HTRF KinEASE-TK kit | Cisbio | 62TK0PEC |
| Aurora-B | Carna | 05-102 |
| MgCl2 | Sigma | M1028 |
| MnCl2 | Sigma | M1787 |
| ATP | Promega | V910B |
| DTT | Sigma | 48316 |
| DMSO | Sigma | D8418 |
| Danusertib | MCE | HY-15999 |
| **Consumables and Instrument** | **Vendor** | **Cat# or Model** |
| 384-well plate, white, low volume, round-bottom | Greiner | 784075 |
| 384-Well Polypropylene microplate, Clear, Flatt Bottom, Bar Code | Labcyte | P-05525-BC |
| 96-well polypropylene plate | Nunc | 249944 |
| Plate shaker | Thermo | 4625-1 CECN/THZ Q |
| Centrifuge | Eppendorf | 5810R |
| Envision 2104 multi-label Reader | PerkinElmer | Oct-04 |
| Echo | Labcyte | 550 |

**Kinase assay for MET**

1. Compound’s information

| **Entry** | **Cpd No.** | **Stock conc.** | **Solvent** | **Start Conc.** |
| --- | --- | --- | --- | --- |
| **1** | CDDP | 200mg/ml | DMSO | 1mg/ml |
| **Ref** | Cabozantinib | 10mM | DMSO | 1000nM |

Notes: Cabozantinib was used as a positive control. All test samples were prepared in dimethyl sulfoxide (DMSO). The start concentration for Cabozantinib and CDDP was 1000 nM and 1000 μg/ml, respectively. Ten concentration points were obtained by 3 dilution fold.

2. Assay Procedure

2.1 Preparation of 1x kinase Buffer: 1 volume of enzymatic buffer 5X with 4 volumes of distilled water; 5mM MgCl2; 1mM MnCl2; 1mM DTT.

2.2 Compound screening: a) Transfer compound dilutions into each well of assay plates (784075, Greiner) using Echo 550; b) Seal the assay plate, centrifuge compound plates at 1000g for 1min. c) Prepare 2X MET in 1x kinase buffer. d) Add 5μl of 2X MET into 384-well assay plate (784075, Greiner). e) Centrifuge plates at 1000g for 30s, RT for 10 min. f) Prepare 2x TK-substrate-biotin and ATP mixture in 1X kinase buffer. g) Start the reaction by adding 5μl TK-substrate-biotin and ATP (prepared at step g). h) Centrifuge plates at 1000g for 30s. Seal the assay plate, RT for 30min. i) Prepare 4X Sa-XL 665 in HTRF detection buffer. j) Add 5μl Sa-XL 665 and 5μl TK-antibody-Cryptate (prepared at step i) into each well of the assay plate. k) Centrifuge plate at 1000g for 30s, RT for 1h. l) Read fluorescence signal at 615 nm (Cryptate) and 665 nm (XL665) on Envision 2104 plate reader.

3. Data analysis

3.1 A Signal (Ratio 665/615nm) is calculated for each well.

3.2 % Inhibition is calculated as follow: % inhibition =100-(Signalcompound - SignalAve_PC)/ (SignalAve_VC - SignalAve_PC) × 100.

3.3 Calculate IC50 and Plot effect-dose curve of compounds: Calculate IC50 by fitting % Inhibition values and log of compound concentrations to nonlinear regression (dose response – variable slope) with GraphPad 6.0.

Y=Bottom + (Top-Bottom)/(1+10^((LogIC50-X) * HillSlope))

X: log of inhibitor concentration; Y: % Inhibition.

| **Materials and Reagents** | **Vendor** | **Cat#** |
| --- | --- | --- |
| HTRF KinEASE-TK kit | Cisbio | 62TK0PEC |
| MET | Carna | 08-151 |
| MgCl2 | Sigma | M1028 |
| MnCl2 | Sigma | M1787 |
| ATP | Promega | V910B |
| DTT | Sigma | 48316 |
| DMSO | Sigma | D8418 |
| Cabozantinib | MCE | HY-13016 |
| **Consumables and Instrument** | **Vendor** | **Cat# or Model** |
| 384-well plate, white, low volume, round-bottom | Greiner | 784075 |
| 384-Well Polypropylene microplate,Clear, Flatt Bottom,Bar Code | Labcyte | P-05525-BC |
| 96-well polypropylene plate | Nunc | 249944 |
| Plate shaker | Thermo | 4625-1 CECN/THZ Q |
| Centrifuge | Eppendorf | 5810R |
| Envision 2104 multi-label Reader | PerkinElmer | Oct-04 |
| Echo | Labcyte | 550 |

**Kinase assay for PIM1**

1. Compound’s information

| **Entry** | **Cpd No.** | **Stock conc.** | **Solvent** | **Start Conc.** |
| --- | --- | --- | --- | --- |
| **1** | CDDP | 200mg/ml | DMSO | 1mg/ml |
| **Ref** | AZD1208 | 10mM | DMSO | 200nM |

Notes: AZD1208 was used as a positive control. All test samples were prepared in dimethyl sulfoxide (DMSO). The start concentration for AZD1208 and CDDP was 200 nM and 1000 μg/ml, respectively. Ten concentration points were obtained by 3 dilution fold.

2. Assay Procedure

2.1 Preparation of 1x kinase Buffer: 1 volume of enzymatic buffer 5X with 4 volumes of distilled water; 5mM MgCl2; 1mM DTT.

2.2 Compound screening: a) Transfer compound dilutions into each well of assay plates (784075, Greiner) using Echo 550; b) Seal the assay plate, centrifuge compound plates at 1000g for 1min. c) Prepare 2X PIM1 in 1x kinase buffer. d) Add 5μl of 2X PIM1 into 384-well assay plate (784075, Greiner). e) Centrifuge plates at 1000g for 30s, RT for 10 min. f) Prepare 2x STK3-substrate-biotin and ATP mixture in 1X kinase buffer g) Start the reaction by adding 5μl STK3-substrate-biotin and ATP (prepared at step g). h) Centrifuge plates at 1000g for 30s. Seal the assay plate, RT for 30min. i) Prepare 4X Sa-XL 665 in HTRF detection buffer. j) Add 5μl Sa-XL 665 and 5μl STK3-antibody-Cryptate (prepared at step i) into each well of the assay plate. k) Centrifuge plate at 1000g for 30s, RT for 1h. l) Read fluorescence signal at 615 nm (Cryptate) and 665 nm (XL665) on Envision 2104 plate reader.

3. Data analysis

3.1 A Signal (Ratio 665/615nm) is calculated for each well.

3.2 % Inhibition is calculated as follow: % inhibition =100-(Signalcompound - SignalAve_PC)/ (SignalAve_VC - SignalAve_PC) × 100.

3.3 Calculate IC50 and Plot effect-dose curve of Compounds: Calculate IC50 by fitting % Inhibition values and log of compound concentrations to nonlinear regression (dose response – variable slope) with GraphPad 6.0.

Y=Bottom + (Top-Bottom)/(1+10^((LogIC50-X) * HillSlope))

X: log of inhibitor concentration; Y: % Inhibition.

| **Materials and Reagents** | **Vendor** | **Cat#** |
| --- | --- | --- |
| HTRF KinEASE-TK kit | Cisbio | 62TK0PEC |
| PIM1 | Carna | 02-054 |
| MgCl2 | Sigma | M1028 |
| MnCl2 | Sigma | M1787 |
| ATP | Promega | V910B |
| DTT | Sigma | 48316 |
| DMSO | Sigma | D8418 |
| AZD1208 | Selleck | S7104 |
| **Consumables and Instrument** | **Vendor** | **Cat# or Model** |
| 384-well plate, white, low volume, round-bottom | Greiner | 784075 |
| 384-Well Polypropylene microplate,Clear, Flatt Bottom,Bar Code | Labcyte | P-05525-BC |
| 96-well polypropylene plate | Nunc | 249944 |
| Plate shaker | Thermo | 4625-1 CECN/THZ Q |
| Centrifuge | Eppendorf | 5810R |
| Envision 2104 multi-label Reader | PerkinElmer | Oct-04 |
| Echo | Labcyte | 550 |

**Kinase assay for SYK**

1. Compound’s information

| **Entry** | **Cpd No.** | **Stock conc.** | **Solvent** | **Start Conc.** |
| --- | --- | --- | --- | --- |
| **1** | CDDP | 200mg/ml | DMSO | 1mg/ml |
| **Ref** | Cerdulatinib |  |  | 1uM |

Notes: Cerdulatinib was used as a positive control. All test samples were prepared in dimethyl sulfoxide (DMSO). The start concentration for Cerdulatinib and CDDP was 1000 nM and 1000 μg/ml, respectively. Ten concentration points were obtained by 3 dilution fold.

2. Assay Procedure

2.1 Preparation of 1x kinase Buffer: 1 volume of enzymatic buffer 5X with 4 volumes of distilled water; 5mM MgCl2; 1mM MnCl2; 1mM DTT.

2.2 Compound screening: a) Transfer compound dilutions into each well of assay plates (784075, Greiner) using Echo 550; b) Seal the assay plate, centrifuge compound plates at 1000g for 1min. c) Prepare 2X SYK in 1x kinase buffer. d) Add 5μl of 2X SYK into 384-well assay plate (784075, Greiner). e) Centrifuge plates at 1000g for 30s, RT for 10 min. f) Prepare 2x TK-substrate-biotin and ATP mixture in 1X kinase buffer. g) Start the reaction by adding 5μl TK-substrate-biotin and ATP (prepared at step g). h) Centrifuge plates at 1000g for 30s. Seal the assay plate, RT for 30min. i) Prepare 4X Sa-XL 665 in HTRF detection buffer. j) Add 5μl Sa-XL 665 and 5μl TK-antibody-Cryptate (prepared at step i) into each well of the assay plate. k) Centrifuge plate at 1000g for 30s, RT for 1h. l) Read fluorescence signal at 615 nm (Cryptate) and 665 nm (XL665) on Envision 2104 plate reader.

3. Data analysis

3.1 A Signal (Ratio 665/615nm) is calculated for each well.

3.2 % Inhibition is calculated as follow: % inhibition =100-(Signalcompound - SignalAve_PC)/ (SignalAve_VC - SignalAve_PC) × 100.

3.3 Calculate IC50 and Plot effect-dose curve of compounds:

Calculate IC50 by fitting % Inhibition values and log of compound concentrations to nonlinear regression (dose response – variable slope) with GraphPad 6.0.

Y=Bottom + (Top-Bottom)/(1+10^((LogIC50-X) * HillSlope))

X: log of inhibitor concentration; Y: % Inhibition.

| **Materials and Reagents** | **Vendor** | **Cat#** |
| --- | --- | --- |
| HTRF KinEASE-TK kit | Cisbio | 62TK0PEC |
| SYK | Carna | 08-176 |
| MgCl2 | Sigma | M1028 |
| MnCl2 | Sigma | M1787 |
| ATP | Promega | V910B |
| DTT | Sigma | 48316 |
| DMSO | Sigma | D8418 |
| Cerdulatinib | MCE | HY-15999 |
| **Consumables and Instrument** | **Vendor** | **Cat# or Model** |
| 384-well plate, white, low volume, round-bottom | Greiner | 784075 |
| 384-Well Polypropylene microplate,Clear, Flatt Bottom,Bar Code | Labcyte | P-05525-BC |
| 96-well polypropylene plate | Nunc | 249944 |
| Plate shaker | Thermo | 4625-1 CECN/THZ Q |
| Centrifuge | Eppendorf | 5810R |
| Envision 2104 multi-label Reader | PerkinElmer | Oct-04 |
| Echo | Labcyte | 550 |

**Table S2. Known and predicted targets of 40 important components and their relationships**

| **Molecule ID** | **Gene name** | **Source** | **Entrez Gene Name** | **Location** | **Type(s)** |
| --- | --- | --- | --- | --- | --- |
| CDDP 02 | MAPT | Known interactions | microtubule associated protein tau | Plasma Membrane | other |
| CDDP 02 | TDP1 | Known interactions | tyrosyl-DNA phosphodiesterase 1 | Nucleus | enzyme |
| CDDP 02 | GMNN | Known interactions | geminin DNA replication inhibitor | Nucleus | transcription regulator |
| CDDP 02 | BAZ2B | Known interactions | bromodomain adjacent to zinc finger domain 2B | Extracellular Space | other |
| CDDP 02 | POLB | Known interactions | DNA polymerase beta | Nucleus | enzyme |
| CDDP 02 | KDM4A | Known interactions | lysine demethylase 4A | Nucleus | transcription regulator |
| CDDP 02 | POLH | Known interactions | DNA polymerase eta | Nucleus | enzyme |
| CDDP 02 | POLK | Known interactions | DNA polymerase kappa | Nucleus | enzyme |
| CDDP 02 | ATXN2 | Known interactions | ataxin 2 | Nucleus | other |
| CDDP 02 | USP1 | Known interactions | ubiquitin specific peptidase 1 | Cytoplasm | peptidase |
| CDDP 02 | RAPGEF3 | Known interactions | Rap guanine nucleotide exchange factor 3 | Nucleus | other |
| CDDP 03 | MAPT | Known interactions | microtubule associated protein tau | Plasma Membrane | other |
| CDDP 03 | PTPN11 | Known interactions | protein tyrosine phosphatase non-receptor type 11 | Cytoplasm | phosphatase |
| CDDP 03 | ALDH1A1 | Known interactions | aldehyde dehydrogenase 1 family member A1 | Cytoplasm | enzyme |
| CDDP 03 | POLB | Known interactions | DNA polymerase beta | Nucleus | enzyme |
| CDDP 03 | KDM4A | Known interactions | lysine demethylase 4A | Nucleus | transcription regulator |
| CDDP 03 | BAZ2B | Known interactions | bromodomain adjacent to zinc finger domain 2B | Extracellular Space | other |
| CDDP 03 | VDR | Known interactions | vitamin D receptor | Nucleus | transcription regulator |
| CDDP 03 | POLI | Known interactions | DNA polymerase iota | Nucleus | enzyme |
| CDDP 03 | POLH | Known interactions | DNA polymerase eta | Nucleus | enzyme |
| CDDP 03 | SMAD3 | Known interactions | SMAD family member 3 | Nucleus | transcription regulator |
| CDDP 03 | GMNN | Known interactions | geminin DNA replication inhibitor | Nucleus | transcription regulator |
| CDDP 03 | TDP1 | Known interactions | tyrosyl-DNA phosphodiesterase 1 | Nucleus | enzyme |
| CDDP 03 | USP1 | Known interactions | ubiquitin specific peptidase 1 | Cytoplasm | peptidase |
| CDDP 03 | PLK1 | Known interactions | polo like kinase 1 | Nucleus | kinase |
| CDDP 03 | RAPGEF3 | Known interactions | Rap guanine nucleotide exchange factor 3 | Nucleus | other |
| CDDP 04 | APAF1 | Known interactions | apoptotic peptidase activating factor 1 | Cytoplasm | other |
| CDDP 04 | ALDH1A1 | Known interactions | aldehyde dehydrogenase 1 family member A1 | Cytoplasm | enzyme |
| CDDP 04 | HSD17B10 | Known interactions | hydroxysteroid 17-beta dehydrogenase 10 | Cytoplasm | enzyme |
| CDDP 04 | MAPT | Known interactions | microtubule associated protein tau | Plasma Membrane | other |
| CDDP 04 | HPGD | Known interactions | 15-hydroxyprostaglandin dehydrogenase | Cytoplasm | enzyme |
| CDDP 04 | POLB | Known interactions | DNA polymerase beta | Nucleus | enzyme |
| CDDP 04 | KDM4A | Known interactions | lysine demethylase 4A | Nucleus | transcription regulator |
| CDDP 04 | KAT2A | Known interactions | lysine acetyltransferase 2A | Cytoplasm | enzyme |
| CDDP 04 | EHMT2 | Known interactions | euchromatic histone lysine methyltransferase 2 | Nucleus | transcription regulator |
| CDDP 04 | BAZ2B | Known interactions | bromodomain adjacent to zinc finger domain 2B | Extracellular Space | other |
| CDDP 04 | VDR | Known interactions | vitamin D receptor | Nucleus | transcription regulator |
| CDDP 04 | POLH | Known interactions | DNA polymerase eta | Nucleus | enzyme |
| CDDP 04 | AR | Known interactions | androgen receptor | Nucleus | ligand-dependent nuclear receptor |
| CDDP 04 | TDP1 | Known interactions | tyrosyl-DNA phosphodiesterase 1 | Nucleus | enzyme |
| CDDP 05 | MAPT | Known interactions | microtubule associated protein tau | Plasma Membrane | other |
| CDDP 05 | VDR | Known interactions | vitamin D receptor | Nucleus | transcription regulator |
| CDDP 11 | KDM4E | Known interactions | lysine demethylase 4E | Nucleus | enzyme |
| CDDP 11 | KDM4A | Known interactions | lysine demethylase 4A | Nucleus | transcription regulator |
| CDDP 11 | BAZ2B | Known interactions | bromodomain adjacent to zinc finger domain 2B | Extracellular Space | other |
| CDDP 12 | APEX1 | Known interactions | apurinic/apyrimidinic endodeoxyribonuclease 1 | Nucleus | enzyme |
| CDDP 12 | RECQL | Known interactions | RecQ like helicase | Nucleus | enzyme |
| CDDP 12 | POLB | Known interactions | DNA polymerase beta | Nucleus | enzyme |
| CDDP 12 | MCL1 | Known interactions | MCL1 apoptosis regulator, BCL2 family member | Cytoplasm | transporter |
| CDDP 12 | MAPT | Known interactions | microtubule associated protein tau | Plasma Membrane | other |
| CDDP 12 | THRB | Known interactions | thyroid hormone receptor beta | Nucleus | ligand-dependent nuclear receptor |
| CDDP 12 | BLM | Known interactions | BLM RecQ like helicase | Nucleus | enzyme |
| CDDP 12 | BAZ2B | Known interactions | bromodomain adjacent to zinc finger domain 2B | Extracellular Space | other |
| CDDP 16 | SIRT1 | Known interactions | sirtuin 1 | Nucleus | transcription regulator |
| CDDP 22 | KDM4E | Known interactions | lysine demethylase 4E | Nucleus | enzyme |
| CDDP 22 | HSD17B10 | Known interactions | hydroxysteroid 17-beta dehydrogenase 10 | Cytoplasm | enzyme |
| CDDP 22 | GAA | Known interactions | glucosidase alpha, acid | Cytoplasm | enzyme |
| CDDP 22 | EHMT2 | Known interactions | euchromatic histone lysine methyltransferase 2 | Nucleus | transcription regulator |
| CDDP 22 | AKR1B10 | Known interactions | aldo-keto reductase family 1 member B10 | Cytoplasm | enzyme |
| CDDP 35 | ABCB1 | Known interactions | ATP binding cassette subfamily B member 1 | Plasma Membrane | transporter |
| CDDP 36 | PSMB5 | Known interactions | proteasome subunit beta 5 | Cytoplasm | peptidase |
| CDDP 36 | GAA | Known interactions | glucosidase alpha, acid | Cytoplasm | enzyme |
| CDDP 36 | POLB | Known interactions | DNA polymerase beta | Nucleus | enzyme |
| CDDP 37 | HSD17B10 | Known interactions | hydroxysteroid 17-beta dehydrogenase 10 | Cytoplasm | enzyme |
| CDDP 37 | MTOR | Known interactions | mechanistic target of rapamycin kinase | Nucleus | kinase |
| CDDP 37 | CA1 | Known interactions | carbonic anhydrase 1 | Cytoplasm | enzyme |
| CDDP 37 | RECQL | Known interactions | RecQ like helicase | Nucleus | enzyme |
| CDDP 37 | KDM4E | Known interactions | lysine demethylase 4E | Nucleus | enzyme |
| CDDP 37 | ALDH1A1 | Known interactions | aldehyde dehydrogenase 1 family member A1 | Cytoplasm | enzyme |
| CDDP 37 | ALOX15 | Known interactions | arachidonate 15-lipoxygenase | Cytoplasm | enzyme |
| CDDP 37 | HIF1A | Known interactions | hypoxia inducible factor 1 subunit alpha | Nucleus | transcription regulator |
| CDDP 37 | L3MBTL1 | Known interactions | L3MBTL histone methyl-lysine binding protein 1 | Nucleus | other |
| CDDP 37 | APEX1 | Known interactions | apurinic/apyrimidinic endodeoxyribonuclease 1 | Nucleus | enzyme |
| CDDP 37 | HPGD | Known interactions | 15-hydroxyprostaglandin dehydrogenase | Cytoplasm | enzyme |
| CDDP 37 | POLB | Known interactions | DNA polymerase beta | Nucleus | enzyme |
| CDDP 37 | KDM4A | Known interactions | lysine demethylase 4A | Nucleus | transcription regulator |
| CDDP 37 | MPHOSPH8 | Known interactions | M-phase phosphoprotein 8 | Nucleus | transcription regulator |
| CDDP 37 | EHMT2 | Known interactions | euchromatic histone lysine methyltransferase 2 | Nucleus | transcription regulator |
| CDDP 37 | FEN1 | Known interactions | flap structure-specific endonuclease 1 | Nucleus | enzyme |
| CDDP 37 | ESR1 | Known interactions | estrogen receptor 1 | Nucleus | ligand-dependent nuclear receptor |
| CDDP 37 | POLI | Known interactions | DNA polymerase iota | Nucleus | enzyme |
| CDDP 37 | POLK | Known interactions | DNA polymerase kappa | Nucleus | enzyme |
| CDDP 37 | NR3C1 | Known interactions | nuclear receptor subfamily 3 group C member 1 | Nucleus | ligand-dependent nuclear receptor |
| CDDP 37 | NFE2L2 | Known interactions | nuclear factor, erythroid 2 like 2 | Nucleus | transcription regulator |
| CDDP 37 | RORC | Known interactions | RAR related orphan receptor C | Nucleus | ligand-dependent nuclear receptor |
| CDDP 02 | APAF1 | Known interactions | apoptotic peptidase activating factor 1 | Cytoplasm | other |
| CDDP 02 | EIF4H | Known interactions | eukaryotic translation initiation factor 4H | Cytoplasm | translation regulator |
| CDDP 02 | PABPC1 | Known interactions | poly(A) binding protein cytoplasmic 1 | Cytoplasm | translation regulator |
| CDDP 02 | DNMT1 | Known interactions | DNA methyltransferase 1 | Nucleus | enzyme |
| CDDP 02 | PLA2G7 | Known interactions | phospholipase A2 group VII | Extracellular Space | enzyme |
| CDDP 02 | MAP4K2 | Known interactions | mitogen-activated protein kinase kinase kinase kinase 2 | Cytoplasm | kinase |
| CDDP 02 | GSTO1 | Known interactions | glutathione S-transferase omega 1 | Cytoplasm | enzyme |
| CDDP 02 | GAPDH | Known interactions | glyceraldehyde-3-phosphate dehydrogenase | Cytoplasm | enzyme |
| CDDP 02 | MDM2 | Known interactions | MDM2 proto-oncogene | Nucleus | transcription regulator |
| CDDP 02 | MDM4 | Known interactions | MDM4 regulator of p53 | Nucleus | transcription regulator |
| CDDP 02 | PAFAH2 | Known interactions | platelet activating factor acetylhydrolase 2 | Cytoplasm | enzyme |
| CDDP 02 | FASN | Known interactions | fatty acid synthase | Cytoplasm | enzyme |
| CDDP 02 | TNFRSF10B | Known interactions | TNF receptor superfamily member 10b | Plasma Membrane | transmembrane receptor |
| CDDP 02 | PIP4K2A | Known interactions | phosphatidylinositol-5-phosphate 4-kinase type 2 alpha | Cytoplasm | kinase |
| CDDP 02 | IFNG | Known interactions | interferon gamma | Extracellular Space | cytokine |
| CDDP 03 | STAT1 | Known interactions | signal transducer and activator of transcription 1 | Nucleus | transcription regulator |
| CDDP 03 | GSTO1 | Known interactions | glutathione S-transferase omega 1 | Cytoplasm | enzyme |
| CDDP 03 | PLA2G7 | Known interactions | phospholipase A2 group VII | Extracellular Space | enzyme |
| CDDP 03 | MITF | Known interactions | melanocyte inducing transcription factor | Nucleus | transcription regulator |
| CDDP 03 | PAFAH2 | Known interactions | platelet activating factor acetylhydrolase 2 | Cytoplasm | enzyme |
| CDDP 03 | PAFAH1B3 | Known interactions | platelet activating factor acetylhydrolase 1b catalytic subunit 3 | Cytoplasm | enzyme |
| CDDP 03 | UCHL5 | Known interactions | ubiquitin C-terminal hydrolase L5 | Cytoplasm | peptidase |
| CDDP 03 | CFTR | Known interactions | CF transmembrane conductance regulator | Plasma Membrane | ion channel |
| CDDP 03 | KCNK3 | Known interactions | potassium two pore domain channel subfamily K member 3 | Plasma Membrane | ion channel |
| CDDP 03 | PIP4K2A | Known interactions | phosphatidylinositol-5-phosphate 4-kinase type 2 alpha | Cytoplasm | kinase |
| CDDP 04 | SENP8 | Known interactions | SUMO peptidase family member, NEDD8 specific | Cytoplasm | peptidase |
| CDDP 04 | CASP9 | Known interactions | caspase 9 | Cytoplasm | peptidase |
| CDDP 04 | CASP3 | Known interactions | caspase 3 | Cytoplasm | peptidase |
| CDDP 04 | EIF4H | Known interactions | eukaryotic translation initiation factor 4H | Cytoplasm | translation regulator |
| CDDP 04 | PABPC1 | Known interactions | poly(A) binding protein cytoplasmic 1 | Cytoplasm | translation regulator |
| CDDP 04 | STK33 | Known interactions | serine/threonine kinase 33 | Cytoplasm | kinase |
| CDDP 04 | GAPDH | Known interactions | glyceraldehyde-3-phosphate dehydrogenase | Cytoplasm | enzyme |
| CDDP 04 | PLA2G7 | Known interactions | phospholipase A2 group VII | Extracellular Space | enzyme |
| CDDP 04 | MDM2 | Known interactions | MDM2 proto-oncogene | Nucleus | transcription regulator |
| CDDP 04 | MDM4 | Known interactions | MDM4 regulator of p53 | Nucleus | transcription regulator |
| CDDP 04 | MITF | Known interactions | melanocyte inducing transcription factor | Nucleus | transcription regulator |
| CDDP 04 | PAFAH1B2 | Known interactions | platelet activating factor acetylhydrolase 1b catalytic subunit 2 | Cytoplasm | enzyme |
| CDDP 04 | PAFAH2 | Known interactions | platelet activating factor acetylhydrolase 2 | Cytoplasm | enzyme |
| CDDP 04 | DAGLB | Known interactions | diacylglycerol lipase beta | Plasma Membrane | enzyme |
| CDDP 04 | DNMT1 | Known interactions | DNA methyltransferase 1 | Nucleus | enzyme |
| CDDP 04 | CFTR | Known interactions | CF transmembrane conductance regulator | Plasma Membrane | ion channel |
| CDDP 04 | FASN | Known interactions | fatty acid synthase | Cytoplasm | enzyme |
| CDDP 04 | PIP4K2A | Known interactions | phosphatidylinositol-5-phosphate 4-kinase type 2 alpha | Cytoplasm | kinase |
| CDDP 05 | GAPDH | Known interactions | glyceraldehyde-3-phosphate dehydrogenase | Cytoplasm | enzyme |
| CDDP 06 | LCK | Known interactions | LCK proto-oncogene, Src family tyrosine kinase | Cytoplasm | kinase |
| CDDP 06 | SRC | Known interactions | SRC proto-oncogene, non-receptor tyrosine kinase | Cytoplasm | kinase |
| CDDP 11 | GFER | Known interactions | growth factor, augmenter of liver regeneration | Nucleus | enzyme |
| CDDP 11 | IL1B | Known interactions | interleukin 1 beta | Extracellular Space | cytokine |
| CDDP 12 | RGS12 | Known interactions | regulator of G protein signaling 12 | Nucleus | enzyme |
| CDDP 12 | GNAI1 | Known interactions | G protein subunit alpha i1 | Plasma Membrane | enzyme |
| CDDP 12 | EIF4H | Known interactions | eukaryotic translation initiation factor 4H | Cytoplasm | translation regulator |
| CDDP 12 | UBA2 | Known interactions | ubiquitin like modifier activating enzyme 2 | Cytoplasm | enzyme |
| CDDP 12 | SAE1 | Known interactions | SUMO1 activating enzyme subunit 1 | Cytoplasm | enzyme |
| CDDP 12 | UBE2I | Known interactions | ubiquitin conjugating enzyme E2 I | Nucleus | enzyme |
| CDDP 12 | PABPC1 | Known interactions | poly(A) binding protein cytoplasmic 1 | Cytoplasm | translation regulator |
| CDDP 12 | GAPDH | Known interactions | glyceraldehyde-3-phosphate dehydrogenase | Cytoplasm | enzyme |
| CDDP 12 | MITF | Known interactions | melanocyte inducing transcription factor | Nucleus | transcription regulator |
| CDDP 22 | GLS | Known interactions | glutaminase | Cytoplasm | enzyme |
| CDDP 22 | HSP90AB1 | Known interactions | heat shock protein 90 alpha family class B member 1 | Cytoplasm | enzyme |
| CDDP 22 | HSP90AA1 | Known interactions | heat shock protein 90 alpha family class A member 1 | Cytoplasm | enzyme |
| CDDP 22 | ESR2 | Known interactions | estrogen receptor 2 | Nucleus | ligand-dependent nuclear receptor |
| CDDP 22 | ALPL | Known interactions | alkaline phosphatase, biomineralization associated | Plasma Membrane | phosphatase |
| CDDP 22 | MCL1 | Known interactions | MCL1 apoptosis regulator, BCL2 family member | Cytoplasm | transporter |
| CDDP 22 | MPI | Known interactions | mannose phosphate isomerase | Cytoplasm | enzyme |
| CDDP 22 | GAPDH | Known interactions | glyceraldehyde-3-phosphate dehydrogenase | Cytoplasm | enzyme |
| CDDP 22 | GFER | Known interactions | growth factor, augmenter of liver regeneration | Nucleus | enzyme |
| CDDP 36 | GFER | Known interactions | growth factor, augmenter of liver regeneration | Nucleus | enzyme |
| CDDP 36 | GALR3 | Known interactions | galanin receptor 3 | Plasma Membrane | G-protein coupled receptor |
| CDDP 37 | GLI3 | Known interactions | GLI family zinc finger 3 | Nucleus | transcription regulator |
| CDDP 37 | GLS | Known interactions | glutaminase | Cytoplasm | enzyme |
| CDDP 37 | ESRRA | Known interactions | estrogen related receptor alpha | Nucleus | ligand-dependent nuclear receptor |
| CDDP 37 | ESR2 | Known interactions | estrogen receptor 2 | Nucleus | ligand-dependent nuclear receptor |
| CDDP 37 | NR1I2 | Known interactions | nuclear receptor subfamily 1 group I member 2 | Nucleus | ligand-dependent nuclear receptor |
| CDDP 37 | CYP19A1 | Known interactions | cytochrome P450 family 19 subfamily A member 1 | Cytoplasm | enzyme |
| CDDP 37 | AHR | Known interactions | aryl hydrocarbon receptor | Nucleus | ligand-dependent nuclear receptor |
| CDDP 37 | HSP90AB1 | Known interactions | heat shock protein 90 alpha family class B member 1 | Cytoplasm | enzyme |
| CDDP 37 | HSP90AA1 | Known interactions | heat shock protein 90 alpha family class A member 1 | Cytoplasm | enzyme |
| CDDP 37 | NR1I3 | Known interactions | nuclear receptor subfamily 1 group I member 3 | Nucleus | ligand-dependent nuclear receptor |
| CDDP 37 | SMAD2 | Known interactions | SMAD family member 2 | Nucleus | transcription regulator |
| CDDP 37 | SMAD3 | Known interactions | SMAD family member 3 | Nucleus | transcription regulator |
| CDDP 40 | CA2 | Known interactions | carbonic anhydrase 2 | Cytoplasm | enzyme |
| CDDP 40 | CA7 | Known interactions | carbonic anhydrase 7 | Cytoplasm | enzyme |
| CDDP 40 | CA1 | Known interactions | carbonic anhydrase 1 | Cytoplasm | enzyme |
| CDDP 40 | CA3 | Known interactions | carbonic anhydrase 3 | Cytoplasm | enzyme |
| CDDP 40 | CA6 | Known interactions | carbonic anhydrase 6 | Extracellular Space | enzyme |
| CDDP 40 | CA12 | Known interactions | carbonic anhydrase 12 | Plasma Membrane | enzyme |
| CDDP 40 | CA14 | Known interactions | carbonic anhydrase 14 | Plasma Membrane | enzyme |
| CDDP 40 | CA9 | Known interactions | carbonic anhydrase 9 | Nucleus | enzyme |
| CDDP 40 | CA4 | Known interactions | carbonic anhydrase 4 | Plasma Membrane | enzyme |
| CDDP 40 | CA5A | Known interactions | carbonic anhydrase 5A | Cytoplasm | enzyme |
| CDDP 40 | HPGD | Known interactions | 15-hydroxyprostaglandin dehydrogenase | Cytoplasm | enzyme |
| CDDP 40 | ALOX15 | Known interactions | arachidonate 15-lipoxygenase | Cytoplasm | enzyme |
| CDDP 40 | CYP1A2 | Known interactions | cytochrome P450 family 1 subfamily A member 2 | Cytoplasm | enzyme |
| CDDP 40 | CYP2C9 | Known interactions | cytochrome P450 family 2 subfamily C member 9 | Cytoplasm | enzyme |
| CDDP 40 | TDP1 | Known interactions | tyrosyl-DNA phosphodiesterase 1 | Nucleus | enzyme |
| CDDP 40 | BLM | Known interactions | BLM RecQ like helicase | Nucleus | enzyme |
| CDDP 40 | NFKB1 | Known interactions | nuclear factor kappa B subunit 1 | Nucleus | transcription regulator |
| CDDP 40 | CA5B | Known interactions | carbonic anhydrase 5B | Cytoplasm | enzyme |
| CDDP 40 | CYP19A1 | Known interactions | cytochrome P450 family 19 subfamily A member 1 | Cytoplasm | enzyme |
| CDDP 40 | CYP1B1 | Known interactions | cytochrome P450 family 1 subfamily B member 1 | Cytoplasm | enzyme |
| CDDP 40 | F2 | Known interactions | coagulation factor II, thrombin | Extracellular Space | peptidase |
| CDDP 40 | PIM1 | Known interactions | Pim-1 proto-oncogene, serine/threonine kinase | Cytoplasm | kinase |
| CDDP 40 | SRC | Known interactions | SRC proto-oncogene, non-receptor tyrosine kinase | Cytoplasm | kinase |
| CDDP 40 | POLB | Known interactions | DNA polymerase beta | Nucleus | enzyme |
| CDDP 40 | POLK | Known interactions | DNA polymerase kappa | Nucleus | enzyme |
| CDDP 40 | KDR | Known interactions | kinase insert domain receptor | Plasma Membrane | kinase |
| CDDP 40 | XDH | Known interactions | xanthine dehydrogenase | Cytoplasm | enzyme |
| CDDP 40 | IGF1R | Known interactions | insulin like growth factor 1 receptor | Plasma Membrane | transmembrane receptor |
| CDDP 40 | ALOX12 | Known interactions | arachidonate 12-lipoxygenase, 12S type | Cytoplasm | enzyme |
| CDDP 40 | CDK1 | Known interactions | cyclin dependent kinase 1 | Nucleus | kinase |
| CDDP 40 | CSNK2B | Known interactions | casein kinase 2 beta | Cytoplasm | kinase |
| CDDP 40 | CSNK2A1 | Known interactions | casein kinase 2 alpha 1 | Nucleus | kinase |
| CDDP 40 | CSNK2A2 | Known interactions | casein kinase 2 alpha 2 | Cytoplasm | kinase |
| CDDP 40 | MET | Known interactions | MET proto-oncogene, receptor tyrosine kinase | Plasma Membrane | kinase |
| CDDP 40 | FLT3 | Known interactions | fms related tyrosine kinase 3 | Plasma Membrane | kinase |
| CDDP 40 | CYP1A1 | Known interactions | cytochrome P450 family 1 subfamily A member 1 | Cytoplasm | enzyme |
| CDDP 40 | NOX4 | Known interactions | NADPH oxidase 4 | Cytoplasm | enzyme |
| CDDP 40 | ALOX5 | Known interactions | arachidonate 5-lipoxygenase | Cytoplasm | enzyme |
| CDDP 40 | POLH | Known interactions | DNA polymerase eta | Nucleus | enzyme |
| CDDP 40 | EGFR | Known interactions | epidermal growth factor receptor | Plasma Membrane | kinase |
| CDDP 40 | AURKB | Known interactions | aurora kinase B | Nucleus | kinase |
| CDDP 40 | AXL | Known interactions | AXL receptor tyrosine kinase | Plasma Membrane | kinase |
| CDDP 40 | PTK2 | Known interactions | protein tyrosine kinase 2 | Cytoplasm | kinase |
| CDDP 40 | MPO | Known interactions | myeloperoxidase | Cytoplasm | enzyme |
| CDDP 40 | CYP2C8 | Known interactions | cytochrome P450 family 2 subfamily C member 8 | Cytoplasm | enzyme |
| CDDP 40 | HSD17B2 | Known interactions | hydroxysteroid 17-beta dehydrogenase 2 | Cytoplasm | enzyme |
| CDDP 40 | MMP9 | Known interactions | matrix metallopeptidase 9 | Extracellular Space | peptidase |
| CDDP 40 | PLA2G1B | Known interactions | phospholipase A2 group IB | Extracellular Space | enzyme |
| CDDP 40 | GSK3B | Known interactions | glycogen synthase kinase 3 beta | Nucleus | kinase |
| CDDP 40 | GSK3A | Known interactions | glycogen synthase kinase 3 alpha | Nucleus | kinase |
| CDDP 40 | CYP3A4 | Known interactions | cytochrome P450 family 3 subfamily A member 4 | Cytoplasm | enzyme |
| CDDP 40 | FEN1 | Known interactions | flap structure-specific endonuclease 1 | Nucleus | enzyme |
| CDDP 40 | ABCC1 | Known interactions | ATP binding cassette subfamily C member 1 | Plasma Membrane | transporter |
| CDDP 40 | MAPT | Known interactions | microtubule associated protein tau | Plasma Membrane | other |
| CDDP 40 | MAOA | Known interactions | monoamine oxidase A | Cytoplasm | enzyme |
| CDDP 40 | CAMK2B | Known interactions | calcium/calmodulin dependent protein kinase II beta | Cytoplasm | kinase |
| CDDP 40 | GLO1 | Known interactions | glyoxalase I | Cytoplasm | enzyme |
| CDDP 40 | ALK | Known interactions | ALK receptor tyrosine kinase | Plasma Membrane | kinase |
| CDDP 40 | PIK3R1 | Known interactions | phosphoinositide-3-kinase regulatory subunit 1 | Cytoplasm | kinase |
| CDDP 40 | DRD1 | Known interactions | dopamine receptor D1 | Plasma Membrane | G-protein coupled receptor |
| CDDP 40 | NEK6 | Known interactions | NIMA related kinase 6 | Nucleus | kinase |
| CDDP 40 | PYGL | Known interactions | glycogen phosphorylase L | Cytoplasm | enzyme |
| CDDP 40 | CXCR1 | Known interactions | C-X-C motif chemokine receptor 1 | Plasma Membrane | G-protein coupled receptor |
| CDDP 40 | NUAK1 | Known interactions | NUAK family kinase 1 | Nucleus | kinase |
| CDDP 40 | AKT1 | Known interactions | AKT serine/threonine kinase 1 | Cytoplasm | kinase |
| CDDP 40 | BACE1 | Known interactions | beta-secretase 1 | Cytoplasm | peptidase |
| CDDP 40 | MMP3 | Known interactions | matrix metallopeptidase 3 | Extracellular Space | peptidase |
| CDDP 40 | NEK2 | Known interactions | NIMA related kinase 2 | Cytoplasm | kinase |
| CDDP 40 | PLK1 | Known interactions | polo like kinase 1 | Nucleus | kinase |
| CDDP 40 | PKN1 | Known interactions | protein kinase N1 | Cytoplasm | kinase |
| CDDP 40 | GPR35 | Known interactions | G protein-coupled receptor 35 | Plasma Membrane | G-protein coupled receptor |
| CDDP 40 | AVPR2 | Known interactions | arginine vasopressin receptor 2 | Plasma Membrane | G-protein coupled receptor |
| CDDP 40 | MMP2 | Known interactions | matrix metallopeptidase 2 | Extracellular Space | peptidase |
| CDDP 40 | ABCG2 | Known interactions | ATP binding cassette subfamily G member 2 (Junior blood group) | Plasma Membrane | transporter |
| CDDP 40 | ABCB1 | Known interactions | ATP binding cassette subfamily B member 1 | Plasma Membrane | transporter |
| CDDP 40 | DRD4 | Known interactions | dopamine receptor D4 | Plasma Membrane | G-protein coupled receptor |
| CDDP 40 | MMP13 | Known interactions | matrix metallopeptidase 13 | Extracellular Space | peptidase |
| CDDP 40 | DAPK1 | Known interactions | death associated protein kinase 1 | Cytoplasm | kinase |
| CDDP 40 | HIF1A | Known interactions | hypoxia inducible factor 1 subunit alpha | Nucleus | transcription regulator |
| CDDP 40 | CCR4 | Known interactions | C-C motif chemokine receptor 4 | Plasma Membrane | G-protein coupled receptor |
| CDDP 40 | HIF1AN | Known interactions | hypoxia inducible factor 1 subunit alpha inhibitor | Nucleus | enzyme |
| CDDP 40 | MMP12 | Known interactions | matrix metallopeptidase 12 | Extracellular Space | peptidase |
| CDDP 40 | CYP2C19 | Known interactions | cytochrome P450 family 2 subfamily C member 19 | Cytoplasm | enzyme |
| CDDP 40 | ESR1 | Known interactions | estrogen receptor 1 | Nucleus | ligand-dependent nuclear receptor |
| CDDP 40 | PLAU | Known interactions | plasminogen activator, urokinase | Extracellular Space | peptidase |
| CDDP 40 | ALOX15B | Known interactions | arachidonate 15-lipoxygenase type B | Cytoplasm | enzyme |
| CDDP 40 | ALDH1A1 | Known interactions | aldehyde dehydrogenase 1 family member A1 | Cytoplasm | enzyme |
| CDDP 40 | LCK | Known interactions | LCK proto-oncogene, Src family tyrosine kinase | Cytoplasm | kinase |
| CDDP 40 | PREP | Known interactions | prolyl endopeptidase | Cytoplasm | peptidase |
| CDDP 40 | PRSS1 | Known interactions | serine protease 1 | Cytoplasm | peptidase |
| CDDP 40 | PRSS2 | Known interactions | serine protease 2 | Extracellular Space | peptidase |
| CDDP 40 | PRSS3 | Known interactions | serine protease 3 | Extracellular Space | peptidase |
| CDDP 40 | ESRRA | Known interactions | estrogen related receptor alpha | Nucleus | ligand-dependent nuclear receptor |
| CDDP 40 | PIK3CG | Known interactions | phosphatidylinositol-4,5-bisphosphate 3-kinase catalytic subunit gamma | Cytoplasm | kinase |
| CDDP 40 | AR | Known interactions | androgen receptor | Nucleus | ligand-dependent nuclear receptor |
| CDDP 40 | PGR | Known interactions | progesterone receptor | Nucleus | ligand-dependent nuclear receptor |
| CDDP 40 | APP | Known interactions | amyloid beta precursor protein | Plasma Membrane | other |
| CDDP 40 | YES1 | Known interactions | YES proto-oncogene 1, Src family tyrosine kinase | Cytoplasm | kinase |
| CDDP 40 | IFNG | Known interactions | interferon gamma | Extracellular Space | cytokine |
| CDDP 40 | RXRA | Known interactions | retinoid X receptor alpha | Nucleus | ligand-dependent nuclear receptor |
| CDDP 40 | CYP2D6 | Known interactions | cytochrome P450 family 2 subfamily D member 6 | Cytoplasm | enzyme |
| CDDP 40 | CCNB1 | Known interactions | cyclin B1 | Cytoplasm | kinase |
| CDDP 40 | SYK | Known interactions | spleen associated tyrosine kinase | Cytoplasm | kinase |
| CDDP 40 | ATAD5 | Known interactions | ATPase family AAA domain containing 5 | Nucleus | enzyme |
| CDDP 40 | HDAC9 | Known interactions | histone deacetylase 9 | Nucleus | transcription regulator |
| CDDP 40 | CDK5R1 | Known interactions | cyclin dependent kinase 5 regulatory subunit 1 | Nucleus | kinase |
| CDDP 40 | CDK5 | Known interactions | cyclin dependent kinase 5 | Nucleus | kinase |
| CDDP 40 | PTPN1 | Known interactions | protein tyrosine phosphatase non-receptor type 1 | Cytoplasm | phosphatase |
| CDDP 40 | CDK6 | Known interactions | cyclin dependent kinase 6 | Nucleus | kinase |
| CDDP 40 | PTGS2 | Known interactions | prostaglandin-endoperoxide synthase 2 | Cytoplasm | enzyme |
| CDDP 40 | ADORA3 | Known interactions | adenosine A3 receptor | Plasma Membrane | G-protein coupled receptor |
| CDDP 40 | DPP4 | Known interactions | dipeptidyl peptidase 4 | Plasma Membrane | peptidase |
| CDDP 40 | EPHX2 | Known interactions | epoxide hydrolase 2 | Cytoplasm | enzyme |
| CDDP 40 | SORD | Known interactions | sorbitol dehydrogenase | Cytoplasm | enzyme |
| CDDP 40 | AHR | Known interactions | aryl hydrocarbon receptor | Nucleus | ligand-dependent nuclear receptor |
| CDDP 40 | CD38 | Known interactions | CD38 molecule | Plasma Membrane | enzyme |
| CDDP 40 | ACHE | Known interactions | acetylcholinesterase (Cartwright blood group) | Plasma Membrane | enzyme |
| CDDP 40 | MPHOSPH8 | Known interactions | M-phase phosphoprotein 8 | Nucleus | transcription regulator |
| CDDP 40 | CDK2 | Known interactions | cyclin dependent kinase 2 | Nucleus | kinase |
| CDDP 40 | GALK1 | Known interactions | galactokinase 1 | Cytoplasm | kinase |
| CDDP 40 | TP53 | Known interactions | tumor protein p53 | Nucleus | transcription regulator |
| CDDP 40 | NR3C1 | Known interactions | nuclear receptor subfamily 3 group C member 1 | Nucleus | ligand-dependent nuclear receptor |
| CDDP 40 | NR1I2 | Known interactions | nuclear receptor subfamily 1 group I member 2 | Nucleus | ligand-dependent nuclear receptor |
| CDDP 40 | RNASEH1 | Known interactions | ribonuclease H1 | Nucleus | enzyme |
| CDDP 40 | CLK1 | Known interactions | CDC like kinase 1 | Nucleus | kinase |
| CDDP 40 | CLK3 | Known interactions | CDC like kinase 3 | Nucleus | kinase |
| CDDP 40 | CSNK1G2 | Known interactions | casein kinase 1 gamma 2 | Cytoplasm | kinase |
| CDDP 40 | YWHAB | Known interactions | tyrosine 3-monooxygenase/tryptophan 5-monooxygenase activation protein beta | Cytoplasm | other |
| CDDP 40 | DIRAS1 | Known interactions | DIRAS family GTPase 1 | Plasma Membrane | enzyme |
| CDDP 40 | STK16 | Known interactions | serine/threonine kinase 16 | Cytoplasm | kinase |
| CDDP 40 | HSP90AB1 | Known interactions | heat shock protein 90 alpha family class B member 1 | Cytoplasm | enzyme |
| CDDP 40 | HSP90AA1 | Known interactions | heat shock protein 90 alpha family class A member 1 | Cytoplasm | enzyme |
| CDDP 40 | CSNK1G1 | Known interactions | casein kinase 1 gamma 1 | Cytoplasm | kinase |
| CDDP 40 | SLK | Known interactions | STE20 like kinase | Nucleus | kinase |
| CDDP 40 | MAP3K5 | Known interactions | mitogen-activated protein kinase kinase kinase 5 | Cytoplasm | kinase |
| CDDP 40 | PIM2 | Known interactions | Pim-2 proto-oncogene, serine/threonine kinase | Nucleus | kinase |
| CDDP 40 | TOP2A | Known interactions | DNA topoisomerase II alpha | Nucleus | enzyme |
| CDDP 40 | HSPB1 | Known interactions | heat shock protein family B (small) member 1 | Cytoplasm | other |
| CDDP 40 | HSF1 | Known interactions | heat shock transcription factor 1 | Nucleus | transcription regulator |
| CDDP 40 | TOP1 | Known interactions | DNA topoisomerase I | Nucleus | enzyme |
| CDDP 40 | GCK | Known interactions | glucokinase | Cytoplasm | kinase |
| CDDP 40 | CALM3 | Known interactions | Calmodulin 3 | plasma membrane | Kinase |
| CDDP 40 | PIK3CA | Known interactions | phosphatidylinositol-4,5-bisphosphate 3-kinase catalytic subunit alpha | Cytoplasm | kinase |
| CDDP 40 | EZH2 | Known interactions | enhancer of zeste 2 polycomb repressive complex 2 subunit | Nucleus | transcription regulator |
| CDDP 40 | PON1 | Known interactions | paraoxonase 1 | Extracellular Space | phosphatase |
| CDDP 40 | PGD | Known interactions | phosphogluconate dehydrogenase | Cytoplasm | enzyme |
| CDDP 40 | NFKB2 | Known interactions | nuclear factor kappa B subunit 2 | Nucleus | transcription regulator |
| CDDP 40 | RELA | Known interactions | RELA proto-oncogene, NF-kB subunit | Nucleus | transcription regulator |
| CDDP 40 | AKR1B10 | Known interactions | aldo-keto reductase family 1 member B10 | Cytoplasm | enzyme |
| CDDP 40 | SMAD2 | Known interactions | SMAD family member 2 | Nucleus | transcription regulator |
| CDDP 40 | SMAD3 | Known interactions | SMAD family member 3 | Nucleus | transcription regulator |
| CDDP 02 | MAPT | Known interactions | microtubule associated protein tau | Plasma Membrane | other |
| CDDP 02 | PTPN6 | Known interactions | protein tyrosine phosphatase non-receptor type 6 | Cytoplasm | phosphatase |
| CDDP 02 | CES2 | Known interactions | carboxylesterase 2 | Cytoplasm | enzyme |
| CDDP 02 | PTPN11 | Known interactions | protein tyrosine phosphatase non-receptor type 11 | Cytoplasm | phosphatase |
| CDDP 02 | TDP1 | Known interactions | tyrosyl-DNA phosphodiesterase 1 | Nucleus | enzyme |
| CDDP 02 | BLM | Known interactions | BLM RecQ like helicase | Nucleus | enzyme |
| CDDP 02 | GMNN | Known interactions | geminin DNA replication inhibitor | Nucleus | transcription regulator |
| CDDP 02 | BAZ2B | Known interactions | bromodomain adjacent to zinc finger domain 2B | Extracellular Space | other |
| CDDP 02 | CES1 | Known interactions | carboxylesterase 1 | Cytoplasm | enzyme |
| CDDP 02 | POLB | Known interactions | DNA polymerase beta | Nucleus | enzyme |
| CDDP 02 | TERT | Known interactions | telomerase reverse transcriptase | Nucleus | enzyme |
| CDDP 02 | TP53 | Known interactions | tumor protein p53 | Nucleus | transcription regulator |
| CDDP 02 | ABCB1 | Known interactions | ATP binding cassette subfamily B member 1 | Plasma Membrane | transporter |
| CDDP 02 | BRCA1 | Known interactions | BRCA1 DNA repair associated | Nucleus | transcription regulator |
| CDDP 03 | CES1 | Known interactions | carboxylesterase 1 | Cytoplasm | enzyme |
| CDDP 03 | CES2 | Known interactions | carboxylesterase 2 | Cytoplasm | enzyme |
| CDDP 03 | STAT3 | Known interactions | signal transducer and activator of transcription 3 | Nucleus | transcription regulator |
| CDDP 03 | MAPT | Known interactions | microtubule associated protein tau | Plasma Membrane | other |
| CDDP 03 | ACHE | Known interactions | acetylcholinesterase (Cartwright blood group) | Plasma Membrane | enzyme |
| CDDP 03 | TERT | Known interactions | telomerase reverse transcriptase | Nucleus | enzyme |
| CDDP 03 | PTPN6 | Known interactions | protein tyrosine phosphatase non-receptor type 6 | Cytoplasm | phosphatase |
| CDDP 03 | PTPN1 | Known interactions | protein tyrosine phosphatase non-receptor type 1 | Cytoplasm | phosphatase |
| CDDP 03 | PTPN11 | Known interactions | protein tyrosine phosphatase non-receptor type 11 | Cytoplasm | phosphatase |
| CDDP 04 | APAF1 | Known interactions | apoptotic peptidase activating factor 1 | Cytoplasm | other |
| CDDP 04 | AKR1B1 | Known interactions | aldo-keto reductase family 1 member B | Cytoplasm | enzyme |
| CDDP 04 | CES1 | Known interactions | carboxylesterase 1 | Cytoplasm | enzyme |
| CDDP 04 | TERT | Known interactions | telomerase reverse transcriptase | Nucleus | enzyme |
| CDDP 04 | PTPN6 | Known interactions | protein tyrosine phosphatase non-receptor type 6 | Cytoplasm | phosphatase |
| CDDP 04 | CES2 | Known interactions | carboxylesterase 2 | Cytoplasm | enzyme |
| CDDP 04 | PTPN11 | Known interactions | protein tyrosine phosphatase non-receptor type 11 | Cytoplasm | phosphatase |
| CDDP 04 | ALDH1A1 | Known interactions | aldehyde dehydrogenase 1 family member A1 | Cytoplasm | enzyme |
| CDDP 04 | HSD17B10 | Known interactions | hydroxysteroid 17-beta dehydrogenase 10 | Cytoplasm | enzyme |
| CDDP 05 | TDP1 | Known interactions | tyrosyl-DNA phosphodiesterase 1 | Nucleus | enzyme |
| CDDP 05 | PTPN11 | Known interactions | protein tyrosine phosphatase non-receptor type 11 | Cytoplasm | phosphatase |
| CDDP 05 | PTPN6 | Known interactions | protein tyrosine phosphatase non-receptor type 6 | Cytoplasm | phosphatase |
| CDDP 05 | CES1 | Known interactions | carboxylesterase 1 | Cytoplasm | enzyme |
| CDDP 05 | CES2 | Known interactions | carboxylesterase 2 | Cytoplasm | enzyme |
| CDDP 05 | ACHE | Known interactions | acetylcholinesterase (Cartwright blood group) | Plasma Membrane | enzyme |
| CDDP 05 | MAPK1 | Known interactions | mitogen-activated protein kinase 1 | Cytoplasm | kinase |
| CDDP 05 | MAPT | Known interactions | microtubule associated protein tau | Plasma Membrane | other |
| CDDP 05 | KDM4E | Known interactions | lysine demethylase 4E | Nucleus | enzyme |
| CDDP 06 | CA7 | Known interactions | carbonic anhydrase 7 | Cytoplasm | enzyme |
| CDDP 06 | CA12 | Known interactions | carbonic anhydrase 12 | Plasma Membrane | enzyme |
| CDDP 06 | CA4 | Known interactions | carbonic anhydrase 4 | Plasma Membrane | enzyme |
| CDDP 06 | CA2 | Known interactions | carbonic anhydrase 2 | Cytoplasm | enzyme |
| CDDP 11 | KDM4E | Known interactions | lysine demethylase 4E | Nucleus | enzyme |
| CDDP 11 | KDM4A | Known interactions | lysine demethylase 4A | Nucleus | transcription regulator |
| CDDP 12 | AKR1B1 | Known interactions | aldo-keto reductase family 1 member B | Cytoplasm | enzyme |
| CDDP 12 | TTR | Known interactions | transthyretin | Extracellular Space | transporter |
| CDDP 12 | APEX1 | Known interactions | apurinic/apyrimidinic endodeoxyribonuclease 1 | Nucleus | enzyme |
| CDDP 12 | TDP1 | Known interactions | tyrosyl-DNA phosphodiesterase 1 | Nucleus | enzyme |
| CDDP 12 | PKM | Known interactions | pyruvate kinase M1/2 | Cytoplasm | kinase |
| CDDP 12 | KDM4E | Known interactions | lysine demethylase 4E | Nucleus | enzyme |
| CDDP 12 | RECQL | Known interactions | RecQ like helicase | Nucleus | enzyme |
| CDDP 12 | POLB | Known interactions | DNA polymerase beta | Nucleus | enzyme |
| CDDP 12 | GAA | Known interactions | glucosidase alpha, acid | Cytoplasm | enzyme |
| CDDP 12 | MMP1 | Known interactions | matrix metallopeptidase 1 | Extracellular Space | peptidase |
| CDDP 12 | MCL1 | Known interactions | MCL1 apoptosis regulator, BCL2 family member | Cytoplasm | transporter |
| CDDP 12 | FYN | Known interactions | FYN proto-oncogene, Src family tyrosine kinase | Plasma Membrane | kinase |
| CDDP 13 | CA7 | Known interactions | carbonic anhydrase 7 | Cytoplasm | enzyme |
| CDDP 13 | CA12 | Known interactions | carbonic anhydrase 12 | Plasma Membrane | enzyme |
| CDDP 13 | CA4 | Known interactions | carbonic anhydrase 4 | Plasma Membrane | enzyme |
| CDDP 13 | CA2 | Known interactions | carbonic anhydrase 2 | Cytoplasm | enzyme |
| CDDP 22 | TDP1 | Known interactions | tyrosyl-DNA phosphodiesterase 1 | Nucleus | enzyme |
| CDDP 22 | KDM4E | Known interactions | lysine demethylase 4E | Nucleus | enzyme |
| CDDP 22 | CA2 | Known interactions | carbonic anhydrase 2 | Cytoplasm | enzyme |
| CDDP 22 | CA7 | Known interactions | carbonic anhydrase 7 | Cytoplasm | enzyme |
| CDDP 22 | CA1 | Known interactions | carbonic anhydrase 1 | Cytoplasm | enzyme |
| CDDP 22 | CA3 | Known interactions | carbonic anhydrase 3 | Cytoplasm | enzyme |
| CDDP 22 | CA6 | Known interactions | carbonic anhydrase 6 | Extracellular Space | enzyme |
| CDDP 22 | CA12 | Known interactions | carbonic anhydrase 12 | Plasma Membrane | enzyme |
| CDDP 22 | NFKB1 | Known interactions | nuclear factor kappa B subunit 1 | Nucleus | transcription regulator |
| CDDP 22 | CA14 | Known interactions | carbonic anhydrase 14 | Plasma Membrane | enzyme |
| CDDP 22 | CA9 | Known interactions | carbonic anhydrase 9 | Nucleus | enzyme |
| CDDP 22 | CA4 | Known interactions | carbonic anhydrase 4 | Plasma Membrane | enzyme |
| CDDP 22 | CA5B | Known interactions | carbonic anhydrase 5B | Cytoplasm | enzyme |
| CDDP 22 | HSD17B10 | Known interactions | hydroxysteroid 17-beta dehydrogenase 10 | Cytoplasm | enzyme |
| CDDP 22 | CA5A | Known interactions | carbonic anhydrase 5A | Cytoplasm | enzyme |
| CDDP 22 | MMP9 | Known interactions | matrix metallopeptidase 9 | Extracellular Space | peptidase |
| CDDP 22 | MMP1 | Known interactions | matrix metallopeptidase 1 | Extracellular Space | peptidase |
| CDDP 22 | MMP2 | Known interactions | matrix metallopeptidase 2 | Extracellular Space | peptidase |
| CDDP 22 | PTPN1 | Known interactions | protein tyrosine phosphatase non-receptor type 1 | Cytoplasm | phosphatase |
| CDDP 22 | EGFR | Known interactions | epidermal growth factor receptor | Plasma Membrane | kinase |
| CDDP 22 | AKR1B1 | Known interactions | aldo-keto reductase family 1 member B | Cytoplasm | enzyme |
| CDDP 22 | XDH | Known interactions | xanthine dehydrogenase | Cytoplasm | enzyme |
| CDDP 22 | APEX1 | Known interactions | apurinic/apyrimidinic endodeoxyribonuclease 1 | Nucleus | enzyme |
| CDDP 35 | CES1 | Known interactions | carboxylesterase 1 | Cytoplasm | enzyme |
| CDDP 35 | CES2 | Known interactions | carboxylesterase 2 | Cytoplasm | enzyme |
| CDDP 35 | PTPN1 | Known interactions | protein tyrosine phosphatase non-receptor type 1 | Cytoplasm | phosphatase |
| CDDP 35 | CDC25A | Known interactions | cell division cycle 25A | Nucleus | phosphatase |
| CDDP 35 | CDC25B | Known interactions | cell division cycle 25B | Nucleus | phosphatase |
| CDDP 36 | CA2 | Known interactions | carbonic anhydrase 2 | Cytoplasm | enzyme |
| CDDP 36 | CA7 | Known interactions | carbonic anhydrase 7 | Cytoplasm | enzyme |
| CDDP 36 | CA1 | Known interactions | carbonic anhydrase 1 | Cytoplasm | enzyme |
| CDDP 36 | CA6 | Known interactions | carbonic anhydrase 6 | Extracellular Space | enzyme |
| CDDP 36 | CA12 | Known interactions | carbonic anhydrase 12 | Plasma Membrane | enzyme |
| CDDP 36 | CA14 | Known interactions | carbonic anhydrase 14 | Plasma Membrane | enzyme |
| CDDP 36 | CA9 | Known interactions | carbonic anhydrase 9 | Nucleus | enzyme |
| CDDP 36 | CA4 | Known interactions | carbonic anhydrase 4 | Plasma Membrane | enzyme |
| CDDP 36 | KDM4E | Known interactions | lysine demethylase 4E | Nucleus | enzyme |
| CDDP 36 | MMP9 | Known interactions | matrix metallopeptidase 9 | Extracellular Space | peptidase |
| CDDP 36 | MMP1 | Known interactions | matrix metallopeptidase 1 | Extracellular Space | peptidase |
| CDDP 36 | MMP2 | Known interactions | matrix metallopeptidase 2 | Extracellular Space | peptidase |
| CDDP 36 | PSMB5 | Known interactions | proteasome subunit beta 5 | Cytoplasm | peptidase |
| CDDP 36 | EGLN1 | Known interactions | egl-9 family hypoxia inducible factor 1 | Cytoplasm | enzyme |
| CDDP 37 | THPO | Known interactions | thrombopoietin | Extracellular Space | cytokine |
| CDDP 37 | CA2 | Known interactions | carbonic anhydrase 2 | Cytoplasm | enzyme |
| CDDP 37 | CA12 | Known interactions | carbonic anhydrase 12 | Plasma Membrane | enzyme |
| CDDP 37 | NFKB1 | Known interactions | nuclear factor kappa B subunit 1 | Nucleus | transcription regulator |
| CDDP 37 | CA5B | Known interactions | carbonic anhydrase 5B | Cytoplasm | enzyme |
| CDDP 37 | HSD17B10 | Known interactions | hydroxysteroid 17-beta dehydrogenase 10 | Cytoplasm | enzyme |
| CDDP 37 | MTOR | Known interactions | mechanistic target of rapamycin kinase | Nucleus | kinase |
| CDDP 37 | CYP3A4 | Known interactions | cytochrome P450 family 3 subfamily A member 4 | Cytoplasm | enzyme |
| CDDP 37 | FYN | Known interactions | FYN proto-oncogene, Src family tyrosine kinase | Plasma Membrane | kinase |
| CDDP 37 | CA7 | Known interactions | carbonic anhydrase 7 | Cytoplasm | enzyme |
| CDDP 37 | CA1 | Known interactions | carbonic anhydrase 1 | Cytoplasm | enzyme |
| CDDP 37 | CA3 | Known interactions | carbonic anhydrase 3 | Cytoplasm | enzyme |
| CDDP 37 | CA6 | Known interactions | carbonic anhydrase 6 | Extracellular Space | enzyme |
| CDDP 37 | CA14 | Known interactions | carbonic anhydrase 14 | Plasma Membrane | enzyme |
| CDDP 37 | CA9 | Known interactions | carbonic anhydrase 9 | Nucleus | enzyme |
| CDDP 37 | CA4 | Known interactions | carbonic anhydrase 4 | Plasma Membrane | enzyme |
| CDDP 37 | CA5A | Known interactions | carbonic anhydrase 5A | Cytoplasm | enzyme |
| CDDP 38 | TPMT | Known interactions | thiopurine S-methyltransferase | Cytoplasm | enzyme |
| CDDP 39 | TPMT | Known interactions | thiopurine S-methyltransferase | Cytoplasm | enzyme |
| CDDP 40 | TPMT | Known interactions | thiopurine S-methyltransferase | Cytoplasm | enzyme |
| CDDP 36 | FUT7 | Known interactions | fucosyltransferase 7 | Cytoplasm | enzyme |
| CDDP 02 | KMT2A | Known interactions | lysine methyltransferase 2A | Nucleus | transcription regulator |
| CDDP 02 | SENP8 | Known interactions | SUMO peptidase family member, NEDD8 specific | Cytoplasm | peptidase |
| CDDP 02 | SENP7 | Known interactions | SUMO specific peptidase 7 | Nucleus | peptidase |
| CDDP 02 | SENP6 | Known interactions | SUMO specific peptidase 6 | Cytoplasm | peptidase |
| CDDP 02 | APAF1 | Known interactions | apoptotic peptidase activating factor 1 | Cytoplasm | other |
| CDDP 02 | THRB | Known interactions | thyroid hormone receptor beta | Nucleus | ligand-dependent nuclear receptor |
| CDDP 02 | ATM | Known interactions | ATM serine/threonine kinase | Nucleus | kinase |
| CDDP 02 | PKM | Known interactions | pyruvate kinase M1/2 | Cytoplasm | kinase |
| CDDP 02 | GPR55 | Known interactions | G protein-coupled receptor 55 | Plasma Membrane | G-protein coupled receptor |
| CDDP 02 | NOD1 | Known interactions | nucleotide binding oligomerization domain containing 1 | Cytoplasm | other |
| CDDP 02 | USP2 | Known interactions | ubiquitin specific peptidase 2 | Cytoplasm | peptidase |
| CDDP 02 | PLEC | Known interactions | plectin | Cytoplasm | other |
| CDDP 02 | GALK1 | Known interactions | galactokinase 1 | Cytoplasm | kinase |
| CDDP 02 | SMAD3 | Known interactions | SMAD family member 3 | Nucleus | transcription regulator |
| CDDP 02 | NOD2 | Known interactions | nucleotide binding oligomerization domain containing 2 | Cytoplasm | other |
| CDDP 02 | GPR35 | Known interactions | G protein-coupled receptor 35 | Plasma Membrane | G-protein coupled receptor |
| CDDP 02 | EIF4H | Known interactions | eukaryotic translation initiation factor 4H | Cytoplasm | translation regulator |
| CDDP 02 | NPC1 | Known interactions | NPC intracellular cholesterol transporter 1 | Cytoplasm | transporter |
| CDDP 02 | PABPC1 | Known interactions | poly(A) binding protein cytoplasmic 1 | Cytoplasm | translation regulator |
| CDDP 02 | RAB9A | Known interactions | RAB9A, member RAS oncogene family | Cytoplasm | enzyme |
| CDDP 02 | HKDC1 | Known interactions | hexokinase domain containing 1 | Cytoplasm | kinase |
| CDDP 02 | NLRP3 | Known interactions | NLR family pyrin domain containing 3 | Cytoplasm | other |
| CDDP 02 | ATG4B | Known interactions | autophagy related 4B cysteine peptidase | Cytoplasm | peptidase |
| CDDP 02 | CTDSP1 | Known interactions | CTD small phosphatase 1 | Nucleus | phosphatase |
| CDDP 02 | TSHR | Known interactions | thyroid stimulating hormone receptor | Plasma Membrane | G-protein coupled receptor |
| CDDP 02 | DNMT1 | Known interactions | DNA methyltransferase 1 | Nucleus | enzyme |
| CDDP 02 | CASP3 | Known interactions | caspase 3 | Cytoplasm | peptidase |
| CDDP 02 | GLA | Known interactions | galactosidase alpha | Cytoplasm | enzyme |
| CDDP 02 | GAA | Known interactions | glucosidase alpha, acid | Cytoplasm | enzyme |
| CDDP 02 | GSK3B | Known interactions | glycogen synthase kinase 3 beta | Nucleus | kinase |
| CDDP 02 | DUSP3 | Known interactions | dual specificity phosphatase 3 | Cytoplasm | phosphatase |
| CDDP 02 | F12 | Known interactions | coagulation factor XII | Extracellular Space | peptidase |
| CDDP 02 | GSK3A | Known interactions | glycogen synthase kinase 3 alpha | Nucleus | kinase |
| CDDP 02 | AHR | Known interactions | aryl hydrocarbon receptor | Nucleus | ligand-dependent nuclear receptor |
| CDDP 02 | PLA2G7 | Known interactions | phospholipase A2 group VII | Extracellular Space | enzyme |
| CDDP 02 | CDK5 | Known interactions | cyclin dependent kinase 5 | Nucleus | kinase |
| CDDP 02 | MAPK1 | Known interactions | mitogen-activated protein kinase 1 | Cytoplasm | kinase |
| CDDP 02 | NTSR1 | Known interactions | neurotensin receptor 1 | Plasma Membrane | G-protein coupled receptor |
| CDDP 02 | MCL1 | Known interactions | MCL1 apoptosis regulator, BCL2 family member | Cytoplasm | transporter |
| CDDP 02 | NR2E3 | Known interactions | nuclear receptor subfamily 2 group E member 3 | Nucleus | ligand-dependent nuclear receptor |
| CDDP 02 | NPSR1 | Known interactions | neuropeptide S receptor 1 | Plasma Membrane | G-protein coupled receptor |
| CDDP 02 | MAP4K2 | Known interactions | mitogen-activated protein kinase kinase kinase kinase 2 | Cytoplasm | kinase |
| CDDP 02 | HTT | Known interactions | huntingtin | Cytoplasm | transcription regulator |
| CDDP 02 | ALPI | Known interactions | alkaline phosphatase, intestinal | Plasma Membrane | phosphatase |
| CDDP 02 | TLR9 | Known interactions | toll like receptor 9 | Plasma Membrane | transmembrane receptor |
| CDDP 04 | ALOX15 | Known interactions | arachidonate 15-lipoxygenase | Cytoplasm | enzyme |
| CDDP 04 | CYP3A4 | Known interactions | cytochrome P450 family 3 subfamily A member 4 | Cytoplasm | enzyme |
| CDDP 04 | SENP8 | Known interactions | SUMO peptidase family member, NEDD8 specific | Cytoplasm | peptidase |
| CDDP 04 | SENP6 | Known interactions | SUMO specific peptidase 6 | Cytoplasm | peptidase |
| CDDP 05 | MITF | Known interactions | melanocyte inducing transcription factor | Nucleus | transcription regulator |
| CDDP 05 | GLA | Known interactions | galactosidase alpha | Cytoplasm | enzyme |
| CDDP 11 | TDP1 | Known interactions | tyrosyl-DNA phosphodiesterase 1 | Nucleus | enzyme |
| CDDP 11 | BLM | Known interactions | BLM RecQ like helicase | Nucleus | enzyme |
| CDDP 11 | PTPN22 | Known interactions | protein tyrosine phosphatase non-receptor type 22 | Cytoplasm | phosphatase |
| CDDP 11 | NFKB1 | Known interactions | nuclear factor kappa B subunit 1 | Nucleus | transcription regulator |
| CDDP 11 | CASP6 | Known interactions | caspase 6 | Cytoplasm | peptidase |
| CDDP 11 | ERAP1 | Known interactions | endoplasmic reticulum aminopeptidase 1 | Extracellular Space | peptidase |
| CDDP 20 | NFKB1 | Known interactions | nuclear factor kappa B subunit 1 | Nucleus | transcription regulator |
| CDDP 22 | HIF1A | Known interactions | hypoxia inducible factor 1 subunit alpha | Nucleus | transcription regulator |
| CDDP 22 | BLM | Known interactions | BLM RecQ like helicase | Nucleus | enzyme |
| CDDP 22 | SMAD3 | Known interactions | SMAD family member 3 | Nucleus | transcription regulator |
| CDDP 22 | GPR35 | Known interactions | G protein-coupled receptor 35 | Plasma Membrane | G-protein coupled receptor |
| CDDP 22 | ATG4B | Known interactions | autophagy related 4B cysteine peptidase | Cytoplasm | peptidase |
| CDDP 22 | PTPN22 | Known interactions | protein tyrosine phosphatase non-receptor type 22 | Cytoplasm | phosphatase |
| CDDP 22 | ALOX15 | Known interactions | arachidonate 15-lipoxygenase | Cytoplasm | enzyme |
| CDDP 22 | ERAP1 | Known interactions | endoplasmic reticulum aminopeptidase 1 | Extracellular Space | peptidase |
| CDDP 22 | SNCA | Known interactions | synuclein alpha | Cytoplasm | enzyme |
| CDDP 36 | TDP1 | Known interactions | tyrosyl-DNA phosphodiesterase 1 | Nucleus | enzyme |
| CDDP 36 | NFKB1 | Known interactions | nuclear factor kappa B subunit 1 | Nucleus | transcription regulator |
| CDDP 36 | HIF1A | Known interactions | hypoxia inducible factor 1 subunit alpha | Nucleus | transcription regulator |
| CDDP 36 | BLM | Known interactions | BLM RecQ like helicase | Nucleus | enzyme |
| CDDP 36 | GPR35 | Known interactions | G protein-coupled receptor 35 | Plasma Membrane | G-protein coupled receptor |
| CDDP 36 | CASP6 | Known interactions | caspase 6 | Cytoplasm | peptidase |
| CDDP 36 | ERAP1 | Known interactions | endoplasmic reticulum aminopeptidase 1 | Extracellular Space | peptidase |
| CDDP 37 | GLA | Known interactions | galactosidase alpha | Cytoplasm | enzyme |
| CDDP 38 | HPGD | Known interactions | 15-hydroxyprostaglandin dehydrogenase | Cytoplasm | enzyme |
| CDDP 38 | NPSR1 | Known interactions | neuropeptide S receptor 1 | Plasma Membrane | G-protein coupled receptor |
| CDDP 38 | TDP1 | Known interactions | tyrosyl-DNA phosphodiesterase 1 | Nucleus | enzyme |
| CDDP 38 | BLM | Known interactions | BLM RecQ like helicase | Nucleus | enzyme |
| CDDP 38 | NFKB1 | Known interactions | nuclear factor kappa B subunit 1 | Nucleus | transcription regulator |
| CDDP 38 | ERAP1 | Known interactions | endoplasmic reticulum aminopeptidase 1 | Extracellular Space | peptidase |
| CDDP 40 | CA2 | Known interactions | carbonic anhydrase 2 | Cytoplasm | enzyme |
| CDDP 40 | CA7 | Known interactions | carbonic anhydrase 7 | Cytoplasm | enzyme |
| CDDP 40 | CA1 | Known interactions | carbonic anhydrase 1 | Cytoplasm | enzyme |
| CDDP 40 | CA3 | Known interactions | carbonic anhydrase 3 | Cytoplasm | enzyme |
| CDDP 40 | CA6 | Known interactions | carbonic anhydrase 6 | Extracellular Space | enzyme |
| CDDP 40 | CA12 | Known interactions | carbonic anhydrase 12 | Plasma Membrane | enzyme |
| CDDP 40 | CA14 | Known interactions | carbonic anhydrase 14 | Plasma Membrane | enzyme |
| CDDP 40 | CA9 | Known interactions | carbonic anhydrase 9 | Nucleus | enzyme |
| CDDP 40 | CA4 | Known interactions | carbonic anhydrase 4 | Plasma Membrane | enzyme |
| CDDP 40 | CA5A | Known interactions | carbonic anhydrase 5A | Cytoplasm | enzyme |
| CDDP 40 | HPGD | Known interactions | 15-hydroxyprostaglandin dehydrogenase | Cytoplasm | enzyme |
| CDDP 40 | ALOX15 | Known interactions | arachidonate 15-lipoxygenase | Cytoplasm | enzyme |
| CDDP 40 | CYP1A2 | Known interactions | cytochrome P450 family 1 subfamily A member 2 | Cytoplasm | enzyme |
| CDDP 40 | CYP2C9 | Known interactions | cytochrome P450 family 2 subfamily C member 9 | Cytoplasm | enzyme |
| CDDP 40 | NPSR1 | Known interactions | neuropeptide S receptor 1 | Plasma Membrane | G-protein coupled receptor |
| CDDP 40 | TDP1 | Known interactions | tyrosyl-DNA phosphodiesterase 1 | Nucleus | enzyme |
| CDDP 40 | BLM | Known interactions | BLM RecQ like helicase | Nucleus | enzyme |
| CDDP 40 | NFKB1 | Known interactions | nuclear factor kappa B subunit 1 | Nucleus | transcription regulator |
| CDDP 40 | CA5B | Known interactions | carbonic anhydrase 5B | Cytoplasm | enzyme |
| CDDP 01 | APEX1 | Predicted interactions | apurinic/apyrimidinic endodeoxyribonuclease 1 | Nucleus | enzyme |
| CDDP 01 | BLM | Predicted interactions | BLM RecQ like helicase | Nucleus | enzyme |
| CDDP 01 | CA3 | Predicted interactions | carbonic anhydrase 3 | Cytoplasm | enzyme |
| CDDP 01 | CA4 | Predicted interactions | carbonic anhydrase 4 | Plasma Membrane | enzyme |
| CDDP 01 | CA5A | Predicted interactions | carbonic anhydrase 5A | Cytoplasm | enzyme |
| CDDP 01 | CA5B | Predicted interactions | carbonic anhydrase 5B | Cytoplasm | enzyme |
| CDDP 01 | CA6 | Predicted interactions | carbonic anhydrase 6 | Extracellular Space | enzyme |
| CDDP 01 | CTBP2 | Predicted interactions | C-terminal binding protein 2 | Nucleus | transcription regulator |
| CDDP 01 | EGFR | Predicted interactions | epidermal growth factor receptor | Plasma Membrane | kinase |
| CDDP 01 | FOLH1 | Predicted interactions | folate hydrolase 1 | Plasma Membrane | peptidase |
| CDDP 01 | FYN | Predicted interactions | FYN proto-oncogene, Src family tyrosine kinase | Plasma Membrane | kinase |
| CDDP 01 | HSD17B10 | Predicted interactions | hydroxysteroid 17-beta dehydrogenase 10 | Cytoplasm | enzyme |
| CDDP 01 | HIF1A | Predicted interactions | hypoxia inducible factor 1 subunit alpha | Nucleus | transcription regulator |
| CDDP 01 | LCK | Predicted interactions | LCK proto-oncogene, Src family tyrosine kinase | Cytoplasm | kinase |
| CDDP 01 | ALOX15 | Predicted interactions | arachidonate 15-lipoxygenase | Cytoplasm | enzyme |
| CDDP 01 | NFKB1 | Predicted interactions | nuclear factor kappa B subunit 1 | Nucleus | transcription regulator |
| CDDP 01 | NR1H4 | Predicted interactions | nuclear receptor subfamily 1 group H member 4 | Nucleus | ligand-dependent nuclear receptor |
| CDDP 01 | NR1I2 | Predicted interactions | nuclear receptor subfamily 1 group I member 2 | Nucleus | ligand-dependent nuclear receptor |
| CDDP 01 | ALPG | Predicted interactions | alkaline phosphatase, germ cell | Plasma Membrane | phosphatase |
| CDDP 01 | RECQL | Predicted interactions | RecQ like helicase | Nucleus | enzyme |
| CDDP 01 | THPO | Predicted interactions | thrombopoietin | Extracellular Space | cytokine |
| CDDP 01 | TDP1 | Predicted interactions | tyrosyl-DNA phosphodiesterase 1 | Nucleus | enzyme |
| CDDP 02 | MITF | Predicted interactions | melanocyte inducing transcription factor | Nucleus | transcription regulator |
| CDDP 02 | PTPRC | Predicted interactions | protein tyrosine phosphatase receptor type C | Plasma Membrane | phosphatase |
| CDDP 02 | HSP90AA1 | Predicted interactions | heat shock protein 90 alpha family class A member 1 | Cytoplasm | enzyme |
| CDDP 02 | DYRK4 | Predicted interactions | dual specificity tyrosine phosphorylation regulated kinase 4 | Nucleus | kinase |
| CDDP 02 | HIPK4 | Predicted interactions | homeodomain interacting protein kinase 4 | Cytoplasm | kinase |
| CDDP 02 | L3MBTL1 | Predicted interactions | L3MBTL histone methyl-lysine binding protein 1 | Nucleus | other |
| CDDP 02 | KDM4E | Predicted interactions | lysine demethylase 4E | Nucleus | enzyme |
| CDDP 02 | NR4A1 | Predicted interactions | nuclear receptor subfamily 4 group A member 1 | Nucleus | ligand-dependent nuclear receptor |
| CDDP 02 | SMN1 | Predicted interactions | survival Of Motor Neuron 1, Telomeric | Nucleus | other |
| CDDP 02 | SMN2 | Predicted interactions | survival Of Motor Neuron 2, Centromeric | Nucleus | other |
| CDDP 02 | LMNA | Predicted interactions | lamin A/C | Nucleus | other |
| CDDP 02 | RECQL | Predicted interactions | RecQ like helicase | Nucleus | enzyme |
| CDDP 02 | HPGD | Predicted interactions | 15-hydroxyprostaglandin dehydrogenase | Cytoplasm | enzyme |
| CDDP 02 | THPO | Predicted interactions | thrombopoietin | Extracellular Space | cytokine |
| CDDP 02 | PMP22 | Predicted interactions | peripheral myelin protein 22 | Plasma Membrane | other |
| CDDP 02 | XBP1 | Predicted interactions | X-box binding protein 1 | Nucleus | transcription regulator |
| CDDP 02 | TNF | Predicted interactions | tumor necrosis factor | Extracellular Space | cytokine |
| CDDP 02 | FYN | Predicted interactions | FYN proto-oncogene, Src family tyrosine kinase | Plasma Membrane | kinase |
| CDDP 02 | CSF1R | Predicted interactions | colony stimulating factor 1 receptor | Plasma Membrane | kinase |
| CDDP 02 | FLT1 | Predicted interactions | fms related tyrosine kinase 1 | Plasma Membrane | kinase |
| CDDP 02 | AR | Predicted interactions | androgen receptor | Nucleus | ligand-dependent nuclear receptor |
| CDDP 02 | AKR1B1 | Predicted interactions | aldo-keto reductase family 1 member B | Cytoplasm | enzyme |
| CDDP 02 | KIT | Predicted interactions | KIT proto-oncogene, receptor tyrosine kinase | Plasma Membrane | transmembrane receptor |
| CDDP 02 | MAOA | Predicted interactions | monoamine oxidase A | Cytoplasm | enzyme |
| CDDP 02 | FLT4 | Predicted interactions | fms related tyrosine kinase 4 | Plasma Membrane | transmembrane receptor |
| CDDP 02 | FLT3 | Predicted interactions | fms related tyrosine kinase 3 | Plasma Membrane | kinase |
| CDDP 02 | INSR | Predicted interactions | insulin receptor | Plasma Membrane | kinase |
| CDDP 02 | PDGFRA | Predicted interactions | platelet derived growth factor receptor alpha | Plasma Membrane | kinase |
| CDDP 02 | MAOB | Predicted interactions | monoamine oxidase B | Cytoplasm | enzyme |
| CDDP 02 | RET | Predicted interactions | ret proto-oncogene | Plasma Membrane | kinase |
| CDDP 02 | FGB | Predicted interactions | fibrinogen beta chain | Extracellular Space | other |
| CDDP 02 | CA2 | Predicted interactions | carbonic anhydrase 2 | Cytoplasm | enzyme |
| CDDP 02 | ESR1 | Predicted interactions | estrogen receptor 1 | Nucleus | ligand-dependent nuclear receptor |
| CDDP 02 | PGR | Predicted interactions | progesterone receptor | Nucleus | ligand-dependent nuclear receptor |
| CDDP 02 | PIM1 | Predicted interactions | Pim-1 proto-oncogene, serine/threonine kinase | Cytoplasm | kinase |
| CDDP 02 | TDP2 | Predicted interactions | tyrosyl-DNA phosphodiesterase 2 | Cytoplasm | transcription regulator |
| CDDP 02 | PTGS1 | Predicted interactions | prostaglandin-endoperoxide synthase 1 | Cytoplasm | enzyme |
| CDDP 02 | CYP1A1 | Predicted interactions | cytochrome P450 family 1 subfamily A member 1 | Cytoplasm | enzyme |
| CDDP 02 | BLK | Predicted interactions | BLK proto-oncogene, Src family tyrosine kinase | Cytoplasm | kinase |
| CDDP 02 | DYRK1A | Predicted interactions | dual specificity tyrosine phosphorylation regulated kinase 1A | Nucleus | kinase |
| CDDP 02 | PTGS2 | Predicted interactions | prostaglandin-endoperoxide synthase 2 | Cytoplasm | enzyme |
| CDDP 02 | CA7 | Predicted interactions | carbonic anhydrase 7 | Cytoplasm | enzyme |
| CDDP 02 | FTO | Predicted interactions | FTO alpha-ketoglutarate dependent dioxygenase | Nucleus | enzyme |
| CDDP 02 | RPS6KA3 | Predicted interactions | ribosomal protein S6 kinase A3 | Cytoplasm | kinase |
| CDDP 02 | PHKG2 | Predicted interactions | phosphorylase kinase catalytic subunit gamma 2 | Cytoplasm | kinase |
| CDDP 02 | RAD52 | Predicted interactions | RAD52 homolog, DNA repair protein | Nucleus | other |
| CDDP 02 | PAX8 | Predicted interactions | paired box 8 | Nucleus | transcription regulator |
| CDDP 02 | CDC25C | Predicted interactions | cell division cycle 25C | Nucleus | phosphatase |
| CDDP 02 | KDM5A | Predicted interactions | lysine demethylase 5A | Nucleus | transcription regulator |
| CDDP 02 | CSNK1G1 | Predicted interactions | casein kinase 1 gamma 1 | Cytoplasm | kinase |
| CDDP 02 | ESR2 | Predicted interactions | estrogen receptor 2 | Nucleus | ligand-dependent nuclear receptor |
| CDDP 02 | KLK7 | Predicted interactions | kallikrein related peptidase 7 | Extracellular Space | peptidase |
| CDDP 02 | DAPK3 | Predicted interactions | death associated protein kinase 3 | Cytoplasm | kinase |
| CDDP 02 | ELANE | Predicted interactions | elastase, neutrophil expressed | Extracellular Space | peptidase |
| CDDP 02 | CAMK1 | Predicted interactions | calcium/calmodulin dependent protein kinase I | Cytoplasm | kinase |
| CDDP 02 | ADORA2A | Predicted interactions | adenosine A2a receptor | Plasma Membrane | G-protein coupled receptor |
| CDDP 02 | PDPK1 | Predicted interactions | 3-phosphoinositide dependent protein kinase 1 | Cytoplasm | kinase |
| CDDP 02 | CNR2 | Predicted interactions | cannabinoid receptor 2 | Plasma Membrane | G-protein coupled receptor |
| CDDP 02 | CSNK1G2 | Predicted interactions | casein kinase 1 gamma 2 | Cytoplasm | kinase |
| CDDP 02 | ADORA3 | Predicted interactions | adenosine A3 receptor | Plasma Membrane | G-protein coupled receptor |
| CDDP 02 | LCK | Predicted interactions | LCK proto-oncogene, Src family tyrosine kinase | Cytoplasm | kinase |
| CDDP 02 | PRKD3 | Predicted interactions | protein kinase D3 | Nucleus | kinase |
| CDDP 02 | CA1 | Predicted interactions | carbonic anhydrase 1 | Cytoplasm | enzyme |
| CDDP 02 | TGM2 | Predicted interactions | transglutaminase 2 | Cytoplasm | enzyme |
| CDDP 02 | FGFR3 | Predicted interactions | fibroblast growth factor receptor 3 | Plasma Membrane | kinase |
| CDDP 02 | MPI | Predicted interactions | mannose phosphate isomerase | Cytoplasm | enzyme |
| CDDP 02 | CSNK1A1 | Predicted interactions | casein kinase 1 alpha 1 | Cytoplasm | kinase |
| CDDP 02 | MAP4K4 | Predicted interactions | mitogen-activated protein kinase kinase kinase kinase 4 | Cytoplasm | kinase |
| CDDP 02 | CSNK1D | Predicted interactions | casein kinase 1 delta | Cytoplasm | kinase |
| CDDP 02 | CA3 | Predicted interactions | carbonic anhydrase 3 | Cytoplasm | enzyme |
| CDDP 02 | NFKBIA | Predicted interactions | NFKB inhibitor alpha | Cytoplasm | transcription regulator |
| CDDP 02 | CYP2D6 | Predicted interactions | cytochrome P450 family 2 subfamily D member 6 | Cytoplasm | enzyme |
| CDDP 02 | ALOX15 | Predicted interactions | arachidonate 15-lipoxygenase | Cytoplasm | enzyme |
| CDDP 02 | CAMK2D | Predicted interactions | calcium/calmodulin dependent protein kinase II delta | Cytoplasm | kinase |
| CDDP 02 | MAPK13 | Predicted interactions | mitogen-activated protein kinase 13 | Cytoplasm | kinase |
| CDDP 02 | PRKCG | Predicted interactions | protein kinase C gamma | Cytoplasm | kinase |
| CDDP 02 | PTPN13 | Predicted interactions | protein tyrosine phosphatase non-receptor type 13 | Cytoplasm | phosphatase |
| CDDP 02 | PRKCA | Predicted interactions | protein kinase C alpha | Cytoplasm | kinase |
| CDDP 02 | S1PR2 | Predicted interactions | sphingosine-1-phosphate receptor 2 | Plasma Membrane | G-protein coupled receptor |
| CDDP 02 | CDK2 | Predicted interactions | cyclin dependent kinase 2 | Nucleus | kinase |
| CDDP 02 | CA6 | Predicted interactions | carbonic anhydrase 6 | Extracellular Space | enzyme |
| CDDP 02 | PKN2 | Predicted interactions | protein kinase N2 | Cytoplasm | kinase |
| CDDP 02 | CDK1 | Predicted interactions | cyclin dependent kinase 1 | Nucleus | kinase |
| CDDP 02 | PARP1 | Predicted interactions | poly(ADP-ribose) polymerase 1 | Nucleus | enzyme |
| CDDP 02 | BAZ2A | Predicted interactions | bromodomain adjacent to zinc finger domain 2A | Nucleus | transcription regulator |
| CDDP 02 | NSD2 | Predicted interactions | nuclear receptor binding SET domain protein 2 | Nucleus | enzyme |
| CDDP 02 | S1PR4 | Predicted interactions | sphingosine-1-phosphate receptor 4 | Plasma Membrane | G-protein coupled receptor |
| CDDP 02 | ROCK1 | Predicted interactions | Rho associated coiled-coil containing protein kinase 1 | Cytoplasm | kinase |
| CDDP 02 | NFKB1 | Predicted interactions | nuclear factor kappa B subunit 1 | Nucleus | transcription regulator |
| CDDP 02 | SHBG | Predicted interactions | sex hormone binding globulin | Extracellular Space | other |
| CDDP 02 | CASP6 | Predicted interactions | caspase 6 | Cytoplasm | peptidase |
| CDDP 02 | CYP1A2 | Predicted interactions | cytochrome P450 family 1 subfamily A member 2 | Cytoplasm | enzyme |
| CDDP 02 | ABCC1 | Predicted interactions | ATP binding cassette subfamily C member 1 | Plasma Membrane | transporter |
| CDDP 02 | PTPN1 | Predicted interactions | protein tyrosine phosphatase non-receptor type 1 | Cytoplasm | phosphatase |
| CDDP 02 | CYP2C9 | Predicted interactions | cytochrome P450 family 2 subfamily C member 9 | Cytoplasm | enzyme |
| CDDP 02 | CYP3A4 | Predicted interactions | cytochrome P450 family 3 subfamily A member 4 | Cytoplasm | enzyme |
| CDDP 02 | IRAK1 | Predicted interactions | interleukin 1 receptor associated kinase 1 | Plasma Membrane | kinase |
| CDDP 02 | PTPRF | Predicted interactions | protein tyrosine phosphatase receptor type F | Plasma Membrane | phosphatase |
| CDDP 02 | ALDH1A1 | Predicted interactions | aldehyde dehydrogenase 1 family member A1 | Cytoplasm | enzyme |
| CDDP 02 | CYP2C19 | Predicted interactions | cytochrome P450 family 2 subfamily C member 19 | Cytoplasm | enzyme |
| CDDP 02 | PRKCZ | Predicted interactions | protein kinase C zeta | Cytoplasm | kinase |
| CDDP 02 | CSNK2A1 | Predicted interactions | casein kinase 2 alpha 1 | Nucleus | kinase |
| CDDP 02 | FGFR1 | Predicted interactions | fibroblast growth factor receptor 1 | Plasma Membrane | kinase |
| CDDP 02 | NQO1 | Predicted interactions | NAD(P)H quinone dehydrogenase 1 | Cytoplasm | enzyme |
| CDDP 02 | TMIGD3 | Predicted interactions | transmembrane and immunoglobulin domain containing 3 | Plasma Membrane | other |
| CDDP 02 | CYP2C8 | Predicted interactions | cytochrome P450 family 2 subfamily C member 8 | Cytoplasm | enzyme |
| CDDP 02 | CA4 | Predicted interactions | carbonic anhydrase 4 | Plasma Membrane | enzyme |
| CDDP 02 | CDC25A | Predicted interactions | cell division cycle 25A | Nucleus | phosphatase |
| CDDP 02 | IRAK4 | Predicted interactions | interleukin 1 receptor associated kinase 4 | Cytoplasm | kinase |
| CDDP 02 | PLK4 | Predicted interactions | polo like kinase 4 | Cytoplasm | kinase |
| CDDP 02 | CAMK2G | Predicted interactions | calcium/calmodulin dependent protein kinase II gamma | Cytoplasm | kinase |
| CDDP 02 | MARK2 | Predicted interactions | microtubule affinity regulating kinase 2 | Cytoplasm | kinase |
| CDDP 02 | ALOX12 | Predicted interactions | arachidonate 12-lipoxygenase, 12S type | Cytoplasm | enzyme |
| CDDP 02 | CA13 | Predicted interactions | carbonic anhydrase 13 | Cytoplasm | enzyme |
| CDDP 02 | LIMK1 | Predicted interactions | LIM domain kinase 1 | Cytoplasm | kinase |
| CDDP 02 | CA5B | Predicted interactions | carbonic anhydrase 5B | Cytoplasm | enzyme |
| CDDP 02 | STK17B | Predicted interactions | serine/threonine kinase 17b | Nucleus | kinase |
| CDDP 02 | FER | Predicted interactions | FER tyrosine kinase | Cytoplasm | kinase |
| CDDP 02 | NPY2R | Predicted interactions | neuropeptide Y receptor Y2 | Plasma Membrane | G-protein coupled receptor |
| CDDP 02 | PLCG1 | Predicted interactions | phospholipase C gamma 1 | Cytoplasm | enzyme |
| CDDP 02 | CAMK2B | Predicted interactions | calcium/calmodulin dependent protein kinase II beta | Cytoplasm | kinase |
| CDDP 02 | HSD17B10 | Predicted interactions | hydroxysteroid 17-beta dehydrogenase 10 | Cytoplasm | enzyme |
| CDDP 02 | SLK | Predicted interactions | STE20 like kinase | Nucleus | kinase |
| CDDP 02 | FRK | Predicted interactions | fyn related Src family tyrosine kinase | Nucleus | kinase |
| CDDP 02 | STAT3 | Predicted interactions | signal transducer and activator of transcription 3 | Nucleus | transcription regulator |
| CDDP 02 | HSD11B1 | Predicted interactions | hydroxysteroid 11-beta dehydrogenase 1 | Cytoplasm | enzyme |
| CDDP 02 | ALK | Predicted interactions | ALK receptor tyrosine kinase | Plasma Membrane | kinase |
| CDDP 02 | HIF1A | Predicted interactions | hypoxia inducible factor 1 subunit alpha | Nucleus | transcription regulator |
| CDDP 02 | PRKG1 | Predicted interactions | protein kinase cGMP-dependent 1 | Cytoplasm | kinase |
| CDDP 02 | HBB | Predicted interactions | hemoglobin subunit beta | Cytoplasm | transporter |
| CDDP 02 | CLK1 | Predicted interactions | CDC like kinase 1 | Nucleus | kinase |
| CDDP 02 | DYRK2 | Predicted interactions | dual specificity tyrosine phosphorylation regulated kinase 2 | Cytoplasm | kinase |
| CDDP 02 | PLA2G1B | Predicted interactions | phospholipase A2 group IB | Extracellular Space | enzyme |
| CDDP 02 | SIRT1 | Predicted interactions | sirtuin 1 | Nucleus | transcription regulator |
| CDDP 02 | CDC42BPA | Predicted interactions | CDC42 binding protein kinase alpha | Cytoplasm | kinase |
| CDDP 02 | SRPK1 | Predicted interactions | SRSF protein kinase 1 | Nucleus | kinase |
| CDDP 02 | DYRK3 | Predicted interactions | dual specificity tyrosine phosphorylation regulated kinase 3 | Nucleus | kinase |
| CDDP 02 | HIPK2 | Predicted interactions | homeodomain interacting protein kinase 2 | Nucleus | kinase |
| CDDP 02 | C1R | Predicted interactions | complement C1r | Extracellular Space | peptidase |
| CDDP 02 | STK17A | Predicted interactions | serine/threonine kinase 17a | Nucleus | kinase |
| CDDP 02 | PDF | Predicted interactions | peptide deformylase, mitochondrial | Cytoplasm | enzyme |
| CDDP 02 | MAPK12 | Predicted interactions | mitogen-activated protein kinase 12 | Cytoplasm | kinase |
| CDDP 02 | AKR1C3 | Predicted interactions | aldo-keto reductase family 1 member C3 | Cytoplasm | enzyme |
| CDDP 02 | STK3 | Predicted interactions | serine/threonine kinase 3 | Cytoplasm | kinase |
| CDDP 02 | CA5A | Predicted interactions | carbonic anhydrase 5A | Cytoplasm | enzyme |
| CDDP 02 | CASP1 | Predicted interactions | caspase 1 | Cytoplasm | peptidase |
| CDDP 02 | CHEK1 | Predicted interactions | checkpoint kinase 1 | Nucleus | kinase |
| CDDP 02 | AKT3 | Predicted interactions | AKT serine/threonine kinase 3 | Cytoplasm | kinase |
| CDDP 02 | MAP4K5 | Predicted interactions | mitogen-activated protein kinase kinase kinase kinase 5 | Cytoplasm | kinase |
| CDDP 02 | PRNP | Predicted interactions | prion protein | Plasma Membrane | other |
| CDDP 02 | CDC25B | Predicted interactions | cell division cycle 25B | Nucleus | phosphatase |
| CDDP 02 | CYP1B1 | Predicted interactions | cytochrome P450 family 1 subfamily B member 1 | Cytoplasm | enzyme |
| CDDP 02 | PLK3 | Predicted interactions | polo like kinase 3 | Nucleus | kinase |
| CDDP 02 | NTRK2 | Predicted interactions | neurotrophic receptor tyrosine kinase 2 | Plasma Membrane | kinase |
| CDDP 02 | AXL | Predicted interactions | AXL receptor tyrosine kinase | Plasma Membrane | kinase |
| CDDP 02 | CSNK1G3 | Predicted interactions | casein kinase 1 gamma 3 | Cytoplasm | kinase |
| CDDP 02 | MAP3K5 | Predicted interactions | mitogen-activated protein kinase kinase kinase 5 | Cytoplasm | kinase |
| CDDP 02 | PRKD2 | Predicted interactions | protein kinase D2 | Cytoplasm | kinase |
| CDDP 02 | PIM3 | Predicted interactions | Pim-3 proto-oncogene, serine/threonine kinase | Cytoplasm | kinase |
| CDDP 02 | PTK2B | Predicted interactions | protein tyrosine kinase 2 beta | Cytoplasm | kinase |
| CDDP 02 | WEE1 | Predicted interactions | WEE1 G2 checkpoint kinase | Nucleus | kinase |
| CDDP 02 | MINK1 | Predicted interactions | misshapen like kinase 1 | Cytoplasm | kinase |
| CDDP 02 | RELA | Predicted interactions | RELA proto-oncogene, NF-kB subunit | Nucleus | transcription regulator |
| CDDP 02 | MARK3 | Predicted interactions | microtubule affinity regulating kinase 3 | Cytoplasm | kinase |
| CDDP 02 | ABCG2 | Predicted interactions | ATP binding cassette subfamily G member 2 (Junior blood group) | Plasma Membrane | transporter |
| CDDP 02 | NTRK3 | Predicted interactions | neurotrophic receptor tyrosine kinase 3 | Plasma Membrane | kinase |
| CDDP 02 | BRSK1 | Predicted interactions | BR serine/threonine kinase 1 | Cytoplasm | kinase |
| CDDP 02 | SGK2 | Predicted interactions | serum/glucocorticoid regulated kinase 2 | Cytoplasm | kinase |
| CDDP 02 | APEX1 | Predicted interactions | apurinic/apyrimidinic endodeoxyribonuclease 1 | Nucleus | enzyme |
| CDDP 02 | NEK1 | Predicted interactions | NIMA related kinase 1 | Nucleus | kinase |
| CDDP 02 | NEK4 | Predicted interactions | NIMA related kinase 4 | Nucleus | kinase |
| CDDP 02 | STAT1 | Predicted interactions | signal transducer and activator of transcription 1 | Nucleus | transcription regulator |
| CDDP 02 | SLC22A12 | Predicted interactions | solute carrier family 22 member 12 | Plasma Membrane | transporter |
| CDDP 02 | GRK6 | Predicted interactions | G protein-coupled receptor kinase 6 | Plasma Membrane | kinase |
| CDDP 02 | TNKS2 | Predicted interactions | tankyrase 2 | Nucleus | enzyme |
| CDDP 02 | TNKS | Predicted interactions | tankyrase | Nucleus | enzyme |
| CDDP 02 | ERAP1 | Predicted interactions | endoplasmic reticulum aminopeptidase 1 | Extracellular Space | peptidase |
| CDDP 03 | APAF1 | Predicted interactions | apoptotic peptidase activating factor 1 | Cytoplasm | other |
| CDDP 03 | AKR1B1 | Predicted interactions | aldo-keto reductase family 1 member B | Cytoplasm | enzyme |
| CDDP 03 | MALT1 | Predicted interactions | MALT1 paracaspase | Cytoplasm | peptidase |
| CDDP 03 | CDC25A | Predicted interactions | cell division cycle 25A | Nucleus | phosphatase |
| CDDP 03 | CDC25B | Predicted interactions | cell division cycle 25B | Nucleus | phosphatase |
| CDDP 04 | ESR1 | Predicted interactions | estrogen receptor 1 | Nucleus | ligand-dependent nuclear receptor |
| CDDP 04 | ESR2 | Predicted interactions | estrogen receptor 2 | Nucleus | ligand-dependent nuclear receptor |
| CDDP 04 | ELANE | Predicted interactions | elastase, neutrophil expressed | Extracellular Space | peptidase |
| CDDP 04 | PTPN1 | Predicted interactions | protein tyrosine phosphatase non-receptor type 1 | Cytoplasm | phosphatase |
| CDDP 04 | MALT1 | Predicted interactions | MALT1 paracaspase | Cytoplasm | peptidase |
| CDDP 04 | CDC25A | Predicted interactions | cell division cycle 25A | Nucleus | phosphatase |
| CDDP 04 | STAT3 | Predicted interactions | signal transducer and activator of transcription 3 | Nucleus | transcription regulator |
| CDDP 04 | CDC25B | Predicted interactions | cell division cycle 25B | Nucleus | phosphatase |
| CDDP 05 | FGB | Predicted interactions | fibrinogen beta chain | Extracellular Space | other |
| CDDP 05 | BRCA1 | Predicted interactions | BRCA1 DNA repair associated | Nucleus | transcription regulator |
| CDDP 05 | TOP2A | Predicted interactions | DNA topoisomerase II alpha | Nucleus | enzyme |
| CDDP 05 | MAP3K5 | Predicted interactions | mitogen-activated protein kinase kinase kinase 5 | Cytoplasm | kinase |
| CDDP 05 | TDP2 | Predicted interactions | tyrosyl-DNA phosphodiesterase 2 | Cytoplasm | transcription regulator |
| CDDP 05 | ESR2 | Predicted interactions | estrogen receptor 2 | Nucleus | ligand-dependent nuclear receptor |
| CDDP 05 | NSD2 | Predicted interactions | nuclear receptor binding SET domain protein 2 | Nucleus | enzyme |
| CDDP 05 | NCEH1 | Predicted interactions | neutral cholesterol ester hydrolase 1 | Plasma Membrane | enzyme |
| CDDP 05 | GALK1 | Predicted interactions | galactokinase 1 | Cytoplasm | kinase |
| CDDP 05 | APAF1 | Predicted interactions | apoptotic peptidase activating factor 1 | Cytoplasm | other |
| CDDP 05 | APEX1 | Predicted interactions | apurinic/apyrimidinic endodeoxyribonuclease 1 | Nucleus | enzyme |
| CDDP 05 | HKDC1 | Predicted interactions | hexokinase domain containing 1 | Cytoplasm | kinase |
| CDDP 05 | NUAK1 | Predicted interactions | NUAK family kinase 1 | Nucleus | kinase |
| CDDP 05 | MAOA | Predicted interactions | monoamine oxidase A | Cytoplasm | enzyme |
| CDDP 05 | CDC25A | Predicted interactions | cell division cycle 25A | Nucleus | phosphatase |
| CDDP 05 | NLRP3 | Predicted interactions | NLR family pyrin domain containing 3 | Cytoplasm | other |
| CDDP 05 | ELANE | Predicted interactions | elastase, neutrophil expressed | Extracellular Space | peptidase |
| CDDP 05 | ESR1 | Predicted interactions | estrogen receptor 1 | Nucleus | ligand-dependent nuclear receptor |
| CDDP 05 | HBB | Predicted interactions | hemoglobin subunit beta | Cytoplasm | transporter |
| CDDP 05 | CDC25B | Predicted interactions | cell division cycle 25B | Nucleus | phosphatase |
| CDDP 05 | IDO1 | Predicted interactions | indoleamine 2,3-dioxygenase 1 | Cytoplasm | enzyme |
| CDDP 05 | STAT3 | Predicted interactions | signal transducer and activator of transcription 3 | Nucleus | transcription regulator |
| CDDP 05 | AR | Predicted interactions | androgen receptor | Nucleus | ligand-dependent nuclear receptor |
| CDDP 05 | MCL1 | Predicted interactions | MCL1 apoptosis regulator, BCL2 family member | Cytoplasm | transporter |
| CDDP 05 | ATG4B | Predicted interactions | autophagy related 4B cysteine peptidase | Cytoplasm | peptidase |
| CDDP 05 | FGFR1 | Predicted interactions | fibroblast growth factor receptor 1 | Plasma Membrane | kinase |
| CDDP 05 | CA13 | Predicted interactions | carbonic anhydrase 13 | Cytoplasm | enzyme |
| CDDP 05 | TLR9 | Predicted interactions | toll like receptor 9 | Plasma Membrane | transmembrane receptor |
| CDDP 05 | XBP1 | Predicted interactions | X-box binding protein 1 | Nucleus | transcription regulator |
| CDDP 05 | SENP7 | Predicted interactions | SUMO specific peptidase 7 | Nucleus | peptidase |
| CDDP 05 | PAX8 | Predicted interactions | paired box 8 | Nucleus | transcription regulator |
| CDDP 05 | ATM | Predicted interactions | ATM serine/threonine kinase | Nucleus | kinase |
| CDDP 05 | MAOB | Predicted interactions | monoamine oxidase B | Cytoplasm | enzyme |
| CDDP 05 | CREBBP | Predicted interactions | CREB binding protein | Nucleus | transcription regulator |
| CDDP 05 | GSK3B | Predicted interactions | glycogen synthase kinase 3 beta | Nucleus | kinase |
| CDDP 05 | SENP8 | Predicted interactions | SUMO peptidase family member, NEDD8 specific | Cytoplasm | peptidase |
| CDDP 05 | CYP1A2 | Predicted interactions | cytochrome P450 family 1 subfamily A member 2 | Cytoplasm | enzyme |
| CDDP 05 | CDK2 | Predicted interactions | cyclin dependent kinase 2 | Nucleus | kinase |
| CDDP 05 | USP2 | Predicted interactions | ubiquitin specific peptidase 2 | Cytoplasm | peptidase |
| CDDP 05 | ERN1 | Predicted interactions | endoplasmic reticulum to nucleus signaling 1 | Cytoplasm | kinase |
| CDDP 05 | PTGS1 | Predicted interactions | prostaglandin-endoperoxide synthase 1 | Cytoplasm | enzyme |
| CDDP 05 | DUSP3 | Predicted interactions | dual specificity phosphatase 3 | Cytoplasm | phosphatase |
| CDDP 05 | CASP1 | Predicted interactions | caspase 1 | Cytoplasm | peptidase |
| CDDP 05 | SIRT2 | Predicted interactions | sirtuin 2 | Nucleus | transcription regulator |
| CDDP 05 | SLC22A12 | Predicted interactions | solute carrier family 22 member 12 | Plasma Membrane | transporter |
| CDDP 05 | PKM | Predicted interactions | pyruvate kinase M1/2 | Cytoplasm | kinase |
| CDDP 05 | WEE1 | Predicted interactions | WEE1 G2 checkpoint kinase | Nucleus | kinase |
| CDDP 05 | SENP6 | Predicted interactions | SUMO specific peptidase 6 | Cytoplasm | peptidase |
| CDDP 05 | NPY2R | Predicted interactions | neuropeptide Y receptor Y2 | Plasma Membrane | G-protein coupled receptor |
| CDDP 05 | ABCG2 | Predicted interactions | ATP binding cassette subfamily G member 2 (Junior blood group) | Plasma Membrane | transporter |
| CDDP 05 | ALOX5 | Predicted interactions | arachidonate 5-lipoxygenase | Cytoplasm | enzyme |
| CDDP 05 | TERT | Predicted interactions | telomerase reverse transcriptase | Nucleus | enzyme |
| CDDP 06 | AKR1B1 | Predicted interactions | aldo-keto reductase family 1 member B | Cytoplasm | enzyme |
| CDDP 06 | TTR | Predicted interactions | transthyretin | Extracellular Space | transporter |
| CDDP 06 | MMP1 | Predicted interactions | matrix metallopeptidase 1 | Extracellular Space | peptidase |
| CDDP 06 | APEX1 | Predicted interactions | apurinic/apyrimidinic endodeoxyribonuclease 1 | Nucleus | enzyme |
| CDDP 06 | TDP1 | Predicted interactions | tyrosyl-DNA phosphodiesterase 1 | Nucleus | enzyme |
| CDDP 06 | PKM | Predicted interactions | pyruvate kinase M1/2 | Cytoplasm | kinase |
| CDDP 06 | KDM4E | Predicted interactions | lysine demethylase 4E | Nucleus | enzyme |
| CDDP 06 | RECQL | Predicted interactions | RecQ like helicase | Nucleus | enzyme |
| CDDP 06 | POLB | Predicted interactions | DNA polymerase beta | Nucleus | enzyme |
| CDDP 06 | GAA | Predicted interactions | glucosidase alpha, acid | Cytoplasm | enzyme |
| CDDP 06 | HSD17B10 | Predicted interactions | hydroxysteroid 17-beta dehydrogenase 10 | Cytoplasm | enzyme |
| CDDP 06 | MCL1 | Predicted interactions | MCL1 apoptosis regulator, BCL2 family member | Cytoplasm | transporter |
| CDDP 06 | NOX4 | Predicted interactions | NADPH oxidase 4 | Cytoplasm | enzyme |
| CDDP 06 | CYSLTR1 | Predicted interactions | cysteinyl leukotriene receptor 1 | Plasma Membrane | G-protein coupled receptor |
| CDDP 06 | KLK1 | Predicted interactions | kallikrein 1 | Cytoplasm | peptidase |
| CDDP 06 | KLK2 | Predicted interactions | kallikrein related peptidase 2 | Extracellular Space | peptidase |
| CDDP 06 | APP | Predicted interactions | amyloid beta precursor protein | Plasma Membrane | other |
| CDDP 06 | HSD17B2 | Predicted interactions | hydroxysteroid 17-beta dehydrogenase 2 | Cytoplasm | enzyme |
| CDDP 06 | PRKCG | Predicted interactions | protein kinase C gamma | Cytoplasm | kinase |
| CDDP 06 | PRKCD | Predicted interactions | protein kinase C delta | Cytoplasm | kinase |
| CDDP 06 | PRKCA | Predicted interactions | protein kinase C alpha | Cytoplasm | kinase |
| CDDP 06 | CDK2 | Predicted interactions | cyclin dependent kinase 2 | Nucleus | kinase |
| CDDP 06 | PRKCB | Predicted interactions | protein kinase C beta | Cytoplasm | kinase |
| CDDP 06 | SELL | Predicted interactions | selectin L | Plasma Membrane | transmembrane receptor |
| CDDP 06 | HSD17B1 | Predicted interactions | hydroxysteroid 17-beta dehydrogenase 1 | Cytoplasm | enzyme |
| CDDP 06 | PTPN1 | Predicted interactions | protein tyrosine phosphatase non-receptor type 1 | Cytoplasm | phosphatase |
| CDDP 06 | PLA2G2A | Predicted interactions | phospholipase A2 group IIA | Cytoplasm | enzyme |
| CDDP 06 | PRKCE | Predicted interactions | protein kinase C epsilon | Cytoplasm | kinase |
| CDDP 06 | PRKCH | Predicted interactions | protein kinase C eta | Cytoplasm | kinase |
| CDDP 06 | SELE | Predicted interactions | selectin E | Plasma Membrane | transmembrane receptor |
| CDDP 06 | PRKACA | Predicted interactions | protein kinase cAMP-activated catalytic subunit alpha | Cytoplasm | kinase |
| CDDP 06 | MMP12 | Predicted interactions | matrix metallopeptidase 12 | Extracellular Space | peptidase |
| CDDP 06 | ACP1 | Predicted interactions | acid phosphatase 1 | Cytoplasm | phosphatase |
| CDDP 06 | SELP | Predicted interactions | selectin P | Plasma Membrane | transmembrane receptor |
| CDDP 06 | AKR1B10 | Predicted interactions | aldo-keto reductase family 1 member B10 | Cytoplasm | enzyme |
| CDDP 07 | CA7 | Predicted interactions | carbonic anhydrase 7 | Cytoplasm | enzyme |
| CDDP 07 | CA12 | Predicted interactions | carbonic anhydrase 12 | Plasma Membrane | enzyme |
| CDDP 07 | CA4 | Predicted interactions | carbonic anhydrase 4 | Plasma Membrane | enzyme |
| CDDP 07 | APP | Predicted interactions | amyloid beta precursor protein | Plasma Membrane | other |
| CDDP 07 | MMP12 | Predicted interactions | matrix metallopeptidase 12 | Extracellular Space | peptidase |
| CDDP 07 | JUN | Predicted interactions | Jun proto-oncogene, AP-1 transcription factor subunit | Nucleus | transcription regulator |
| CDDP 07 | MMP9 | Predicted interactions | matrix metallopeptidase 9 | Extracellular Space | peptidase |
| CDDP 07 | MMP1 | Predicted interactions | matrix metallopeptidase 1 | Extracellular Space | peptidase |
| CDDP 07 | MMP3 | Predicted interactions | matrix metallopeptidase 3 | Extracellular Space | peptidase |
| CDDP 07 | KLK1 | Predicted interactions | kallikrein 1 | Cytoplasm | peptidase |
| CDDP 07 | KLK2 | Predicted interactions | kallikrein related peptidase 2 | Extracellular Space | peptidase |
| CDDP 09 | AKR1B1 | Predicted interactions | aldo-keto reductase family 1 member B | Cytoplasm | enzyme |
| CDDP 09 | TTR | Predicted interactions | transthyretin | Extracellular Space | transporter |
| CDDP 09 | APEX1 | Predicted interactions | apurinic/apyrimidinic endodeoxyribonuclease 1 | Nucleus | enzyme |
| CDDP 09 | TDP1 | Predicted interactions | tyrosyl-DNA phosphodiesterase 1 | Nucleus | enzyme |
| CDDP 09 | PKM | Predicted interactions | pyruvate kinase M1/2 | Cytoplasm | kinase |
| CDDP 09 | KDM4E | Predicted interactions | lysine demethylase 4E | Nucleus | enzyme |
| CDDP 09 | RECQL | Predicted interactions | RecQ like helicase | Nucleus | enzyme |
| CDDP 09 | POLB | Predicted interactions | DNA polymerase beta | Nucleus | enzyme |
| CDDP 09 | GAA | Predicted interactions | glucosidase alpha, acid | Cytoplasm | enzyme |
| CDDP 09 | MMP1 | Predicted interactions | matrix metallopeptidase 1 | Extracellular Space | peptidase |
| CDDP 09 | HSD17B10 | Predicted interactions | hydroxysteroid 17-beta dehydrogenase 10 | Cytoplasm | enzyme |
| CDDP 09 | MCL1 | Predicted interactions | MCL1 apoptosis regulator, BCL2 family member | Cytoplasm | transporter |
| CDDP 09 | CA7 | Predicted interactions | carbonic anhydrase 7 | Cytoplasm | enzyme |
| CDDP 09 | CA4 | Predicted interactions | carbonic anhydrase 4 | Plasma Membrane | enzyme |
| CDDP 09 | CA2 | Predicted interactions | carbonic anhydrase 2 | Cytoplasm | enzyme |
| CDDP 09 | CA12 | Predicted interactions | carbonic anhydrase 12 | Plasma Membrane | enzyme |
| CDDP 09 | SELL | Predicted interactions | selectin L | Plasma Membrane | transmembrane receptor |
| CDDP 09 | YARS | Predicted interactions | Tyrosyl-TRNA Synthetase 1 | Nucleus | ligase |
| CDDP 09 | DYRK2 | Predicted interactions | dual specificity tyrosine phosphorylation regulated kinase 2 | Cytoplasm | kinase |
| CDDP 09 | MMP12 | Predicted interactions | matrix metallopeptidase 12 | Extracellular Space | peptidase |
| CDDP 09 | SELP | Predicted interactions | selectin P | Plasma Membrane | transmembrane receptor |
| CDDP 11 | ERN1 | Predicted interactions | endoplasmic reticulum to nucleus signaling 1 | Cytoplasm | kinase |
| CDDP 11 | RECQL | Predicted interactions | RecQ like helicase | Nucleus | enzyme |
| CDDP 11 | THPO | Predicted interactions | thrombopoietin | Extracellular Space | cytokine |
| CDDP 11 | FYN | Predicted interactions | FYN proto-oncogene, Src family tyrosine kinase | Plasma Membrane | kinase |
| CDDP 11 | FOLH1 | Predicted interactions | folate hydrolase 1 | Plasma Membrane | peptidase |
| CDDP 11 | XDH | Predicted interactions | xanthine dehydrogenase | Cytoplasm | enzyme |
| CDDP 11 | KDM3A | Predicted interactions | lysine demethylase 3A | Nucleus | transcription regulator |
| CDDP 11 | KDM2A | Predicted interactions | lysine demethylase 2A | Nucleus | enzyme |
| CDDP 11 | CA2 | Predicted interactions | carbonic anhydrase 2 | Cytoplasm | enzyme |
| CDDP 11 | KDM5C | Predicted interactions | lysine demethylase 5C | Nucleus | enzyme |
| CDDP 11 | CA7 | Predicted interactions | carbonic anhydrase 7 | Cytoplasm | enzyme |
| CDDP 11 | FTO | Predicted interactions | FTO alpha-ketoglutarate dependent dioxygenase | Nucleus | enzyme |
| CDDP 11 | KDM5A | Predicted interactions | lysine demethylase 5A | Nucleus | transcription regulator |
| CDDP 11 | CA1 | Predicted interactions | carbonic anhydrase 1 | Cytoplasm | enzyme |
| CDDP 11 | CA3 | Predicted interactions | carbonic anhydrase 3 | Cytoplasm | enzyme |
| CDDP 11 | CA6 | Predicted interactions | carbonic anhydrase 6 | Extracellular Space | enzyme |
| CDDP 11 | EGLN2 | Predicted interactions | egl-9 family hypoxia inducible factor 2 | Cytoplasm | enzyme |
| CDDP 11 | DBH | Predicted interactions | dopamine beta-hydroxylase | Cytoplasm | enzyme |
| CDDP 11 | CA12 | Predicted interactions | carbonic anhydrase 12 | Plasma Membrane | enzyme |
| CDDP 11 | CA14 | Predicted interactions | carbonic anhydrase 14 | Plasma Membrane | enzyme |
| CDDP 11 | CA9 | Predicted interactions | carbonic anhydrase 9 | Nucleus | enzyme |
| CDDP 11 | FUT7 | Predicted interactions | fucosyltransferase 7 | Cytoplasm | enzyme |
| CDDP 11 | CA4 | Predicted interactions | carbonic anhydrase 4 | Plasma Membrane | enzyme |
| CDDP 11 | KDM5B | Predicted interactions | lysine demethylase 5B | Nucleus | transcription regulator |
| CDDP 11 | CA5B | Predicted interactions | carbonic anhydrase 5B | Cytoplasm | enzyme |
| CDDP 11 | HSD17B10 | Predicted interactions | hydroxysteroid 17-beta dehydrogenase 10 | Cytoplasm | enzyme |
| CDDP 11 | HIF1A | Predicted interactions | hypoxia inducible factor 1 subunit alpha | Nucleus | transcription regulator |
| CDDP 11 | CA5A | Predicted interactions | carbonic anhydrase 5A | Cytoplasm | enzyme |
| CDDP 11 | DAO | Predicted interactions | D-amino acid oxidase | Cytoplasm | enzyme |
| CDDP 11 | APEX1 | Predicted interactions | apurinic/apyrimidinic endodeoxyribonuclease 1 | Nucleus | enzyme |
| CDDP 11 | EGLN1 | Predicted interactions | egl-9 family hypoxia inducible factor 1 | Cytoplasm | enzyme |
| CDDP 11 | EGLN3 | Predicted interactions | egl-9 family hypoxia inducible factor 3 | Cytoplasm | enzyme |
| CDDP 11 | KDM4D | Predicted interactions | lysine demethylase 4D | Nucleus | enzyme |
| CDDP 11 | KDM4C | Predicted interactions | lysine demethylase 4C | Nucleus | enzyme |
| CDDP 12 | HSD17B10 | Predicted interactions | hydroxysteroid 17-beta dehydrogenase 10 | Cytoplasm | enzyme |
| CDDP 12 | CA7 | Predicted interactions | carbonic anhydrase 7 | Cytoplasm | enzyme |
| CDDP 12 | CA4 | Predicted interactions | carbonic anhydrase 4 | Plasma Membrane | enzyme |
| CDDP 12 | CA2 | Predicted interactions | carbonic anhydrase 2 | Cytoplasm | enzyme |
| CDDP 12 | CA12 | Predicted interactions | carbonic anhydrase 12 | Plasma Membrane | enzyme |
| CDDP 12 | MMP12 | Predicted interactions | matrix metallopeptidase 12 | Extracellular Space | peptidase |
| CDDP 12 | MMP9 | Predicted interactions | matrix metallopeptidase 9 | Extracellular Space | peptidase |
| CDDP 12 | MMP2 | Predicted interactions | matrix metallopeptidase 2 | Extracellular Space | peptidase |
| CDDP 12 | THPO | Predicted interactions | thrombopoietin | Extracellular Space | cytokine |
| CDDP 12 | GMNN | Predicted interactions | geminin DNA replication inhibitor | Nucleus | transcription regulator |
| CDDP 12 | TOP1 | Predicted interactions | DNA topoisomerase I | Nucleus | enzyme |
| CDDP 12 | MME | Predicted interactions | membrane metalloendopeptidase | Plasma Membrane | peptidase |
| CDDP 12 | TYR | Predicted interactions | tyrosinase | Cytoplasm | enzyme |
| CDDP 12 | MIF | Predicted interactions | macrophage migration inhibitory factor | Extracellular Space | cytokine |
| CDDP 12 | TERT | Predicted interactions | telomerase reverse transcriptase | Nucleus | enzyme |
| CDDP 12 | CA6 | Predicted interactions | carbonic anhydrase 6 | Extracellular Space | enzyme |
| CDDP 12 | NSD2 | Predicted interactions | nuclear receptor binding SET domain protein 2 | Nucleus | enzyme |
| CDDP 12 | SELL | Predicted interactions | selectin L | Plasma Membrane | transmembrane receptor |
| CDDP 12 | YARS | Predicted interactions | Tyrosyl-TRNA Synthetase 1 | Nucleus | ligase |
| CDDP 12 | NFKB1 | Predicted interactions | nuclear factor kappa B subunit 1 | Nucleus | transcription regulator |
| CDDP 12 | CA14 | Predicted interactions | carbonic anhydrase 14 | Plasma Membrane | enzyme |
| CDDP 12 | CA9 | Predicted interactions | carbonic anhydrase 9 | Nucleus | enzyme |
| CDDP 12 | MET | Predicted interactions | MET proto-oncogene, receptor tyrosine kinase | Plasma Membrane | kinase |
| CDDP 12 | CA5B | Predicted interactions | carbonic anhydrase 5B | Cytoplasm | enzyme |
| CDDP 12 | AKR1C3 | Predicted interactions | aldo-keto reductase family 1 member C3 | Cytoplasm | enzyme |
| CDDP 12 | CA5A | Predicted interactions | carbonic anhydrase 5A | Cytoplasm | enzyme |
| CDDP 12 | AKR1C4 | Predicted interactions | aldo-keto reductase family 1 member C4 | Cytoplasm | enzyme |
| CDDP 12 | SELP | Predicted interactions | selectin P | Plasma Membrane | transmembrane receptor |
| CDDP 12 | AKR1C2 | Predicted interactions | Aldo-Keto Reductase Family 1 Member C2 | Cytosol | enzyme |
| CDDP 12 | AKR1B10 | Predicted interactions | aldo-keto reductase family 1 member B10 | Cytoplasm | enzyme |
| CDDP 12 | SNCA | Predicted interactions | synuclein alpha | Cytoplasm | enzyme |
| CDDP 13 | APP | Predicted interactions | amyloid beta precursor protein | Plasma Membrane | other |
| CDDP 13 | MMP12 | Predicted interactions | matrix metallopeptidase 12 | Extracellular Space | peptidase |
| CDDP 13 | MMP3 | Predicted interactions | matrix metallopeptidase 3 | Extracellular Space | peptidase |
| CDDP 13 | MMP9 | Predicted interactions | matrix metallopeptidase 9 | Extracellular Space | peptidase |
| CDDP 13 | MMP1 | Predicted interactions | matrix metallopeptidase 1 | Extracellular Space | peptidase |
| CDDP 13 | VEGFA | Predicted interactions | vascular endothelial growth factor A | Extracellular Space | growth factor |
| CDDP 13 | KLK1 | Predicted interactions | kallikrein 1 | Cytoplasm | peptidase |
| CDDP 13 | KLK2 | Predicted interactions | kallikrein related peptidase 2 | Extracellular Space | peptidase |
| CDDP 13 | PRKCG | Predicted interactions | protein kinase C gamma | Cytoplasm | kinase |
| CDDP 13 | PRKCD | Predicted interactions | protein kinase C delta | Cytoplasm | kinase |
| CDDP 13 | PRKCA | Predicted interactions | protein kinase C alpha | Cytoplasm | kinase |
| CDDP 13 | ABCC1 | Predicted interactions | ATP binding cassette subfamily C member 1 | Plasma Membrane | transporter |
| CDDP 13 | PRKCB | Predicted interactions | protein kinase C beta | Cytoplasm | kinase |
| CDDP 13 | PRKCE | Predicted interactions | protein kinase C epsilon | Cytoplasm | kinase |
| CDDP 13 | PRKCH | Predicted interactions | protein kinase C eta | Cytoplasm | kinase |
| CDDP 13 | PRKACA | Predicted interactions | protein kinase cAMP-activated catalytic subunit alpha | Cytoplasm | kinase |
| CDDP 13 | JUN | Predicted interactions | Jun proto-oncogene, AP-1 transcription factor subunit | Nucleus | transcription regulator |
| CDDP 14 | STAT3 | Predicted interactions | signal transducer and activator of transcription 3 | Nucleus | transcription regulator |
| CDDP 14 | L3MBTL1 | Predicted interactions | L3MBTL histone methyl-lysine binding protein 1 | Nucleus | other |
| CDDP 14 | USP2 | Predicted interactions | ubiquitin specific peptidase 2 | Cytoplasm | peptidase |
| CDDP 14 | F2 | Predicted interactions | coagulation factor II, thrombin | Extracellular Space | peptidase |
| CDDP 14 | PAX8 | Predicted interactions | paired box 8 | Nucleus | transcription regulator |
| CDDP 14 | TP53 | Predicted interactions | tumor protein p53 | Nucleus | transcription regulator |
| CDDP 14 | NPSR1 | Predicted interactions | neuropeptide S receptor 1 | Plasma Membrane | G-protein coupled receptor |
| CDDP 14 | IL2 | Predicted interactions | interleukin 2 | Extracellular Space | cytokine |
| CDDP 14 | SLCO1B1 | Predicted interactions | solute carrier organic anion transporter family member 1B1 | Plasma Membrane | transporter |
| CDDP 14 | SLCO1B3 | Predicted interactions | solute carrier organic anion transporter family member 1B3 | Plasma Membrane | transporter |
| CDDP 14 | HSD11B2 | Predicted interactions | hydroxysteroid 11-beta dehydrogenase 2 | Cytoplasm | enzyme |
| CDDP 15 | PAX8 | Predicted interactions | paired box 8 | Nucleus | transcription regulator |
| CDDP 15 | STAT3 | Predicted interactions | signal transducer and activator of transcription 3 | Nucleus | transcription regulator |
| CDDP 15 | L3MBTL1 | Predicted interactions | L3MBTL histone methyl-lysine binding protein 1 | Nucleus | other |
| CDDP 15 | USP2 | Predicted interactions | ubiquitin specific peptidase 2 | Cytoplasm | peptidase |
| CDDP 15 | F2 | Predicted interactions | coagulation factor II, thrombin | Extracellular Space | peptidase |
| CDDP 15 | TP53 | Predicted interactions | tumor protein p53 | Nucleus | transcription regulator |
| CDDP 15 | NPSR1 | Predicted interactions | neuropeptide S receptor 1 | Plasma Membrane | G-protein coupled receptor |
| CDDP 15 | IL2 | Predicted interactions | interleukin 2 | Extracellular Space | cytokine |
| CDDP 15 | SLCO1B3 | Predicted interactions | solute carrier organic anion transporter family member 1B3 | Plasma Membrane | transporter |
| CDDP 16 | L3MBTL1 | Predicted interactions | L3MBTL histone methyl-lysine binding protein 1 | Nucleus | other |
| CDDP 16 | STAT3 | Predicted interactions | signal transducer and activator of transcription 3 | Nucleus | transcription regulator |
| CDDP 16 | KLF5 | Predicted interactions | Kruppel like factor 5 | Nucleus | transcription regulator |
| CDDP 16 | GMNN | Predicted interactions | geminin DNA replication inhibitor | Nucleus | transcription regulator |
| CDDP 16 | NR1H3 | Predicted interactions | nuclear receptor subfamily 1 group H member 3 | Nucleus | ligand-dependent nuclear receptor |
| CDDP 16 | NFKB1 | Predicted interactions | nuclear factor kappa B subunit 1 | Nucleus | transcription regulator |
| CDDP 16 | HIF1A | Predicted interactions | hypoxia inducible factor 1 subunit alpha | Nucleus | transcription regulator |
| CDDP 16 | IL2 | Predicted interactions | interleukin 2 | Extracellular Space | cytokine |
| CDDP 17 | PAX8 | Predicted interactions | paired box 8 | Nucleus | transcription regulator |
| CDDP 17 | STAT3 | Predicted interactions | signal transducer and activator of transcription 3 | Nucleus | transcription regulator |
| CDDP 17 | L3MBTL1 | Predicted interactions | L3MBTL histone methyl-lysine binding protein 1 | Nucleus | other |
| CDDP 17 | USP2 | Predicted interactions | ubiquitin specific peptidase 2 | Cytoplasm | peptidase |
| CDDP 17 | F2 | Predicted interactions | coagulation factor II, thrombin | Extracellular Space | peptidase |
| CDDP 17 | TP53 | Predicted interactions | tumor protein p53 | Nucleus | transcription regulator |
| CDDP 17 | NPSR1 | Predicted interactions | neuropeptide S receptor 1 | Plasma Membrane | G-protein coupled receptor |
| CDDP 17 | IL2 | Predicted interactions | interleukin 2 | Extracellular Space | cytokine |
| CDDP 17 | SLCO1B1 | Predicted interactions | solute carrier organic anion transporter family member 1B1 | Plasma Membrane | transporter |
| CDDP 17 | RORC | Predicted interactions | RAR related orphan receptor C | Nucleus | ligand-dependent nuclear receptor |
| CDDP 17 | SLCO1B3 | Predicted interactions | solute carrier organic anion transporter family member 1B3 | Plasma Membrane | transporter |
| CDDP 17 | HSD11B2 | Predicted interactions | hydroxysteroid 11-beta dehydrogenase 2 | Cytoplasm | enzyme |
| CDDP 17 | PTPN2 | Predicted interactions | protein tyrosine phosphatase non-receptor type 2 | Cytoplasm | phosphatase |
| CDDP 17 | HBB | Predicted interactions | hemoglobin subunit beta | Cytoplasm | transporter |
| CDDP 18 | L3MBTL1 | Predicted interactions | L3MBTL histone methyl-lysine binding protein 1 | Nucleus | other |
| CDDP 18 | STAT3 | Predicted interactions | signal transducer and activator of transcription 3 | Nucleus | transcription regulator |
| CDDP 18 | PAX8 | Predicted interactions | paired box 8 | Nucleus | transcription regulator |
| CDDP 18 | USP2 | Predicted interactions | ubiquitin specific peptidase 2 | Cytoplasm | peptidase |
| CDDP 18 | F2 | Predicted interactions | coagulation factor II, thrombin | Extracellular Space | peptidase |
| CDDP 18 | TP53 | Predicted interactions | tumor protein p53 | Nucleus | transcription regulator |
| CDDP 18 | NPSR1 | Predicted interactions | neuropeptide S receptor 1 | Plasma Membrane | G-protein coupled receptor |
| CDDP 18 | IL2 | Predicted interactions | interleukin 2 | Extracellular Space | cytokine |
| CDDP 18 | SLCO1B1 | Predicted interactions | solute carrier organic anion transporter family member 1B1 | Plasma Membrane | transporter |
| CDDP 18 | RORC | Predicted interactions | RAR related orphan receptor C | Nucleus | ligand-dependent nuclear receptor |
| CDDP 18 | SLCO1B3 | Predicted interactions | solute carrier organic anion transporter family member 1B3 | Plasma Membrane | transporter |
| CDDP 18 | HSD11B2 | Predicted interactions | hydroxysteroid 11-beta dehydrogenase 2 | Cytoplasm | enzyme |
| CDDP 18 | HBB | Predicted interactions | hemoglobin subunit beta | Cytoplasm | transporter |
| CDDP 19 | L3MBTL1 | Predicted interactions | L3MBTL histone methyl-lysine binding protein 1 | Nucleus | other |
| CDDP 19 | PAX8 | Predicted interactions | paired box 8 | Nucleus | transcription regulator |
| CDDP 19 | STAT3 | Predicted interactions | signal transducer and activator of transcription 3 | Nucleus | transcription regulator |
| CDDP 19 | USP2 | Predicted interactions | ubiquitin specific peptidase 2 | Cytoplasm | peptidase |
| CDDP 19 | F2 | Predicted interactions | coagulation factor II, thrombin | Extracellular Space | peptidase |
| CDDP 19 | TP53 | Predicted interactions | tumor protein p53 | Nucleus | transcription regulator |
| CDDP 19 | NPSR1 | Predicted interactions | neuropeptide S receptor 1 | Plasma Membrane | G-protein coupled receptor |
| CDDP 19 | IL2 | Predicted interactions | interleukin 2 | Extracellular Space | cytokine |
| CDDP 19 | SLCO1B3 | Predicted interactions | solute carrier organic anion transporter family member 1B3 | Plasma Membrane | transporter |
| CDDP 19 | ADRA2B | Predicted interactions | adrenoceptor alpha 2B | Plasma Membrane | G-protein coupled receptor |
| CDDP 20 | CDC25A | Predicted interactions | cell division cycle 25A | Nucleus | phosphatase |
| CDDP 20 | CDC25B | Predicted interactions | cell division cycle 25B | Nucleus | phosphatase |
| CDDP 20 | THPO | Predicted interactions | thrombopoietin | Extracellular Space | cytokine |
| CDDP 20 | APOBEC3A | Predicted interactions | apolipoprotein B mRNA editing enzyme catalytic subunit 3A | Cytoplasm | enzyme |
| CDDP 20 | CHRNA7 | Predicted interactions | cholinergic receptor nicotinic alpha 7 subunit | Plasma Membrane | transmembrane receptor |
| CDDP 21 | CDC25A | Predicted interactions | cell division cycle 25A | Nucleus | phosphatase |
| CDDP 21 | CDC25B | Predicted interactions | cell division cycle 25B | Nucleus | phosphatase |
| CDDP 21 | THPO | Predicted interactions | thrombopoietin | Extracellular Space | cytokine |
| CDDP 21 | APOBEC3A | Predicted interactions | apolipoprotein B mRNA editing enzyme catalytic subunit 3A | Cytoplasm | enzyme |
| CDDP 21 | CHRNA7 | Predicted interactions | cholinergic receptor nicotinic alpha 7 subunit | Plasma Membrane | transmembrane receptor |
| CDDP 21 | NFKB1 | Predicted interactions | nuclear factor kappa B subunit 1 | Nucleus | transcription regulator |
| CDDP 22 | RECQL | Predicted interactions | RecQ like helicase | Nucleus | enzyme |
| CDDP 22 | FYN | Predicted interactions | FYN proto-oncogene, Src family tyrosine kinase | Plasma Membrane | kinase |
| CDDP 22 | SRD5A2 | Predicted interactions | steroid 5 alpha-reductase 2 | Cytoplasm | enzyme |
| CDDP 22 | MIF | Predicted interactions | macrophage migration inhibitory factor | Extracellular Space | cytokine |
| CDDP 22 | CAPN2 | Predicted interactions | calpain 2 | Cytoplasm | peptidase |
| CDDP 22 | PTPRC | Predicted interactions | protein tyrosine phosphatase receptor type C | Plasma Membrane | phosphatase |
| CDDP 22 | NR1I2 | Predicted interactions | nuclear receptor subfamily 1 group I member 2 | Nucleus | ligand-dependent nuclear receptor |
| CDDP 22 | QDPR | Predicted interactions | quinoid dihydropteridine reductase | Cytoplasm | enzyme |
| CDDP 22 | CTBP2 | Predicted interactions | C-terminal binding protein 2 | Nucleus | transcription regulator |
| CDDP 22 | CAPN1 | Predicted interactions | calpain 1 | Cytoplasm | peptidase |
| CDDP 22 | NQO2 | Predicted interactions | N-ribosyldihydronicotinamide:quinone reductase 2 | Cytoplasm | enzyme |
| CDDP 22 | DAO | Predicted interactions | D-amino acid oxidase | Cytoplasm | enzyme |
| CDDP 23 | APAF1 | Predicted interactions | apoptotic peptidase activating factor 1 | Cytoplasm | other |
| CDDP 23 | AKR1B1 | Predicted interactions | aldo-keto reductase family 1 member B | Cytoplasm | enzyme |
| CDDP 23 | CES1 | Predicted interactions | carboxylesterase 1 | Cytoplasm | enzyme |
| CDDP 23 | TERT | Predicted interactions | telomerase reverse transcriptase | Nucleus | enzyme |
| CDDP 23 | PTPN6 | Predicted interactions | protein tyrosine phosphatase non-receptor type 6 | Cytoplasm | phosphatase |
| CDDP 23 | CES2 | Predicted interactions | carboxylesterase 2 | Cytoplasm | enzyme |
| CDDP 23 | PTPN11 | Predicted interactions | protein tyrosine phosphatase non-receptor type 11 | Cytoplasm | phosphatase |
| CDDP 23 | ALDH1A1 | Predicted interactions | aldehyde dehydrogenase 1 family member A1 | Cytoplasm | enzyme |
| CDDP 23 | ELANE | Predicted interactions | elastase, neutrophil expressed | Extracellular Space | peptidase |
| CDDP 23 | CDC25A | Predicted interactions | cell division cycle 25A | Nucleus | phosphatase |
| CDDP 23 | STAT3 | Predicted interactions | signal transducer and activator of transcription 3 | Nucleus | transcription regulator |
| CDDP 23 | CDC25B | Predicted interactions | cell division cycle 25B | Nucleus | phosphatase |
| CDDP 24 | ERN1 | Predicted interactions | endoplasmic reticulum to nucleus signaling 1 | Cytoplasm | kinase |
| CDDP 24 | APAF1 | Predicted interactions | apoptotic peptidase activating factor 1 | Cytoplasm | other |
| CDDP 24 | AKR1B1 | Predicted interactions | aldo-keto reductase family 1 member B | Cytoplasm | enzyme |
| CDDP 24 | CES1 | Predicted interactions | carboxylesterase 1 | Cytoplasm | enzyme |
| CDDP 24 | ELANE | Predicted interactions | elastase, neutrophil expressed | Extracellular Space | peptidase |
| CDDP 24 | TERT | Predicted interactions | telomerase reverse transcriptase | Nucleus | enzyme |
| CDDP 24 | PTPN6 | Predicted interactions | protein tyrosine phosphatase non-receptor type 6 | Cytoplasm | phosphatase |
| CDDP 24 | CES2 | Predicted interactions | carboxylesterase 2 | Cytoplasm | enzyme |
| CDDP 24 | PTPN11 | Predicted interactions | protein tyrosine phosphatase non-receptor type 11 | Cytoplasm | phosphatase |
| CDDP 25 | CA7 | Predicted interactions | carbonic anhydrase 7 | Cytoplasm | enzyme |
| CDDP 25 | CA4 | Predicted interactions | carbonic anhydrase 4 | Plasma Membrane | enzyme |
| CDDP 25 | CA12 | Predicted interactions | carbonic anhydrase 12 | Plasma Membrane | enzyme |
| CDDP 25 | APP | Predicted interactions | amyloid beta precursor protein | Plasma Membrane | other |
| CDDP 25 | CA2 | Predicted interactions | carbonic anhydrase 2 | Cytoplasm | enzyme |
| CDDP 25 | VCP | Predicted interactions | valosin containing protein | Cytoplasm | enzyme |
| CDDP 25 | VEGFA | Predicted interactions | vascular endothelial growth factor A | Extracellular Space | growth factor |
| CDDP 25 | CYSLTR1 | Predicted interactions | cysteinyl leukotriene receptor 1 | Plasma Membrane | G-protein coupled receptor |
| CDDP 25 | AKR1B1 | Predicted interactions | aldo-keto reductase family 1 member B | Cytoplasm | enzyme |
| CDDP 25 | KLK1 | Predicted interactions | kallikrein 1 | Cytoplasm | peptidase |
| CDDP 25 | KLK2 | Predicted interactions | kallikrein related peptidase 2 | Extracellular Space | peptidase |
| CDDP 25 | PRKCG | Predicted interactions | protein kinase C gamma | Cytoplasm | kinase |
| CDDP 25 | PRKCD | Predicted interactions | protein kinase C delta | Cytoplasm | kinase |
| CDDP 25 | PRKCA | Predicted interactions | protein kinase C alpha | Cytoplasm | kinase |
| CDDP 25 | CDK2 | Predicted interactions | cyclin dependent kinase 2 | Nucleus | kinase |
| CDDP 25 | PRKCB | Predicted interactions | protein kinase C beta | Cytoplasm | kinase |
| CDDP 25 | SELL | Predicted interactions | selectin L | Plasma Membrane | transmembrane receptor |
| CDDP 25 | TTR | Predicted interactions | transthyretin | Extracellular Space | transporter |
| CDDP 25 | MMP1 | Predicted interactions | matrix metallopeptidase 1 | Extracellular Space | peptidase |
| CDDP 25 | PTPN1 | Predicted interactions | protein tyrosine phosphatase non-receptor type 1 | Cytoplasm | phosphatase |
| CDDP 25 | PLA2G2A | Predicted interactions | phospholipase A2 group IIA | Cytoplasm | enzyme |
| CDDP 25 | PTPRF | Predicted interactions | protein tyrosine phosphatase receptor type F | Plasma Membrane | phosphatase |
| CDDP 25 | PRKCE | Predicted interactions | protein kinase C epsilon | Cytoplasm | kinase |
| CDDP 25 | PTPN2 | Predicted interactions | protein tyrosine phosphatase non-receptor type 2 | Cytoplasm | phosphatase |
| CDDP 25 | SELE | Predicted interactions | selectin E | Plasma Membrane | transmembrane receptor |
| CDDP 25 | MMP12 | Predicted interactions | matrix metallopeptidase 12 | Extracellular Space | peptidase |
| CDDP 25 | ACP1 | Predicted interactions | acid phosphatase 1 | Cytoplasm | phosphatase |
| CDDP 25 | SELP | Predicted interactions | selectin P | Plasma Membrane | transmembrane receptor |
| CDDP 25 | APEX1 | Predicted interactions | apurinic/apyrimidinic endodeoxyribonuclease 1 | Nucleus | enzyme |
| CDDP 26 | L3MBTL1 | Predicted interactions | L3MBTL histone methyl-lysine binding protein 1 | Nucleus | other |
| CDDP 26 | STAT3 | Predicted interactions | signal transducer and activator of transcription 3 | Nucleus | transcription regulator |
| CDDP 26 | USP2 | Predicted interactions | ubiquitin specific peptidase 2 | Cytoplasm | peptidase |
| CDDP 26 | F2 | Predicted interactions | coagulation factor II, thrombin | Extracellular Space | peptidase |
| CDDP 26 | PAX8 | Predicted interactions | paired box 8 | Nucleus | transcription regulator |
| CDDP 26 | TP53 | Predicted interactions | tumor protein p53 | Nucleus | transcription regulator |
| CDDP 26 | NPSR1 | Predicted interactions | neuropeptide S receptor 1 | Plasma Membrane | G-protein coupled receptor |
| CDDP 26 | IL2 | Predicted interactions | interleukin 2 | Extracellular Space | cytokine |
| CDDP 26 | SLCO1B1 | Predicted interactions | solute carrier organic anion transporter family member 1B1 | Plasma Membrane | transporter |
| CDDP 26 | RORC | Predicted interactions | RAR related orphan receptor C | Nucleus | ligand-dependent nuclear receptor |
| CDDP 26 | SLCO1B3 | Predicted interactions | solute carrier organic anion transporter family member 1B3 | Plasma Membrane | transporter |
| CDDP 26 | HSD11B2 | Predicted interactions | hydroxysteroid 11-beta dehydrogenase 2 | Cytoplasm | enzyme |
| CDDP 26 | HBB | Predicted interactions | hemoglobin subunit beta | Cytoplasm | transporter |
| CDDP 27 | STAT3 | Predicted interactions | signal transducer and activator of transcription 3 | Nucleus | transcription regulator |
| CDDP 27 | IL2 | Predicted interactions | interleukin 2 | Extracellular Space | cytokine |
| CDDP 27 | L3MBTL1 | Predicted interactions | L3MBTL histone methyl-lysine binding protein 1 | Nucleus | other |
| CDDP 27 | USP2 | Predicted interactions | ubiquitin specific peptidase 2 | Cytoplasm | peptidase |
| CDDP 27 | F2 | Predicted interactions | coagulation factor II, thrombin | Extracellular Space | peptidase |
| CDDP 27 | PAX8 | Predicted interactions | paired box 8 | Nucleus | transcription regulator |
| CDDP 27 | TP53 | Predicted interactions | tumor protein p53 | Nucleus | transcription regulator |
| CDDP 27 | NPSR1 | Predicted interactions | neuropeptide S receptor 1 | Plasma Membrane | G-protein coupled receptor |
| CDDP 27 | SLCO1B1 | Predicted interactions | solute carrier organic anion transporter family member 1B1 | Plasma Membrane | transporter |
| CDDP 27 | RORC | Predicted interactions | RAR related orphan receptor C | Nucleus | ligand-dependent nuclear receptor |
| CDDP 27 | SLCO1B3 | Predicted interactions | solute carrier organic anion transporter family member 1B3 | Plasma Membrane | transporter |
| CDDP 27 | PTPN1 | Predicted interactions | protein tyrosine phosphatase non-receptor type 1 | Cytoplasm | phosphatase |
| CDDP 27 | HSD11B2 | Predicted interactions | hydroxysteroid 11-beta dehydrogenase 2 | Cytoplasm | enzyme |
| CDDP 27 | PTPN2 | Predicted interactions | protein tyrosine phosphatase non-receptor type 2 | Cytoplasm | phosphatase |
| CDDP 27 | HBB | Predicted interactions | hemoglobin subunit beta | Cytoplasm | transporter |
| CDDP 28 | L3MBTL1 | Predicted interactions | L3MBTL histone methyl-lysine binding protein 1 | Nucleus | other |
| CDDP 28 | STAT3 | Predicted interactions | signal transducer and activator of transcription 3 | Nucleus | transcription regulator |
| CDDP 28 | KLF5 | Predicted interactions | Kruppel like factor 5 | Nucleus | transcription regulator |
| CDDP 28 | GMNN | Predicted interactions | geminin DNA replication inhibitor | Nucleus | transcription regulator |
| CDDP 28 | SLCO1B1 | Predicted interactions | solute carrier organic anion transporter family member 1B1 | Plasma Membrane | transporter |
| CDDP 28 | SLCO1B3 | Predicted interactions | solute carrier organic anion transporter family member 1B3 | Plasma Membrane | transporter |
| CDDP 28 | NR1H3 | Predicted interactions | nuclear receptor subfamily 1 group H member 3 | Nucleus | ligand-dependent nuclear receptor |
| CDDP 28 | NFKB1 | Predicted interactions | nuclear factor kappa B subunit 1 | Nucleus | transcription regulator |
| CDDP 28 | HIF1A | Predicted interactions | hypoxia inducible factor 1 subunit alpha | Nucleus | transcription regulator |
| CDDP 28 | HBB | Predicted interactions | hemoglobin subunit beta | Cytoplasm | transporter |
| CDDP 28 | IL2 | Predicted interactions | interleukin 2 | Extracellular Space | cytokine |
| CDDP 29 | PAX8 | Predicted interactions | paired box 8 | Nucleus | transcription regulator |
| CDDP 29 | STAT3 | Predicted interactions | signal transducer and activator of transcription 3 | Nucleus | transcription regulator |
| CDDP 29 | L3MBTL1 | Predicted interactions | L3MBTL histone methyl-lysine binding protein 1 | Nucleus | other |
| CDDP 29 | USP2 | Predicted interactions | ubiquitin specific peptidase 2 | Cytoplasm | peptidase |
| CDDP 29 | F2 | Predicted interactions | coagulation factor II, thrombin | Extracellular Space | peptidase |
| CDDP 29 | TP53 | Predicted interactions | tumor protein p53 | Nucleus | transcription regulator |
| CDDP 29 | NPSR1 | Predicted interactions | neuropeptide S receptor 1 | Plasma Membrane | G-protein coupled receptor |
| CDDP 29 | IL2 | Predicted interactions | interleukin 2 | Extracellular Space | cytokine |
| CDDP 29 | SLCO1B3 | Predicted interactions | solute carrier organic anion transporter family member 1B3 | Plasma Membrane | transporter |
| CDDP 30 | PAX8 | Predicted interactions | paired box 8 | Nucleus | transcription regulator |
| CDDP 30 | STAT3 | Predicted interactions | signal transducer and activator of transcription 3 | Nucleus | transcription regulator |
| CDDP 30 | L3MBTL1 | Predicted interactions | L3MBTL histone methyl-lysine binding protein 1 | Nucleus | other |
| CDDP 30 | USP2 | Predicted interactions | ubiquitin specific peptidase 2 | Cytoplasm | peptidase |
| CDDP 30 | F2 | Predicted interactions | coagulation factor II, thrombin | Extracellular Space | peptidase |
| CDDP 30 | TP53 | Predicted interactions | tumor protein p53 | Nucleus | transcription regulator |
| CDDP 30 | NPSR1 | Predicted interactions | neuropeptide S receptor 1 | Plasma Membrane | G-protein coupled receptor |
| CDDP 30 | IL2 | Predicted interactions | interleukin 2 | Extracellular Space | cytokine |
| CDDP 30 | SLCO1B3 | Predicted interactions | solute carrier organic anion transporter family member 1B3 | Plasma Membrane | transporter |
| CDDP 31 | L3MBTL1 | Predicted interactions | L3MBTL histone methyl-lysine binding protein 1 | Nucleus | other |
| CDDP 31 | STAT3 | Predicted interactions | signal transducer and activator of transcription 3 | Nucleus | transcription regulator |
| CDDP 31 | USP2 | Predicted interactions | ubiquitin specific peptidase 2 | Cytoplasm | peptidase |
| CDDP 31 | F2 | Predicted interactions | coagulation factor II, thrombin | Extracellular Space | peptidase |
| CDDP 31 | SLCO1B1 | Predicted interactions | solute carrier organic anion transporter family member 1B1 | Plasma Membrane | transporter |
| CDDP 31 | RORC | Predicted interactions | RAR related orphan receptor C | Nucleus | ligand-dependent nuclear receptor |
| CDDP 31 | SLCO1B3 | Predicted interactions | solute carrier organic anion transporter family member 1B3 | Plasma Membrane | transporter |
| CDDP 31 | HSD11B2 | Predicted interactions | hydroxysteroid 11-beta dehydrogenase 2 | Cytoplasm | enzyme |
| CDDP 31 | HBB | Predicted interactions | hemoglobin subunit beta | Cytoplasm | transporter |
| CDDP 32 | L3MBTL1 | Predicted interactions | L3MBTL histone methyl-lysine binding protein 1 | Nucleus | other |
| CDDP 32 | STAT3 | Predicted interactions | signal transducer and activator of transcription 3 | Nucleus | transcription regulator |
| CDDP 32 | USP2 | Predicted interactions | ubiquitin specific peptidase 2 | Cytoplasm | peptidase |
| CDDP 32 | F2 | Predicted interactions | coagulation factor II, thrombin | Extracellular Space | peptidase |
| CDDP 32 | PAX8 | Predicted interactions | paired box 8 | Nucleus | transcription regulator |
| CDDP 32 | TP53 | Predicted interactions | tumor protein p53 | Nucleus | transcription regulator |
| CDDP 32 | NPSR1 | Predicted interactions | neuropeptide S receptor 1 | Plasma Membrane | G-protein coupled receptor |
| CDDP 32 | IL2 | Predicted interactions | interleukin 2 | Extracellular Space | cytokine |
| CDDP 32 | SLCO1B1 | Predicted interactions | solute carrier organic anion transporter family member 1B1 | Plasma Membrane | transporter |
| CDDP 32 | RORC | Predicted interactions | RAR related orphan receptor C | Nucleus | ligand-dependent nuclear receptor |
| CDDP 32 | SLCO1B3 | Predicted interactions | solute carrier organic anion transporter family member 1B3 | Plasma Membrane | transporter |
| CDDP 32 | HSD11B2 | Predicted interactions | hydroxysteroid 11-beta dehydrogenase 2 | Cytoplasm | enzyme |
| CDDP 32 | HBB | Predicted interactions | hemoglobin subunit beta | Cytoplasm | transporter |
| CDDP 34 | L3MBTL1 | Predicted interactions | L3MBTL histone methyl-lysine binding protein 1 | Nucleus | other |
| CDDP 34 | STAT3 | Predicted interactions | signal transducer and activator of transcription 3 | Nucleus | transcription regulator |
| CDDP 34 | IL2 | Predicted interactions | interleukin 2 | Extracellular Space | cytokine |
| CDDP 34 | USP2 | Predicted interactions | ubiquitin specific peptidase 2 | Cytoplasm | peptidase |
| CDDP 34 | F2 | Predicted interactions | coagulation factor II, thrombin | Extracellular Space | peptidase |
| CDDP 34 | PAX8 | Predicted interactions | paired box 8 | Nucleus | transcription regulator |
| CDDP 34 | TP53 | Predicted interactions | tumor protein p53 | Nucleus | transcription regulator |
| CDDP 34 | NPSR1 | Predicted interactions | neuropeptide S receptor 1 | Plasma Membrane | G-protein coupled receptor |
| CDDP 34 | SLCO1B1 | Predicted interactions | solute carrier organic anion transporter family member 1B1 | Plasma Membrane | transporter |
| CDDP 34 | RORC | Predicted interactions | RAR related orphan receptor C | Nucleus | ligand-dependent nuclear receptor |
| CDDP 34 | SLCO1B3 | Predicted interactions | solute carrier organic anion transporter family member 1B3 | Plasma Membrane | transporter |
| CDDP 34 | PTPN1 | Predicted interactions | protein tyrosine phosphatase non-receptor type 1 | Cytoplasm | phosphatase |
| CDDP 34 | HSD11B2 | Predicted interactions | hydroxysteroid 11-beta dehydrogenase 2 | Cytoplasm | enzyme |
| CDDP 34 | PTPN2 | Predicted interactions | protein tyrosine phosphatase non-receptor type 2 | Cytoplasm | phosphatase |
| CDDP 34 | HBB | Predicted interactions | hemoglobin subunit beta | Cytoplasm | transporter |
| CDDP 35 | THPO | Predicted interactions | thrombopoietin | Extracellular Space | cytokine |
| CDDP 35 | APAF1 | Predicted interactions | apoptotic peptidase activating factor 1 | Cytoplasm | other |
| CDDP 35 | AKR1B1 | Predicted interactions | aldo-keto reductase family 1 member B | Cytoplasm | enzyme |
| CDDP 35 | TDP2 | Predicted interactions | tyrosyl-DNA phosphodiesterase 2 | Cytoplasm | transcription regulator |
| CDDP 35 | GLA | Predicted interactions | galactosidase alpha | Cytoplasm | enzyme |
| CDDP 35 | TERT | Predicted interactions | telomerase reverse transcriptase | Nucleus | enzyme |
| CDDP 35 | PTPN6 | Predicted interactions | protein tyrosine phosphatase non-receptor type 6 | Cytoplasm | phosphatase |
| CDDP 35 | NFKB1 | Predicted interactions | nuclear factor kappa B subunit 1 | Nucleus | transcription regulator |
| CDDP 35 | NQO1 | Predicted interactions | NAD(P)H quinone dehydrogenase 1 | Cytoplasm | enzyme |
| CDDP 35 | MALT1 | Predicted interactions | MALT1 paracaspase | Cytoplasm | peptidase |
| CDDP 35 | PTPN11 | Predicted interactions | protein tyrosine phosphatase non-receptor type 11 | Cytoplasm | phosphatase |
| CDDP 35 | STAT3 | Predicted interactions | signal transducer and activator of transcription 3 | Nucleus | transcription regulator |
| CDDP 35 | IDO1 | Predicted interactions | indoleamine 2,3-dioxygenase 1 | Cytoplasm | enzyme |
| CDDP 36 | CA3 | Predicted interactions | carbonic anhydrase 3 | Cytoplasm | enzyme |
| CDDP 36 | CA5B | Predicted interactions | carbonic anhydrase 5B | Cytoplasm | enzyme |
| CDDP 36 | HSD17B10 | Predicted interactions | hydroxysteroid 17-beta dehydrogenase 10 | Cytoplasm | enzyme |
| CDDP 36 | CA5A | Predicted interactions | carbonic anhydrase 5A | Cytoplasm | enzyme |
| CDDP 36 | PTPN1 | Predicted interactions | protein tyrosine phosphatase non-receptor type 1 | Cytoplasm | phosphatase |
| CDDP 36 | ERN1 | Predicted interactions | endoplasmic reticulum to nucleus signaling 1 | Cytoplasm | kinase |
| CDDP 36 | RECQL | Predicted interactions | RecQ like helicase | Nucleus | enzyme |
| CDDP 36 | THPO | Predicted interactions | thrombopoietin | Extracellular Space | cytokine |
| CDDP 36 | FYN | Predicted interactions | FYN proto-oncogene, Src family tyrosine kinase | Plasma Membrane | kinase |
| CDDP 36 | XDH | Predicted interactions | xanthine dehydrogenase | Cytoplasm | enzyme |
| CDDP 36 | KDM3A | Predicted interactions | lysine demethylase 3A | Nucleus | transcription regulator |
| CDDP 36 | KDM2A | Predicted interactions | lysine demethylase 2A | Nucleus | enzyme |
| CDDP 36 | KDM5C | Predicted interactions | lysine demethylase 5C | Nucleus | enzyme |
| CDDP 36 | FTO | Predicted interactions | FTO alpha-ketoglutarate dependent dioxygenase | Nucleus | enzyme |
| CDDP 36 | KDM5A | Predicted interactions | lysine demethylase 5A | Nucleus | transcription regulator |
| CDDP 36 | EGLN2 | Predicted interactions | egl-9 family hypoxia inducible factor 2 | Cytoplasm | enzyme |
| CDDP 36 | DBH | Predicted interactions | dopamine beta-hydroxylase | Cytoplasm | enzyme |
| CDDP 36 | NSD2 | Predicted interactions | nuclear receptor binding SET domain protein 2 | Nucleus | enzyme |
| CDDP 36 | KDM5B | Predicted interactions | lysine demethylase 5B | Nucleus | transcription regulator |
| CDDP 36 | HCAR3 | Predicted interactions | hydroxycarboxylic acid receptor 3 | Plasma Membrane | other |
| CDDP 36 | APEX1 | Predicted interactions | apurinic/apyrimidinic endodeoxyribonuclease 1 | Nucleus | enzyme |
| CDDP 36 | EGLN3 | Predicted interactions | egl-9 family hypoxia inducible factor 3 | Cytoplasm | enzyme |
| CDDP 36 | KDM4A | Predicted interactions | lysine demethylase 4A | Nucleus | transcription regulator |
| CDDP 36 | KDM4D | Predicted interactions | lysine demethylase 4D | Nucleus | enzyme |
| CDDP 36 | KDM4C | Predicted interactions | lysine demethylase 4C | Nucleus | enzyme |
| CDDP 37 | DBH | Predicted interactions | dopamine beta-hydroxylase | Cytoplasm | enzyme |
| CDDP 37 | CA13 | Predicted interactions | carbonic anhydrase 13 | Cytoplasm | enzyme |
| CDDP 37 | DAO | Predicted interactions | D-amino acid oxidase | Cytoplasm | enzyme |
| CDDP 38 | CA2 | Predicted interactions | carbonic anhydrase 2 | Cytoplasm | enzyme |
| CDDP 38 | CA7 | Predicted interactions | carbonic anhydrase 7 | Cytoplasm | enzyme |
| CDDP 38 | CA1 | Predicted interactions | carbonic anhydrase 1 | Cytoplasm | enzyme |
| CDDP 38 | CA3 | Predicted interactions | carbonic anhydrase 3 | Cytoplasm | enzyme |
| CDDP 38 | CA6 | Predicted interactions | carbonic anhydrase 6 | Extracellular Space | enzyme |
| CDDP 38 | CA12 | Predicted interactions | carbonic anhydrase 12 | Plasma Membrane | enzyme |
| CDDP 38 | CA14 | Predicted interactions | carbonic anhydrase 14 | Plasma Membrane | enzyme |
| CDDP 38 | CA9 | Predicted interactions | carbonic anhydrase 9 | Nucleus | enzyme |
| CDDP 38 | CA4 | Predicted interactions | carbonic anhydrase 4 | Plasma Membrane | enzyme |
| CDDP 38 | CA5A | Predicted interactions | carbonic anhydrase 5A | Cytoplasm | enzyme |
| CDDP 38 | ALOX15 | Predicted interactions | arachidonate 15-lipoxygenase | Cytoplasm | enzyme |
| CDDP 38 | CYP1A2 | Predicted interactions | cytochrome P450 family 1 subfamily A member 2 | Cytoplasm | enzyme |
| CDDP 38 | CYP2C9 | Predicted interactions | cytochrome P450 family 2 subfamily C member 9 | Cytoplasm | enzyme |
| CDDP 38 | HSD17B10 | Predicted interactions | hydroxysteroid 17-beta dehydrogenase 10 | Cytoplasm | enzyme |
| CDDP 38 | ERN1 | Predicted interactions | endoplasmic reticulum to nucleus signaling 1 | Cytoplasm | kinase |
| CDDP 38 | KDM3A | Predicted interactions | lysine demethylase 3A | Nucleus | transcription regulator |
| CDDP 38 | KDM2A | Predicted interactions | lysine demethylase 2A | Nucleus | enzyme |
| CDDP 38 | KDM5C | Predicted interactions | lysine demethylase 5C | Nucleus | enzyme |
| CDDP 38 | FTO | Predicted interactions | FTO alpha-ketoglutarate dependent dioxygenase | Nucleus | enzyme |
| CDDP 38 | KDM5A | Predicted interactions | lysine demethylase 5A | Nucleus | transcription regulator |
| CDDP 38 | EGLN2 | Predicted interactions | egl-9 family hypoxia inducible factor 2 | Cytoplasm | enzyme |
| CDDP 38 | FUT7 | Predicted interactions | fucosyltransferase 7 | Cytoplasm | enzyme |
| CDDP 38 | KDM5B | Predicted interactions | lysine demethylase 5B | Nucleus | transcription regulator |
| CDDP 38 | CA13 | Predicted interactions | carbonic anhydrase 13 | Cytoplasm | enzyme |
| CDDP 38 | CA5B | Predicted interactions | carbonic anhydrase 5B | Cytoplasm | enzyme |
| CDDP 38 | PSMB5 | Predicted interactions | proteasome subunit beta 5 | Cytoplasm | peptidase |
| CDDP 38 | EGLN1 | Predicted interactions | egl-9 family hypoxia inducible factor 1 | Cytoplasm | enzyme |
| CDDP 38 | EGLN3 | Predicted interactions | egl-9 family hypoxia inducible factor 3 | Cytoplasm | enzyme |
| CDDP 38 | KDM4A | Predicted interactions | lysine demethylase 4A | Nucleus | transcription regulator |
| CDDP 38 | KDM4D | Predicted interactions | lysine demethylase 4D | Nucleus | enzyme |
| CDDP 38 | KDM4C | Predicted interactions | lysine demethylase 4C | Nucleus | enzyme |
| CDDP 39 | TDP1 | Predicted interactions | tyrosyl-DNA phosphodiesterase 1 | Nucleus | enzyme |
| CDDP 39 | NFKB1 | Predicted interactions | nuclear factor kappa B subunit 1 | Nucleus | transcription regulator |
| CDDP 39 | RECQL | Predicted interactions | RecQ like helicase | Nucleus | enzyme |
| CDDP 39 | BLM | Predicted interactions | BLM RecQ like helicase | Nucleus | enzyme |
| CDDP 39 | FYN | Predicted interactions | FYN proto-oncogene, Src family tyrosine kinase | Plasma Membrane | kinase |
| CDDP 39 | ALOX15 | Predicted interactions | arachidonate 15-lipoxygenase | Cytoplasm | enzyme |
| CDDP 39 | CA6 | Predicted interactions | carbonic anhydrase 6 | Extracellular Space | enzyme |
| CDDP 39 | NR1I2 | Predicted interactions | nuclear receptor subfamily 1 group I member 2 | Nucleus | ligand-dependent nuclear receptor |
| CDDP 39 | ALPG | Predicted interactions | alkaline phosphatase, germ cell | Plasma Membrane | phosphatase |
| CDDP 39 | CA14 | Predicted interactions | carbonic anhydrase 14 | Plasma Membrane | enzyme |
| CDDP 39 | HSD17B10 | Predicted interactions | hydroxysteroid 17-beta dehydrogenase 10 | Cytoplasm | enzyme |
| CDDP 39 | CA5A | Predicted interactions | carbonic anhydrase 5A | Cytoplasm | enzyme |
| CDDP 39 | APEX1 | Predicted interactions | apurinic/apyrimidinic endodeoxyribonuclease 1 | Nucleus | enzyme |
| CDDP 40 | CTDSP1 | Predicted interactions | CTD small phosphatase 1 | Nucleus | phosphatase |
| CDDP 40 | CISD1 | Predicted interactions | CDGSH iron sulfur domain 1 | Cytoplasm | other |
| CDDP 40 | INSR | Predicted interactions | insulin receptor | Plasma Membrane | kinase |
| CDDP 40 | PTPRS | Predicted interactions | protein tyrosine phosphatase receptor type S | Plasma Membrane | phosphatase |
| CDDP 40 | MYLK | Predicted interactions | myosin light chain kinase | Cytoplasm | kinase |
| CDDP 40 | CASP6 | Predicted interactions | caspase 6 | Cytoplasm | peptidase |
| CDDP 40 | MCL1 | Predicted interactions | MCL1 apoptosis regulator, BCL2 family member | Cytoplasm | transporter |
| CDDP 40 | SLC22A12 | Predicted interactions | solute carrier family 22 member 12 | Plasma Membrane | transporter |
| CDDP 40 | PARP1 | Predicted interactions | poly(ADP-ribose) polymerase 1 | Nucleus | enzyme |
| CDDP 40 | TTR | Predicted interactions | transthyretin | Extracellular Space | transporter |
| CDDP 40 | TNKS2 | Predicted interactions | tankyrase 2 | Nucleus | enzyme |
| CDDP 40 | TNKS | Predicted interactions | tankyrase | Nucleus | enzyme |
| CDDP 40 | L3MBTL1 | Predicted interactions | L3MBTL histone methyl-lysine binding protein 1 | Nucleus | other |
| CDDP 40 | IKBKB | Predicted interactions | inhibitor of nuclear factor kappa B kinase subunit beta | Cytoplasm | kinase |
| CDDP 40 | MGAM | Predicted interactions | maltase-glucoamylase | Plasma Membrane | enzyme |
| CDDP 40 | MIF | Predicted interactions | macrophage migration inhibitory factor | Extracellular Space | cytokine |
| CDDP 40 | PFKFB3 | Predicted interactions | 6-phosphofructo-2-kinase/fructose-2,6-biphosphatase 3 | Cytoplasm | kinase |
| CDDP 40 | NR1H3 | Predicted interactions | nuclear receptor subfamily 1 group H member 3 | Nucleus | ligand-dependent nuclear receptor |
| CDDP 40 | TERT | Predicted interactions | telomerase reverse transcriptase | Nucleus | enzyme |
| CDDP 40 | AOX1 | Predicted interactions | aldehyde oxidase 1 | Cytoplasm | enzyme |
| CDDP 40 | ST6GAL1 | Predicted interactions | ST6 beta-galactoside alpha-2,6-sialyltransferase 1 | Cytoplasm | enzyme |
| CDDP 40 | ESRRB | Predicted interactions | estrogen related receptor beta | Nucleus | ligand-dependent nuclear receptor |
| CDDP 40 | BCL2L1 | Predicted interactions | BCL2 like 1 | Cytoplasm | other |
| CDDP 40 | BCL2 | Predicted interactions | BCL2 apoptosis regulator | Cytoplasm | transporter |
| CDDP 40 | NTRK2 | Predicted interactions | neurotrophic receptor tyrosine kinase 2 | Plasma Membrane | kinase |
| CDDP 40 | ALPI | Predicted interactions | alkaline phosphatase, intestinal | Plasma Membrane | phosphatase |
| CDDP 40 | GRK6 | Predicted interactions | G protein-coupled receptor kinase 6 | Plasma Membrane | kinase |
| CDDP 40 | TBXAS1 | Predicted interactions | thromboxane A synthase 1 | Plasma Membrane | enzyme |
| CDDP 40 | ABL1 | Predicted interactions | ABL proto-oncogene 1, non-receptor tyrosine kinase | Nucleus | kinase |
| CDDP 40 | HTR2A | Predicted interactions | 5-hydroxytryptamine receptor 2A | Plasma Membrane | G-protein coupled receptor |
| CDDP 40 | ACP1 | Predicted interactions | acid phosphatase 1 | Cytoplasm | phosphatase |
| CDDP 40 | HSPA8 | Predicted interactions | heat shock protein family A (Hsp70) member 8 | Cytoplasm | enzyme |
| CDDP 40 | SLC22A6 | Predicted interactions | solute carrier family 22 member 6 | Plasma Membrane | transporter |
| CDDP 40 | GFER | Predicted interactions | growth factor, augmenter of liver regeneration | Nucleus | enzyme |
| CDDP 40 | NLRP3 | Predicted interactions | NLR family pyrin domain containing 3 | Cytoplasm | other |
| CDDP 40 | ATG4B | Predicted interactions | autophagy related 4B cysteine peptidase | Cytoplasm | peptidase |
| CDDP 40 | POLA1 | Predicted interactions | DNA polymerase alpha 1, catalytic subunit | Nucleus | enzyme |
| CDDP 40 | MPL | Predicted interactions | MPL proto-oncogene, thrombopoietin receptor | Plasma Membrane | transmembrane receptor |
| CDDP 40 | ALDH2 | Predicted interactions | aldehyde dehydrogenase 2 family member | Cytoplasm | enzyme |
| CDDP 40 | PDGFRA | Predicted interactions | platelet derived growth factor receptor alpha | Plasma Membrane | kinase |
| CDDP 40 | RAPGEF4 | Predicted interactions | Rap guanine nucleotide exchange factor 4 | Cytoplasm | other |
| CDDP 40 | PCSK7 | Predicted interactions | proprotein convertase subtilisin/kexin type 7 | Cytoplasm | peptidase |
| CDDP 40 | DYRK1A | Predicted interactions | dual specificity tyrosine phosphorylation regulated kinase 1A | Nucleus | kinase |
| CDDP 40 | FTO | Predicted interactions | FTO alpha-ketoglutarate dependent dioxygenase | Nucleus | enzyme |
| CDDP 40 | CHRNA7 | Predicted interactions | cholinergic receptor nicotinic alpha 7 subunit | Plasma Membrane | transmembrane receptor |
| CDDP 40 | IGFBP5 | Predicted interactions | insulin like growth factor binding protein 5 | Extracellular Space | other |
| CDDP 40 | MPI | Predicted interactions | mannose phosphate isomerase | Cytoplasm | enzyme |
| CDDP 40 | CSNK1A1 | Predicted interactions | casein kinase 1 alpha 1 | Cytoplasm | kinase |
| CDDP 40 | MAP3K10 | Predicted interactions | mitogen-activated protein kinase kinase kinase 10 | Cytoplasm | kinase |
| CDDP 40 | PTPN22 | Predicted interactions | protein tyrosine phosphatase non-receptor type 22 | Cytoplasm | phosphatase |
| CDDP 40 | PDE4D | Predicted interactions | phosphodiesterase 4D | Cytoplasm | enzyme |
| CDDP 40 | CES2 | Predicted interactions | carboxylesterase 2 | Cytoplasm | enzyme |
| CDDP 40 | HCK | Predicted interactions | HCK proto-oncogene, Src family tyrosine kinase | Cytoplasm | kinase |
| CDDP 40 | IKBKE | Predicted interactions | inhibitor of nuclear factor kappa B kinase subunit epsilon | Cytoplasm | kinase |
| CDDP 40 | NQO1 | Predicted interactions | NAD(P)H quinone dehydrogenase 1 | Cytoplasm | enzyme |
| CDDP 40 | TMIGD3 | Predicted interactions | transmembrane and immunoglobulin domain containing 3 | Plasma Membrane | other |
| CDDP 40 | CDC25A | Predicted interactions | cell division cycle 25A | Nucleus | phosphatase |
| CDDP 40 | PTPN11 | Predicted interactions | protein tyrosine phosphatase non-receptor type 11 | Cytoplasm | phosphatase |
| CDDP 40 | CA13 | Predicted interactions | carbonic anhydrase 13 | Cytoplasm | enzyme |
| CDDP 40 | NQO2 | Predicted interactions | N-ribosyldihydronicotinamide:quinone reductase 2 | Cytoplasm | enzyme |
| CDDP 40 | IGFBP3 | Predicted interactions | insulin like growth factor binding protein 3 | Extracellular Space | other |
| CDDP 40 | FASN | Predicted interactions | fatty acid synthase | Cytoplasm | enzyme |
| CDDP 40 | SIRT1 | Predicted interactions | sirtuin 1 | Nucleus | transcription regulator |
| CDDP 40 | IDO1 | Predicted interactions | indoleamine 2,3-dioxygenase 1 | Cytoplasm | enzyme |
| CDDP 40 | STK3 | Predicted interactions | serine/threonine kinase 3 | Cytoplasm | kinase |
| CDDP 40 | AURKA | Predicted interactions | aurora kinase A | Nucleus | kinase |
| CDDP 40 | LDHA | Predicted interactions | lactate dehydrogenase A | Cytoplasm | enzyme |
| CDDP 40 | MAP3K8 | Predicted interactions | mitogen-activated protein kinase kinase kinase 8 | Cytoplasm | kinase |
| CDDP 40 | LDHB | Predicted interactions | lactate dehydrogenase B | Cytoplasm | enzyme |
| CDDP 40 | HDAC10 | Predicted interactions | histone deacetylase 10 | Nucleus | transcription regulator |
| CDDP 40 | EPHB4 | Predicted interactions | EPH receptor B4 | Plasma Membrane | kinase |
| CDDP 40 | TAOK1 | Predicted interactions | TAO kinase 1 | Cytoplasm | kinase |
| CDDP 40 | TYRO3 | Predicted interactions | TYRO3 protein tyrosine kinase | Plasma Membrane | kinase |
| CDDP 40 | TBK1 | Predicted interactions | TANK binding kinase 1 | Cytoplasm | kinase |
| CDDP 40 | HSPA1A | Predicted interactions | Heat Shock Protein Family A (Hsp70) Member 1A | Cytoskeleton | enzyme |
| CDDP 40 | WEE1 | Predicted interactions | WEE1 G2 checkpoint kinase | Nucleus | kinase |
| CDDP 40 | DYRK1B | Predicted interactions | dual specificity tyrosine phosphorylation regulated kinase 1B | Nucleus | kinase |
| CDDP 40 | ROS1 | Predicted interactions | ROS proto-oncogene 1, receptor tyrosine kinase | Plasma Membrane | kinase |
| CDDP 40 | MARK3 | Predicted interactions | microtubule affinity regulating kinase 3 | Cytoplasm | kinase |
| CDDP 40 | LTK | Predicted interactions | leukocyte receptor tyrosine kinase | Plasma Membrane | kinase |
| CDDP 40 | GRK5 | Predicted interactions | G protein-coupled receptor kinase 5 | Plasma Membrane | kinase |
| CDDP 40 | SIK2 | Predicted interactions | salt inducible kinase 2 | Cytoplasm | kinase |
| CDDP 40 | CDK8 | Predicted interactions | cyclin dependent kinase 8 | Nucleus | kinase |
| CDDP 40 | MARK4 | Predicted interactions | microtubule affinity regulating kinase 4 | Cytoplasm | kinase |
| CDDP 40 | SGK2 | Predicted interactions | serum/glucocorticoid regulated kinase 2 | Cytoplasm | kinase |
| CDDP 40 | PRKX | Predicted interactions | protein kinase X-linked | Cytoplasm | kinase |
| CDDP 40 | NEK1 | Predicted interactions | NIMA related kinase 1 | Nucleus | kinase |
| CDDP 40 | KDM4A | Predicted interactions | lysine demethylase 4A | Nucleus | transcription regulator |
| CDDP 40 | ACVR1 | Predicted interactions | activin A receptor type 1 | Plasma Membrane | kinase |
| CDDP 40 | ALPL | Predicted interactions | alkaline phosphatase, biomineralization associated | Plasma Membrane | phosphatase |
| CDDP 40 | CDC42 | Predicted interactions | cell division cycle 42 | Cytoplasm | enzyme |
| CDDP 40 | RAC1 | Predicted interactions | Rac family small GTPase 1 | Plasma Membrane | enzyme |
| CDDP 40 | KDM4C | Predicted interactions | lysine demethylase 4C | Nucleus | enzyme |
| CDDP 02 | MAPT | Predicted interactions | microtubule associated protein tau | Plasma Membrane | other |
| CDDP 02 | PTPN6 | Predicted interactions | protein tyrosine phosphatase non-receptor type 6 | Cytoplasm | phosphatase |
| CDDP 02 | CES2 | Predicted interactions | carboxylesterase 2 | Cytoplasm | enzyme |
| CDDP 02 | PTPN11 | Predicted interactions | protein tyrosine phosphatase non-receptor type 11 | Cytoplasm | phosphatase |
| CDDP 02 | TDP1 | Predicted interactions | tyrosyl-DNA phosphodiesterase 1 | Nucleus | enzyme |
| CDDP 02 | BLM | Predicted interactions | BLM RecQ like helicase | Nucleus | enzyme |
| CDDP 02 | GMNN | Predicted interactions | geminin DNA replication inhibitor | Nucleus | transcription regulator |
| CDDP 02 | BAZ2B | Predicted interactions | bromodomain adjacent to zinc finger domain 2B | Extracellular Space | other |
| CDDP 02 | CES1 | Predicted interactions | carboxylesterase 1 | Cytoplasm | enzyme |
| CDDP 02 | POLB | Predicted interactions | DNA polymerase beta | Nucleus | enzyme |
| CDDP 02 | TERT | Predicted interactions | telomerase reverse transcriptase | Nucleus | enzyme |
| CDDP 02 | TP53 | Predicted interactions | tumor protein p53 | Nucleus | transcription regulator |
| CDDP 02 | ABCB1 | Predicted interactions | ATP binding cassette subfamily B member 1 | Plasma Membrane | transporter |
| CDDP 02 | BRCA1 | Predicted interactions | BRCA1 DNA repair associated | Nucleus | transcription regulator |
| CDDP 03 | CES1 | Predicted interactions | carboxylesterase 1 | Cytoplasm | enzyme |
| CDDP 03 | CES2 | Predicted interactions | carboxylesterase 2 | Cytoplasm | enzyme |
| CDDP 03 | STAT3 | Predicted interactions | signal transducer and activator of transcription 3 | Nucleus | transcription regulator |
| CDDP 03 | MAPT | Predicted interactions | microtubule associated protein tau | Plasma Membrane | other |
| CDDP 03 | ACHE | Predicted interactions | acetylcholinesterase (Cartwright blood group) | Plasma Membrane | enzyme |
| CDDP 03 | TERT | Predicted interactions | telomerase reverse transcriptase | Nucleus | enzyme |
| CDDP 03 | PTPN6 | Predicted interactions | protein tyrosine phosphatase non-receptor type 6 | Cytoplasm | phosphatase |
| CDDP 03 | PTPN1 | Predicted interactions | protein tyrosine phosphatase non-receptor type 1 | Cytoplasm | phosphatase |
| CDDP 03 | PTPN11 | Predicted interactions | protein tyrosine phosphatase non-receptor type 11 | Cytoplasm | phosphatase |
| CDDP 04 | APAF1 | Predicted interactions | apoptotic peptidase activating factor 1 | Cytoplasm | other |
| CDDP 04 | AKR1B1 | Predicted interactions | aldo-keto reductase family 1 member B | Cytoplasm | enzyme |
| CDDP 04 | CES1 | Predicted interactions | carboxylesterase 1 | Cytoplasm | enzyme |
| CDDP 04 | TERT | Predicted interactions | telomerase reverse transcriptase | Nucleus | enzyme |
| CDDP 04 | PTPN6 | Predicted interactions | protein tyrosine phosphatase non-receptor type 6 | Cytoplasm | phosphatase |
| CDDP 04 | CES2 | Predicted interactions | carboxylesterase 2 | Cytoplasm | enzyme |
| CDDP 04 | PTPN11 | Predicted interactions | protein tyrosine phosphatase non-receptor type 11 | Cytoplasm | phosphatase |
| CDDP 04 | ALDH1A1 | Predicted interactions | aldehyde dehydrogenase 1 family member A1 | Cytoplasm | enzyme |
| CDDP 04 | HSD17B10 | Predicted interactions | hydroxysteroid 17-beta dehydrogenase 10 | Cytoplasm | enzyme |
| CDDP 05 | TDP1 | Predicted interactions | tyrosyl-DNA phosphodiesterase 1 | Nucleus | enzyme |
| CDDP 05 | PTPN11 | Predicted interactions | protein tyrosine phosphatase non-receptor type 11 | Cytoplasm | phosphatase |
| CDDP 05 | PTPN6 | Predicted interactions | protein tyrosine phosphatase non-receptor type 6 | Cytoplasm | phosphatase |
| CDDP 05 | CES1 | Predicted interactions | carboxylesterase 1 | Cytoplasm | enzyme |
| CDDP 05 | CES2 | Predicted interactions | carboxylesterase 2 | Cytoplasm | enzyme |
| CDDP 05 | ACHE | Predicted interactions | acetylcholinesterase (Cartwright blood group) | Plasma Membrane | enzyme |
| CDDP 05 | MAPK1 | Predicted interactions | mitogen-activated protein kinase 1 | Cytoplasm | kinase |
| CDDP 05 | MAPT | Predicted interactions | microtubule associated protein tau | Plasma Membrane | other |
| CDDP 05 | KDM4E | Predicted interactions | lysine demethylase 4E | Nucleus | enzyme |
| CDDP 06 | CA7 | Predicted interactions | carbonic anhydrase 7 | Cytoplasm | enzyme |
| CDDP 06 | CA12 | Predicted interactions | carbonic anhydrase 12 | Plasma Membrane | enzyme |
| CDDP 06 | CA4 | Predicted interactions | carbonic anhydrase 4 | Plasma Membrane | enzyme |
| CDDP 06 | CA2 | Predicted interactions | carbonic anhydrase 2 | Cytoplasm | enzyme |
| CDDP 11 | KDM4E | Predicted interactions | lysine demethylase 4E | Nucleus | enzyme |
| CDDP 11 | KDM4A | Predicted interactions | lysine demethylase 4A | Nucleus | transcription regulator |
| CDDP 12 | AKR1B1 | Predicted interactions | aldo-keto reductase family 1 member B | Cytoplasm | enzyme |
| CDDP 12 | TTR | Predicted interactions | transthyretin | Extracellular Space | transporter |
| CDDP 12 | APEX1 | Predicted interactions | apurinic/apyrimidinic endodeoxyribonuclease 1 | Nucleus | enzyme |
| CDDP 12 | TDP1 | Predicted interactions | tyrosyl-DNA phosphodiesterase 1 | Nucleus | enzyme |
| CDDP 12 | PKM | Predicted interactions | pyruvate kinase M1/2 | Cytoplasm | kinase |
| CDDP 12 | KDM4E | Predicted interactions | lysine demethylase 4E | Nucleus | enzyme |
| CDDP 12 | RECQL | Predicted interactions | RecQ like helicase | Nucleus | enzyme |
| CDDP 12 | POLB | Predicted interactions | DNA polymerase beta | Nucleus | enzyme |
| CDDP 12 | GAA | Predicted interactions | glucosidase alpha, acid | Cytoplasm | enzyme |
| CDDP 12 | MMP1 | Predicted interactions | matrix metallopeptidase 1 | Extracellular Space | peptidase |
| CDDP 12 | MCL1 | Predicted interactions | MCL1 apoptosis regulator, BCL2 family member | Cytoplasm | transporter |
| CDDP 12 | FYN | Predicted interactions | FYN proto-oncogene, Src family tyrosine kinase | Plasma Membrane | kinase |
| CDDP 13 | CA7 | Predicted interactions | carbonic anhydrase 7 | Cytoplasm | enzyme |
| CDDP 13 | CA12 | Predicted interactions | carbonic anhydrase 12 | Plasma Membrane | enzyme |
| CDDP 13 | CA4 | Predicted interactions | carbonic anhydrase 4 | Plasma Membrane | enzyme |
| CDDP 13 | CA2 | Predicted interactions | carbonic anhydrase 2 | Cytoplasm | enzyme |
| CDDP 22 | TDP1 | Predicted interactions | tyrosyl-DNA phosphodiesterase 1 | Nucleus | enzyme |
| CDDP 22 | KDM4E | Predicted interactions | lysine demethylase 4E | Nucleus | enzyme |
| CDDP 22 | CA2 | Predicted interactions | carbonic anhydrase 2 | Cytoplasm | enzyme |
| CDDP 22 | CA7 | Predicted interactions | carbonic anhydrase 7 | Cytoplasm | enzyme |
| CDDP 22 | CA1 | Predicted interactions | carbonic anhydrase 1 | Cytoplasm | enzyme |
| CDDP 22 | CA3 | Predicted interactions | carbonic anhydrase 3 | Cytoplasm | enzyme |
| CDDP 22 | CA6 | Predicted interactions | carbonic anhydrase 6 | Extracellular Space | enzyme |
| CDDP 22 | CA12 | Predicted interactions | carbonic anhydrase 12 | Plasma Membrane | enzyme |
| CDDP 22 | NFKB1 | Predicted interactions | nuclear factor kappa B subunit 1 | Nucleus | transcription regulator |
| CDDP 22 | CA14 | Predicted interactions | carbonic anhydrase 14 | Plasma Membrane | enzyme |
| CDDP 22 | CA9 | Predicted interactions | carbonic anhydrase 9 | Nucleus | enzyme |
| CDDP 22 | CA4 | Predicted interactions | carbonic anhydrase 4 | Plasma Membrane | enzyme |
| CDDP 22 | CA5B | Predicted interactions | carbonic anhydrase 5B | Cytoplasm | enzyme |
| CDDP 22 | HSD17B10 | Predicted interactions | hydroxysteroid 17-beta dehydrogenase 10 | Cytoplasm | enzyme |
| CDDP 22 | CA5A | Predicted interactions | carbonic anhydrase 5A | Cytoplasm | enzyme |
| CDDP 22 | MMP9 | Predicted interactions | matrix metallopeptidase 9 | Extracellular Space | peptidase |
| CDDP 22 | MMP1 | Predicted interactions | matrix metallopeptidase 1 | Extracellular Space | peptidase |
| CDDP 22 | MMP2 | Predicted interactions | matrix metallopeptidase 2 | Extracellular Space | peptidase |
| CDDP 22 | PTPN1 | Predicted interactions | protein tyrosine phosphatase non-receptor type 1 | Cytoplasm | phosphatase |
| CDDP 22 | EGFR | Predicted interactions | epidermal growth factor receptor | Plasma Membrane | kinase |
| CDDP 22 | AKR1B1 | Predicted interactions | aldo-keto reductase family 1 member B | Cytoplasm | enzyme |
| CDDP 22 | XDH | Predicted interactions | xanthine dehydrogenase | Cytoplasm | enzyme |
| CDDP 22 | APEX1 | Predicted interactions | apurinic/apyrimidinic endodeoxyribonuclease 1 | Nucleus | enzyme |
| CDDP 35 | CES1 | Predicted interactions | carboxylesterase 1 | Cytoplasm | enzyme |
| CDDP 35 | CES2 | Predicted interactions | carboxylesterase 2 | Cytoplasm | enzyme |
| CDDP 35 | PTPN1 | Predicted interactions | protein tyrosine phosphatase non-receptor type 1 | Cytoplasm | phosphatase |
| CDDP 35 | CDC25A | Predicted interactions | cell division cycle 25A | Nucleus | phosphatase |
| CDDP 35 | CDC25B | Predicted interactions | cell division cycle 25B | Nucleus | phosphatase |
| CDDP 36 | CA2 | Predicted interactions | carbonic anhydrase 2 | Cytoplasm | enzyme |
| CDDP 36 | CA7 | Predicted interactions | carbonic anhydrase 7 | Cytoplasm | enzyme |
| CDDP 36 | CA1 | Predicted interactions | carbonic anhydrase 1 | Cytoplasm | enzyme |
| CDDP 36 | CA6 | Predicted interactions | carbonic anhydrase 6 | Extracellular Space | enzyme |
| CDDP 36 | CA12 | Predicted interactions | carbonic anhydrase 12 | Plasma Membrane | enzyme |
| CDDP 36 | CA14 | Predicted interactions | carbonic anhydrase 14 | Plasma Membrane | enzyme |
| CDDP 36 | CA9 | Predicted interactions | carbonic anhydrase 9 | Nucleus | enzyme |
| CDDP 36 | CA4 | Predicted interactions | carbonic anhydrase 4 | Plasma Membrane | enzyme |
| CDDP 36 | KDM4E | Predicted interactions | lysine demethylase 4E | Nucleus | enzyme |
| CDDP 36 | MMP9 | Predicted interactions | matrix metallopeptidase 9 | Extracellular Space | peptidase |
| CDDP 36 | MMP1 | Predicted interactions | matrix metallopeptidase 1 | Extracellular Space | peptidase |
| CDDP 36 | MMP2 | Predicted interactions | matrix metallopeptidase 2 | Extracellular Space | peptidase |
| CDDP 36 | PSMB5 | Predicted interactions | proteasome subunit beta 5 | Cytoplasm | peptidase |
| CDDP 36 | EGLN1 | Predicted interactions | egl-9 family hypoxia inducible factor 1 | Cytoplasm | enzyme |
| CDDP 37 | THPO | Predicted interactions | thrombopoietin | Extracellular Space | cytokine |
| CDDP 37 | CA2 | Predicted interactions | carbonic anhydrase 2 | Cytoplasm | enzyme |
| CDDP 37 | CA12 | Predicted interactions | carbonic anhydrase 12 | Plasma Membrane | enzyme |
| CDDP 37 | NFKB1 | Predicted interactions | nuclear factor kappa B subunit 1 | Nucleus | transcription regulator |
| CDDP 37 | CA5B | Predicted interactions | carbonic anhydrase 5B | Cytoplasm | enzyme |
| CDDP 37 | HSD17B10 | Predicted interactions | hydroxysteroid 17-beta dehydrogenase 10 | Cytoplasm | enzyme |
| CDDP 37 | MTOR | Predicted interactions | mechanistic target of rapamycin kinase | Nucleus | kinase |
| CDDP 37 | CYP3A4 | Predicted interactions | cytochrome P450 family 3 subfamily A member 4 | Cytoplasm | enzyme |
| CDDP 37 | FYN | Predicted interactions | FYN proto-oncogene, Src family tyrosine kinase | Plasma Membrane | kinase |
| CDDP 37 | CA7 | Predicted interactions | carbonic anhydrase 7 | Cytoplasm | enzyme |
| CDDP 37 | CA1 | Predicted interactions | carbonic anhydrase 1 | Cytoplasm | enzyme |
| CDDP 37 | CA3 | Predicted interactions | carbonic anhydrase 3 | Cytoplasm | enzyme |
| CDDP 37 | CA6 | Predicted interactions | carbonic anhydrase 6 | Extracellular Space | enzyme |
| CDDP 37 | CA14 | Predicted interactions | carbonic anhydrase 14 | Plasma Membrane | enzyme |
| CDDP 37 | CA9 | Predicted interactions | carbonic anhydrase 9 | Nucleus | enzyme |
| CDDP 37 | CA4 | Predicted interactions | carbonic anhydrase 4 | Plasma Membrane | enzyme |
| CDDP 37 | CA5A | Predicted interactions | carbonic anhydrase 5A | Cytoplasm | enzyme |
| CDDP 38 | TPMT | Predicted interactions | thiopurine S-methyltransferase | Cytoplasm | enzyme |
| CDDP 39 | TPMT | Predicted interactions | thiopurine S-methyltransferase | Cytoplasm | enzyme |
| CDDP 40 | TPMT | Predicted interactions | thiopurine S-methyltransferase | Cytoplasm | enzyme |
| CDDP 36 | FUT7 | Predicted interactions | fucosyltransferase 7 | Cytoplasm | enzyme |
| CDDP 02 | KMT2A | Predicted interactions | lysine methyltransferase 2A | Nucleus | transcription regulator |
| CDDP 02 | SENP8 | Predicted interactions | SUMO peptidase family member, NEDD8 specific | Cytoplasm | peptidase |
| CDDP 02 | SENP7 | Predicted interactions | SUMO specific peptidase 7 | Nucleus | peptidase |
| CDDP 02 | SENP6 | Predicted interactions | SUMO specific peptidase 6 | Cytoplasm | peptidase |
| CDDP 02 | APAF1 | Predicted interactions | apoptotic peptidase activating factor 1 | Cytoplasm | other |
| CDDP 02 | THRB | Predicted interactions | thyroid hormone receptor beta | Nucleus | ligand-dependent nuclear receptor |
| CDDP 02 | ATM | Predicted interactions | ATM serine/threonine kinase | Nucleus | kinase |
| CDDP 02 | PKM | Predicted interactions | pyruvate kinase M1/2 | Cytoplasm | kinase |
| CDDP 02 | GPR55 | Predicted interactions | G protein-coupled receptor 55 | Plasma Membrane | G-protein coupled receptor |
| CDDP 02 | NOD1 | Predicted interactions | nucleotide binding oligomerization domain containing 1 | Cytoplasm | other |
| CDDP 02 | USP2 | Predicted interactions | ubiquitin specific peptidase 2 | Cytoplasm | peptidase |
| CDDP 02 | PLEC | Predicted interactions | plectin | Cytoplasm | other |
| CDDP 02 | GALK1 | Predicted interactions | galactokinase 1 | Cytoplasm | kinase |
| CDDP 02 | SMAD3 | Predicted interactions | SMAD family member 3 | Nucleus | transcription regulator |
| CDDP 02 | NOD2 | Predicted interactions | nucleotide binding oligomerization domain containing 2 | Cytoplasm | other |
| CDDP 02 | GPR35 | Predicted interactions | G protein-coupled receptor 35 | Plasma Membrane | G-protein coupled receptor |
| CDDP 02 | EIF4H | Predicted interactions | eukaryotic translation initiation factor 4H | Cytoplasm | translation regulator |
| CDDP 02 | NPC1 | Predicted interactions | NPC intracellular cholesterol transporter 1 | Cytoplasm | transporter |
| CDDP 02 | PABPC1 | Predicted interactions | poly(A) binding protein cytoplasmic 1 | Cytoplasm | translation regulator |
| CDDP 02 | RAB9A | Predicted interactions | RAB9A, member RAS oncogene family | Cytoplasm | enzyme |
| CDDP 02 | HKDC1 | Predicted interactions | hexokinase domain containing 1 | Cytoplasm | kinase |
| CDDP 02 | NLRP3 | Predicted interactions | NLR family pyrin domain containing 3 | Cytoplasm | other |
| CDDP 02 | ATG4B | Predicted interactions | autophagy related 4B cysteine peptidase | Cytoplasm | peptidase |
| CDDP 02 | CTDSP1 | Predicted interactions | CTD small phosphatase 1 | Nucleus | phosphatase |
| CDDP 02 | TSHR | Predicted interactions | thyroid stimulating hormone receptor | Plasma Membrane | G-protein coupled receptor |
| CDDP 02 | DNMT1 | Predicted interactions | DNA methyltransferase 1 | Nucleus | enzyme |
| CDDP 02 | CASP3 | Predicted interactions | caspase 3 | Cytoplasm | peptidase |
| CDDP 02 | GLA | Predicted interactions | galactosidase alpha | Cytoplasm | enzyme |
| CDDP 02 | GAA | Predicted interactions | glucosidase alpha, acid | Cytoplasm | enzyme |
| CDDP 02 | GSK3B | Predicted interactions | glycogen synthase kinase 3 beta | Nucleus | kinase |
| CDDP 02 | DUSP3 | Predicted interactions | dual specificity phosphatase 3 | Cytoplasm | phosphatase |
| CDDP 02 | F12 | Predicted interactions | coagulation factor XII | Extracellular Space | peptidase |
| CDDP 02 | GSK3A | Predicted interactions | glycogen synthase kinase 3 alpha | Nucleus | kinase |
| CDDP 02 | AHR | Predicted interactions | aryl hydrocarbon receptor | Nucleus | ligand-dependent nuclear receptor |
| CDDP 02 | PLA2G7 | Predicted interactions | phospholipase A2 group VII | Extracellular Space | enzyme |
| CDDP 02 | CDK5 | Predicted interactions | cyclin dependent kinase 5 | Nucleus | kinase |
| CDDP 02 | MAPK1 | Predicted interactions | mitogen-activated protein kinase 1 | Cytoplasm | kinase |
| CDDP 02 | NTSR1 | Predicted interactions | neurotensin receptor 1 | Plasma Membrane | G-protein coupled receptor |
| CDDP 02 | MCL1 | Predicted interactions | MCL1 apoptosis regulator, BCL2 family member | Cytoplasm | transporter |
| CDDP 02 | NR2E3 | Predicted interactions | nuclear receptor subfamily 2 group E member 3 | Nucleus | ligand-dependent nuclear receptor |
| CDDP 02 | NPSR1 | Predicted interactions | neuropeptide S receptor 1 | Plasma Membrane | G-protein coupled receptor |
| CDDP 02 | MAP4K2 | Predicted interactions | mitogen-activated protein kinase kinase kinase kinase 2 | Cytoplasm | kinase |
| CDDP 02 | HTT | Predicted interactions | huntingtin | Cytoplasm | transcription regulator |
| CDDP 02 | ALPI | Predicted interactions | alkaline phosphatase, intestinal | Plasma Membrane | phosphatase |
| CDDP 02 | TLR9 | Predicted interactions | toll like receptor 9 | Plasma Membrane | transmembrane receptor |
| CDDP 04 | ALOX15 | Predicted interactions | arachidonate 15-lipoxygenase | Cytoplasm | enzyme |
| CDDP 04 | CYP3A4 | Predicted interactions | cytochrome P450 family 3 subfamily A member 4 | Cytoplasm | enzyme |
| CDDP 04 | SENP8 | Predicted interactions | SUMO peptidase family member, NEDD8 specific | Cytoplasm | peptidase |
| CDDP 04 | SENP6 | Predicted interactions | SUMO specific peptidase 6 | Cytoplasm | peptidase |
| CDDP 05 | MITF | Predicted interactions | melanocyte inducing transcription factor | Nucleus | transcription regulator |
| CDDP 05 | GLA | Predicted interactions | galactosidase alpha | Cytoplasm | enzyme |
| CDDP 11 | TDP1 | Predicted interactions | tyrosyl-DNA phosphodiesterase 1 | Nucleus | enzyme |
| CDDP 11 | BLM | Predicted interactions | BLM RecQ like helicase | Nucleus | enzyme |
| CDDP 11 | PTPN22 | Predicted interactions | protein tyrosine phosphatase non-receptor type 22 | Cytoplasm | phosphatase |
| CDDP 11 | NFKB1 | Predicted interactions | nuclear factor kappa B subunit 1 | Nucleus | transcription regulator |
| CDDP 11 | CASP6 | Predicted interactions | caspase 6 | Cytoplasm | peptidase |
| CDDP 11 | ERAP1 | Predicted interactions | endoplasmic reticulum aminopeptidase 1 | Extracellular Space | peptidase |
| CDDP 20 | NFKB1 | Predicted interactions | nuclear factor kappa B subunit 1 | Nucleus | transcription regulator |
| CDDP 22 | HIF1A | Predicted interactions | hypoxia inducible factor 1 subunit alpha | Nucleus | transcription regulator |
| CDDP 22 | BLM | Predicted interactions | BLM RecQ like helicase | Nucleus | enzyme |
| CDDP 22 | SMAD3 | Predicted interactions | SMAD family member 3 | Nucleus | transcription regulator |
| CDDP 22 | GPR35 | Predicted interactions | G protein-coupled receptor 35 | Plasma Membrane | G-protein coupled receptor |
| CDDP 22 | ATG4B | Predicted interactions | autophagy related 4B cysteine peptidase | Cytoplasm | peptidase |
| CDDP 22 | PTPN22 | Predicted interactions | protein tyrosine phosphatase non-receptor type 22 | Cytoplasm | phosphatase |
| CDDP 22 | ALOX15 | Predicted interactions | arachidonate 15-lipoxygenase | Cytoplasm | enzyme |
| CDDP 22 | ERAP1 | Predicted interactions | endoplasmic reticulum aminopeptidase 1 | Extracellular Space | peptidase |
| CDDP 22 | SNCA | Predicted interactions | synuclein alpha | Cytoplasm | enzyme |
| CDDP 36 | TDP1 | Predicted interactions | tyrosyl-DNA phosphodiesterase 1 | Nucleus | enzyme |
| CDDP 36 | NFKB1 | Predicted interactions | nuclear factor kappa B subunit 1 | Nucleus | transcription regulator |
| CDDP 36 | HIF1A | Predicted interactions | hypoxia inducible factor 1 subunit alpha | Nucleus | transcription regulator |
| CDDP 36 | BLM | Predicted interactions | BLM RecQ like helicase | Nucleus | enzyme |
| CDDP 36 | GPR35 | Predicted interactions | G protein-coupled receptor 35 | Plasma Membrane | G-protein coupled receptor |
| CDDP 36 | CASP6 | Predicted interactions | caspase 6 | Cytoplasm | peptidase |
| CDDP 36 | ERAP1 | Predicted interactions | endoplasmic reticulum aminopeptidase 1 | Extracellular Space | peptidase |
| CDDP 37 | GLA | Predicted interactions | galactosidase alpha | Cytoplasm | enzyme |
| CDDP 38 | HPGD | Predicted interactions | 15-hydroxyprostaglandin dehydrogenase | Cytoplasm | enzyme |
| CDDP 38 | NPSR1 | Predicted interactions | neuropeptide S receptor 1 | Plasma Membrane | G-protein coupled receptor |
| CDDP 38 | TDP1 | Predicted interactions | tyrosyl-DNA phosphodiesterase 1 | Nucleus | enzyme |
| CDDP 38 | BLM | Predicted interactions | BLM RecQ like helicase | Nucleus | enzyme |
| CDDP 38 | NFKB1 | Predicted interactions | nuclear factor kappa B subunit 1 | Nucleus | transcription regulator |
| CDDP 38 | ERAP1 | Predicted interactions | endoplasmic reticulum aminopeptidase 1 | Extracellular Space | peptidase |
| CDDP 40 | CA2 | Predicted interactions | carbonic anhydrase 2 | Cytoplasm | enzyme |
| CDDP 40 | CA7 | Predicted interactions | carbonic anhydrase 7 | Cytoplasm | enzyme |
| CDDP 40 | CA1 | Predicted interactions | carbonic anhydrase 1 | Cytoplasm | enzyme |
| CDDP 40 | CA3 | Predicted interactions | carbonic anhydrase 3 | Cytoplasm | enzyme |
| CDDP 40 | CA6 | Predicted interactions | carbonic anhydrase 6 | Extracellular Space | enzyme |
| CDDP 40 | CA12 | Predicted interactions | carbonic anhydrase 12 | Plasma Membrane | enzyme |
| CDDP 40 | CA14 | Predicted interactions | carbonic anhydrase 14 | Plasma Membrane | enzyme |
| CDDP 40 | CA9 | Predicted interactions | carbonic anhydrase 9 | Nucleus | enzyme |
| CDDP 40 | CA4 | Predicted interactions | carbonic anhydrase 4 | Plasma Membrane | enzyme |
| CDDP 40 | CA5A | Predicted interactions | carbonic anhydrase 5A | Cytoplasm | enzyme |
| CDDP 40 | HPGD | Predicted interactions | 15-hydroxyprostaglandin dehydrogenase | Cytoplasm | enzyme |
| CDDP 40 | ALOX15 | Predicted interactions | arachidonate 15-lipoxygenase | Cytoplasm | enzyme |
| CDDP 40 | CYP1A2 | Predicted interactions | cytochrome P450 family 1 subfamily A member 2 | Cytoplasm | enzyme |
| CDDP 40 | CYP2C9 | Predicted interactions | cytochrome P450 family 2 subfamily C member 9 | Cytoplasm | enzyme |
| CDDP 40 | NPSR1 | Predicted interactions | neuropeptide S receptor 1 | Plasma Membrane | G-protein coupled receptor |
| CDDP 40 | TDP1 | Predicted interactions | tyrosyl-DNA phosphodiesterase 1 | Nucleus | enzyme |
| CDDP 40 | BLM | Predicted interactions | BLM RecQ like helicase | Nucleus | enzyme |
| CDDP 40 | NFKB1 | Predicted interactions | nuclear factor kappa B subunit 1 | Nucleus | transcription regulator |
| CDDP 40 | CA5B | Predicted interactions | carbonic anhydrase 5B | Cytoplasm | enzyme |

**Table S3. Predicted kinase targets of 40 important components by KinomeX and their relationships**

| **Molecule ID** | **Gene Symbol** | **Entrez Gene Name** | **Location** | **Type(s)** |
| --- | --- | --- | --- | --- |
| CDDP 01 | ACVR1 | activin A receptor type 1 | Plasma Membrane | kinase |
| CDDP 01 | CDK15 | cyclin dependent kinase 15 | Plasma Membrane | kinase |
| CDDP 01 | AURKA | aurora kinase A | Nucleus | kinase |
| CDDP 01 | AURKB | aurora kinase B | Nucleus | kinase |
| CDDP 01 | AURKC | aurora kinase C | Nucleus | kinase |
| CDDP 01 | AXL | AXL receptor tyrosine kinase | Plasma Membrane | kinase |
| CDDP 01 | BMPR1A | bone morphogenetic protein receptor type 1A | Plasma Membrane | kinase |
| CDDP 01 | BTK | Bruton tyrosine kinase | Cytoplasm | kinase |
| CDDP 01 | CAMKK2 | calcium/calmodulin dependent protein kinase kinase 2 | Cytoplasm | kinase |
| CDDP 01 | CDC7 | cell division cycle 7 | Nucleus | kinase |
| CDDP 01 | CDK5 | cyclin dependent kinase 5 | Nucleus | kinase |
| CDDP 01 | CDK9 | cyclin dependent kinase 9 | Nucleus | kinase |
| CDDP 01 | CDKL2 | cyclin dependent kinase like 2 | Nucleus | kinase |
| CDDP 01 | CDKL3 | cyclin dependent kinase like 3 | Cytoplasm | kinase |
| CDDP 01 | DAPK3 | death associated protein kinase 3 | Cytoplasm | kinase |
| CDDP 01 | DCLK2 | doublecortin like kinase 2 | Cytoplasm | kinase |
| CDDP 01 | DYRK1B | dual specificity tyrosine phosphorylation regulated kinase 1B | Nucleus | kinase |
| CDDP 01 | EPHA4 | EPH receptor A4 | Plasma Membrane | kinase |
| CDDP 01 | EPHB1 | EPH receptor B1 | Plasma Membrane | kinase |
| CDDP 01 | EPHB6 | EPH receptor B6 | Plasma Membrane | kinase |
| CDDP 01 | MAPK12 | mitogen-activated protein kinase 12 | Cytoplasm | kinase |
| CDDP 01 | MAPK7 | mitogen-activated protein kinase 7 | Cytoplasm | kinase |
| CDDP 01 | FER | FER tyrosine kinase | Cytoplasm | kinase |
| CDDP 01 | FES | FES proto-oncogene, tyrosine kinase | Cytoplasm | kinase |
| CDDP 01 | GRK1 | G protein-coupled receptor kinase 1 | Plasma Membrane | kinase |
| CDDP 01 | GRK5 | G protein-coupled receptor kinase 5 | Plasma Membrane | kinase |
| CDDP 01 | HCK | HCK proto-oncogene, Src family tyrosine kinase | Cytoplasm | kinase |
| CDDP 01 | HIPK3 | homeodomain interacting protein kinase 3 | Nucleus | kinase |
| CDDP 01 | EIF2AK1 | eukaryotic translation initiation factor 2 alpha kinase 1 | Cytoplasm | kinase |
| CDDP 01 | IGF1R | insulin like growth factor 1 receptor | Plasma Membrane | transmembrane receptor |
| CDDP 01 | INSR | insulin receptor | Plasma Membrane | kinase |
| CDDP 01 | IRAK1 | interleukin 1 receptor associated kinase 1 | Plasma Membrane | kinase |
| CDDP 01 | IRAK4 | interleukin 1 receptor associated kinase 4 | Cytoplasm | kinase |
| CDDP 01 | LIMK1 | LIM domain kinase 1 | Cytoplasm | kinase |
| CDDP 01 | LTK | leukocyte receptor tyrosine kinase | Plasma Membrane | kinase |
| CDDP 01 | MAP3K10 | mitogen-activated protein kinase kinase kinase 10 | Cytoplasm | kinase |
| CDDP 01 | MARK4 | microtubule affinity regulating kinase 4 | Cytoplasm | kinase |
| CDDP 01 | MELK | maternal embryonic leucine zipper kinase | Cytoplasm | kinase |
| CDDP 01 | MAP2K7 | mitogen-activated protein kinase kinase 7 | Cytoplasm | kinase |
| CDDP 01 | MKNK1 | MAPK interacting serine/threonine kinase 1 | Cytoplasm | kinase |
| CDDP 01 | NLK | nemo like kinase | Nucleus | kinase |
| CDDP 01 | NTRK3 | neurotrophic receptor tyrosine kinase 3 | Plasma Membrane | kinase |
| CDDP 01 | PASK | PAS domain containing serine/threonine kinase | Cytoplasm | kinase |
| CDDP 01 | PLK3 | polo like kinase 3 | Nucleus | kinase |
| CDDP 01 | PLK4 | polo like kinase 4 | Cytoplasm | kinase |
| CDDP 01 | RIPK1 | receptor interacting serine/threonine kinase 1 | Plasma Membrane | kinase |
| CDDP 01 | ROS1 | ROS proto-oncogene 1, receptor tyrosine kinase | Plasma Membrane | kinase |
| CDDP 01 | SIK2 | salt inducible kinase 2 | Cytoplasm | kinase |
| CDDP 01 | STK36 | serine/threonine kinase 36 | Cytoplasm | kinase |
| CDDP 01 | SYK | spleen associated tyrosine kinase | Cytoplasm | kinase |
| CDDP 01 | TAOK1 | TAO kinase 1 | Cytoplasm | kinase |
| CDDP 01 | TGFBR1 | transforming growth factor beta receptor 1 | Plasma Membrane | kinase |
| CDDP 01 | TNK2 | tyrosine kinase non receptor 2 | Cytoplasm | kinase |
| CDDP 01 | TXK | TXK tyrosine kinase | Cytoplasm | kinase |
| CDDP 01 | TYK2 | tyrosine kinase 2 | Plasma Membrane | kinase |
| CDDP 02 | ACVRL1 | activin A receptor like type 1 | Plasma Membrane | kinase |
| CDDP 02 | ACVR1 | activin A receptor type 1 | Plasma Membrane | kinase |
| CDDP 02 | CDK15 | cyclin dependent kinase 15 | Plasma Membrane | kinase |
| CDDP 02 | CDKL2 | cyclin dependent kinase like 2 | Nucleus | kinase |
| CDDP 02 | CDKL3 | cyclin dependent kinase like 3 | Cytoplasm | kinase |
| CDDP 02 | EPHA8 | EPH receptor A8 | Plasma Membrane | kinase |
| CDDP 02 | EPHB6 | EPH receptor B6 | Plasma Membrane | kinase |
| CDDP 02 | GAK | cyclin G associated kinase | Nucleus | kinase |
| CDDP 02 | HIPK3 | homeodomain interacting protein kinase 3 | Nucleus | kinase |
| CDDP 02 | EIF2AK1 | eukaryotic translation initiation factor 2 alpha kinase 1 | Cytoplasm | kinase |
| CDDP 02 | LIMK1 | LIM domain kinase 1 | Cytoplasm | kinase |
| CDDP 02 | NLK | nemo like kinase | Nucleus | kinase |
| CDDP 02 | RIPK2 | receptor interacting serine/threonine kinase 2 | Plasma Membrane | kinase |
| CDDP 02 | MAPK14 | mitogen-activated protein kinase 14 | Cytoplasm | kinase |
| CDDP 03 | ACVRL1 | activin A receptor like type 1 | Plasma Membrane | kinase |
| CDDP 03 | CDK15 | cyclin dependent kinase 15 | Plasma Membrane | kinase |
| CDDP 03 | BRAF | B-Raf proto-oncogene, serine/threonine kinase | Cytoplasm | kinase |
| CDDP 03 | CDKL2 | cyclin dependent kinase like 2 | Nucleus | kinase |
| CDDP 03 | CDKL3 | cyclin dependent kinase like 3 | Cytoplasm | kinase |
| CDDP 03 | CSNK2A2 | casein kinase 2 alpha 2 | Cytoplasm | kinase |
| CDDP 03 | EPHB6 | EPH receptor B6 | Plasma Membrane | kinase |
| CDDP 03 | FRK | fyn related Src family tyrosine kinase | Nucleus | kinase |
| CDDP 03 | GAK | cyclin G associated kinase | Nucleus | kinase |
| CDDP 03 | HCK | HCK proto-oncogene, Src family tyrosine kinase | Cytoplasm | kinase |
| CDDP 03 | HIPK3 | homeodomain interacting protein kinase 3 | Nucleus | kinase |
| CDDP 03 | EIF2AK1 | eukaryotic translation initiation factor 2 alpha kinase 1 | Cytoplasm | kinase |
| CDDP 03 | MAPK10 | mitogen-activated protein kinase 10 | Cytoplasm | kinase |
| CDDP 03 | MKNK1 | MAPK interacting serine/threonine kinase 1 | Cytoplasm | kinase |
| CDDP 03 | PKMYT1 | protein kinase, membrane associated tyrosine/threonine 1 | Cytoplasm | kinase |
| CDDP 03 | RIPK2 | receptor interacting serine/threonine kinase 2 | Plasma Membrane | kinase |
| CDDP 03 | SIK2 | salt inducible kinase 2 | Cytoplasm | kinase |
| CDDP 03 | SRC | SRC proto-oncogene, non-receptor tyrosine kinase | Cytoplasm | kinase |
| CDDP 03 | TGFBR1 | transforming growth factor beta receptor 1 | Plasma Membrane | kinase |
| CDDP 03 | MAPK14 | mitogen-activated protein kinase 14 | Cytoplasm | kinase |
| CDDP 03 | MAPK1 | mitogen-activated protein kinase 1 | Cytoplasm | kinase |
| CDDP 04 | ACVRL1 | activin A receptor like type 1 | Plasma Membrane | kinase |
| CDDP 04 | ACVR1B | activin A receptor type 1B | Plasma Membrane | kinase |
| CDDP 04 | CDK15 | cyclin dependent kinase 15 | Plasma Membrane | kinase |
| CDDP 04 | CDC7 | cell division cycle 7 | Nucleus | kinase |
| CDDP 04 | CDKL2 | cyclin dependent kinase like 2 | Nucleus | kinase |
| CDDP 04 | CDKL3 | cyclin dependent kinase like 3 | Cytoplasm | kinase |
| CDDP 04 | CSK | C-terminal Src kinase | Cytoplasm | kinase |
| CDDP 04 | CSNK2A2 | casein kinase 2 alpha 2 | Cytoplasm | kinase |
| CDDP 04 | EPHA8 | EPH receptor A8 | Plasma Membrane | kinase |
| CDDP 04 | EPHB6 | EPH receptor B6 | Plasma Membrane | kinase |
| CDDP 04 | FES | FES proto-oncogene, tyrosine kinase | Cytoplasm | kinase |
| CDDP 04 | FRK | fyn related Src family tyrosine kinase | Nucleus | kinase |
| CDDP 04 | GAK | cyclin G associated kinase | Nucleus | kinase |
| CDDP 04 | HIPK3 | homeodomain interacting protein kinase 3 | Nucleus | kinase |
| CDDP 04 | EIF2AK1 | eukaryotic translation initiation factor 2 alpha kinase 1 | Cytoplasm | kinase |
| CDDP 04 | ITK | IL2 inducible T cell kinase | Cytoplasm | kinase |
| CDDP 04 | MAPK10 | mitogen-activated protein kinase 10 | Cytoplasm | kinase |
| CDDP 04 | LIMK1 | LIM domain kinase 1 | Cytoplasm | kinase |
| CDDP 04 | MAP4K1 | mitogen-activated protein kinase kinase kinase kinase 1 | Cytoplasm | kinase |
| CDDP 04 | MAP4K3 | mitogen-activated protein kinase kinase kinase kinase 3 | Other | kinase |
| CDDP 04 | MAPKAPK5 | MAPK activated protein kinase 5 | Cytoplasm | kinase |
| CDDP 04 | MINK1 | misshapen like kinase 1 | Cytoplasm | kinase |
| CDDP 04 | MKNK1 | MAPK interacting serine/threonine kinase 1 | Cytoplasm | kinase |
| CDDP 04 | PIM2 | Pim-2 proto-oncogene, serine/threonine kinase | Nucleus | kinase |
| CDDP 04 | PRKCA | protein kinase C alpha | Cytoplasm | kinase |
| CDDP 04 | PRKG1 | protein kinase cGMP-dependent 1 | Cytoplasm | kinase |
| CDDP 04 | RIPK2 | receptor interacting serine/threonine kinase 2 | Plasma Membrane | kinase |
| CDDP 04 | ROCK2 | Rho associated coiled-coil containing protein kinase 2 | Cytoplasm | kinase |
| CDDP 04 | SIK2 | salt inducible kinase 2 | Cytoplasm | kinase |
| CDDP 04 | SRMS | src-related kinase lacking C-terminal regulatory tyrosine and N-terminal myristylation sites | Cytoplasm | kinase |
| CDDP 04 | TESK1 | testis associated actin remodelling kinase 1 | Nucleus | kinase |
| CDDP 04 | TGFBR1 | transforming growth factor beta receptor 1 | Plasma Membrane | kinase |
| CDDP 04 | MAPK14 | mitogen-activated protein kinase 14 | Cytoplasm | kinase |
| CDDP 05 | ACVR2A | activin A receptor type 2A | Plasma Membrane | kinase |
| CDDP 05 | ACVRL1 | activin A receptor like type 1 | Plasma Membrane | kinase |
| CDDP 05 | CDK15 | cyclin dependent kinase 15 | Plasma Membrane | kinase |
| CDDP 05 | CDKL2 | cyclin dependent kinase like 2 | Nucleus | kinase |
| CDDP 05 | CDKL3 | cyclin dependent kinase like 3 | Cytoplasm | kinase |
| CDDP 05 | EPHA8 | EPH receptor A8 | Plasma Membrane | kinase |
| CDDP 05 | EPHB6 | EPH receptor B6 | Plasma Membrane | kinase |
| CDDP 05 | FRK | fyn related Src family tyrosine kinase | Nucleus | kinase |
| CDDP 05 | GAK | cyclin G associated kinase | Nucleus | kinase |
| CDDP 05 | HCK | HCK proto-oncogene, Src family tyrosine kinase | Cytoplasm | kinase |
| CDDP 05 | HIPK3 | homeodomain interacting protein kinase 3 | Nucleus | kinase |
| CDDP 05 | EIF2AK1 | eukaryotic translation initiation factor 2 alpha kinase 1 | Cytoplasm | kinase |
| CDDP 05 | NLK | nemo like kinase | Nucleus | kinase |
| CDDP 05 | RIPK2 | receptor interacting serine/threonine kinase 2 | Plasma Membrane | kinase |
| CDDP 05 | SIK2 | salt inducible kinase 2 | Cytoplasm | kinase |
| CDDP 05 | SRC | SRC proto-oncogene, non-receptor tyrosine kinase | Cytoplasm | kinase |
| CDDP 05 | MAPK14 | mitogen-activated protein kinase 14 | Cytoplasm | kinase |
| CDDP 06 | AAK1 | AP2 associated kinase 1 | Cytoplasm | kinase |
| CDDP 06 | AKT2 | AKT serine/threonine kinase 2 | Cytoplasm | kinase |
| CDDP 06 | ACVR1 | activin A receptor type 1 | Plasma Membrane | kinase |
| CDDP 06 | ACVR1B | activin A receptor type 1B | Plasma Membrane | kinase |
| CDDP 06 | ANKK1 | ankyrin repeat and kinase domain containing 1 | Cytoplasm | kinase |
| CDDP 06 | AURKA | aurora kinase A | Nucleus | kinase |
| CDDP 06 | AURKB | aurora kinase B | Nucleus | kinase |
| CDDP 06 | AURKC | aurora kinase C | Nucleus | kinase |
| CDDP 06 | AXL | AXL receptor tyrosine kinase | Plasma Membrane | kinase |
| CDDP 06 | BLK | BLK proto-oncogene, Src family tyrosine kinase | Cytoplasm | kinase |
| CDDP 06 | BMP2K | BMP2 inducible kinase | Nucleus | kinase |
| CDDP 06 | BMPR1B | bone morphogenetic protein receptor type 1B | Plasma Membrane | kinase |
| CDDP 06 | BMX | BMX non-receptor tyrosine kinase | Cytoplasm | kinase |
| CDDP 06 | BRSK1 | BR serine/threonine kinase 1 | Cytoplasm | kinase |
| CDDP 06 | BTK | Bruton tyrosine kinase | Cytoplasm | kinase |
| CDDP 06 | CAMK1D | calcium/calmodulin dependent protein kinase ID | Cytoplasm | kinase |
| CDDP 06 | CAMK1G | calcium/calmodulin dependent protein kinase IG | Cytoplasm | kinase |
| CDDP 06 | CAMK2A | calcium/calmodulin dependent protein kinase II alpha | Cytoplasm | kinase |
| CDDP 06 | CAMK2B | calcium/calmodulin dependent protein kinase II beta | Cytoplasm | kinase |
| CDDP 06 | CAMK2D | calcium/calmodulin dependent protein kinase II delta | Cytoplasm | kinase |
| CDDP 06 | CAMK2G | calcium/calmodulin dependent protein kinase II gamma | Cytoplasm | kinase |
| CDDP 06 | CAMKK1 | calcium/calmodulin dependent protein kinase kinase 1 | Cytoplasm | kinase |
| CDDP 06 | CAMKK2 | calcium/calmodulin dependent protein kinase kinase 2 | Cytoplasm | kinase |
| CDDP 06 | CDC42BPB | CDC42 binding protein kinase beta | Cytoplasm | kinase |
| CDDP 06 | CDC7 | cell division cycle 7 | Nucleus | kinase |
| CDDP 06 | CDK3 | cyclin dependent kinase 3 | Other | kinase |
| CDDP 06 | CDK5 | cyclin dependent kinase 5 | Nucleus | kinase |
| CDDP 06 | CDK6 | cyclin dependent kinase 6 | Nucleus | kinase |
| CDDP 06 | CDK7 | cyclin dependent kinase 7 | Nucleus | kinase |
| CDDP 06 | CDK8 | cyclin dependent kinase 8 | Nucleus | kinase |
| CDDP 06 | CDK9 | cyclin dependent kinase 9 | Nucleus | kinase |
| CDDP 06 | CDKL2 | cyclin dependent kinase like 2 | Nucleus | kinase |
| CDDP 06 | CDKL3 | cyclin dependent kinase like 3 | Cytoplasm | kinase |
| CDDP 06 | CDKL5 | cyclin dependent kinase like 5 | Nucleus | kinase |
| CDDP 06 | CHUK | component of inhibitor of nuclear factor kappa B kinase complex | Cytoplasm | kinase |
| CDDP 06 | CIT | citron rho-interacting serine/threonine kinase | Cytoplasm | kinase |
| CDDP 06 | CLK4 | CDC like kinase 4 | Nucleus | kinase |
| CDDP 06 | CSK | C-terminal Src kinase | Cytoplasm | kinase |
| CDDP 06 | CSNK1A1 | casein kinase 1 alpha 1 | Cytoplasm | kinase |
| CDDP 06 | CSNK1A1L | casein kinase 1 alpha 1 like | Cytoplasm | kinase |
| CDDP 06 | CSNK1D | casein kinase 1 delta | Cytoplasm | kinase |
| CDDP 06 | CSNK1E | casein kinase 1 epsilon | Cytoplasm | kinase |
| CDDP 06 | CSNK1G2 | casein kinase 1 gamma 2 | Cytoplasm | kinase |
| CDDP 06 | CSNK2A1 | casein kinase 2 alpha 1 | Nucleus | kinase |
| CDDP 06 | CSNK2A2 | casein kinase 2 alpha 2 | Cytoplasm | kinase |
| CDDP 06 | CHEK1 | checkpoint kinase 1 | Nucleus | kinase |
| CDDP 06 | CHEK2 | checkpoint kinase 2 | Nucleus | kinase |
| CDDP 06 | DAPK1 | death associated protein kinase 1 | Cytoplasm | kinase |
| CDDP 06 | DAPK3 | death associated protein kinase 3 | Cytoplasm | kinase |
| CDDP 06 | DDR1 | discoidin domain receptor tyrosine kinase 1 | Plasma Membrane | kinase |
| CDDP 06 | DYRK1A | dual specificity tyrosine phosphorylation regulated kinase 1A | Nucleus | kinase |
| CDDP 06 | DYRK1B | dual specificity tyrosine phosphorylation regulated kinase 1B | Nucleus | kinase |
| CDDP 06 | DYRK2 | dual specificity tyrosine phosphorylation regulated kinase 2 | Cytoplasm | kinase |
| CDDP 06 | DYRK3 | dual specificity tyrosine phosphorylation regulated kinase 3 | Nucleus | kinase |
| CDDP 06 | DYRK4 | dual specificity tyrosine phosphorylation regulated kinase 4 | Nucleus | kinase |
| CDDP 06 | EIF2AK2 | eukaryotic translation initiation factor 2 alpha kinase 2 | Cytoplasm | kinase |
| CDDP 06 | EPHA1 | EPH receptor A1 | Plasma Membrane | kinase |
| CDDP 06 | EPHA3 | EPH receptor A3 | Plasma Membrane | kinase |
| CDDP 06 | EPHA4 | EPH receptor A4 | Plasma Membrane | kinase |
| CDDP 06 | EPHA5 | EPH receptor A5 | Plasma Membrane | kinase |
| CDDP 06 | EPHA7 | EPH receptor A7 | Plasma Membrane | kinase |
| CDDP 06 | EPHA8 | EPH receptor A8 | Plasma Membrane | kinase |
| CDDP 06 | EPHB6 | EPH receptor B6 | Plasma Membrane | kinase |
| CDDP 06 | MAPK1 | mitogen-activated protein kinase 1 | Cytoplasm | kinase |
| CDDP 06 | MAPK7 | mitogen-activated protein kinase 7 | Cytoplasm | kinase |
| CDDP 06 | MAPK15 | mitogen-activated protein kinase 15 | Cytoplasm | kinase |
| CDDP 06 | FER | FER tyrosine kinase | Cytoplasm | kinase |
| CDDP 06 | FES | FES proto-oncogene, tyrosine kinase | Cytoplasm | kinase |
| CDDP 06 | FGFR1 | fibroblast growth factor receptor 1 | Plasma Membrane | kinase |
| CDDP 06 | FGFR2 | fibroblast growth factor receptor 2 | Plasma Membrane | kinase |
| CDDP 06 | FGFR3 | fibroblast growth factor receptor 3 | Plasma Membrane | kinase |
| CDDP 06 | FGR | FGR proto-oncogene, Src family tyrosine kinase | Nucleus | kinase |
| CDDP 06 | FLT3 | fms related tyrosine kinase 3 | Plasma Membrane | kinase |
| CDDP 06 | FRK | fyn related Src family tyrosine kinase | Nucleus | kinase |
| CDDP 06 | FYN | FYN proto-oncogene, Src family tyrosine kinase | Plasma Membrane | kinase |
| CDDP 06 | GAK | cyclin G associated kinase | Nucleus | kinase |
| CDDP 06 | GRK4 | G protein-coupled receptor kinase 4 | Plasma Membrane | kinase |
| CDDP 06 | GRK5 | G protein-coupled receptor kinase 5 | Plasma Membrane | kinase |
| CDDP 06 | GRK7 | G protein-coupled receptor kinase 7 | Cytoplasm | kinase |
| CDDP 06 | GSK3A | glycogen synthase kinase 3 alpha | Nucleus | kinase |
| CDDP 06 | GSK3B | glycogen synthase kinase 3 beta | Nucleus | kinase |
| CDDP 06 | HCK | HCK proto-oncogene, Src family tyrosine kinase | Cytoplasm | kinase |
| CDDP 06 | HIPK1 | homeodomain interacting protein kinase 1 | Nucleus | kinase |
| CDDP 06 | HIPK2 | homeodomain interacting protein kinase 2 | Nucleus | kinase |
| CDDP 06 | HIPK3 | homeodomain interacting protein kinase 3 | Nucleus | kinase |
| CDDP 06 | HIPK4 | homeodomain interacting protein kinase 4 | Cytoplasm | kinase |
| CDDP 06 | EIF2AK1 | eukaryotic translation initiation factor 2 alpha kinase 1 | Cytoplasm | kinase |
| CDDP 06 | CILK1 | ciliogenesis Associated Kinase 1 | Nucleus | kinase |
| CDDP 06 | IKBKE | inhibitor of nuclear factor kappa B kinase subunit epsilon | Cytoplasm | kinase |
| CDDP 06 | INSR | insulin receptor | Plasma Membrane | kinase |
| CDDP 06 | IRAK1 | interleukin 1 receptor associated kinase 1 | Plasma Membrane | kinase |
| CDDP 06 | IRAK4 | interleukin 1 receptor associated kinase 4 | Cytoplasm | kinase |
| CDDP 06 | JAK2 | Janus kinase 2 | Cytoplasm | kinase |
| CDDP 06 | JAK3 | Janus kinase 3 | Cytoplasm | kinase |
| CDDP 06 | MAPK8 | mitogen-activated protein kinase 8 | Cytoplasm | kinase |
| CDDP 06 | MAPK9 | mitogen-activated protein kinase 9 | Cytoplasm | kinase |
| CDDP 06 | MAPK10 | mitogen-activated protein kinase 10 | Cytoplasm | kinase |
| CDDP 06 | LATS1 | large tumor suppressor kinase 1 | Nucleus | kinase |
| CDDP 06 | LIMK1 | LIM domain kinase 1 | Cytoplasm | kinase |
| CDDP 06 | LRRK2 | leucine rich repeat kinase 2 | Cytoplasm | kinase |
| CDDP 06 | LTK | leukocyte receptor tyrosine kinase | Plasma Membrane | kinase |
| CDDP 06 | LYN | LYN proto-oncogene, Src family tyrosine kinase | Cytoplasm | kinase |
| CDDP 06 | MAP3K10 | mitogen-activated protein kinase kinase kinase 10 | Cytoplasm | kinase |
| CDDP 06 | MAP3K11 | mitogen-activated protein kinase kinase kinase 11 | Cytoplasm | kinase |
| CDDP 06 | MAP3K13 | mitogen-activated protein kinase kinase kinase 13 | Cytoplasm | kinase |
| CDDP 06 | MAP3K2 | mitogen-activated protein kinase kinase kinase 2 | Cytoplasm | kinase |
| CDDP 06 | MAP3K3 | mitogen-activated protein kinase kinase kinase 3 | Cytoplasm | kinase |
| CDDP 06 | MAP3K5 | mitogen-activated protein kinase kinase kinase 5 | Cytoplasm | kinase |
| CDDP 06 | MAP3K6 | mitogen-activated protein kinase kinase kinase 6 | Other | kinase |
| CDDP 06 | MAP3K9 | mitogen-activated protein kinase kinase kinase 9 | Cytoplasm | kinase |
| CDDP 06 | MAP4K1 | mitogen-activated protein kinase kinase kinase kinase 1 | Cytoplasm | kinase |
| CDDP 06 | MAP4K2 | mitogen-activated protein kinase kinase kinase kinase 2 | Cytoplasm | kinase |
| CDDP 06 | MAP4K3 | mitogen-activated protein kinase kinase kinase kinase 3 | Other | kinase |
| CDDP 06 | MAP4K4 | mitogen-activated protein kinase kinase kinase kinase 4 | Cytoplasm | kinase |
| CDDP 06 | MAP4K5 | mitogen-activated protein kinase kinase kinase kinase 5 | Cytoplasm | kinase |
| CDDP 06 | MAPKAPK3 | MAPK activated protein kinase 3 | Nucleus | kinase |
| CDDP 06 | MAPKAPK5 | MAPK activated protein kinase 5 | Cytoplasm | kinase |
| CDDP 06 | MARK1 | microtubule affinity regulating kinase 1 | Cytoplasm | kinase |
| CDDP 06 | MARK2 | microtubule affinity regulating kinase 2 | Cytoplasm | kinase |
| CDDP 06 | MARK3 | microtubule affinity regulating kinase 3 | Cytoplasm | kinase |
| CDDP 06 | MARK4 | microtubule affinity regulating kinase 4 | Cytoplasm | kinase |
| CDDP 06 | MAST1 | microtubule associated serine/threonine kinase 1 | Cytoplasm | kinase |
| CDDP 06 | MATK | megakaryocyte-associated tyrosine kinase | Cytoplasm | kinase |
| CDDP 06 | MAP2K1 | mitogen-activated protein kinase kinase 1 | Cytoplasm | kinase |
| CDDP 06 | MAP2K2 | mitogen-activated protein kinase kinase 2 | Cytoplasm | kinase |
| CDDP 06 | MELK | maternal embryonic leucine zipper kinase | Cytoplasm | kinase |
| CDDP 06 | MET | MET proto-oncogene, receptor tyrosine kinase | Plasma Membrane | kinase |
| CDDP 06 | MINK1 | misshapen like kinase 1 | Cytoplasm | kinase |
| CDDP 06 | MAP2K3 | mitogen-activated protein kinase kinase 3 | Cytoplasm | kinase |
| CDDP 06 | MAP2K4 | mitogen-activated protein kinase kinase 4 | Cytoplasm | kinase |
| CDDP 06 | MAP2K6 | mitogen-activated protein kinase kinase 6 | Cytoplasm | kinase |
| CDDP 06 | MKNK2 | MAPK interacting serine/threonine kinase 2 | Cytoplasm | kinase |
| CDDP 06 | TTK | TTK protein kinase | Nucleus | kinase |
| CDDP 06 | RPS6KA5 | ribosomal protein S6 kinase A5 | Nucleus | kinase |
| CDDP 06 | RPS6KA4 | ribosomal protein S6 kinase A4 | Cytoplasm | kinase |
| CDDP 06 | SRPK3 | SRSF protein kinase 3 | Cytoplasm | kinase |
| CDDP 06 | MST1R | macrophage stimulating 1 receptor | Plasma Membrane | kinase |
| CDDP 06 | STK26 | serine/threonine kinase 26 | Nucleus | kinase |
| CDDP 06 | MYLK | myosin light chain kinase | Cytoplasm | kinase |
| CDDP 06 | MYLK2 | myosin light chain kinase 2 | Cytoplasm | kinase |
| CDDP 06 | MYO3A | myosin IIIA | Cytoplasm | kinase |
| CDDP 06 | MYO3B | myosin IIIB | Plasma Membrane | kinase |
| CDDP 06 | NEK2 | NIMA related kinase 2 | Cytoplasm | kinase |
| CDDP 06 | NEK4 | NIMA related kinase 4 | Nucleus | kinase |
| CDDP 06 | NEK5 | NIMA related kinase 5 | Other | kinase |
| CDDP 06 | NEK7 | NIMA related kinase 7 | Nucleus | kinase |
| CDDP 06 | NLK | nemo like kinase | Nucleus | kinase |
| CDDP 06 | NTRK1 | neurotrophic receptor tyrosine kinase 1 | Plasma Membrane | kinase |
| CDDP 06 | NUAK2 | NUAK family kinase 2 | Other | kinase |
| CDDP 06 | OXSR1 | oxidative stress responsive kinase 1 | Nucleus | kinase |
| CDDP 06 | PAK2 | p21 (RAC1) activated kinase 2 | Cytoplasm | kinase |
| CDDP 06 | PAK3 | p21 (RAC1) activated kinase 3 | Cytoplasm | kinase |
| CDDP 06 | PAK6 | p21 (RAC1) activated kinase 6 | Cytoplasm | kinase |
| CDDP 06 | PAK5 | p21 (RAC1) activated kinase 5 | Nucleus | kinase |
| CDDP 06 | PASK | PAS domain containing serine/threonine kinase | Cytoplasm | kinase |
| CDDP 06 | PBK | PDZ binding kinase | Cytoplasm | kinase |
| CDDP 06 | CDK17 | cyclin dependent kinase 17 | Cytoplasm | kinase |
| CDDP 06 | PDPK1 | 3-phosphoinositide dependent protein kinase 1 | Cytoplasm | kinase |
| CDDP 06 | EIF2AK3 | eukaryotic translation initiation factor 2 alpha kinase 3 | Cytoplasm | kinase |
| CDDP 06 | PHKG1 | phosphorylase kinase catalytic subunit gamma 1 | Cytoplasm | kinase |
| CDDP 06 | PHKG2 | phosphorylase kinase catalytic subunit gamma 2 | Cytoplasm | kinase |
| CDDP 06 | PIM1 | Pim-1 proto-oncogene, serine/threonine kinase | Cytoplasm | kinase |
| CDDP 06 | PIM3 | Pim-3 proto-oncogene, serine/threonine kinase | Cytoplasm | kinase |
| CDDP 06 | PRKCA | protein kinase C alpha | Cytoplasm | kinase |
| CDDP 06 | PRKCB | protein kinase C beta | Cytoplasm | kinase |
| CDDP 06 | PRKCD | protein kinase C delta | Cytoplasm | kinase |
| CDDP 06 | PRKCQ | protein kinase C theta | Cytoplasm | kinase |
| CDDP 06 | PKD1 | polycystin 1, transient receptor potential channel interacting | Plasma Membrane | kinase |
| CDDP 06 | PKD2 | polycystin 2, transient receptor potential cation channel | Plasma Membrane | kinase |
| CDDP 06 | PKD3 | polycystic Kidney Disease 3 (Autosomal Dominant) | Plasma Membrane | kinase |
| CDDP 06 | PKN1 | protein kinase N1 | Cytoplasm | kinase |
| CDDP 06 | PKN2 | protein kinase N2 | Cytoplasm | kinase |
| CDDP 06 | PLK1 | polo like kinase 1 | Nucleus | kinase |
| CDDP 06 | PLK2 | polo like kinase 2 | Nucleus | kinase |
| CDDP 06 | PLK3 | polo like kinase 3 | Nucleus | kinase |
| CDDP 06 | PLK4 | polo like kinase 4 | Cytoplasm | kinase |
| CDDP 06 | PRKAA1 | protein kinase AMP-activated catalytic subunit alpha 1 | Cytoplasm | kinase |
| CDDP 06 | PRKAA2 | protein kinase AMP-activated catalytic subunit alpha 2 | Cytoplasm | kinase |
| CDDP 06 | PRKG1 | protein kinase cGMP-dependent 1 | Cytoplasm | kinase |
| CDDP 06 | PRKG2 | protein kinase cGMP-dependent 2 | Cytoplasm | kinase |
| CDDP 06 | PRKX | protein kinase X-linked | Cytoplasm | kinase |
| CDDP 06 | SIK3 | SIK family kinase 3 | Cytoplasm | kinase |
| CDDP 06 | RAF1 | Raf-1 proto-oncogene, serine/threonine kinase | Cytoplasm | kinase |
| CDDP 06 | RIPK2 | receptor interacting serine/threonine kinase 2 | Plasma Membrane | kinase |
| CDDP 06 | ROCK1 | Rho associated coiled-coil containing protein kinase 1 | Cytoplasm | kinase |
| CDDP 06 | ROCK2 | Rho associated coiled-coil containing protein kinase 2 | Cytoplasm | kinase |
| CDDP 06 | ROS1 | ROS proto-oncogene 1, receptor tyrosine kinase | Plasma Membrane | kinase |
| CDDP 06 | RPS6KB1 | ribosomal protein S6 kinase B1 | Cytoplasm | kinase |
| CDDP 06 | RPS6KA3 | ribosomal protein S6 kinase A3 | Cytoplasm | kinase |
| CDDP 06 | SGK1 | serum/glucocorticoid regulated kinase 1 | Cytoplasm | kinase |
| CDDP 06 | SLK | STE20 like kinase | Nucleus | kinase |
| CDDP 06 | SIK2 | salt inducible kinase 2 | Cytoplasm | kinase |
| CDDP 06 | SRMS | src-related kinase lacking C-terminal regulatory tyrosine and N-terminal myristylation sites | Cytoplasm | kinase |
| CDDP 06 | SRPK1 | SRSF protein kinase 1 | Nucleus | kinase |
| CDDP 06 | STK10 | serine/threonine kinase 10 | Cytoplasm | kinase |
| CDDP 06 | STK11 | serine/threonine kinase 11 | Cytoplasm | kinase |
| CDDP 06 | STK16 | serine/threonine kinase 16 | Cytoplasm | kinase |
| CDDP 06 | STK17A | serine/threonine kinase 17a | Nucleus | kinase |
| CDDP 06 | STK17B | serine/threonine kinase 17b | Nucleus | kinase |
| CDDP 06 | STK24 | serine/threonine kinase 24 | Cytoplasm | kinase |
| CDDP 06 | STK25 | serine/threonine kinase 25 | Cytoplasm | kinase |
| CDDP 06 | STK3 | serine/threonine kinase 3 | Cytoplasm | kinase |
| CDDP 06 | STK33 | serine/threonine kinase 33 | Cytoplasm | kinase |
| CDDP 06 | STK35 | serine/threonine kinase 35 | Cytoplasm | kinase |
| CDDP 06 | STK38L | serine/threonine kinase 38 like | Cytoplasm | kinase |
| CDDP 06 | STK39 | serine/threonine kinase 39 | Nucleus | kinase |
| CDDP 06 | STK4 | serine/threonine kinase 4 | Cytoplasm | kinase |
| CDDP 06 | SYK | spleen associated tyrosine kinase | Cytoplasm | kinase |
| CDDP 06 | MYLK4 | myosin light chain kinase family member 4 | Cytoplasm | kinase |
| CDDP 06 | SBK3 | SH3 domain binding kinase family member 3 | Other | other |
| CDDP 06 | TAOK1 | TAO kinase 1 | Cytoplasm | kinase |
| CDDP 06 | TAOK3 | TAO kinase 3 | Cytoplasm | kinase |
| CDDP 06 | TBK1 | TANK binding kinase 1 | Cytoplasm | kinase |
| CDDP 06 | TESK1 | testis associated actin remodelling kinase 1 | Nucleus | kinase |
| CDDP 06 | TLK1 | tousled like kinase 1 | Nucleus | kinase |
| CDDP 06 | TLK2 | tousled like kinase 2 | Cytoplasm | kinase |
| CDDP 06 | TNIK | TRAF2 and NCK interacting kinase | Plasma Membrane | kinase |
| CDDP 06 | TNK1 | tyrosine kinase non receptor 1 | Cytoplasm | kinase |
| CDDP 06 | TNK2 | tyrosine kinase non receptor 2 | Cytoplasm | kinase |
| CDDP 06 | TNNI3K | TNNI3 interacting kinase | Cytoplasm | kinase |
| CDDP 06 | TSSK1B | testis specific serine kinase 1B | Cytoplasm | kinase |
| CDDP 06 | TSSK2 | testis specific serine kinase 2 | Cytoplasm | kinase |
| CDDP 06 | TXK | TXK tyrosine kinase | Cytoplasm | kinase |
| CDDP 06 | TYK2 | tyrosine kinase 2 | Plasma Membrane | kinase |
| CDDP 06 | TYRO3 | TYRO3 protein tyrosine kinase | Plasma Membrane | kinase |
| CDDP 06 | ULK1 | unc-51 like autophagy activating kinase 1 | Cytoplasm | kinase |
| CDDP 06 | ULK2 | unc-51 like autophagy activating kinase 2 | Cytoplasm | kinase |
| CDDP 06 | ULK3 | unc-51 like kinase 3 | Cytoplasm | kinase |
| CDDP 06 | FLT4 | fms related tyrosine kinase 4 | Plasma Membrane | transmembrane receptor |
| CDDP 06 | YES1 | YES proto-oncogene 1, Src family tyrosine kinase | Cytoplasm | kinase |
| CDDP 06 | MAP3K20 | mitogen-activated protein kinase kinase kinase 20 | Cytoplasm | kinase |
| CDDP 06 | ZAP70 | zeta chain of T cell receptor associated protein kinase 70 | Plasma Membrane | kinase |
| CDDP 07 | AKT2 | AKT serine/threonine kinase 2 | Cytoplasm | kinase |
| CDDP 07 | AURKA | aurora kinase A | Nucleus | kinase |
| CDDP 07 | AURKB | aurora kinase B | Nucleus | kinase |
| CDDP 07 | AURKC | aurora kinase C | Nucleus | kinase |
| CDDP 07 | AXL | AXL receptor tyrosine kinase | Plasma Membrane | kinase |
| CDDP 07 | BLK | BLK proto-oncogene, Src family tyrosine kinase | Cytoplasm | kinase |
| CDDP 07 | BMX | BMX non-receptor tyrosine kinase | Cytoplasm | kinase |
| CDDP 07 | BRSK1 | BR serine/threonine kinase 1 | Cytoplasm | kinase |
| CDDP 07 | BTK | Bruton tyrosine kinase | Cytoplasm | kinase |
| CDDP 07 | CAMK1D | calcium/calmodulin dependent protein kinase ID | Cytoplasm | kinase |
| CDDP 07 | CAMK2D | calcium/calmodulin dependent protein kinase II delta | Cytoplasm | kinase |
| CDDP 07 | CAMKK1 | calcium/calmodulin dependent protein kinase kinase 1 | Cytoplasm | kinase |
| CDDP 07 | CDK9 | cyclin dependent kinase 9 | Nucleus | kinase |
| CDDP 07 | CDKL2 | cyclin dependent kinase like 2 | Nucleus | kinase |
| CDDP 07 | CDKL3 | cyclin dependent kinase like 3 | Cytoplasm | kinase |
| CDDP 07 | CLK4 | CDC like kinase 4 | Nucleus | kinase |
| CDDP 07 | CSNK1E | casein kinase 1 epsilon | Cytoplasm | kinase |
| CDDP 07 | CHEK1 | checkpoint kinase 1 | Nucleus | kinase |
| CDDP 07 | DDR1 | discoidin domain receptor tyrosine kinase 1 | Plasma Membrane | kinase |
| CDDP 07 | DYRK1B | dual specificity tyrosine phosphorylation regulated kinase 1B | Nucleus | kinase |
| CDDP 07 | EPHA3 | EPH receptor A3 | Plasma Membrane | kinase |
| CDDP 07 | EPHA5 | EPH receptor A5 | Plasma Membrane | kinase |
| CDDP 07 | EPHA7 | EPH receptor A7 | Plasma Membrane | kinase |
| CDDP 07 | EPHB6 | EPH receptor B6 | Plasma Membrane | kinase |
| CDDP 07 | MAPK7 | mitogen-activated protein kinase 7 | Cytoplasm | kinase |
| CDDP 07 | FER | FER tyrosine kinase | Cytoplasm | kinase |
| CDDP 07 | FES | FES proto-oncogene, tyrosine kinase | Cytoplasm | kinase |
| CDDP 07 | FGFR1 | fibroblast growth factor receptor 1 | Plasma Membrane | kinase |
| CDDP 07 | FGFR3 | fibroblast growth factor receptor 3 | Plasma Membrane | kinase |
| CDDP 07 | FGR | FGR proto-oncogene, Src family tyrosine kinase | Nucleus | kinase |
| CDDP 07 | FLT3 | fms related tyrosine kinase 3 | Plasma Membrane | kinase |
| CDDP 07 | FYN | FYN proto-oncogene, Src family tyrosine kinase | Plasma Membrane | kinase |
| CDDP 07 | GAK | cyclin G associated kinase | Nucleus | kinase |
| CDDP 07 | GRK1 | G protein-coupled receptor kinase 1 | Plasma Membrane | kinase |
| CDDP 07 | GRK4 | G protein-coupled receptor kinase 4 | Plasma Membrane | kinase |
| CDDP 07 | GRK5 | G protein-coupled receptor kinase 5 | Plasma Membrane | kinase |
| CDDP 07 | GRK7 | G protein-coupled receptor kinase 7 | Cytoplasm | kinase |
| CDDP 07 | HCK | HCK proto-oncogene, Src family tyrosine kinase | Cytoplasm | kinase |
| CDDP 07 | ERBB2 | erb-b2 receptor tyrosine kinase 2 | Plasma Membrane | kinase |
| CDDP 07 | HIPK3 | homeodomain interacting protein kinase 3 | Nucleus | kinase |
| CDDP 07 | EIF2AK1 | eukaryotic translation initiation factor 2 alpha kinase 1 | Cytoplasm | kinase |
| CDDP 07 | CILK1 | ciliogenesis Associated Kinase 1 | Nucleus | kinase |
| CDDP 07 | INSR | insulin receptor | Plasma Membrane | kinase |
| CDDP 07 | MAPK10 | mitogen-activated protein kinase 10 | Cytoplasm | kinase |
| CDDP 07 | KIT | KIT proto-oncogene, receptor tyrosine kinase | Plasma Membrane | transmembrane receptor |
| CDDP 07 | LIMK1 | LIM domain kinase 1 | Cytoplasm | kinase |
| CDDP 07 | LRRK2 | leucine rich repeat kinase 2 | Cytoplasm | kinase |
| CDDP 07 | LTK | leukocyte receptor tyrosine kinase | Plasma Membrane | kinase |
| CDDP 07 | LYN | LYN proto-oncogene, Src family tyrosine kinase | Cytoplasm | kinase |
| CDDP 07 | MAP3K11 | mitogen-activated protein kinase kinase kinase 11 | Cytoplasm | kinase |
| CDDP 07 | MAP3K13 | mitogen-activated protein kinase kinase kinase 13 | Cytoplasm | kinase |
| CDDP 07 | MAP3K15 | mitogen-activated protein kinase kinase kinase 15 | Other | other |
| CDDP 07 | MAP3K2 | mitogen-activated protein kinase kinase kinase 2 | Cytoplasm | kinase |
| CDDP 07 | MAP3K3 | mitogen-activated protein kinase kinase kinase 3 | Cytoplasm | kinase |
| CDDP 07 | MAP3K9 | mitogen-activated protein kinase kinase kinase 9 | Cytoplasm | kinase |
| CDDP 07 | MAP4K1 | mitogen-activated protein kinase kinase kinase kinase 1 | Cytoplasm | kinase |
| CDDP 07 | MAP4K3 | mitogen-activated protein kinase kinase kinase kinase 3 | Other | kinase |
| CDDP 07 | MAP4K5 | mitogen-activated protein kinase kinase kinase kinase 5 | Cytoplasm | kinase |
| CDDP 07 | MARK2 | microtubule affinity regulating kinase 2 | Cytoplasm | kinase |
| CDDP 07 | MAST1 | microtubule associated serine/threonine kinase 1 | Cytoplasm | kinase |
| CDDP 07 | MAP2K1 | mitogen-activated protein kinase kinase 1 | Cytoplasm | kinase |
| CDDP 07 | MAP2K2 | mitogen-activated protein kinase kinase 2 | Cytoplasm | kinase |
| CDDP 07 | MELK | maternal embryonic leucine zipper kinase | Cytoplasm | kinase |
| CDDP 07 | MET | MET proto-oncogene, receptor tyrosine kinase | Plasma Membrane | kinase |
| CDDP 07 | RPS6KA4 | ribosomal protein S6 kinase A4 | Cytoplasm | kinase |
| CDDP 07 | MYLK2 | myosin light chain kinase 2 | Cytoplasm | kinase |
| CDDP 07 | NEK2 | NIMA related kinase 2 | Cytoplasm | kinase |
| CDDP 07 | NLK | nemo like kinase | Nucleus | kinase |
| CDDP 07 | NTRK1 | neurotrophic receptor tyrosine kinase 1 | Plasma Membrane | kinase |
| CDDP 07 | OXSR1 | oxidative stress responsive kinase 1 | Nucleus | kinase |
| CDDP 07 | PDPK1 | 3-phosphoinositide dependent protein kinase 1 | Cytoplasm | kinase |
| CDDP 07 | PKD2 | polycystin 2, transient receptor potential cation channel | Plasma Membrane | kinase |
| CDDP 07 | PLK1 | polo like kinase 1 | Nucleus | kinase |
| CDDP 07 | PLK3 | polo like kinase 3 | Nucleus | kinase |
| CDDP 07 | PLK4 | polo like kinase 4 | Cytoplasm | kinase |
| CDDP 07 | PRKAA1 | protein kinase AMP-activated catalytic subunit alpha 1 | Cytoplasm | kinase |
| CDDP 07 | RAF1 | Raf-1 proto-oncogene, serine/threonine kinase | Cytoplasm | kinase |
| CDDP 07 | RIPK2 | receptor interacting serine/threonine kinase 2 | Plasma Membrane | kinase |
| CDDP 07 | ROS1 | ROS proto-oncogene 1, receptor tyrosine kinase | Plasma Membrane | kinase |
| CDDP 07 | RPS6KB1 | ribosomal protein S6 kinase B1 | Cytoplasm | kinase |
| CDDP 07 | SLK | STE20 like kinase | Nucleus | kinase |
| CDDP 07 | SIK2 | salt inducible kinase 2 | Cytoplasm | kinase |
| CDDP 07 | STK17B | serine/threonine kinase 17b | Nucleus | kinase |
| CDDP 07 | STK3 | serine/threonine kinase 3 | Cytoplasm | kinase |
| CDDP 07 | STK39 | serine/threonine kinase 39 | Nucleus | kinase |
| CDDP 07 | SYK | spleen associated tyrosine kinase | Cytoplasm | kinase |
| CDDP 07 | TAOK1 | TAO kinase 1 | Cytoplasm | kinase |
| CDDP 07 | TAOK3 | TAO kinase 3 | Cytoplasm | kinase |
| CDDP 07 | TESK1 | testis associated actin remodelling kinase 1 | Nucleus | kinase |
| CDDP 07 | TNK2 | tyrosine kinase non receptor 2 | Cytoplasm | kinase |
| CDDP 07 | TSSK1B | testis specific serine kinase 1B | Cytoplasm | kinase |
| CDDP 07 | TSSK2 | testis specific serine kinase 2 | Cytoplasm | kinase |
| CDDP 07 | TXK | TXK tyrosine kinase | Cytoplasm | kinase |
| CDDP 07 | TYRO3 | TYRO3 protein tyrosine kinase | Plasma Membrane | kinase |
| CDDP 07 | ULK1 | unc-51 like autophagy activating kinase 1 | Cytoplasm | kinase |
| CDDP 07 | ULK2 | unc-51 like autophagy activating kinase 2 | Cytoplasm | kinase |
| CDDP 07 | ULK3 | unc-51 like kinase 3 | Cytoplasm | kinase |
| CDDP 07 | FLT4 | fms related tyrosine kinase 4 | Plasma Membrane | transmembrane receptor |
| CDDP 07 | YES1 | YES proto-oncogene 1, Src family tyrosine kinase | Cytoplasm | kinase |
| CDDP 07 | ZAP70 | zeta chain of T cell receptor associated protein kinase 70 | Plasma Membrane | kinase |
| CDDP 08 | AKT2 | AKT serine/threonine kinase 2 | Cytoplasm | kinase |
| CDDP 08 | AURKA | aurora kinase A | Nucleus | kinase |
| CDDP 08 | AURKB | aurora kinase B | Nucleus | kinase |
| CDDP 08 | AURKC | aurora kinase C | Nucleus | kinase |
| CDDP 08 | AXL | AXL receptor tyrosine kinase | Plasma Membrane | kinase |
| CDDP 08 | BLK | BLK proto-oncogene, Src family tyrosine kinase | Cytoplasm | kinase |
| CDDP 08 | BMX | BMX non-receptor tyrosine kinase | Cytoplasm | kinase |
| CDDP 08 | BRSK1 | BR serine/threonine kinase 1 | Cytoplasm | kinase |
| CDDP 08 | BTK | Bruton tyrosine kinase | Cytoplasm | kinase |
| CDDP 08 | CAMK1D | calcium/calmodulin dependent protein kinase ID | Cytoplasm | kinase |
| CDDP 08 | CAMK2D | calcium/calmodulin dependent protein kinase II delta | Cytoplasm | kinase |
| CDDP 08 | CAMKK1 | calcium/calmodulin dependent protein kinase kinase 1 | Cytoplasm | kinase |
| CDDP 08 | CDK9 | cyclin dependent kinase 9 | Nucleus | kinase |
| CDDP 08 | CDKL2 | cyclin dependent kinase like 2 | Nucleus | kinase |
| CDDP 08 | CDKL3 | cyclin dependent kinase like 3 | Cytoplasm | kinase |
| CDDP 08 | CLK4 | CDC like kinase 4 | Nucleus | kinase |
| CDDP 08 | CSNK1E | casein kinase 1 epsilon | Cytoplasm | kinase |
| CDDP 08 | CHEK1 | checkpoint kinase 1 | Nucleus | kinase |
| CDDP 08 | DDR1 | discoidin domain receptor tyrosine kinase 1 | Plasma Membrane | kinase |
| CDDP 08 | DYRK1B | dual specificity tyrosine phosphorylation regulated kinase 1B | Nucleus | kinase |
| CDDP 08 | EPHA3 | EPH receptor A3 | Plasma Membrane | kinase |
| CDDP 08 | EPHA5 | EPH receptor A5 | Plasma Membrane | kinase |
| CDDP 08 | EPHA7 | EPH receptor A7 | Plasma Membrane | kinase |
| CDDP 08 | EPHB6 | EPH receptor B6 | Plasma Membrane | kinase |
| CDDP 08 | MAPK7 | mitogen-activated protein kinase 7 | Cytoplasm | kinase |
| CDDP 08 | FER | FER tyrosine kinase | Cytoplasm | kinase |
| CDDP 08 | FES | FES proto-oncogene, tyrosine kinase | Cytoplasm | kinase |
| CDDP 08 | FGFR1 | fibroblast growth factor receptor 1 | Plasma Membrane | kinase |
| CDDP 08 | FGFR3 | fibroblast growth factor receptor 3 | Plasma Membrane | kinase |
| CDDP 08 | FGR | FGR proto-oncogene, Src family tyrosine kinase | Nucleus | kinase |
| CDDP 08 | FLT3 | fms related tyrosine kinase 3 | Plasma Membrane | kinase |
| CDDP 08 | FYN | FYN proto-oncogene, Src family tyrosine kinase | Plasma Membrane | kinase |
| CDDP 08 | GAK | cyclin G associated kinase | Nucleus | kinase |
| CDDP 08 | GRK1 | G protein-coupled receptor kinase 1 | Plasma Membrane | kinase |
| CDDP 08 | GRK4 | G protein-coupled receptor kinase 4 | Plasma Membrane | kinase |
| CDDP 08 | GRK5 | G protein-coupled receptor kinase 5 | Plasma Membrane | kinase |
| CDDP 08 | GRK7 | G protein-coupled receptor kinase 7 | Cytoplasm | kinase |
| CDDP 08 | HCK | HCK proto-oncogene, Src family tyrosine kinase | Cytoplasm | kinase |
| CDDP 08 | ERBB2 | erb-b2 receptor tyrosine kinase 2 | Plasma Membrane | kinase |
| CDDP 08 | HIPK3 | homeodomain interacting protein kinase 3 | Nucleus | kinase |
| CDDP 08 | EIF2AK1 | eukaryotic translation initiation factor 2 alpha kinase 1 | Cytoplasm | kinase |
| CDDP 08 | CILK1 | ciliogenesis Associated Kinase 1 | Nucleus | kinase |
| CDDP 08 | INSR | insulin receptor | Plasma Membrane | kinase |
| CDDP 08 | MAPK10 | mitogen-activated protein kinase 10 | Cytoplasm | kinase |
| CDDP 08 | KIT | KIT proto-oncogene, receptor tyrosine kinase | Plasma Membrane | transmembrane receptor |
| CDDP 08 | LIMK1 | LIM domain kinase 1 | Cytoplasm | kinase |
| CDDP 08 | LRRK2 | leucine rich repeat kinase 2 | Cytoplasm | kinase |
| CDDP 08 | LTK | leukocyte receptor tyrosine kinase | Plasma Membrane | kinase |
| CDDP 08 | LYN | LYN proto-oncogene, Src family tyrosine kinase | Cytoplasm | kinase |
| CDDP 08 | MAP3K11 | mitogen-activated protein kinase kinase kinase 11 | Cytoplasm | kinase |
| CDDP 08 | MAP3K13 | mitogen-activated protein kinase kinase kinase 13 | Cytoplasm | kinase |
| CDDP 08 | MAP3K15 | mitogen-activated protein kinase kinase kinase 15 | Other | other |
| CDDP 08 | MAP3K2 | mitogen-activated protein kinase kinase kinase 2 | Cytoplasm | kinase |
| CDDP 08 | MAP3K3 | mitogen-activated protein kinase kinase kinase 3 | Cytoplasm | kinase |
| CDDP 08 | MAP3K9 | mitogen-activated protein kinase kinase kinase 9 | Cytoplasm | kinase |
| CDDP 08 | MAP4K1 | mitogen-activated protein kinase kinase kinase kinase 1 | Cytoplasm | kinase |
| CDDP 08 | MAP4K3 | mitogen-activated protein kinase kinase kinase kinase 3 | Other | kinase |
| CDDP 08 | MAP4K5 | mitogen-activated protein kinase kinase kinase kinase 5 | Cytoplasm | kinase |
| CDDP 08 | MARK2 | microtubule affinity regulating kinase 2 | Cytoplasm | kinase |
| CDDP 08 | MAST1 | microtubule associated serine/threonine kinase 1 | Cytoplasm | kinase |
| CDDP 08 | MAP2K1 | mitogen-activated protein kinase kinase 1 | Cytoplasm | kinase |
| CDDP 08 | MAP2K2 | mitogen-activated protein kinase kinase 2 | Cytoplasm | kinase |
| CDDP 08 | MELK | maternal embryonic leucine zipper kinase | Cytoplasm | kinase |
| CDDP 08 | MET | MET proto-oncogene, receptor tyrosine kinase | Plasma Membrane | kinase |
| CDDP 08 | RPS6KA4 | ribosomal protein S6 kinase A4 | Cytoplasm | kinase |
| CDDP 08 | MYLK2 | myosin light chain kinase 2 | Cytoplasm | kinase |
| CDDP 08 | NEK2 | NIMA related kinase 2 | Cytoplasm | kinase |
| CDDP 08 | NLK | nemo like kinase | Nucleus | kinase |
| CDDP 08 | NTRK1 | neurotrophic receptor tyrosine kinase 1 | Plasma Membrane | kinase |
| CDDP 08 | OXSR1 | oxidative stress responsive kinase 1 | Nucleus | kinase |
| CDDP 08 | PDPK1 | 3-phosphoinositide dependent protein kinase 1 | Cytoplasm | kinase |
| CDDP 08 | PKD2 | polycystin 2, transient receptor potential cation channel | Plasma Membrane | kinase |
| CDDP 08 | PLK1 | polo like kinase 1 | Nucleus | kinase |
| CDDP 08 | PLK3 | polo like kinase 3 | Nucleus | kinase |
| CDDP 08 | PLK4 | polo like kinase 4 | Cytoplasm | kinase |
| CDDP 08 | PRKAA1 | protein kinase AMP-activated catalytic subunit alpha 1 | Cytoplasm | kinase |
| CDDP 08 | RAF1 | Raf-1 proto-oncogene, serine/threonine kinase | Cytoplasm | kinase |
| CDDP 08 | RIPK2 | receptor interacting serine/threonine kinase 2 | Plasma Membrane | kinase |
| CDDP 08 | ROS1 | ROS proto-oncogene 1, receptor tyrosine kinase | Plasma Membrane | kinase |
| CDDP 08 | RPS6KB1 | ribosomal protein S6 kinase B1 | Cytoplasm | kinase |
| CDDP 08 | SLK | STE20 like kinase | Nucleus | kinase |
| CDDP 08 | SIK2 | salt inducible kinase 2 | Cytoplasm | kinase |
| CDDP 08 | STK17B | serine/threonine kinase 17b | Nucleus | kinase |
| CDDP 08 | STK3 | serine/threonine kinase 3 | Cytoplasm | kinase |
| CDDP 08 | STK39 | serine/threonine kinase 39 | Nucleus | kinase |
| CDDP 08 | SYK | spleen associated tyrosine kinase | Cytoplasm | kinase |
| CDDP 08 | TAOK1 | TAO kinase 1 | Cytoplasm | kinase |
| CDDP 08 | TAOK3 | TAO kinase 3 | Cytoplasm | kinase |
| CDDP 08 | TESK1 | testis associated actin remodelling kinase 1 | Nucleus | kinase |
| CDDP 08 | TNK2 | tyrosine kinase non receptor 2 | Cytoplasm | kinase |
| CDDP 08 | TSSK1B | testis specific serine kinase 1B | Cytoplasm | kinase |
| CDDP 08 | TSSK2 | testis specific serine kinase 2 | Cytoplasm | kinase |
| CDDP 08 | TXK | TXK tyrosine kinase | Cytoplasm | kinase |
| CDDP 08 | TYRO3 | TYRO3 protein tyrosine kinase | Plasma Membrane | kinase |
| CDDP 08 | ULK1 | unc-51 like autophagy activating kinase 1 | Cytoplasm | kinase |
| CDDP 08 | ULK2 | unc-51 like autophagy activating kinase 2 | Cytoplasm | kinase |
| CDDP 08 | ULK3 | unc-51 like kinase 3 | Cytoplasm | kinase |
| CDDP 08 | FLT4 | fms related tyrosine kinase 4 | Plasma Membrane | transmembrane receptor |
| CDDP 08 | YES1 | YES proto-oncogene 1, Src family tyrosine kinase | Cytoplasm | kinase |
| CDDP 08 | ZAP70 | zeta chain of T cell receptor associated protein kinase 70 | Plasma Membrane | kinase |
| CDDP 09 | AAK1 | AP2 associated kinase 1 | Cytoplasm | kinase |
| CDDP 09 | AKT2 | AKT serine/threonine kinase 2 | Cytoplasm | kinase |
| CDDP 09 | ACVR1 | activin A receptor type 1 | Plasma Membrane | kinase |
| CDDP 09 | ACVR1B | activin A receptor type 1B | Plasma Membrane | kinase |
| CDDP 09 | ANKK1 | ankyrin repeat and kinase domain containing 1 | Cytoplasm | kinase |
| CDDP 09 | AURKA | aurora kinase A | Nucleus | kinase |
| CDDP 09 | AURKB | aurora kinase B | Nucleus | kinase |
| CDDP 09 | AURKC | aurora kinase C | Nucleus | kinase |
| CDDP 09 | AXL | AXL receptor tyrosine kinase | Plasma Membrane | kinase |
| CDDP 09 | BLK | BLK proto-oncogene, Src family tyrosine kinase | Cytoplasm | kinase |
| CDDP 09 | BMP2K | BMP2 inducible kinase | Nucleus | kinase |
| CDDP 09 | BMPR1B | bone morphogenetic protein receptor type 1B | Plasma Membrane | kinase |
| CDDP 09 | BMX | BMX non-receptor tyrosine kinase | Cytoplasm | kinase |
| CDDP 09 | BRSK1 | BR serine/threonine kinase 1 | Cytoplasm | kinase |
| CDDP 09 | BTK | Bruton tyrosine kinase | Cytoplasm | kinase |
| CDDP 09 | CAMK1D | calcium/calmodulin dependent protein kinase ID | Cytoplasm | kinase |
| CDDP 09 | CAMK1G | calcium/calmodulin dependent protein kinase IG | Cytoplasm | kinase |
| CDDP 09 | CAMK2A | calcium/calmodulin dependent protein kinase II alpha | Cytoplasm | kinase |
| CDDP 09 | CAMK2B | calcium/calmodulin dependent protein kinase II beta | Cytoplasm | kinase |
| CDDP 09 | CAMK2D | calcium/calmodulin dependent protein kinase II delta | Cytoplasm | kinase |
| CDDP 09 | CAMK2G | calcium/calmodulin dependent protein kinase II gamma | Cytoplasm | kinase |
| CDDP 09 | CAMK4 | calcium/calmodulin dependent protein kinase IV | Nucleus | kinase |
| CDDP 09 | CAMKK1 | calcium/calmodulin dependent protein kinase kinase 1 | Cytoplasm | kinase |
| CDDP 09 | CAMKK2 | calcium/calmodulin dependent protein kinase kinase 2 | Cytoplasm | kinase |
| CDDP 09 | CDC42BPB | CDC42 binding protein kinase beta | Cytoplasm | kinase |
| CDDP 09 | CDC7 | cell division cycle 7 | Nucleus | kinase |
| CDDP 09 | CDK3 | cyclin dependent kinase 3 | Other | kinase |
| CDDP 09 | CDK4 | cyclin dependent kinase 4 | Nucleus | kinase |
| CDDP 09 | CDK5 | cyclin dependent kinase 5 | Nucleus | kinase |
| CDDP 09 | CDK6 | cyclin dependent kinase 6 | Nucleus | kinase |
| CDDP 09 | CDK7 | cyclin dependent kinase 7 | Nucleus | kinase |
| CDDP 09 | CDK8 | cyclin dependent kinase 8 | Nucleus | kinase |
| CDDP 09 | CDK9 | cyclin dependent kinase 9 | Nucleus | kinase |
| CDDP 09 | CDKL2 | cyclin dependent kinase like 2 | Nucleus | kinase |
| CDDP 09 | CDKL3 | cyclin dependent kinase like 3 | Cytoplasm | kinase |
| CDDP 09 | CDKL5 | cyclin dependent kinase like 5 | Nucleus | kinase |
| CDDP 09 | CHUK | component of inhibitor of nuclear factor kappa B kinase complex | Cytoplasm | kinase |
| CDDP 09 | CIT | citron rho-interacting serine/threonine kinase | Cytoplasm | kinase |
| CDDP 09 | CLK1 | CDC like kinase 1 | Nucleus | kinase |
| CDDP 09 | CLK4 | CDC like kinase 4 | Nucleus | kinase |
| CDDP 09 | CSK | C-terminal Src kinase | Cytoplasm | kinase |
| CDDP 09 | CSNK1A1 | casein kinase 1 alpha 1 | Cytoplasm | kinase |
| CDDP 09 | CSNK1A1L | casein kinase 1 alpha 1 like | Cytoplasm | kinase |
| CDDP 09 | CSNK1E | casein kinase 1 epsilon | Cytoplasm | kinase |
| CDDP 09 | CSNK1G2 | casein kinase 1 gamma 2 | Cytoplasm | kinase |
| CDDP 09 | CSNK1G3 | casein kinase 1 gamma 3 | Cytoplasm | kinase |
| CDDP 09 | CSNK2A1 | casein kinase 2 alpha 1 | Nucleus | kinase |
| CDDP 09 | CSNK2A2 | casein kinase 2 alpha 2 | Cytoplasm | kinase |
| CDDP 09 | CHEK1 | checkpoint kinase 1 | Nucleus | kinase |
| CDDP 09 | CHEK2 | checkpoint kinase 2 | Nucleus | kinase |
| CDDP 09 | DAPK1 | death associated protein kinase 1 | Cytoplasm | kinase |
| CDDP 09 | DAPK3 | death associated protein kinase 3 | Cytoplasm | kinase |
| CDDP 09 | DDR1 | discoidin domain receptor tyrosine kinase 1 | Plasma Membrane | kinase |
| CDDP 09 | DMPK | DM1 protein kinase | Cytoplasm | kinase |
| CDDP 09 | DYRK1A | dual specificity tyrosine phosphorylation regulated kinase 1A | Nucleus | kinase |
| CDDP 09 | DYRK1B | dual specificity tyrosine phosphorylation regulated kinase 1B | Nucleus | kinase |
| CDDP 09 | DYRK2 | dual specificity tyrosine phosphorylation regulated kinase 2 | Cytoplasm | kinase |
| CDDP 09 | DYRK3 | dual specificity tyrosine phosphorylation regulated kinase 3 | Nucleus | kinase |
| CDDP 09 | DYRK4 | dual specificity tyrosine phosphorylation regulated kinase 4 | Nucleus | kinase |
| CDDP 09 | EIF2AK2 | eukaryotic translation initiation factor 2 alpha kinase 2 | Cytoplasm | kinase |
| CDDP 09 | EPHA1 | EPH receptor A1 | Plasma Membrane | kinase |
| CDDP 09 | EPHA2 | EPH receptor A2 | Plasma Membrane | kinase |
| CDDP 09 | EPHA3 | EPH receptor A3 | Plasma Membrane | kinase |
| CDDP 09 | EPHA7 | EPH receptor A7 | Plasma Membrane | kinase |
| CDDP 09 | EPHA8 | EPH receptor A8 | Plasma Membrane | kinase |
| CDDP 09 | EPHB6 | EPH receptor B6 | Plasma Membrane | kinase |
| CDDP 09 | MAPK7 | mitogen-activated protein kinase 7 | Cytoplasm | kinase |
| CDDP 09 | MAPK15 | mitogen-activated protein kinase 15 | Cytoplasm | kinase |
| CDDP 09 | PTK2B | protein tyrosine kinase 2 beta | Cytoplasm | kinase |
| CDDP 09 | FER | FER tyrosine kinase | Cytoplasm | kinase |
| CDDP 09 | FES | FES proto-oncogene, tyrosine kinase | Cytoplasm | kinase |
| CDDP 09 | FGFR1 | fibroblast growth factor receptor 1 | Plasma Membrane | kinase |
| CDDP 09 | FGFR2 | fibroblast growth factor receptor 2 | Plasma Membrane | kinase |
| CDDP 09 | FGFR3 | fibroblast growth factor receptor 3 | Plasma Membrane | kinase |
| CDDP 09 | FGR | FGR proto-oncogene, Src family tyrosine kinase | Nucleus | kinase |
| CDDP 09 | FLT3 | fms related tyrosine kinase 3 | Plasma Membrane | kinase |
| CDDP 09 | FYN | FYN proto-oncogene, Src family tyrosine kinase | Plasma Membrane | kinase |
| CDDP 09 | GAK | cyclin G associated kinase | Nucleus | kinase |
| CDDP 09 | GRK4 | G protein-coupled receptor kinase 4 | Plasma Membrane | kinase |
| CDDP 09 | GRK5 | G protein-coupled receptor kinase 5 | Plasma Membrane | kinase |
| CDDP 09 | GRK7 | G protein-coupled receptor kinase 7 | Cytoplasm | kinase |
| CDDP 09 | GSK3A | glycogen synthase kinase 3 alpha | Nucleus | kinase |
| CDDP 09 | GSK3B | glycogen synthase kinase 3 beta | Nucleus | kinase |
| CDDP 09 | HCK | HCK proto-oncogene, Src family tyrosine kinase | Cytoplasm | kinase |
| CDDP 09 | HIPK1 | homeodomain interacting protein kinase 1 | Nucleus | kinase |
| CDDP 09 | HIPK3 | homeodomain interacting protein kinase 3 | Nucleus | kinase |
| CDDP 09 | EIF2AK1 | eukaryotic translation initiation factor 2 alpha kinase 1 | Cytoplasm | kinase |
| CDDP 09 | CILK1 | ciliogenesis Associated Kinase 1 | Nucleus | kinase |
| CDDP 09 | IKBKE | inhibitor of nuclear factor kappa B kinase subunit epsilon | Cytoplasm | kinase |
| CDDP 09 | INSR | insulin receptor | Plasma Membrane | kinase |
| CDDP 09 | IRAK1 | interleukin 1 receptor associated kinase 1 | Plasma Membrane | kinase |
| CDDP 09 | ITK | IL2 inducible T cell kinase | Cytoplasm | kinase |
| CDDP 09 | JAK3 | Janus kinase 3 | Cytoplasm | kinase |
| CDDP 09 | MAPK8 | mitogen-activated protein kinase 8 | Cytoplasm | kinase |
| CDDP 09 | MAPK9 | mitogen-activated protein kinase 9 | Cytoplasm | kinase |
| CDDP 09 | MAPK10 | mitogen-activated protein kinase 10 | Cytoplasm | kinase |
| CDDP 09 | LATS1 | large tumor suppressor kinase 1 | Nucleus | kinase |
| CDDP 09 | LIMK1 | LIM domain kinase 1 | Cytoplasm | kinase |
| CDDP 09 | LRRK2 | leucine rich repeat kinase 2 | Cytoplasm | kinase |
| CDDP 09 | LTK | leukocyte receptor tyrosine kinase | Plasma Membrane | kinase |
| CDDP 09 | LYN | LYN proto-oncogene, Src family tyrosine kinase | Cytoplasm | kinase |
| CDDP 09 | MAP3K10 | mitogen-activated protein kinase kinase kinase 10 | Cytoplasm | kinase |
| CDDP 09 | MAP3K11 | mitogen-activated protein kinase kinase kinase 11 | Cytoplasm | kinase |
| CDDP 09 | MAP3K13 | mitogen-activated protein kinase kinase kinase 13 | Cytoplasm | kinase |
| CDDP 09 | MAP3K15 | mitogen-activated protein kinase kinase kinase 15 | Other | other |
| CDDP 09 | MAP3K2 | mitogen-activated protein kinase kinase kinase 2 | Cytoplasm | kinase |
| CDDP 09 | MAP3K3 | mitogen-activated protein kinase kinase kinase 3 | Cytoplasm | kinase |
| CDDP 09 | MAP3K5 | mitogen-activated protein kinase kinase kinase 5 | Cytoplasm | kinase |
| CDDP 09 | MAP3K6 | mitogen-activated protein kinase kinase kinase 6 | Other | kinase |
| CDDP 09 | MAP3K9 | mitogen-activated protein kinase kinase kinase 9 | Cytoplasm | kinase |
| CDDP 09 | MAP4K1 | mitogen-activated protein kinase kinase kinase kinase 1 | Cytoplasm | kinase |
| CDDP 09 | MAP4K2 | mitogen-activated protein kinase kinase kinase kinase 2 | Cytoplasm | kinase |
| CDDP 09 | MAP4K3 | mitogen-activated protein kinase kinase kinase kinase 3 | Other | kinase |
| CDDP 09 | MAP4K4 | mitogen-activated protein kinase kinase kinase kinase 4 | Cytoplasm | kinase |
| CDDP 09 | MAP4K5 | mitogen-activated protein kinase kinase kinase kinase 5 | Cytoplasm | kinase |
| CDDP 09 | MAPKAPK3 | MAPK activated protein kinase 3 | Nucleus | kinase |
| CDDP 09 | MAPKAPK5 | MAPK activated protein kinase 5 | Cytoplasm | kinase |
| CDDP 09 | MARK1 | microtubule affinity regulating kinase 1 | Cytoplasm | kinase |
| CDDP 09 | MARK2 | microtubule affinity regulating kinase 2 | Cytoplasm | kinase |
| CDDP 09 | MARK3 | microtubule affinity regulating kinase 3 | Cytoplasm | kinase |
| CDDP 09 | MARK4 | microtubule affinity regulating kinase 4 | Cytoplasm | kinase |
| CDDP 09 | MAST1 | microtubule associated serine/threonine kinase 1 | Cytoplasm | kinase |
| CDDP 09 | MATK | megakaryocyte-associated tyrosine kinase | Cytoplasm | kinase |
| CDDP 09 | MAP2K1 | mitogen-activated protein kinase kinase 1 | Cytoplasm | kinase |
| CDDP 09 | MAP2K2 | mitogen-activated protein kinase kinase 2 | Cytoplasm | kinase |
| CDDP 09 | MELK | maternal embryonic leucine zipper kinase | Cytoplasm | kinase |
| CDDP 09 | MET | MET proto-oncogene, receptor tyrosine kinase | Plasma Membrane | kinase |
| CDDP 09 | MINK1 | misshapen like kinase 1 | Cytoplasm | kinase |
| CDDP 09 | MAP2K3 | mitogen-activated protein kinase kinase 3 | Cytoplasm | kinase |
| CDDP 09 | MAP2K4 | mitogen-activated protein kinase kinase 4 | Cytoplasm | kinase |
| CDDP 09 | MAP2K6 | mitogen-activated protein kinase kinase 6 | Cytoplasm | kinase |
| CDDP 09 | MKNK2 | MAPK interacting serine/threonine kinase 2 | Cytoplasm | kinase |
| CDDP 09 | TTK | TTK protein kinase | Nucleus | kinase |
| CDDP 09 | RPS6KA4 | ribosomal protein S6 kinase A4 | Cytoplasm | kinase |
| CDDP 09 | SRPK3 | SRSF protein kinase 3 | Cytoplasm | kinase |
| CDDP 09 | MST1R | macrophage stimulating 1 receptor | Plasma Membrane | kinase |
| CDDP 09 | STK26 | serine/threonine kinase 26 | Nucleus | kinase |
| CDDP 09 | MYLK | myosin light chain kinase | Cytoplasm | kinase |
| CDDP 09 | MYLK2 | myosin light chain kinase 2 | Cytoplasm | kinase |
| CDDP 09 | MYO3A | myosin IIIA | Cytoplasm | kinase |
| CDDP 09 | MYO3B | myosin IIIB | Plasma Membrane | kinase |
| CDDP 09 | NEK2 | NIMA related kinase 2 | Cytoplasm | kinase |
| CDDP 09 | NEK4 | NIMA related kinase 4 | Nucleus | kinase |
| CDDP 09 | NEK5 | NIMA related kinase 5 | Other | kinase |
| CDDP 09 | NEK7 | NIMA related kinase 7 | Nucleus | kinase |
| CDDP 09 | NLK | nemo like kinase | Nucleus | kinase |
| CDDP 09 | NTRK1 | neurotrophic receptor tyrosine kinase 1 | Plasma Membrane | kinase |
| CDDP 09 | NTRK3 | neurotrophic receptor tyrosine kinase 3 | Plasma Membrane | kinase |
| CDDP 09 | NUAK2 | NUAK family kinase 2 | Other | kinase |
| CDDP 09 | OXSR1 | oxidative stress responsive kinase 1 | Nucleus | kinase |
| CDDP 09 | PAK2 | p21 (RAC1) activated kinase 2 | Cytoplasm | kinase |
| CDDP 09 | PAK3 | p21 (RAC1) activated kinase 3 | Cytoplasm | kinase |
| CDDP 09 | PAK6 | p21 (RAC1) activated kinase 6 | Cytoplasm | kinase |
| CDDP 09 | PAK5 | p21 (RAC1) activated kinase 5 | Nucleus | kinase |
| CDDP 09 | PASK | PAS domain containing serine/threonine kinase | Cytoplasm | kinase |
| CDDP 09 | CDK17 | cyclin dependent kinase 17 | Cytoplasm | kinase |
| CDDP 09 | PDPK1 | 3-phosphoinositide dependent protein kinase 1 | Cytoplasm | kinase |
| CDDP 09 | EIF2AK3 | eukaryotic translation initiation factor 2 alpha kinase 3 | Cytoplasm | kinase |
| CDDP 09 | PHKG1 | phosphorylase kinase catalytic subunit gamma 1 | Cytoplasm | kinase |
| CDDP 09 | PHKG2 | phosphorylase kinase catalytic subunit gamma 2 | Cytoplasm | kinase |
| CDDP 09 | PIM1 | Pim-1 proto-oncogene, serine/threonine kinase | Cytoplasm | kinase |
| CDDP 09 | PIM3 | Pim-3 proto-oncogene, serine/threonine kinase | Cytoplasm | kinase |
| CDDP 09 | PRKCB | protein kinase C beta | Cytoplasm | kinase |
| CDDP 09 | PRKCI | protein kinase C iota | Cytoplasm | kinase |
| CDDP 09 | PRKCQ | protein kinase C theta | Cytoplasm | kinase |
| CDDP 09 | PKD1 | polycystin 1, transient receptor potential channel interacting | Plasma Membrane | kinase |
| CDDP 09 | PKD2 | polycystin 2, transient receptor potential cation channel | Plasma Membrane | kinase |
| CDDP 09 | PKD3 | polycystic Kidney Disease 3 (Autosomal Dominant) | Plasma Membrane | kinase |
| CDDP 09 | PKN1 | protein kinase N1 | Cytoplasm | kinase |
| CDDP 09 | PKN2 | protein kinase N2 | Cytoplasm | kinase |
| CDDP 09 | PLK1 | polo like kinase 1 | Nucleus | kinase |
| CDDP 09 | PLK2 | polo like kinase 2 | Nucleus | kinase |
| CDDP 09 | PLK3 | polo like kinase 3 | Nucleus | kinase |
| CDDP 09 | PLK4 | polo like kinase 4 | Cytoplasm | kinase |
| CDDP 09 | PRKAA1 | protein kinase AMP-activated catalytic subunit alpha 1 | Cytoplasm | kinase |
| CDDP 09 | PRKAA2 | protein kinase AMP-activated catalytic subunit alpha 2 | Cytoplasm | kinase |
| CDDP 09 | PRKX | protein kinase X-linked | Cytoplasm | kinase |
| CDDP 09 | SIK3 | SIK family kinase 3 | Cytoplasm | kinase |
| CDDP 09 | RAF1 | Raf-1 proto-oncogene, serine/threonine kinase | Cytoplasm | kinase |
| CDDP 09 | RET | ret proto-oncogene | Plasma Membrane | kinase |
| CDDP 09 | RIPK2 | receptor interacting serine/threonine kinase 2 | Plasma Membrane | kinase |
| CDDP 09 | RPS6KB1 | ribosomal protein S6 kinase B1 | Cytoplasm | kinase |
| CDDP 09 | SGK1 | serum/glucocorticoid regulated kinase 1 | Cytoplasm | kinase |
| CDDP 09 | SLK | STE20 like kinase | Nucleus | kinase |
| CDDP 09 | SIK2 | salt inducible kinase 2 | Cytoplasm | kinase |
| CDDP 09 | SRMS | src-related kinase lacking C-terminal regulatory tyrosine and N-terminal myristylation sites | Cytoplasm | kinase |
| CDDP 09 | SRPK1 | SRSF protein kinase 1 | Nucleus | kinase |
| CDDP 09 | STK10 | serine/threonine kinase 10 | Cytoplasm | kinase |
| CDDP 09 | STK11 | serine/threonine kinase 11 | Cytoplasm | kinase |
| CDDP 09 | STK16 | serine/threonine kinase 16 | Cytoplasm | kinase |
| CDDP 09 | STK17A | serine/threonine kinase 17a | Nucleus | kinase |
| CDDP 09 | STK17B | serine/threonine kinase 17b | Nucleus | kinase |
| CDDP 09 | STK24 | serine/threonine kinase 24 | Cytoplasm | kinase |
| CDDP 09 | STK25 | serine/threonine kinase 25 | Cytoplasm | kinase |
| CDDP 09 | STK3 | serine/threonine kinase 3 | Cytoplasm | kinase |
| CDDP 09 | STK33 | serine/threonine kinase 33 | Cytoplasm | kinase |
| CDDP 09 | STK35 | serine/threonine kinase 35 | Cytoplasm | kinase |
| CDDP 09 | STK38L | serine/threonine kinase 38 like | Cytoplasm | kinase |
| CDDP 09 | STK39 | serine/threonine kinase 39 | Nucleus | kinase |
| CDDP 09 | STK4 | serine/threonine kinase 4 | Cytoplasm | kinase |
| CDDP 09 | SYK | spleen associated tyrosine kinase | Cytoplasm | kinase |
| CDDP 09 | MYLK4 | myosin light chain kinase family member 4 | Cytoplasm | kinase |
| CDDP 09 | SBK3 | SH3 domain binding kinase family member 3 | Other | other |
| CDDP 09 | TAOK1 | TAO kinase 1 | Cytoplasm | kinase |
| CDDP 09 | TAOK3 | TAO kinase 3 | Cytoplasm | kinase |
| CDDP 09 | TBK1 | TANK binding kinase 1 | Cytoplasm | kinase |
| CDDP 09 | TESK1 | testis associated actin remodelling kinase 1 | Nucleus | kinase |
| CDDP 09 | TGFBR1 | transforming growth factor beta receptor 1 | Plasma Membrane | kinase |
| CDDP 09 | TIE1 | tyrosine kinase with immunoglobulin like and EGF like domains 1 | Plasma Membrane | kinase |
| CDDP 09 | TLK1 | tousled like kinase 1 | Nucleus | kinase |
| CDDP 09 | TLK2 | tousled like kinase 2 | Cytoplasm | kinase |
| CDDP 09 | TNIK | TRAF2 and NCK interacting kinase | Plasma Membrane | kinase |
| CDDP 09 | TNK1 | tyrosine kinase non receptor 1 | Cytoplasm | kinase |
| CDDP 09 | TNK2 | tyrosine kinase non receptor 2 | Cytoplasm | kinase |
| CDDP 09 | TNNI3K | TNNI3 interacting kinase | Cytoplasm | kinase |
| CDDP 09 | TSSK1B | testis specific serine kinase 1B | Cytoplasm | kinase |
| CDDP 09 | TSSK2 | testis specific serine kinase 2 | Cytoplasm | kinase |
| CDDP 09 | TXK | TXK tyrosine kinase | Cytoplasm | kinase |
| CDDP 09 | TYK2 | tyrosine kinase 2 | Plasma Membrane | kinase |
| CDDP 09 | TYRO3 | TYRO3 protein tyrosine kinase | Plasma Membrane | kinase |
| CDDP 09 | ULK1 | unc-51 like autophagy activating kinase 1 | Cytoplasm | kinase |
| CDDP 09 | ULK2 | unc-51 like autophagy activating kinase 2 | Cytoplasm | kinase |
| CDDP 09 | ULK3 | unc-51 like kinase 3 | Cytoplasm | kinase |
| CDDP 09 | FLT1 | fms related tyrosine kinase 1 | Plasma Membrane | kinase |
| CDDP 09 | KDR | kinase insert domain receptor | Plasma Membrane | kinase |
| CDDP 09 | FLT4 | fms related tyrosine kinase 4 | Plasma Membrane | transmembrane receptor |
| CDDP 09 | YES1 | YES proto-oncogene 1, Src family tyrosine kinase | Cytoplasm | kinase |
| CDDP 09 | MAP3K20 | mitogen-activated protein kinase kinase kinase 20 | Cytoplasm | kinase |
| CDDP 09 | ZAP70 | zeta chain of T cell receptor associated protein kinase 70 | Plasma Membrane | kinase |
| CDDP 10 | AAK1 | AP2 associated kinase 1 | Cytoplasm | kinase |
| CDDP 10 | AKT2 | AKT serine/threonine kinase 2 | Cytoplasm | kinase |
| CDDP 10 | ACVR1 | activin A receptor type 1 | Plasma Membrane | kinase |
| CDDP 10 | ACVR1B | activin A receptor type 1B | Plasma Membrane | kinase |
| CDDP 10 | ANKK1 | ankyrin repeat and kinase domain containing 1 | Cytoplasm | kinase |
| CDDP 10 | AURKA | aurora kinase A | Nucleus | kinase |
| CDDP 10 | AURKB | aurora kinase B | Nucleus | kinase |
| CDDP 10 | AURKC | aurora kinase C | Nucleus | kinase |
| CDDP 10 | AXL | AXL receptor tyrosine kinase | Plasma Membrane | kinase |
| CDDP 10 | BLK | BLK proto-oncogene, Src family tyrosine kinase | Cytoplasm | kinase |
| CDDP 10 | BMP2K | BMP2 inducible kinase | Nucleus | kinase |
| CDDP 10 | BMPR1B | bone morphogenetic protein receptor type 1B | Plasma Membrane | kinase |
| CDDP 10 | BMX | BMX non-receptor tyrosine kinase | Cytoplasm | kinase |
| CDDP 10 | BRSK1 | BR serine/threonine kinase 1 | Cytoplasm | kinase |
| CDDP 10 | BTK | Bruton tyrosine kinase | Cytoplasm | kinase |
| CDDP 10 | CAMK1D | calcium/calmodulin dependent protein kinase ID | Cytoplasm | kinase |
| CDDP 10 | CAMK1G | calcium/calmodulin dependent protein kinase IG | Cytoplasm | kinase |
| CDDP 10 | CAMK2A | calcium/calmodulin dependent protein kinase II alpha | Cytoplasm | kinase |
| CDDP 10 | CAMK2B | calcium/calmodulin dependent protein kinase II beta | Cytoplasm | kinase |
| CDDP 10 | CAMK2D | calcium/calmodulin dependent protein kinase II delta | Cytoplasm | kinase |
| CDDP 10 | CAMK2G | calcium/calmodulin dependent protein kinase II gamma | Cytoplasm | kinase |
| CDDP 10 | CAMK4 | calcium/calmodulin dependent protein kinase IV | Nucleus | kinase |
| CDDP 10 | CAMKK1 | calcium/calmodulin dependent protein kinase kinase 1 | Cytoplasm | kinase |
| CDDP 10 | CAMKK2 | calcium/calmodulin dependent protein kinase kinase 2 | Cytoplasm | kinase |
| CDDP 10 | CDC42BPB | CDC42 binding protein kinase beta | Cytoplasm | kinase |
| CDDP 10 | CDC7 | cell division cycle 7 | Nucleus | kinase |
| CDDP 10 | CDK3 | cyclin dependent kinase 3 | Other | kinase |
| CDDP 10 | CDK4 | cyclin dependent kinase 4 | Nucleus | kinase |
| CDDP 10 | CDK5 | cyclin dependent kinase 5 | Nucleus | kinase |
| CDDP 10 | CDK6 | cyclin dependent kinase 6 | Nucleus | kinase |
| CDDP 10 | CDK7 | cyclin dependent kinase 7 | Nucleus | kinase |
| CDDP 10 | CDK8 | cyclin dependent kinase 8 | Nucleus | kinase |
| CDDP 10 | CDK9 | cyclin dependent kinase 9 | Nucleus | kinase |
| CDDP 10 | CDKL2 | cyclin dependent kinase like 2 | Nucleus | kinase |
| CDDP 10 | CDKL3 | cyclin dependent kinase like 3 | Cytoplasm | kinase |
| CDDP 10 | CDKL5 | cyclin dependent kinase like 5 | Nucleus | kinase |
| CDDP 10 | CHUK | component of inhibitor of nuclear factor kappa B kinase complex | Cytoplasm | kinase |
| CDDP 10 | CIT | citron rho-interacting serine/threonine kinase | Cytoplasm | kinase |
| CDDP 10 | CLK1 | CDC like kinase 1 | Nucleus | kinase |
| CDDP 10 | CLK4 | CDC like kinase 4 | Nucleus | kinase |
| CDDP 10 | CSK | C-terminal Src kinase | Cytoplasm | kinase |
| CDDP 10 | CSNK1A1 | casein kinase 1 alpha 1 | Cytoplasm | kinase |
| CDDP 10 | CSNK1A1L | casein kinase 1 alpha 1 like | Cytoplasm | kinase |
| CDDP 10 | CSNK1E | casein kinase 1 epsilon | Cytoplasm | kinase |
| CDDP 10 | CSNK1G2 | casein kinase 1 gamma 2 | Cytoplasm | kinase |
| CDDP 10 | CSNK1G3 | casein kinase 1 gamma 3 | Cytoplasm | kinase |
| CDDP 10 | CSNK2A1 | casein kinase 2 alpha 1 | Nucleus | kinase |
| CDDP 10 | CSNK2A2 | casein kinase 2 alpha 2 | Cytoplasm | kinase |
| CDDP 10 | CHEK1 | checkpoint kinase 1 | Nucleus | kinase |
| CDDP 10 | CHEK2 | checkpoint kinase 2 | Nucleus | kinase |
| CDDP 10 | DAPK1 | death associated protein kinase 1 | Cytoplasm | kinase |
| CDDP 10 | DAPK3 | death associated protein kinase 3 | Cytoplasm | kinase |
| CDDP 10 | DDR1 | discoidin domain receptor tyrosine kinase 1 | Plasma Membrane | kinase |
| CDDP 10 | DMPK | DM1 protein kinase | Cytoplasm | kinase |
| CDDP 10 | DYRK1A | dual specificity tyrosine phosphorylation regulated kinase 1A | Nucleus | kinase |
| CDDP 10 | DYRK1B | dual specificity tyrosine phosphorylation regulated kinase 1B | Nucleus | kinase |
| CDDP 10 | DYRK2 | dual specificity tyrosine phosphorylation regulated kinase 2 | Cytoplasm | kinase |
| CDDP 10 | DYRK3 | dual specificity tyrosine phosphorylation regulated kinase 3 | Nucleus | kinase |
| CDDP 10 | DYRK4 | dual specificity tyrosine phosphorylation regulated kinase 4 | Nucleus | kinase |
| CDDP 10 | EIF2AK2 | eukaryotic translation initiation factor 2 alpha kinase 2 | Cytoplasm | kinase |
| CDDP 10 | EPHA1 | EPH receptor A1 | Plasma Membrane | kinase |
| CDDP 10 | EPHA2 | EPH receptor A2 | Plasma Membrane | kinase |
| CDDP 10 | EPHA3 | EPH receptor A3 | Plasma Membrane | kinase |
| CDDP 10 | EPHA7 | EPH receptor A7 | Plasma Membrane | kinase |
| CDDP 10 | EPHA8 | EPH receptor A8 | Plasma Membrane | kinase |
| CDDP 10 | EPHB6 | EPH receptor B6 | Plasma Membrane | kinase |
| CDDP 10 | MAPK7 | mitogen-activated protein kinase 7 | Cytoplasm | kinase |
| CDDP 10 | MAPK15 | mitogen-activated protein kinase 15 | Cytoplasm | kinase |
| CDDP 10 | PTK2B | protein tyrosine kinase 2 beta | Cytoplasm | kinase |
| CDDP 10 | FER | FER tyrosine kinase | Cytoplasm | kinase |
| CDDP 10 | FES | FES proto-oncogene, tyrosine kinase | Cytoplasm | kinase |
| CDDP 10 | FGFR1 | fibroblast growth factor receptor 1 | Plasma Membrane | kinase |
| CDDP 10 | FGFR2 | fibroblast growth factor receptor 2 | Plasma Membrane | kinase |
| CDDP 10 | FGFR3 | fibroblast growth factor receptor 3 | Plasma Membrane | kinase |
| CDDP 10 | FGR | FGR proto-oncogene, Src family tyrosine kinase | Nucleus | kinase |
| CDDP 10 | FLT3 | fms related tyrosine kinase 3 | Plasma Membrane | kinase |
| CDDP 10 | FYN | FYN proto-oncogene, Src family tyrosine kinase | Plasma Membrane | kinase |
| CDDP 10 | GAK | cyclin G associated kinase | Nucleus | kinase |
| CDDP 10 | GRK4 | G protein-coupled receptor kinase 4 | Plasma Membrane | kinase |
| CDDP 10 | GRK5 | G protein-coupled receptor kinase 5 | Plasma Membrane | kinase |
| CDDP 10 | GRK7 | G protein-coupled receptor kinase 7 | Cytoplasm | kinase |
| CDDP 10 | GSK3A | glycogen synthase kinase 3 alpha | Nucleus | kinase |
| CDDP 10 | GSK3B | glycogen synthase kinase 3 beta | Nucleus | kinase |
| CDDP 10 | HCK | HCK proto-oncogene, Src family tyrosine kinase | Cytoplasm | kinase |
| CDDP 10 | HIPK1 | homeodomain interacting protein kinase 1 | Nucleus | kinase |
| CDDP 10 | HIPK3 | homeodomain interacting protein kinase 3 | Nucleus | kinase |
| CDDP 10 | EIF2AK1 | eukaryotic translation initiation factor 2 alpha kinase 1 | Cytoplasm | kinase |
| CDDP 10 | CILK1 | ciliogenesis Associated Kinase 1 | Nucleus | kinase |
| CDDP 10 | IKBKE | inhibitor of nuclear factor kappa B kinase subunit epsilon | Cytoplasm | kinase |
| CDDP 10 | INSR | insulin receptor | Plasma Membrane | kinase |
| CDDP 10 | IRAK1 | interleukin 1 receptor associated kinase 1 | Plasma Membrane | kinase |
| CDDP 10 | ITK | IL2 inducible T cell kinase | Cytoplasm | kinase |
| CDDP 10 | JAK3 | Janus kinase 3 | Cytoplasm | kinase |
| CDDP 10 | MAPK8 | mitogen-activated protein kinase 8 | Cytoplasm | kinase |
| CDDP 10 | MAPK9 | mitogen-activated protein kinase 9 | Cytoplasm | kinase |
| CDDP 10 | MAPK10 | mitogen-activated protein kinase 10 | Cytoplasm | kinase |
| CDDP 10 | LATS1 | large tumor suppressor kinase 1 | Nucleus | kinase |
| CDDP 10 | LIMK1 | LIM domain kinase 1 | Cytoplasm | kinase |
| CDDP 10 | LRRK2 | leucine rich repeat kinase 2 | Cytoplasm | kinase |
| CDDP 10 | LTK | leukocyte receptor tyrosine kinase | Plasma Membrane | kinase |
| CDDP 10 | LYN | LYN proto-oncogene, Src family tyrosine kinase | Cytoplasm | kinase |
| CDDP 10 | MAP3K10 | mitogen-activated protein kinase kinase kinase 10 | Cytoplasm | kinase |
| CDDP 10 | MAP3K11 | mitogen-activated protein kinase kinase kinase 11 | Cytoplasm | kinase |
| CDDP 10 | MAP3K13 | mitogen-activated protein kinase kinase kinase 13 | Cytoplasm | kinase |
| CDDP 10 | MAP3K15 | mitogen-activated protein kinase kinase kinase 15 | Other | other |
| CDDP 10 | MAP3K2 | mitogen-activated protein kinase kinase kinase 2 | Cytoplasm | kinase |
| CDDP 10 | MAP3K3 | mitogen-activated protein kinase kinase kinase 3 | Cytoplasm | kinase |
| CDDP 10 | MAP3K5 | mitogen-activated protein kinase kinase kinase 5 | Cytoplasm | kinase |
| CDDP 10 | MAP3K6 | mitogen-activated protein kinase kinase kinase 6 | Other | kinase |
| CDDP 10 | MAP3K9 | mitogen-activated protein kinase kinase kinase 9 | Cytoplasm | kinase |
| CDDP 10 | MAP4K1 | mitogen-activated protein kinase kinase kinase kinase 1 | Cytoplasm | kinase |
| CDDP 10 | MAP4K2 | mitogen-activated protein kinase kinase kinase kinase 2 | Cytoplasm | kinase |
| CDDP 10 | MAP4K3 | mitogen-activated protein kinase kinase kinase kinase 3 | Other | kinase |
| CDDP 10 | MAP4K4 | mitogen-activated protein kinase kinase kinase kinase 4 | Cytoplasm | kinase |
| CDDP 10 | MAP4K5 | mitogen-activated protein kinase kinase kinase kinase 5 | Cytoplasm | kinase |
| CDDP 10 | MAPKAPK3 | MAPK activated protein kinase 3 | Nucleus | kinase |
| CDDP 10 | MAPKAPK5 | MAPK activated protein kinase 5 | Cytoplasm | kinase |
| CDDP 10 | MARK1 | microtubule affinity regulating kinase 1 | Cytoplasm | kinase |
| CDDP 10 | MARK2 | microtubule affinity regulating kinase 2 | Cytoplasm | kinase |
| CDDP 10 | MARK3 | microtubule affinity regulating kinase 3 | Cytoplasm | kinase |
| CDDP 10 | MARK4 | microtubule affinity regulating kinase 4 | Cytoplasm | kinase |
| CDDP 10 | MAST1 | microtubule associated serine/threonine kinase 1 | Cytoplasm | kinase |
| CDDP 10 | MATK | megakaryocyte-associated tyrosine kinase | Cytoplasm | kinase |
| CDDP 10 | MAP2K1 | mitogen-activated protein kinase kinase 1 | Cytoplasm | kinase |
| CDDP 10 | MAP2K2 | mitogen-activated protein kinase kinase 2 | Cytoplasm | kinase |
| CDDP 10 | MELK | maternal embryonic leucine zipper kinase | Cytoplasm | kinase |
| CDDP 10 | MET | MET proto-oncogene, receptor tyrosine kinase | Plasma Membrane | kinase |
| CDDP 10 | MINK1 | misshapen like kinase 1 | Cytoplasm | kinase |
| CDDP 10 | MAP2K3 | mitogen-activated protein kinase kinase 3 | Cytoplasm | kinase |
| CDDP 10 | MAP2K4 | mitogen-activated protein kinase kinase 4 | Cytoplasm | kinase |
| CDDP 10 | MAP2K6 | mitogen-activated protein kinase kinase 6 | Cytoplasm | kinase |
| CDDP 10 | MKNK2 | MAPK interacting serine/threonine kinase 2 | Cytoplasm | kinase |
| CDDP 10 | TTK | TTK protein kinase | Nucleus | kinase |
| CDDP 10 | RPS6KA4 | ribosomal protein S6 kinase A4 | Cytoplasm | kinase |
| CDDP 10 | SRPK3 | SRSF protein kinase 3 | Cytoplasm | kinase |
| CDDP 10 | MST1R | macrophage stimulating 1 receptor | Plasma Membrane | kinase |
| CDDP 10 | STK26 | serine/threonine kinase 26 | Nucleus | kinase |
| CDDP 10 | MYLK | myosin light chain kinase | Cytoplasm | kinase |
| CDDP 10 | MYLK2 | myosin light chain kinase 2 | Cytoplasm | kinase |
| CDDP 10 | MYO3A | myosin IIIA | Cytoplasm | kinase |
| CDDP 10 | MYO3B | myosin IIIB | Plasma Membrane | kinase |
| CDDP 10 | NEK2 | NIMA related kinase 2 | Cytoplasm | kinase |
| CDDP 10 | NEK4 | NIMA related kinase 4 | Nucleus | kinase |
| CDDP 10 | NEK5 | NIMA related kinase 5 | Other | kinase |
| CDDP 10 | NEK7 | NIMA related kinase 7 | Nucleus | kinase |
| CDDP 10 | NLK | nemo like kinase | Nucleus | kinase |
| CDDP 10 | NTRK1 | neurotrophic receptor tyrosine kinase 1 | Plasma Membrane | kinase |
| CDDP 10 | NTRK3 | neurotrophic receptor tyrosine kinase 3 | Plasma Membrane | kinase |
| CDDP 10 | NUAK2 | NUAK family kinase 2 | Other | kinase |
| CDDP 10 | OXSR1 | oxidative stress responsive kinase 1 | Nucleus | kinase |
| CDDP 10 | PAK2 | p21 (RAC1) activated kinase 2 | Cytoplasm | kinase |
| CDDP 10 | PAK3 | p21 (RAC1) activated kinase 3 | Cytoplasm | kinase |
| CDDP 10 | PAK6 | p21 (RAC1) activated kinase 6 | Cytoplasm | kinase |
| CDDP 10 | PAK5 | p21 (RAC1) activated kinase 5 | Nucleus | kinase |
| CDDP 10 | PASK | PAS domain containing serine/threonine kinase | Cytoplasm | kinase |
| CDDP 10 | CDK17 | cyclin dependent kinase 17 | Cytoplasm | kinase |
| CDDP 10 | PDPK1 | 3-phosphoinositide dependent protein kinase 1 | Cytoplasm | kinase |
| CDDP 10 | EIF2AK3 | eukaryotic translation initiation factor 2 alpha kinase 3 | Cytoplasm | kinase |
| CDDP 10 | PHKG1 | phosphorylase kinase catalytic subunit gamma 1 | Cytoplasm | kinase |
| CDDP 10 | PHKG2 | phosphorylase kinase catalytic subunit gamma 2 | Cytoplasm | kinase |
| CDDP 10 | PIM1 | Pim-1 proto-oncogene, serine/threonine kinase | Cytoplasm | kinase |
| CDDP 10 | PIM3 | Pim-3 proto-oncogene, serine/threonine kinase | Cytoplasm | kinase |
| CDDP 10 | PRKCB | protein kinase C beta | Cytoplasm | kinase |
| CDDP 10 | PRKCI | protein kinase C iota | Cytoplasm | kinase |
| CDDP 10 | PRKCQ | protein kinase C theta | Cytoplasm | kinase |
| CDDP 10 | PKD1 | polycystin 1, transient receptor potential channel interacting | Plasma Membrane | kinase |
| CDDP 10 | PKD2 | polycystin 2, transient receptor potential cation channel | Plasma Membrane | kinase |
| CDDP 10 | PKD3 | polycystic Kidney Disease 3 (Autosomal Dominant) | Plasma Membrane | kinase |
| CDDP 10 | PKN1 | protein kinase N1 | Cytoplasm | kinase |
| CDDP 10 | PKN2 | protein kinase N2 | Cytoplasm | kinase |
| CDDP 10 | PLK1 | polo like kinase 1 | Nucleus | kinase |
| CDDP 10 | PLK2 | polo like kinase 2 | Nucleus | kinase |
| CDDP 10 | PLK3 | polo like kinase 3 | Nucleus | kinase |
| CDDP 10 | PLK4 | polo like kinase 4 | Cytoplasm | kinase |
| CDDP 10 | PRKAA1 | protein kinase AMP-activated catalytic subunit alpha 1 | Cytoplasm | kinase |
| CDDP 10 | PRKAA2 | protein kinase AMP-activated catalytic subunit alpha 2 | Cytoplasm | kinase |
| CDDP 10 | PRKX | protein kinase X-linked | Cytoplasm | kinase |
| CDDP 10 | SIK3 | SIK family kinase 3 | Cytoplasm | kinase |
| CDDP 10 | RAF1 | Raf-1 proto-oncogene, serine/threonine kinase | Cytoplasm | kinase |
| CDDP 10 | RET | ret proto-oncogene | Plasma Membrane | kinase |
| CDDP 10 | RIPK2 | receptor interacting serine/threonine kinase 2 | Plasma Membrane | kinase |
| CDDP 10 | RPS6KB1 | ribosomal protein S6 kinase B1 | Cytoplasm | kinase |
| CDDP 10 | SGK1 | serum/glucocorticoid regulated kinase 1 | Cytoplasm | kinase |
| CDDP 10 | SLK | STE20 like kinase | Nucleus | kinase |
| CDDP 10 | SIK2 | salt inducible kinase 2 | Cytoplasm | kinase |
| CDDP 10 | SRMS | src-related kinase lacking C-terminal regulatory tyrosine and N-terminal myristylation sites | Cytoplasm | kinase |
| CDDP 10 | SRPK1 | SRSF protein kinase 1 | Nucleus | kinase |
| CDDP 10 | STK10 | serine/threonine kinase 10 | Cytoplasm | kinase |
| CDDP 10 | STK11 | serine/threonine kinase 11 | Cytoplasm | kinase |
| CDDP 10 | STK16 | serine/threonine kinase 16 | Cytoplasm | kinase |
| CDDP 10 | STK17A | serine/threonine kinase 17a | Nucleus | kinase |
| CDDP 10 | STK17B | serine/threonine kinase 17b | Nucleus | kinase |
| CDDP 10 | STK24 | serine/threonine kinase 24 | Cytoplasm | kinase |
| CDDP 10 | STK25 | serine/threonine kinase 25 | Cytoplasm | kinase |
| CDDP 10 | STK3 | serine/threonine kinase 3 | Cytoplasm | kinase |
| CDDP 10 | STK33 | serine/threonine kinase 33 | Cytoplasm | kinase |
| CDDP 10 | STK35 | serine/threonine kinase 35 | Cytoplasm | kinase |
| CDDP 10 | STK38L | serine/threonine kinase 38 like | Cytoplasm | kinase |
| CDDP 10 | STK39 | serine/threonine kinase 39 | Nucleus | kinase |
| CDDP 10 | STK4 | serine/threonine kinase 4 | Cytoplasm | kinase |
| CDDP 10 | SYK | spleen associated tyrosine kinase | Cytoplasm | kinase |
| CDDP 10 | MYLK4 | myosin light chain kinase family member 4 | Cytoplasm | kinase |
| CDDP 10 | SBK3 | SH3 domain binding kinase family member 3 | Other | other |
| CDDP 10 | TAOK1 | TAO kinase 1 | Cytoplasm | kinase |
| CDDP 10 | TAOK3 | TAO kinase 3 | Cytoplasm | kinase |
| CDDP 10 | TBK1 | TANK binding kinase 1 | Cytoplasm | kinase |
| CDDP 10 | TESK1 | testis associated actin remodelling kinase 1 | Nucleus | kinase |
| CDDP 10 | TGFBR1 | transforming growth factor beta receptor 1 | Plasma Membrane | kinase |
| CDDP 10 | TIE1 | tyrosine kinase with immunoglobulin like and EGF like domains 1 | Plasma Membrane | kinase |
| CDDP 10 | TLK1 | tousled like kinase 1 | Nucleus | kinase |
| CDDP 10 | TLK2 | tousled like kinase 2 | Cytoplasm | kinase |
| CDDP 10 | TNIK | TRAF2 and NCK interacting kinase | Plasma Membrane | kinase |
| CDDP 10 | TNK1 | tyrosine kinase non receptor 1 | Cytoplasm | kinase |
| CDDP 10 | TNK2 | tyrosine kinase non receptor 2 | Cytoplasm | kinase |
| CDDP 10 | TNNI3K | TNNI3 interacting kinase | Cytoplasm | kinase |
| CDDP 10 | TSSK1B | testis specific serine kinase 1B | Cytoplasm | kinase |
| CDDP 10 | TSSK2 | testis specific serine kinase 2 | Cytoplasm | kinase |
| CDDP 10 | TXK | TXK tyrosine kinase | Cytoplasm | kinase |
| CDDP 10 | TYK2 | tyrosine kinase 2 | Plasma Membrane | kinase |
| CDDP 10 | TYRO3 | TYRO3 protein tyrosine kinase | Plasma Membrane | kinase |
| CDDP 10 | ULK1 | unc-51 like autophagy activating kinase 1 | Cytoplasm | kinase |
| CDDP 10 | ULK2 | unc-51 like autophagy activating kinase 2 | Cytoplasm | kinase |
| CDDP 10 | ULK3 | unc-51 like kinase 3 | Cytoplasm | kinase |
| CDDP 10 | FLT1 | fms related tyrosine kinase 1 | Plasma Membrane | kinase |
| CDDP 10 | KDR | kinase insert domain receptor | Plasma Membrane | kinase |
| CDDP 10 | FLT4 | fms related tyrosine kinase 4 | Plasma Membrane | transmembrane receptor |
| CDDP 10 | YES1 | YES proto-oncogene 1, Src family tyrosine kinase | Cytoplasm | kinase |
| CDDP 10 | MAP3K20 | mitogen-activated protein kinase kinase kinase 20 | Cytoplasm | kinase |
| CDDP 10 | ZAP70 | zeta chain of T cell receptor associated protein kinase 70 | Plasma Membrane | kinase |
| CDDP 11 | ACVR1 | activin A receptor type 1 | Plasma Membrane | kinase |
| CDDP 11 | AURKB | aurora kinase B | Nucleus | kinase |
| CDDP 11 | AURKC | aurora kinase C | Nucleus | kinase |
| CDDP 11 | AXL | AXL receptor tyrosine kinase | Plasma Membrane | kinase |
| CDDP 11 | BRSK1 | BR serine/threonine kinase 1 | Cytoplasm | kinase |
| CDDP 11 | CAMK2B | calcium/calmodulin dependent protein kinase II beta | Cytoplasm | kinase |
| CDDP 11 | CAMK2D | calcium/calmodulin dependent protein kinase II delta | Cytoplasm | kinase |
| CDDP 11 | CAMKK1 | calcium/calmodulin dependent protein kinase kinase 1 | Cytoplasm | kinase |
| CDDP 11 | CAMKK2 | calcium/calmodulin dependent protein kinase kinase 2 | Cytoplasm | kinase |
| CDDP 11 | CDC7 | cell division cycle 7 | Nucleus | kinase |
| CDDP 11 | CDK3 | cyclin dependent kinase 3 | Other | kinase |
| CDDP 11 | CDK5 | cyclin dependent kinase 5 | Nucleus | kinase |
| CDDP 11 | CDK8 | cyclin dependent kinase 8 | Nucleus | kinase |
| CDDP 11 | CDK9 | cyclin dependent kinase 9 | Nucleus | kinase |
| CDDP 11 | CDKL2 | cyclin dependent kinase like 2 | Nucleus | kinase |
| CDDP 11 | CHUK | component of inhibitor of nuclear factor kappa B kinase complex | Cytoplasm | kinase |
| CDDP 11 | CLK2 | CDC like kinase 2 | Nucleus | kinase |
| CDDP 11 | CLK4 | CDC like kinase 4 | Nucleus | kinase |
| CDDP 11 | CSNK1A1 | casein kinase 1 alpha 1 | Cytoplasm | kinase |
| CDDP 11 | CSNK1D | casein kinase 1 delta | Cytoplasm | kinase |
| CDDP 11 | CSNK1E | casein kinase 1 epsilon | Cytoplasm | kinase |
| CDDP 11 | CSNK1G2 | casein kinase 1 gamma 2 | Cytoplasm | kinase |
| CDDP 11 | CSNK2A1 | casein kinase 2 alpha 1 | Nucleus | kinase |
| CDDP 11 | DAPK3 | death associated protein kinase 3 | Cytoplasm | kinase |
| CDDP 11 | DYRK1A | dual specificity tyrosine phosphorylation regulated kinase 1A | Nucleus | kinase |
| CDDP 11 | DYRK1B | dual specificity tyrosine phosphorylation regulated kinase 1B | Nucleus | kinase |
| CDDP 11 | DYRK3 | dual specificity tyrosine phosphorylation regulated kinase 3 | Nucleus | kinase |
| CDDP 11 | EEF2K | eukaryotic elongation factor 2 kinase | Cytoplasm | kinase |
| CDDP 11 | EPHA4 | EPH receptor A4 | Plasma Membrane | kinase |
| CDDP 11 | EPHA7 | EPH receptor A7 | Plasma Membrane | kinase |
| CDDP 11 | EPHB1 | EPH receptor B1 | Plasma Membrane | kinase |
| CDDP 11 | EPHB6 | EPH receptor B6 | Plasma Membrane | kinase |
| CDDP 11 | MAPK7 | mitogen-activated protein kinase 7 | Cytoplasm | kinase |
| CDDP 11 | FER | FER tyrosine kinase | Cytoplasm | kinase |
| CDDP 11 | FES | FES proto-oncogene, tyrosine kinase | Cytoplasm | kinase |
| CDDP 11 | GAK | cyclin G associated kinase | Nucleus | kinase |
| CDDP 11 | GRK1 | G protein-coupled receptor kinase 1 | Plasma Membrane | kinase |
| CDDP 11 | GRK4 | G protein-coupled receptor kinase 4 | Plasma Membrane | kinase |
| CDDP 11 | GRK5 | G protein-coupled receptor kinase 5 | Plasma Membrane | kinase |
| CDDP 11 | GRK7 | G protein-coupled receptor kinase 7 | Cytoplasm | kinase |
| CDDP 11 | HCK | HCK proto-oncogene, Src family tyrosine kinase | Cytoplasm | kinase |
| CDDP 11 | HIPK2 | homeodomain interacting protein kinase 2 | Nucleus | kinase |
| CDDP 11 | HIPK3 | homeodomain interacting protein kinase 3 | Nucleus | kinase |
| CDDP 11 | EIF2AK1 | eukaryotic translation initiation factor 2 alpha kinase 1 | Cytoplasm | kinase |
| CDDP 11 | CILK1 | ciliogenesis Associated Kinase 1 | Nucleus | kinase |
| CDDP 11 | IGF1R | insulin like growth factor 1 receptor | Plasma Membrane | transmembrane receptor |
| CDDP 11 | IKBKE | inhibitor of nuclear factor kappa B kinase subunit epsilon | Cytoplasm | kinase |
| CDDP 11 | INSR | insulin receptor | Plasma Membrane | kinase |
| CDDP 11 | IRAK1 | interleukin 1 receptor associated kinase 1 | Plasma Membrane | kinase |
| CDDP 11 | IRAK4 | interleukin 1 receptor associated kinase 4 | Cytoplasm | kinase |
| CDDP 11 | JAK2 | Janus kinase 2 | Cytoplasm | kinase |
| CDDP 11 | JAK3 | Janus kinase 3 | Cytoplasm | kinase |
| CDDP 11 | MAPK10 | mitogen-activated protein kinase 10 | Cytoplasm | kinase |
| CDDP 11 | LIMK1 | LIM domain kinase 1 | Cytoplasm | kinase |
| CDDP 11 | LRRK2 | leucine rich repeat kinase 2 | Cytoplasm | kinase |
| CDDP 11 | LTK | leukocyte receptor tyrosine kinase | Plasma Membrane | kinase |
| CDDP 11 | MAP3K10 | mitogen-activated protein kinase kinase kinase 10 | Cytoplasm | kinase |
| CDDP 11 | MAP3K13 | mitogen-activated protein kinase kinase kinase 13 | Cytoplasm | kinase |
| CDDP 11 | MAP3K2 | mitogen-activated protein kinase kinase kinase 2 | Cytoplasm | kinase |
| CDDP 11 | MAP3K3 | mitogen-activated protein kinase kinase kinase 3 | Cytoplasm | kinase |
| CDDP 11 | MAP4K3 | mitogen-activated protein kinase kinase kinase kinase 3 | Other | kinase |
| CDDP 11 | MAP4K4 | mitogen-activated protein kinase kinase kinase kinase 4 | Cytoplasm | kinase |
| CDDP 11 | MAP4K5 | mitogen-activated protein kinase kinase kinase kinase 5 | Cytoplasm | kinase |
| CDDP 11 | MARK3 | microtubule affinity regulating kinase 3 | Cytoplasm | kinase |
| CDDP 11 | MARK4 | microtubule affinity regulating kinase 4 | Cytoplasm | kinase |
| CDDP 11 | MAST1 | microtubule associated serine/threonine kinase 1 | Cytoplasm | kinase |
| CDDP 11 | MELK | maternal embryonic leucine zipper kinase | Cytoplasm | kinase |
| CDDP 11 | MAP2K4 | mitogen-activated protein kinase kinase 4 | Cytoplasm | kinase |
| CDDP 11 | MAP2K7 | mitogen-activated protein kinase kinase 7 | Cytoplasm | kinase |
| CDDP 11 | MKNK1 | MAPK interacting serine/threonine kinase 1 | Cytoplasm | kinase |
| CDDP 11 | MKNK2 | MAPK interacting serine/threonine kinase 2 | Cytoplasm | kinase |
| CDDP 11 | SRPK3 | SRSF protein kinase 3 | Cytoplasm | kinase |
| CDDP 11 | MST1R | macrophage stimulating 1 receptor | Plasma Membrane | kinase |
| CDDP 11 | MYLK2 | myosin light chain kinase 2 | Cytoplasm | kinase |
| CDDP 11 | NTRK3 | neurotrophic receptor tyrosine kinase 3 | Plasma Membrane | kinase |
| CDDP 11 | OXSR1 | oxidative stress responsive kinase 1 | Nucleus | kinase |
| CDDP 11 | PASK | PAS domain containing serine/threonine kinase | Cytoplasm | kinase |
| CDDP 11 | PBK | PDZ binding kinase | Cytoplasm | kinase |
| CDDP 11 | CDK16 | cyclin dependent kinase 16 | Cytoplasm | kinase |
| CDDP 11 | PHKG1 | phosphorylase kinase catalytic subunit gamma 1 | Cytoplasm | kinase |
| CDDP 11 | PHKG2 | phosphorylase kinase catalytic subunit gamma 2 | Cytoplasm | kinase |
| CDDP 11 | PIM2 | Pim-2 proto-oncogene, serine/threonine kinase | Nucleus | kinase |
| CDDP 11 | PIM3 | Pim-3 proto-oncogene, serine/threonine kinase | Cytoplasm | kinase |
| CDDP 11 | PKD2 | polycystin 2, transient receptor potential cation channel | Plasma Membrane | kinase |
| CDDP 11 | PKD3 | polycystic Kidney Disease 3 (Autosomal Dominant) | Plasma Membrane | kinase |
| CDDP 11 | PKN2 | protein kinase N2 | Cytoplasm | kinase |
| CDDP 11 | PLK2 | polo like kinase 2 | Nucleus | kinase |
| CDDP 11 | PLK4 | polo like kinase 4 | Cytoplasm | kinase |
| CDDP 11 | RET | ret proto-oncogene | Plasma Membrane | kinase |
| CDDP 11 | RIPK1 | receptor interacting serine/threonine kinase 1 | Plasma Membrane | kinase |
| CDDP 11 | ROS1 | ROS proto-oncogene 1, receptor tyrosine kinase | Plasma Membrane | kinase |
| CDDP 11 | SGK1 | serum/glucocorticoid regulated kinase 1 | Cytoplasm | kinase |
| CDDP 11 | SIK2 | salt inducible kinase 2 | Cytoplasm | kinase |
| CDDP 11 | STK17A | serine/threonine kinase 17a | Nucleus | kinase |
| CDDP 11 | STK17B | serine/threonine kinase 17b | Nucleus | kinase |
| CDDP 11 | STK38L | serine/threonine kinase 38 like | Cytoplasm | kinase |
| CDDP 11 | STK39 | serine/threonine kinase 39 | Nucleus | kinase |
| CDDP 11 | SYK | spleen associated tyrosine kinase | Cytoplasm | kinase |
| CDDP 11 | TBK1 | TANK binding kinase 1 | Cytoplasm | kinase |
| CDDP 11 | TXK | TXK tyrosine kinase | Cytoplasm | kinase |
| CDDP 11 | TYK2 | tyrosine kinase 2 | Plasma Membrane | kinase |
| CDDP 11 | TYRO3 | TYRO3 protein tyrosine kinase | Plasma Membrane | kinase |
| CDDP 11 | ULK1 | unc-51 like autophagy activating kinase 1 | Cytoplasm | kinase |
| CDDP 11 | ULK2 | unc-51 like autophagy activating kinase 2 | Cytoplasm | kinase |
| CDDP 11 | ULK3 | unc-51 like kinase 3 | Cytoplasm | kinase |
| CDDP 11 | YES1 | YES proto-oncogene 1, Src family tyrosine kinase | Cytoplasm | kinase |
| CDDP 12 | AAK1 | AP2 associated kinase 1 | Cytoplasm | kinase |
| CDDP 12 | ACVR1 | activin A receptor type 1 | Plasma Membrane | kinase |
| CDDP 12 | ACVR1B | activin A receptor type 1B | Plasma Membrane | kinase |
| CDDP 12 | ANKK1 | ankyrin repeat and kinase domain containing 1 | Cytoplasm | kinase |
| CDDP 12 | AURKA | aurora kinase A | Nucleus | kinase |
| CDDP 12 | AURKB | aurora kinase B | Nucleus | kinase |
| CDDP 12 | AURKC | aurora kinase C | Nucleus | kinase |
| CDDP 12 | AXL | AXL receptor tyrosine kinase | Plasma Membrane | kinase |
| CDDP 12 | BLK | BLK proto-oncogene, Src family tyrosine kinase | Cytoplasm | kinase |
| CDDP 12 | BMP2K | BMP2 inducible kinase | Nucleus | kinase |
| CDDP 12 | BMPR1B | bone morphogenetic protein receptor type 1B | Plasma Membrane | kinase |
| CDDP 12 | BMX | BMX non-receptor tyrosine kinase | Cytoplasm | kinase |
| CDDP 12 | BRSK1 | BR serine/threonine kinase 1 | Cytoplasm | kinase |
| CDDP 12 | BTK | Bruton tyrosine kinase | Cytoplasm | kinase |
| CDDP 12 | CAMK1D | calcium/calmodulin dependent protein kinase ID | Cytoplasm | kinase |
| CDDP 12 | CAMK1G | calcium/calmodulin dependent protein kinase IG | Cytoplasm | kinase |
| CDDP 12 | CAMK2A | calcium/calmodulin dependent protein kinase II alpha | Cytoplasm | kinase |
| CDDP 12 | CAMK2D | calcium/calmodulin dependent protein kinase II delta | Cytoplasm | kinase |
| CDDP 12 | CAMKK1 | calcium/calmodulin dependent protein kinase kinase 1 | Cytoplasm | kinase |
| CDDP 12 | CAMKK2 | calcium/calmodulin dependent protein kinase kinase 2 | Cytoplasm | kinase |
| CDDP 12 | CDC42BPB | CDC42 binding protein kinase beta | Cytoplasm | kinase |
| CDDP 12 | CDC7 | cell division cycle 7 | Nucleus | kinase |
| CDDP 12 | CDK3 | cyclin dependent kinase 3 | Other | kinase |
| CDDP 12 | CDK5 | cyclin dependent kinase 5 | Nucleus | kinase |
| CDDP 12 | CDK8 | cyclin dependent kinase 8 | Nucleus | kinase |
| CDDP 12 | CDK9 | cyclin dependent kinase 9 | Nucleus | kinase |
| CDDP 12 | CDKL2 | cyclin dependent kinase like 2 | Nucleus | kinase |
| CDDP 12 | CDKL3 | cyclin dependent kinase like 3 | Cytoplasm | kinase |
| CDDP 12 | CDKL5 | cyclin dependent kinase like 5 | Nucleus | kinase |
| CDDP 12 | CHUK | component of inhibitor of nuclear factor kappa B kinase complex | Cytoplasm | kinase |
| CDDP 12 | CLK4 | CDC like kinase 4 | Nucleus | kinase |
| CDDP 12 | CSNK1A1 | casein kinase 1 alpha 1 | Cytoplasm | kinase |
| CDDP 12 | CSNK1A1L | casein kinase 1 alpha 1 like | Cytoplasm | kinase |
| CDDP 12 | CSNK1E | casein kinase 1 epsilon | Cytoplasm | kinase |
| CDDP 12 | CSNK1G2 | casein kinase 1 gamma 2 | Cytoplasm | kinase |
| CDDP 12 | CSNK2A1 | casein kinase 2 alpha 1 | Nucleus | kinase |
| CDDP 12 | CSNK2A2 | casein kinase 2 alpha 2 | Cytoplasm | kinase |
| CDDP 12 | CHEK1 | checkpoint kinase 1 | Nucleus | kinase |
| CDDP 12 | CHEK2 | checkpoint kinase 2 | Nucleus | kinase |
| CDDP 12 | DAPK3 | death associated protein kinase 3 | Cytoplasm | kinase |
| CDDP 12 | DDR1 | discoidin domain receptor tyrosine kinase 1 | Plasma Membrane | kinase |
| CDDP 12 | DYRK1A | dual specificity tyrosine phosphorylation regulated kinase 1A | Nucleus | kinase |
| CDDP 12 | DYRK1B | dual specificity tyrosine phosphorylation regulated kinase 1B | Nucleus | kinase |
| CDDP 12 | DYRK3 | dual specificity tyrosine phosphorylation regulated kinase 3 | Nucleus | kinase |
| CDDP 12 | DYRK4 | dual specificity tyrosine phosphorylation regulated kinase 4 | Nucleus | kinase |
| CDDP 12 | EEF2K | eukaryotic elongation factor 2 kinase | Cytoplasm | kinase |
| CDDP 12 | EIF2AK2 | eukaryotic translation initiation factor 2 alpha kinase 2 | Cytoplasm | kinase |
| CDDP 12 | EPHA1 | EPH receptor A1 | Plasma Membrane | kinase |
| CDDP 12 | EPHA3 | EPH receptor A3 | Plasma Membrane | kinase |
| CDDP 12 | EPHA4 | EPH receptor A4 | Plasma Membrane | kinase |
| CDDP 12 | EPHA7 | EPH receptor A7 | Plasma Membrane | kinase |
| CDDP 12 | EPHB6 | EPH receptor B6 | Plasma Membrane | kinase |
| CDDP 12 | MAPK1 | mitogen-activated protein kinase 1 | Cytoplasm | kinase |
| CDDP 12 | MAPK7 | mitogen-activated protein kinase 7 | Cytoplasm | kinase |
| CDDP 12 | FER | FER tyrosine kinase | Cytoplasm | kinase |
| CDDP 12 | FES | FES proto-oncogene, tyrosine kinase | Cytoplasm | kinase |
| CDDP 12 | FGFR1 | fibroblast growth factor receptor 1 | Plasma Membrane | kinase |
| CDDP 12 | FGFR2 | fibroblast growth factor receptor 2 | Plasma Membrane | kinase |
| CDDP 12 | FGFR3 | fibroblast growth factor receptor 3 | Plasma Membrane | kinase |
| CDDP 12 | FGR | FGR proto-oncogene, Src family tyrosine kinase | Nucleus | kinase |
| CDDP 12 | GAK | cyclin G associated kinase | Nucleus | kinase |
| CDDP 12 | GRK1 | G protein-coupled receptor kinase 1 | Plasma Membrane | kinase |
| CDDP 12 | GRK4 | G protein-coupled receptor kinase 4 | Plasma Membrane | kinase |
| CDDP 12 | GRK5 | G protein-coupled receptor kinase 5 | Plasma Membrane | kinase |
| CDDP 12 | GRK7 | G protein-coupled receptor kinase 7 | Cytoplasm | kinase |
| CDDP 12 | GSK3B | glycogen synthase kinase 3 beta | Nucleus | kinase |
| CDDP 12 | HCK | HCK proto-oncogene, Src family tyrosine kinase | Cytoplasm | kinase |
| CDDP 12 | HIPK1 | homeodomain interacting protein kinase 1 | Nucleus | kinase |
| CDDP 12 | HIPK3 | homeodomain interacting protein kinase 3 | Nucleus | kinase |
| CDDP 12 | EIF2AK1 | eukaryotic translation initiation factor 2 alpha kinase 1 | Cytoplasm | kinase |
| CDDP 12 | CILK1 | ciliogenesis Associated Kinase 1 | Nucleus | kinase |
| CDDP 12 | IGF1R | insulin like growth factor 1 receptor | Plasma Membrane | transmembrane receptor |
| CDDP 12 | IKBKE | inhibitor of nuclear factor kappa B kinase subunit epsilon | Cytoplasm | kinase |
| CDDP 12 | INSR | insulin receptor | Plasma Membrane | kinase |
| CDDP 12 | IRAK1 | interleukin 1 receptor associated kinase 1 | Plasma Membrane | kinase |
| CDDP 12 | JAK2 | Janus kinase 2 | Cytoplasm | kinase |
| CDDP 12 | JAK3 | Janus kinase 3 | Cytoplasm | kinase |
| CDDP 12 | MAPK8 | mitogen-activated protein kinase 8 | Cytoplasm | kinase |
| CDDP 12 | MAPK9 | mitogen-activated protein kinase 9 | Cytoplasm | kinase |
| CDDP 12 | MAPK10 | mitogen-activated protein kinase 10 | Cytoplasm | kinase |
| CDDP 12 | LATS1 | large tumor suppressor kinase 1 | Nucleus | kinase |
| CDDP 12 | LIMK1 | LIM domain kinase 1 | Cytoplasm | kinase |
| CDDP 12 | LRRK2 | leucine rich repeat kinase 2 | Cytoplasm | kinase |
| CDDP 12 | LTK | leukocyte receptor tyrosine kinase | Plasma Membrane | kinase |
| CDDP 12 | MAP3K10 | mitogen-activated protein kinase kinase kinase 10 | Cytoplasm | kinase |
| CDDP 12 | MAP3K11 | mitogen-activated protein kinase kinase kinase 11 | Cytoplasm | kinase |
| CDDP 12 | MAP3K13 | mitogen-activated protein kinase kinase kinase 13 | Cytoplasm | kinase |
| CDDP 12 | MAP3K2 | mitogen-activated protein kinase kinase kinase 2 | Cytoplasm | kinase |
| CDDP 12 | MAP3K3 | mitogen-activated protein kinase kinase kinase 3 | Cytoplasm | kinase |
| CDDP 12 | MAP3K6 | mitogen-activated protein kinase kinase kinase 6 | Other | kinase |
| CDDP 12 | MAP3K9 | mitogen-activated protein kinase kinase kinase 9 | Cytoplasm | kinase |
| CDDP 12 | MAP4K1 | mitogen-activated protein kinase kinase kinase kinase 1 | Cytoplasm | kinase |
| CDDP 12 | MAP4K2 | mitogen-activated protein kinase kinase kinase kinase 2 | Cytoplasm | kinase |
| CDDP 12 | MAP4K3 | mitogen-activated protein kinase kinase kinase kinase 3 | Other | kinase |
| CDDP 12 | MAP4K4 | mitogen-activated protein kinase kinase kinase kinase 4 | Cytoplasm | kinase |
| CDDP 12 | MAP4K5 | mitogen-activated protein kinase kinase kinase kinase 5 | Cytoplasm | kinase |
| CDDP 12 | MAPKAPK3 | MAPK activated protein kinase 3 | Nucleus | kinase |
| CDDP 12 | MAPKAPK5 | MAPK activated protein kinase 5 | Cytoplasm | kinase |
| CDDP 12 | MARK2 | microtubule affinity regulating kinase 2 | Cytoplasm | kinase |
| CDDP 12 | MARK3 | microtubule affinity regulating kinase 3 | Cytoplasm | kinase |
| CDDP 12 | MARK4 | microtubule affinity regulating kinase 4 | Cytoplasm | kinase |
| CDDP 12 | MAST1 | microtubule associated serine/threonine kinase 1 | Cytoplasm | kinase |
| CDDP 12 | MATK | megakaryocyte-associated tyrosine kinase | Cytoplasm | kinase |
| CDDP 12 | MAP2K1 | mitogen-activated protein kinase kinase 1 | Cytoplasm | kinase |
| CDDP 12 | MAP2K2 | mitogen-activated protein kinase kinase 2 | Cytoplasm | kinase |
| CDDP 12 | MELK | maternal embryonic leucine zipper kinase | Cytoplasm | kinase |
| CDDP 12 | MET | MET proto-oncogene, receptor tyrosine kinase | Plasma Membrane | kinase |
| CDDP 12 | MINK1 | misshapen like kinase 1 | Cytoplasm | kinase |
| CDDP 12 | MAP2K3 | mitogen-activated protein kinase kinase 3 | Cytoplasm | kinase |
| CDDP 12 | MAP2K4 | mitogen-activated protein kinase kinase 4 | Cytoplasm | kinase |
| CDDP 12 | MKNK2 | MAPK interacting serine/threonine kinase 2 | Cytoplasm | kinase |
| CDDP 12 | RPS6KA4 | ribosomal protein S6 kinase A4 | Cytoplasm | kinase |
| CDDP 12 | SRPK3 | SRSF protein kinase 3 | Cytoplasm | kinase |
| CDDP 12 | MST1R | macrophage stimulating 1 receptor | Plasma Membrane | kinase |
| CDDP 12 | MYLK2 | myosin light chain kinase 2 | Cytoplasm | kinase |
| CDDP 12 | MYO3A | myosin IIIA | Cytoplasm | kinase |
| CDDP 12 | MYO3B | myosin IIIB | Plasma Membrane | kinase |
| CDDP 12 | NEK2 | NIMA related kinase 2 | Cytoplasm | kinase |
| CDDP 12 | NEK5 | NIMA related kinase 5 | Other | kinase |
| CDDP 12 | NEK6 | NIMA related kinase 6 | Nucleus | kinase |
| CDDP 12 | NEK7 | NIMA related kinase 7 | Nucleus | kinase |
| CDDP 12 | NLK | nemo like kinase | Nucleus | kinase |
| CDDP 12 | NTRK1 | neurotrophic receptor tyrosine kinase 1 | Plasma Membrane | kinase |
| CDDP 12 | NUAK2 | NUAK family kinase 2 | Other | kinase |
| CDDP 12 | OXSR1 | oxidative stress responsive kinase 1 | Nucleus | kinase |
| CDDP 12 | PAK2 | p21 (RAC1) activated kinase 2 | Cytoplasm | kinase |
| CDDP 12 | PAK3 | p21 (RAC1) activated kinase 3 | Cytoplasm | kinase |
| CDDP 12 | PAK6 | p21 (RAC1) activated kinase 6 | Cytoplasm | kinase |
| CDDP 12 | PAK5 | p21 (RAC1) activated kinase 5 | Nucleus | kinase |
| CDDP 12 | PASK | PAS domain containing serine/threonine kinase | Cytoplasm | kinase |
| CDDP 12 | PBK | PDZ binding kinase | Cytoplasm | kinase |
| CDDP 12 | CDK17 | cyclin dependent kinase 17 | Cytoplasm | kinase |
| CDDP 12 | PDPK1 | 3-phosphoinositide dependent protein kinase 1 | Cytoplasm | kinase |
| CDDP 12 | EIF2AK3 | eukaryotic translation initiation factor 2 alpha kinase 3 | Cytoplasm | kinase |
| CDDP 12 | PHKG1 | phosphorylase kinase catalytic subunit gamma 1 | Cytoplasm | kinase |
| CDDP 12 | PHKG2 | phosphorylase kinase catalytic subunit gamma 2 | Cytoplasm | kinase |
| CDDP 12 | PIM1 | Pim-1 proto-oncogene, serine/threonine kinase | Cytoplasm | kinase |
| CDDP 12 | PIM2 | Pim-2 proto-oncogene, serine/threonine kinase | Nucleus | kinase |
| CDDP 12 | PIM3 | Pim-3 proto-oncogene, serine/threonine kinase | Cytoplasm | kinase |
| CDDP 12 | PRKCB | protein kinase C beta | Cytoplasm | kinase |
| CDDP 12 | PKD2 | polycystin 2, transient receptor potential cation channel | Plasma Membrane | kinase |
| CDDP 12 | PKD3 | polycystic Kidney Disease 3 (Autosomal Dominant) | Plasma Membrane | kinase |
| CDDP 12 | PKN2 | protein kinase N2 | Cytoplasm | kinase |
| CDDP 12 | PLK1 | polo like kinase 1 | Nucleus | kinase |
| CDDP 12 | PLK2 | polo like kinase 2 | Nucleus | kinase |
| CDDP 12 | PLK3 | polo like kinase 3 | Nucleus | kinase |
| CDDP 12 | PLK4 | polo like kinase 4 | Cytoplasm | kinase |
| CDDP 12 | PRKAA1 | protein kinase AMP-activated catalytic subunit alpha 1 | Cytoplasm | kinase |
| CDDP 12 | PRKAA2 | protein kinase AMP-activated catalytic subunit alpha 2 | Cytoplasm | kinase |
| CDDP 12 | PRKG1 | protein kinase cGMP-dependent 1 | Cytoplasm | kinase |
| CDDP 12 | PRKX | protein kinase X-linked | Cytoplasm | kinase |
| CDDP 12 | SIK3 | SIK family kinase 3 | Cytoplasm | kinase |
| CDDP 12 | RIPK1 | receptor interacting serine/threonine kinase 1 | Plasma Membrane | kinase |
| CDDP 12 | RIPK2 | receptor interacting serine/threonine kinase 2 | Plasma Membrane | kinase |
| CDDP 12 | ROS1 | ROS proto-oncogene 1, receptor tyrosine kinase | Plasma Membrane | kinase |
| CDDP 12 | SGK1 | serum/glucocorticoid regulated kinase 1 | Cytoplasm | kinase |
| CDDP 12 | SIK2 | salt inducible kinase 2 | Cytoplasm | kinase |
| CDDP 12 | SRPK1 | SRSF protein kinase 1 | Nucleus | kinase |
| CDDP 12 | STK11 | serine/threonine kinase 11 | Cytoplasm | kinase |
| CDDP 12 | STK16 | serine/threonine kinase 16 | Cytoplasm | kinase |
| CDDP 12 | STK17A | serine/threonine kinase 17a | Nucleus | kinase |
| CDDP 12 | STK17B | serine/threonine kinase 17b | Nucleus | kinase |
| CDDP 12 | STK24 | serine/threonine kinase 24 | Cytoplasm | kinase |
| CDDP 12 | STK25 | serine/threonine kinase 25 | Cytoplasm | kinase |
| CDDP 12 | STK3 | serine/threonine kinase 3 | Cytoplasm | kinase |
| CDDP 12 | STK33 | serine/threonine kinase 33 | Cytoplasm | kinase |
| CDDP 12 | STK35 | serine/threonine kinase 35 | Cytoplasm | kinase |
| CDDP 12 | STK38L | serine/threonine kinase 38 like | Cytoplasm | kinase |
| CDDP 12 | STK39 | serine/threonine kinase 39 | Nucleus | kinase |
| CDDP 12 | STK4 | serine/threonine kinase 4 | Cytoplasm | kinase |
| CDDP 12 | SYK | spleen associated tyrosine kinase | Cytoplasm | kinase |
| CDDP 12 | MYLK4 | myosin light chain kinase family member 4 | Cytoplasm | kinase |
| CDDP 12 | SBK3 | SH3 domain binding kinase family member 3 | Other | other |
| CDDP 12 | TAOK1 | TAO kinase 1 | Cytoplasm | kinase |
| CDDP 12 | TAOK3 | TAO kinase 3 | Cytoplasm | kinase |
| CDDP 12 | TBK1 | TANK binding kinase 1 | Cytoplasm | kinase |
| CDDP 12 | TESK1 | testis associated actin remodelling kinase 1 | Nucleus | kinase |
| CDDP 12 | TLK1 | tousled like kinase 1 | Nucleus | kinase |
| CDDP 12 | TLK2 | tousled like kinase 2 | Cytoplasm | kinase |
| CDDP 12 | TNIK | TRAF2 and NCK interacting kinase | Plasma Membrane | kinase |
| CDDP 12 | TNK1 | tyrosine kinase non receptor 1 | Cytoplasm | kinase |
| CDDP 12 | TNK2 | tyrosine kinase non receptor 2 | Cytoplasm | kinase |
| CDDP 12 | TNNI3K | TNNI3 interacting kinase | Cytoplasm | kinase |
| CDDP 12 | TSSK1B | testis specific serine kinase 1B | Cytoplasm | kinase |
| CDDP 12 | TSSK2 | testis specific serine kinase 2 | Cytoplasm | kinase |
| CDDP 12 | TXK | TXK tyrosine kinase | Cytoplasm | kinase |
| CDDP 12 | TYK2 | tyrosine kinase 2 | Plasma Membrane | kinase |
| CDDP 12 | TYRO3 | TYRO3 protein tyrosine kinase | Plasma Membrane | kinase |
| CDDP 12 | ULK1 | unc-51 like autophagy activating kinase 1 | Cytoplasm | kinase |
| CDDP 12 | ULK2 | unc-51 like autophagy activating kinase 2 | Cytoplasm | kinase |
| CDDP 12 | ULK3 | unc-51 like kinase 3 | Cytoplasm | kinase |
| CDDP 12 | FLT4 | fms related tyrosine kinase 4 | Plasma Membrane | transmembrane receptor |
| CDDP 12 | YES1 | YES proto-oncogene 1, Src family tyrosine kinase | Cytoplasm | kinase |
| CDDP 12 | MAP3K20 | mitogen-activated protein kinase kinase kinase 20 | Cytoplasm | kinase |
| CDDP 12 | ZAP70 | zeta chain of T cell receptor associated protein kinase 70 | Plasma Membrane | kinase |
| CDDP 13 | AKT2 | AKT serine/threonine kinase 2 | Cytoplasm | kinase |
| CDDP 13 | ACVR1B | activin A receptor type 1B | Plasma Membrane | kinase |
| CDDP 13 | AURKA | aurora kinase A | Nucleus | kinase |
| CDDP 13 | AURKB | aurora kinase B | Nucleus | kinase |
| CDDP 13 | AURKC | aurora kinase C | Nucleus | kinase |
| CDDP 13 | AXL | AXL receptor tyrosine kinase | Plasma Membrane | kinase |
| CDDP 13 | BLK | BLK proto-oncogene, Src family tyrosine kinase | Cytoplasm | kinase |
| CDDP 13 | BMX | BMX non-receptor tyrosine kinase | Cytoplasm | kinase |
| CDDP 13 | BRSK1 | BR serine/threonine kinase 1 | Cytoplasm | kinase |
| CDDP 13 | BTK | Bruton tyrosine kinase | Cytoplasm | kinase |
| CDDP 13 | CAMK1D | calcium/calmodulin dependent protein kinase ID | Cytoplasm | kinase |
| CDDP 13 | CAMK2D | calcium/calmodulin dependent protein kinase II delta | Cytoplasm | kinase |
| CDDP 13 | CAMKK1 | calcium/calmodulin dependent protein kinase kinase 1 | Cytoplasm | kinase |
| CDDP 13 | CAMKK2 | calcium/calmodulin dependent protein kinase kinase 2 | Cytoplasm | kinase |
| CDDP 13 | CDK3 | cyclin dependent kinase 3 | Other | kinase |
| CDDP 13 | CDK9 | cyclin dependent kinase 9 | Nucleus | kinase |
| CDDP 13 | CDKL2 | cyclin dependent kinase like 2 | Nucleus | kinase |
| CDDP 13 | CDKL3 | cyclin dependent kinase like 3 | Cytoplasm | kinase |
| CDDP 13 | CSF1R | colony stimulating factor 1 receptor | Plasma Membrane | kinase |
| CDDP 13 | CHEK1 | checkpoint kinase 1 | Nucleus | kinase |
| CDDP 13 | DDR1 | discoidin domain receptor tyrosine kinase 1 | Plasma Membrane | kinase |
| CDDP 13 | DYRK1B | dual specificity tyrosine phosphorylation regulated kinase 1B | Nucleus | kinase |
| CDDP 13 | EIF2AK2 | eukaryotic translation initiation factor 2 alpha kinase 2 | Cytoplasm | kinase |
| CDDP 13 | EPHA3 | EPH receptor A3 | Plasma Membrane | kinase |
| CDDP 13 | EPHA5 | EPH receptor A5 | Plasma Membrane | kinase |
| CDDP 13 | EPHA7 | EPH receptor A7 | Plasma Membrane | kinase |
| CDDP 13 | EPHB6 | EPH receptor B6 | Plasma Membrane | kinase |
| CDDP 13 | MAPK7 | mitogen-activated protein kinase 7 | Cytoplasm | kinase |
| CDDP 13 | PTK2B | protein tyrosine kinase 2 beta | Cytoplasm | kinase |
| CDDP 13 | FER | FER tyrosine kinase | Cytoplasm | kinase |
| CDDP 13 | FES | FES proto-oncogene, tyrosine kinase | Cytoplasm | kinase |
| CDDP 13 | FGFR1 | fibroblast growth factor receptor 1 | Plasma Membrane | kinase |
| CDDP 13 | FGFR3 | fibroblast growth factor receptor 3 | Plasma Membrane | kinase |
| CDDP 13 | FLT3 | fms related tyrosine kinase 3 | Plasma Membrane | kinase |
| CDDP 13 | GAK | cyclin G associated kinase | Nucleus | kinase |
| CDDP 13 | GRK1 | G protein-coupled receptor kinase 1 | Plasma Membrane | kinase |
| CDDP 13 | GRK4 | G protein-coupled receptor kinase 4 | Plasma Membrane | kinase |
| CDDP 13 | GRK5 | G protein-coupled receptor kinase 5 | Plasma Membrane | kinase |
| CDDP 13 | GRK7 | G protein-coupled receptor kinase 7 | Cytoplasm | kinase |
| CDDP 13 | HIPK3 | homeodomain interacting protein kinase 3 | Nucleus | kinase |
| CDDP 13 | EIF2AK1 | eukaryotic translation initiation factor 2 alpha kinase 1 | Cytoplasm | kinase |
| CDDP 13 | CILK1 | ciliogenesis Associated Kinase 1 | Nucleus | kinase |
| CDDP 13 | INSR | insulin receptor | Plasma Membrane | kinase |
| CDDP 13 | KIT | KIT proto-oncogene, receptor tyrosine kinase | Plasma Membrane | transmembrane receptor |
| CDDP 13 | LIMK1 | LIM domain kinase 1 | Cytoplasm | kinase |
| CDDP 13 | LRRK2 | leucine rich repeat kinase 2 | Cytoplasm | kinase |
| CDDP 13 | LTK | leukocyte receptor tyrosine kinase | Plasma Membrane | kinase |
| CDDP 13 | LYN | LYN proto-oncogene, Src family tyrosine kinase | Cytoplasm | kinase |
| CDDP 13 | MAP3K11 | mitogen-activated protein kinase kinase kinase 11 | Cytoplasm | kinase |
| CDDP 13 | MAP3K13 | mitogen-activated protein kinase kinase kinase 13 | Cytoplasm | kinase |
| CDDP 13 | MAP3K15 | mitogen-activated protein kinase kinase kinase 15 | Other | other |
| CDDP 13 | MAP3K2 | mitogen-activated protein kinase kinase kinase 2 | Cytoplasm | kinase |
| CDDP 13 | MAP3K3 | mitogen-activated protein kinase kinase kinase 3 | Cytoplasm | kinase |
| CDDP 13 | MAP3K9 | mitogen-activated protein kinase kinase kinase 9 | Cytoplasm | kinase |
| CDDP 13 | MAP4K1 | mitogen-activated protein kinase kinase kinase kinase 1 | Cytoplasm | kinase |
| CDDP 13 | MAP4K3 | mitogen-activated protein kinase kinase kinase kinase 3 | Other | kinase |
| CDDP 13 | MAP4K4 | mitogen-activated protein kinase kinase kinase kinase 4 | Cytoplasm | kinase |
| CDDP 13 | MAP4K5 | mitogen-activated protein kinase kinase kinase kinase 5 | Cytoplasm | kinase |
| CDDP 13 | MAPKAPK5 | MAPK activated protein kinase 5 | Cytoplasm | kinase |
| CDDP 13 | MARK2 | microtubule affinity regulating kinase 2 | Cytoplasm | kinase |
| CDDP 13 | MAST1 | microtubule associated serine/threonine kinase 1 | Cytoplasm | kinase |
| CDDP 13 | MATK | megakaryocyte-associated tyrosine kinase | Cytoplasm | kinase |
| CDDP 13 | MAP2K1 | mitogen-activated protein kinase kinase 1 | Cytoplasm | kinase |
| CDDP 13 | MAP2K2 | mitogen-activated protein kinase kinase 2 | Cytoplasm | kinase |
| CDDP 13 | MELK | maternal embryonic leucine zipper kinase | Cytoplasm | kinase |
| CDDP 13 | MET | MET proto-oncogene, receptor tyrosine kinase | Plasma Membrane | kinase |
| CDDP 13 | RPS6KA4 | ribosomal protein S6 kinase A4 | Cytoplasm | kinase |
| CDDP 13 | MST1R | macrophage stimulating 1 receptor | Plasma Membrane | kinase |
| CDDP 13 | MYLK2 | myosin light chain kinase 2 | Cytoplasm | kinase |
| CDDP 13 | NEK2 | NIMA related kinase 2 | Cytoplasm | kinase |
| CDDP 13 | NLK | nemo like kinase | Nucleus | kinase |
| CDDP 13 | NTRK1 | neurotrophic receptor tyrosine kinase 1 | Plasma Membrane | kinase |
| CDDP 13 | OXSR1 | oxidative stress responsive kinase 1 | Nucleus | kinase |
| CDDP 13 | CDK17 | cyclin dependent kinase 17 | Cytoplasm | kinase |
| CDDP 13 | PDPK1 | 3-phosphoinositide dependent protein kinase 1 | Cytoplasm | kinase |
| CDDP 13 | PKD2 | polycystin 2, transient receptor potential cation channel | Plasma Membrane | kinase |
| CDDP 13 | PLK1 | polo like kinase 1 | Nucleus | kinase |
| CDDP 13 | PLK3 | polo like kinase 3 | Nucleus | kinase |
| CDDP 13 | PLK4 | polo like kinase 4 | Cytoplasm | kinase |
| CDDP 13 | PRKAA1 | protein kinase AMP-activated catalytic subunit alpha 1 | Cytoplasm | kinase |
| CDDP 13 | RAF1 | Raf-1 proto-oncogene, serine/threonine kinase | Cytoplasm | kinase |
| CDDP 13 | RIPK1 | receptor interacting serine/threonine kinase 1 | Plasma Membrane | kinase |
| CDDP 13 | RIPK2 | receptor interacting serine/threonine kinase 2 | Plasma Membrane | kinase |
| CDDP 13 | ROS1 | ROS proto-oncogene 1, receptor tyrosine kinase | Plasma Membrane | kinase |
| CDDP 13 | SLK | STE20 like kinase | Nucleus | kinase |
| CDDP 13 | SIK2 | salt inducible kinase 2 | Cytoplasm | kinase |
| CDDP 13 | SRPK1 | SRSF protein kinase 1 | Nucleus | kinase |
| CDDP 13 | STK17B | serine/threonine kinase 17b | Nucleus | kinase |
| CDDP 13 | STK3 | serine/threonine kinase 3 | Cytoplasm | kinase |
| CDDP 13 | STK38L | serine/threonine kinase 38 like | Cytoplasm | kinase |
| CDDP 13 | STK39 | serine/threonine kinase 39 | Nucleus | kinase |
| CDDP 13 | SYK | spleen associated tyrosine kinase | Cytoplasm | kinase |
| CDDP 13 | TAOK1 | TAO kinase 1 | Cytoplasm | kinase |
| CDDP 13 | TAOK3 | TAO kinase 3 | Cytoplasm | kinase |
| CDDP 13 | TESK1 | testis associated actin remodelling kinase 1 | Nucleus | kinase |
| CDDP 13 | TLK1 | tousled like kinase 1 | Nucleus | kinase |
| CDDP 13 | TNK2 | tyrosine kinase non receptor 2 | Cytoplasm | kinase |
| CDDP 13 | TSSK1B | testis specific serine kinase 1B | Cytoplasm | kinase |
| CDDP 13 | TSSK2 | testis specific serine kinase 2 | Cytoplasm | kinase |
| CDDP 13 | TXK | TXK tyrosine kinase | Cytoplasm | kinase |
| CDDP 13 | TYRO3 | TYRO3 protein tyrosine kinase | Plasma Membrane | kinase |
| CDDP 13 | ULK1 | unc-51 like autophagy activating kinase 1 | Cytoplasm | kinase |
| CDDP 13 | ULK2 | unc-51 like autophagy activating kinase 2 | Cytoplasm | kinase |
| CDDP 13 | ULK3 | unc-51 like kinase 3 | Cytoplasm | kinase |
| CDDP 13 | FLT4 | fms related tyrosine kinase 4 | Plasma Membrane | transmembrane receptor |
| CDDP 13 | YES1 | YES proto-oncogene 1, Src family tyrosine kinase | Cytoplasm | kinase |
| CDDP 13 | ZAP70 | zeta chain of T cell receptor associated protein kinase 70 | Plasma Membrane | kinase |
| CDDP 14 | ALK | ALK receptor tyrosine kinase | Plasma Membrane | kinase |
| CDDP 14 | CSF1R | colony stimulating factor 1 receptor | Plasma Membrane | kinase |
| CDDP 14 | CSNK1E | casein kinase 1 epsilon | Cytoplasm | kinase |
| CDDP 14 | CHEK1 | checkpoint kinase 1 | Nucleus | kinase |
| CDDP 14 | GAK | cyclin G associated kinase | Nucleus | kinase |
| CDDP 14 | GRK1 | G protein-coupled receptor kinase 1 | Plasma Membrane | kinase |
| CDDP 14 | EIF2AK1 | eukaryotic translation initiation factor 2 alpha kinase 1 | Cytoplasm | kinase |
| CDDP 14 | IGF1R | insulin like growth factor 1 receptor | Plasma Membrane | transmembrane receptor |
| CDDP 14 | INSR | insulin receptor | Plasma Membrane | kinase |
| CDDP 14 | MAPK9 | mitogen-activated protein kinase 9 | Cytoplasm | kinase |
| CDDP 14 | MAPK10 | mitogen-activated protein kinase 10 | Cytoplasm | kinase |
| CDDP 14 | LIMK1 | LIM domain kinase 1 | Cytoplasm | kinase |
| CDDP 14 | LTK | leukocyte receptor tyrosine kinase | Plasma Membrane | kinase |
| CDDP 14 | MAP3K15 | mitogen-activated protein kinase kinase kinase 15 | Other | other |
| CDDP 14 | MELK | maternal embryonic leucine zipper kinase | Cytoplasm | kinase |
| CDDP 14 | RIPK1 | receptor interacting serine/threonine kinase 1 | Plasma Membrane | kinase |
| CDDP 14 | ROS1 | ROS proto-oncogene 1, receptor tyrosine kinase | Plasma Membrane | kinase |
| CDDP 14 | STK36 | serine/threonine kinase 36 | Cytoplasm | kinase |
| CDDP 14 | TNK2 | tyrosine kinase non receptor 2 | Cytoplasm | kinase |
| CDDP 15 | GRK1 | G protein-coupled receptor kinase 1 | Plasma Membrane | kinase |
| CDDP 15 | EIF2AK1 | eukaryotic translation initiation factor 2 alpha kinase 1 | Cytoplasm | kinase |
| CDDP 15 | MAPK14 | mitogen-activated protein kinase 14 | Cytoplasm | kinase |
| CDDP 16 | CDK15 | cyclin dependent kinase 15 | Plasma Membrane | kinase |
| CDDP 16 | BLK | BLK proto-oncogene, Src family tyrosine kinase | Cytoplasm | kinase |
| CDDP 16 | BTK | Bruton tyrosine kinase | Cytoplasm | kinase |
| CDDP 16 | CDKL2 | cyclin dependent kinase like 2 | Nucleus | kinase |
| CDDP 16 | CDKL3 | cyclin dependent kinase like 3 | Cytoplasm | kinase |
| CDDP 16 | CSF1R | colony stimulating factor 1 receptor | Plasma Membrane | kinase |
| CDDP 16 | CHEK1 | checkpoint kinase 1 | Nucleus | kinase |
| CDDP 16 | EPHA4 | EPH receptor A4 | Plasma Membrane | kinase |
| CDDP 16 | EPHB6 | EPH receptor B6 | Plasma Membrane | kinase |
| CDDP 16 | FGFR1 | fibroblast growth factor receptor 1 | Plasma Membrane | kinase |
| CDDP 16 | FGFR3 | fibroblast growth factor receptor 3 | Plasma Membrane | kinase |
| CDDP 16 | GAK | cyclin G associated kinase | Nucleus | kinase |
| CDDP 16 | GRK1 | G protein-coupled receptor kinase 1 | Plasma Membrane | kinase |
| CDDP 16 | HCK | HCK proto-oncogene, Src family tyrosine kinase | Cytoplasm | kinase |
| CDDP 16 | HIPK3 | homeodomain interacting protein kinase 3 | Nucleus | kinase |
| CDDP 16 | EIF2AK1 | eukaryotic translation initiation factor 2 alpha kinase 1 | Cytoplasm | kinase |
| CDDP 16 | IGF1R | insulin like growth factor 1 receptor | Plasma Membrane | transmembrane receptor |
| CDDP 16 | INSR | insulin receptor | Plasma Membrane | kinase |
| CDDP 16 | MAPK10 | mitogen-activated protein kinase 10 | Cytoplasm | kinase |
| CDDP 16 | LCK | LCK proto-oncogene, Src family tyrosine kinase | Cytoplasm | kinase |
| CDDP 16 | LIMK1 | LIM domain kinase 1 | Cytoplasm | kinase |
| CDDP 16 | LTK | leukocyte receptor tyrosine kinase | Plasma Membrane | kinase |
| CDDP 16 | LYN | LYN proto-oncogene, Src family tyrosine kinase | Cytoplasm | kinase |
| CDDP 16 | MAP3K15 | mitogen-activated protein kinase kinase kinase 15 | Other | other |
| CDDP 16 | MAP2K1 | mitogen-activated protein kinase kinase 1 | Cytoplasm | kinase |
| CDDP 16 | MELK | maternal embryonic leucine zipper kinase | Cytoplasm | kinase |
| CDDP 16 | MKNK1 | MAPK interacting serine/threonine kinase 1 | Cytoplasm | kinase |
| CDDP 16 | NLK | nemo like kinase | Nucleus | kinase |
| CDDP 16 | RIPK1 | receptor interacting serine/threonine kinase 1 | Plasma Membrane | kinase |
| CDDP 16 | ROS1 | ROS proto-oncogene 1, receptor tyrosine kinase | Plasma Membrane | kinase |
| CDDP 16 | STK36 | serine/threonine kinase 36 | Cytoplasm | kinase |
| CDDP 16 | TAOK1 | TAO kinase 1 | Cytoplasm | kinase |
| CDDP 16 | TNK2 | tyrosine kinase non receptor 2 | Cytoplasm | kinase |
| CDDP 17 | CSNK1E | casein kinase 1 epsilon | Cytoplasm | kinase |
| CDDP 17 | CHEK1 | checkpoint kinase 1 | Nucleus | kinase |
| CDDP 17 | GRK1 | G protein-coupled receptor kinase 1 | Plasma Membrane | kinase |
| CDDP 17 | EIF2AK1 | eukaryotic translation initiation factor 2 alpha kinase 1 | Cytoplasm | kinase |
| CDDP 17 | INSRR | insulin receptor related receptor | Plasma Membrane | kinase |
| CDDP 17 | MKNK1 | MAPK interacting serine/threonine kinase 1 | Cytoplasm | kinase |
| CDDP 17 | MAPK14 | mitogen-activated protein kinase 14 | Cytoplasm | kinase |
| CDDP 18 | ALK | ALK receptor tyrosine kinase | Plasma Membrane | kinase |
| CDDP 18 | BLK | BLK proto-oncogene, Src family tyrosine kinase | Cytoplasm | kinase |
| CDDP 18 | BTK | Bruton tyrosine kinase | Cytoplasm | kinase |
| CDDP 18 | CSF1R | colony stimulating factor 1 receptor | Plasma Membrane | kinase |
| CDDP 18 | CSNK1E | casein kinase 1 epsilon | Cytoplasm | kinase |
| CDDP 18 | CHEK1 | checkpoint kinase 1 | Nucleus | kinase |
| CDDP 18 | EPHB3 | EPH receptor B3 | Plasma Membrane | kinase |
| CDDP 18 | FRK | fyn related Src family tyrosine kinase | Nucleus | kinase |
| CDDP 18 | GAK | cyclin G associated kinase | Nucleus | kinase |
| CDDP 18 | GRK1 | G protein-coupled receptor kinase 1 | Plasma Membrane | kinase |
| CDDP 18 | HCK | HCK proto-oncogene, Src family tyrosine kinase | Cytoplasm | kinase |
| CDDP 18 | EIF2AK1 | eukaryotic translation initiation factor 2 alpha kinase 1 | Cytoplasm | kinase |
| CDDP 18 | IGF1R | insulin like growth factor 1 receptor | Plasma Membrane | transmembrane receptor |
| CDDP 18 | INSR | insulin receptor | Plasma Membrane | kinase |
| CDDP 18 | MAPK9 | mitogen-activated protein kinase 9 | Cytoplasm | kinase |
| CDDP 18 | MAPK10 | mitogen-activated protein kinase 10 | Cytoplasm | kinase |
| CDDP 18 | LCK | LCK proto-oncogene, Src family tyrosine kinase | Cytoplasm | kinase |
| CDDP 18 | LTK | leukocyte receptor tyrosine kinase | Plasma Membrane | kinase |
| CDDP 18 | LYN | LYN proto-oncogene, Src family tyrosine kinase | Cytoplasm | kinase |
| CDDP 18 | MAP3K15 | mitogen-activated protein kinase kinase kinase 15 | Other | other |
| CDDP 18 | MELK | maternal embryonic leucine zipper kinase | Cytoplasm | kinase |
| CDDP 18 | RET | ret proto-oncogene | Plasma Membrane | kinase |
| CDDP 18 | SRC | SRC proto-oncogene, non-receptor tyrosine kinase | Cytoplasm | kinase |
| CDDP 18 | STK36 | serine/threonine kinase 36 | Cytoplasm | kinase |
| CDDP 18 | TNK2 | tyrosine kinase non receptor 2 | Cytoplasm | kinase |
| CDDP 18 | MAPK14 | mitogen-activated protein kinase 14 | Cytoplasm | kinase |
| CDDP 19 | ALK | ALK receptor tyrosine kinase | Plasma Membrane | kinase |
| CDDP 19 | CSF1R | colony stimulating factor 1 receptor | Plasma Membrane | kinase |
| CDDP 19 | CSNK1E | casein kinase 1 epsilon | Cytoplasm | kinase |
| CDDP 19 | CHEK1 | checkpoint kinase 1 | Nucleus | kinase |
| CDDP 19 | GAK | cyclin G associated kinase | Nucleus | kinase |
| CDDP 19 | GRK1 | G protein-coupled receptor kinase 1 | Plasma Membrane | kinase |
| CDDP 19 | EIF2AK1 | eukaryotic translation initiation factor 2 alpha kinase 1 | Cytoplasm | kinase |
| CDDP 19 | IGF1R | insulin like growth factor 1 receptor | Plasma Membrane | transmembrane receptor |
| CDDP 19 | MAPK9 | mitogen-activated protein kinase 9 | Cytoplasm | kinase |
| CDDP 19 | MAPK10 | mitogen-activated protein kinase 10 | Cytoplasm | kinase |
| CDDP 19 | LCK | LCK proto-oncogene, Src family tyrosine kinase | Cytoplasm | kinase |
| CDDP 19 | LTK | leukocyte receptor tyrosine kinase | Plasma Membrane | kinase |
| CDDP 19 | MAP3K15 | mitogen-activated protein kinase kinase kinase 15 | Other | other |
| CDDP 19 | MELK | maternal embryonic leucine zipper kinase | Cytoplasm | kinase |
| CDDP 19 | STK36 | serine/threonine kinase 36 | Cytoplasm | kinase |
| CDDP 19 | TNK2 | tyrosine kinase non receptor 2 | Cytoplasm | kinase |
| CDDP 20 | CDK15 | cyclin dependent kinase 15 | Plasma Membrane | kinase |
| CDDP 20 | AURKC | aurora kinase C | Nucleus | kinase |
| CDDP 20 | BMPR1A | bone morphogenetic protein receptor type 1A | Plasma Membrane | kinase |
| CDDP 20 | CAMKK2 | calcium/calmodulin dependent protein kinase kinase 2 | Cytoplasm | kinase |
| CDDP 20 | CDC7 | cell division cycle 7 | Nucleus | kinase |
| CDDP 20 | CDK3 | cyclin dependent kinase 3 | Other | kinase |
| CDDP 20 | CDK5 | cyclin dependent kinase 5 | Nucleus | kinase |
| CDDP 20 | CDK9 | cyclin dependent kinase 9 | Nucleus | kinase |
| CDDP 20 | CDKL2 | cyclin dependent kinase like 2 | Nucleus | kinase |
| CDDP 20 | CDKL3 | cyclin dependent kinase like 3 | Cytoplasm | kinase |
| CDDP 20 | CSNK1A1 | casein kinase 1 alpha 1 | Cytoplasm | kinase |
| CDDP 20 | CSNK1D | casein kinase 1 delta | Cytoplasm | kinase |
| CDDP 20 | CSNK1G1 | casein kinase 1 gamma 1 | Cytoplasm | kinase |
| CDDP 20 | CSNK2A1 | casein kinase 2 alpha 1 | Nucleus | kinase |
| CDDP 20 | CSNK2A2 | casein kinase 2 alpha 2 | Cytoplasm | kinase |
| CDDP 20 | DAPK3 | death associated protein kinase 3 | Cytoplasm | kinase |
| CDDP 20 | DCLK2 | doublecortin like kinase 2 | Cytoplasm | kinase |
| CDDP 20 | EPHB6 | EPH receptor B6 | Plasma Membrane | kinase |
| CDDP 20 | MAPK12 | mitogen-activated protein kinase 12 | Cytoplasm | kinase |
| CDDP 20 | MAPK15 | mitogen-activated protein kinase 15 | Cytoplasm | kinase |
| CDDP 20 | GRK1 | G protein-coupled receptor kinase 1 | Plasma Membrane | kinase |
| CDDP 20 | HIPK3 | homeodomain interacting protein kinase 3 | Nucleus | kinase |
| CDDP 20 | EIF2AK1 | eukaryotic translation initiation factor 2 alpha kinase 1 | Cytoplasm | kinase |
| CDDP 20 | LIMK1 | LIM domain kinase 1 | Cytoplasm | kinase |
| CDDP 20 | MAP3K15 | mitogen-activated protein kinase kinase kinase 15 | Other | other |
| CDDP 20 | MAP2K1 | mitogen-activated protein kinase kinase 1 | Cytoplasm | kinase |
| CDDP 20 | MAP2K7 | mitogen-activated protein kinase kinase 7 | Cytoplasm | kinase |
| CDDP 20 | MKNK1 | MAPK interacting serine/threonine kinase 1 | Cytoplasm | kinase |
| CDDP 20 | MKNK2 | MAPK interacting serine/threonine kinase 2 | Cytoplasm | kinase |
| CDDP 20 | MUSK | muscle associated receptor tyrosine kinase | Plasma Membrane | kinase |
| CDDP 20 | PASK | PAS domain containing serine/threonine kinase | Cytoplasm | kinase |
| CDDP 20 | CDK11B | cyclin dependent kinase 11B | Nucleus | kinase |
| CDDP 20 | PLK4 | polo like kinase 4 | Cytoplasm | kinase |
| CDDP 20 | RIPK1 | receptor interacting serine/threonine kinase 1 | Plasma Membrane | kinase |
| CDDP 20 | ROS1 | ROS proto-oncogene 1, receptor tyrosine kinase | Plasma Membrane | kinase |
| CDDP 20 | SIK2 | salt inducible kinase 2 | Cytoplasm | kinase |
| CDDP 20 | STK17A | serine/threonine kinase 17a | Nucleus | kinase |
| CDDP 20 | STK36 | serine/threonine kinase 36 | Cytoplasm | kinase |
| CDDP 20 | SYK | spleen associated tyrosine kinase | Cytoplasm | kinase |
| CDDP 20 | TGFBR1 | transforming growth factor beta receptor 1 | Plasma Membrane | kinase |
| CDDP 21 | CDK15 | cyclin dependent kinase 15 | Plasma Membrane | kinase |
| CDDP 21 | AURKC | aurora kinase C | Nucleus | kinase |
| CDDP 21 | BMPR1A | bone morphogenetic protein receptor type 1A | Plasma Membrane | kinase |
| CDDP 21 | CAMKK2 | calcium/calmodulin dependent protein kinase kinase 2 | Cytoplasm | kinase |
| CDDP 21 | CDC7 | cell division cycle 7 | Nucleus | kinase |
| CDDP 21 | CDK3 | cyclin dependent kinase 3 | Other | kinase |
| CDDP 21 | CDK5 | cyclin dependent kinase 5 | Nucleus | kinase |
| CDDP 21 | CDK9 | cyclin dependent kinase 9 | Nucleus | kinase |
| CDDP 21 | CDKL2 | cyclin dependent kinase like 2 | Nucleus | kinase |
| CDDP 21 | CDKL3 | cyclin dependent kinase like 3 | Cytoplasm | kinase |
| CDDP 21 | CSNK1A1 | casein kinase 1 alpha 1 | Cytoplasm | kinase |
| CDDP 21 | CSNK1D | casein kinase 1 delta | Cytoplasm | kinase |
| CDDP 21 | CSNK1G1 | casein kinase 1 gamma 1 | Cytoplasm | kinase |
| CDDP 21 | CSNK2A1 | casein kinase 2 alpha 1 | Nucleus | kinase |
| CDDP 21 | CSNK2A2 | casein kinase 2 alpha 2 | Cytoplasm | kinase |
| CDDP 21 | DAPK3 | death associated protein kinase 3 | Cytoplasm | kinase |
| CDDP 21 | DCLK2 | doublecortin like kinase 2 | Cytoplasm | kinase |
| CDDP 21 | EPHB6 | EPH receptor B6 | Plasma Membrane | kinase |
| CDDP 21 | MAPK12 | mitogen-activated protein kinase 12 | Cytoplasm | kinase |
| CDDP 21 | MAPK15 | mitogen-activated protein kinase 15 | Cytoplasm | kinase |
| CDDP 21 | GRK1 | G protein-coupled receptor kinase 1 | Plasma Membrane | kinase |
| CDDP 21 | HIPK3 | homeodomain interacting protein kinase 3 | Nucleus | kinase |
| CDDP 21 | EIF2AK1 | eukaryotic translation initiation factor 2 alpha kinase 1 | Cytoplasm | kinase |
| CDDP 21 | LIMK1 | LIM domain kinase 1 | Cytoplasm | kinase |
| CDDP 21 | MAP3K15 | mitogen-activated protein kinase kinase kinase 15 | Other | other |
| CDDP 21 | MAP2K1 | mitogen-activated protein kinase kinase 1 | Cytoplasm | kinase |
| CDDP 21 | MAP2K7 | mitogen-activated protein kinase kinase 7 | Cytoplasm | kinase |
| CDDP 21 | MKNK1 | MAPK interacting serine/threonine kinase 1 | Cytoplasm | kinase |
| CDDP 21 | MKNK2 | MAPK interacting serine/threonine kinase 2 | Cytoplasm | kinase |
| CDDP 21 | MUSK | muscle associated receptor tyrosine kinase | Plasma Membrane | kinase |
| CDDP 21 | PASK | PAS domain containing serine/threonine kinase | Cytoplasm | kinase |
| CDDP 21 | CDK11B | cyclin dependent kinase 11B | Nucleus | kinase |
| CDDP 21 | PLK4 | polo like kinase 4 | Cytoplasm | kinase |
| CDDP 21 | RIPK1 | receptor interacting serine/threonine kinase 1 | Plasma Membrane | kinase |
| CDDP 21 | ROS1 | ROS proto-oncogene 1, receptor tyrosine kinase | Plasma Membrane | kinase |
| CDDP 21 | SIK2 | salt inducible kinase 2 | Cytoplasm | kinase |
| CDDP 21 | STK17A | serine/threonine kinase 17a | Nucleus | kinase |
| CDDP 21 | STK36 | serine/threonine kinase 36 | Cytoplasm | kinase |
| CDDP 21 | SYK | spleen associated tyrosine kinase | Cytoplasm | kinase |
| CDDP 21 | TGFBR1 | transforming growth factor beta receptor 1 | Plasma Membrane | kinase |
| CDDP 22 | ALK | ALK receptor tyrosine kinase | Plasma Membrane | kinase |
| CDDP 22 | ACVR1 | activin A receptor type 1 | Plasma Membrane | kinase |
| CDDP 22 | ACVR1B | activin A receptor type 1B | Plasma Membrane | kinase |
| CDDP 22 | AURKA | aurora kinase A | Nucleus | kinase |
| CDDP 22 | AURKB | aurora kinase B | Nucleus | kinase |
| CDDP 22 | AURKC | aurora kinase C | Nucleus | kinase |
| CDDP 22 | AXL | AXL receptor tyrosine kinase | Plasma Membrane | kinase |
| CDDP 22 | BLK | BLK proto-oncogene, Src family tyrosine kinase | Cytoplasm | kinase |
| CDDP 22 | BMP2K | BMP2 inducible kinase | Nucleus | kinase |
| CDDP 22 | BMPR1B | bone morphogenetic protein receptor type 1B | Plasma Membrane | kinase |
| CDDP 22 | BRSK1 | BR serine/threonine kinase 1 | Cytoplasm | kinase |
| CDDP 22 | BTK | Bruton tyrosine kinase | Cytoplasm | kinase |
| CDDP 22 | CAMK1D | calcium/calmodulin dependent protein kinase ID | Cytoplasm | kinase |
| CDDP 22 | CAMK2D | calcium/calmodulin dependent protein kinase II delta | Cytoplasm | kinase |
| CDDP 22 | CAMKK1 | calcium/calmodulin dependent protein kinase kinase 1 | Cytoplasm | kinase |
| CDDP 22 | CAMKK2 | calcium/calmodulin dependent protein kinase kinase 2 | Cytoplasm | kinase |
| CDDP 22 | CDC7 | cell division cycle 7 | Nucleus | kinase |
| CDDP 22 | CDK3 | cyclin dependent kinase 3 | Other | kinase |
| CDDP 22 | CDK5 | cyclin dependent kinase 5 | Nucleus | kinase |
| CDDP 22 | CDK8 | cyclin dependent kinase 8 | Nucleus | kinase |
| CDDP 22 | CDK9 | cyclin dependent kinase 9 | Nucleus | kinase |
| CDDP 22 | CDKL2 | cyclin dependent kinase like 2 | Nucleus | kinase |
| CDDP 22 | CDKL3 | cyclin dependent kinase like 3 | Cytoplasm | kinase |
| CDDP 22 | CDKL5 | cyclin dependent kinase like 5 | Nucleus | kinase |
| CDDP 22 | CHUK | component of inhibitor of nuclear factor kappa B kinase complex | Cytoplasm | kinase |
| CDDP 22 | CLK2 | CDC like kinase 2 | Nucleus | kinase |
| CDDP 22 | CLK4 | CDC like kinase 4 | Nucleus | kinase |
| CDDP 22 | CSNK1A1 | casein kinase 1 alpha 1 | Cytoplasm | kinase |
| CDDP 22 | CSNK1E | casein kinase 1 epsilon | Cytoplasm | kinase |
| CDDP 22 | CSNK1G2 | casein kinase 1 gamma 2 | Cytoplasm | kinase |
| CDDP 22 | CSNK2A1 | casein kinase 2 alpha 1 | Nucleus | kinase |
| CDDP 22 | CSNK2A2 | casein kinase 2 alpha 2 | Cytoplasm | kinase |
| CDDP 22 | CHEK1 | checkpoint kinase 1 | Nucleus | kinase |
| CDDP 22 | DAPK3 | death associated protein kinase 3 | Cytoplasm | kinase |
| CDDP 22 | DYRK1A | dual specificity tyrosine phosphorylation regulated kinase 1A | Nucleus | kinase |
| CDDP 22 | DYRK1B | dual specificity tyrosine phosphorylation regulated kinase 1B | Nucleus | kinase |
| CDDP 22 | DYRK3 | dual specificity tyrosine phosphorylation regulated kinase 3 | Nucleus | kinase |
| CDDP 22 | DYRK4 | dual specificity tyrosine phosphorylation regulated kinase 4 | Nucleus | kinase |
| CDDP 22 | EIF2AK2 | eukaryotic translation initiation factor 2 alpha kinase 2 | Cytoplasm | kinase |
| CDDP 22 | EPHA1 | EPH receptor A1 | Plasma Membrane | kinase |
| CDDP 22 | EPHA4 | EPH receptor A4 | Plasma Membrane | kinase |
| CDDP 22 | EPHA7 | EPH receptor A7 | Plasma Membrane | kinase |
| CDDP 22 | EPHB6 | EPH receptor B6 | Plasma Membrane | kinase |
| CDDP 22 | MAPK1 | mitogen-activated protein kinase 1 | Cytoplasm | kinase |
| CDDP 22 | MAPK7 | mitogen-activated protein kinase 7 | Cytoplasm | kinase |
| CDDP 22 | PTK2 | protein tyrosine kinase 2 | Cytoplasm | kinase |
| CDDP 22 | FER | FER tyrosine kinase | Cytoplasm | kinase |
| CDDP 22 | FES | FES proto-oncogene, tyrosine kinase | Cytoplasm | kinase |
| CDDP 22 | FGFR1 | fibroblast growth factor receptor 1 | Plasma Membrane | kinase |
| CDDP 22 | FGFR3 | fibroblast growth factor receptor 3 | Plasma Membrane | kinase |
| CDDP 22 | FGR | FGR proto-oncogene, Src family tyrosine kinase | Nucleus | kinase |
| CDDP 22 | GAK | cyclin G associated kinase | Nucleus | kinase |
| CDDP 22 | GRK1 | G protein-coupled receptor kinase 1 | Plasma Membrane | kinase |
| CDDP 22 | GRK4 | G protein-coupled receptor kinase 4 | Plasma Membrane | kinase |
| CDDP 22 | GRK5 | G protein-coupled receptor kinase 5 | Plasma Membrane | kinase |
| CDDP 22 | GRK7 | G protein-coupled receptor kinase 7 | Cytoplasm | kinase |
| CDDP 22 | HCK | HCK proto-oncogene, Src family tyrosine kinase | Cytoplasm | kinase |
| CDDP 22 | HIPK3 | homeodomain interacting protein kinase 3 | Nucleus | kinase |
| CDDP 22 | EIF2AK1 | eukaryotic translation initiation factor 2 alpha kinase 1 | Cytoplasm | kinase |
| CDDP 22 | CILK1 | ciliogenesis Associated Kinase 1 | Nucleus | kinase |
| CDDP 22 | IGF1R | insulin like growth factor 1 receptor | Plasma Membrane | transmembrane receptor |
| CDDP 22 | IKBKE | inhibitor of nuclear factor kappa B kinase subunit epsilon | Cytoplasm | kinase |
| CDDP 22 | INSR | insulin receptor | Plasma Membrane | kinase |
| CDDP 22 | IRAK1 | interleukin 1 receptor associated kinase 1 | Plasma Membrane | kinase |
| CDDP 22 | IRAK4 | interleukin 1 receptor associated kinase 4 | Cytoplasm | kinase |
| CDDP 22 | JAK2 | Janus kinase 2 | Cytoplasm | kinase |
| CDDP 22 | JAK3 | Janus kinase 3 | Cytoplasm | kinase |
| CDDP 22 | MAPK8 | mitogen-activated protein kinase 8 | Cytoplasm | kinase |
| CDDP 22 | MAPK9 | mitogen-activated protein kinase 9 | Cytoplasm | kinase |
| CDDP 22 | MAPK10 | mitogen-activated protein kinase 10 | Cytoplasm | kinase |
| CDDP 22 | LATS1 | large tumor suppressor kinase 1 | Nucleus | kinase |
| CDDP 22 | LIMK1 | LIM domain kinase 1 | Cytoplasm | kinase |
| CDDP 22 | LRRK2 | leucine rich repeat kinase 2 | Cytoplasm | kinase |
| CDDP 22 | LTK | leukocyte receptor tyrosine kinase | Plasma Membrane | kinase |
| CDDP 22 | MAP3K10 | mitogen-activated protein kinase kinase kinase 10 | Cytoplasm | kinase |
| CDDP 22 | MAP3K13 | mitogen-activated protein kinase kinase kinase 13 | Cytoplasm | kinase |
| CDDP 22 | MAP3K2 | mitogen-activated protein kinase kinase kinase 2 | Cytoplasm | kinase |
| CDDP 22 | MAP3K3 | mitogen-activated protein kinase kinase kinase 3 | Cytoplasm | kinase |
| CDDP 22 | MAP3K6 | mitogen-activated protein kinase kinase kinase 6 | Other | kinase |
| CDDP 22 | MAP3K9 | mitogen-activated protein kinase kinase kinase 9 | Cytoplasm | kinase |
| CDDP 22 | MAP4K2 | mitogen-activated protein kinase kinase kinase kinase 2 | Cytoplasm | kinase |
| CDDP 22 | MAP4K3 | mitogen-activated protein kinase kinase kinase kinase 3 | Other | kinase |
| CDDP 22 | MAP4K4 | mitogen-activated protein kinase kinase kinase kinase 4 | Cytoplasm | kinase |
| CDDP 22 | MAP4K5 | mitogen-activated protein kinase kinase kinase kinase 5 | Cytoplasm | kinase |
| CDDP 22 | MARK2 | microtubule affinity regulating kinase 2 | Cytoplasm | kinase |
| CDDP 22 | MARK3 | microtubule affinity regulating kinase 3 | Cytoplasm | kinase |
| CDDP 22 | MARK4 | microtubule affinity regulating kinase 4 | Cytoplasm | kinase |
| CDDP 22 | MAST1 | microtubule associated serine/threonine kinase 1 | Cytoplasm | kinase |
| CDDP 22 | MELK | maternal embryonic leucine zipper kinase | Cytoplasm | kinase |
| CDDP 22 | MET | MET proto-oncogene, receptor tyrosine kinase | Plasma Membrane | kinase |
| CDDP 22 | MAP2K3 | mitogen-activated protein kinase kinase 3 | Cytoplasm | kinase |
| CDDP 22 | MAP2K4 | mitogen-activated protein kinase kinase 4 | Cytoplasm | kinase |
| CDDP 22 | MKNK1 | MAPK interacting serine/threonine kinase 1 | Cytoplasm | kinase |
| CDDP 22 | MKNK2 | MAPK interacting serine/threonine kinase 2 | Cytoplasm | kinase |
| CDDP 22 | SRPK3 | SRSF protein kinase 3 | Cytoplasm | kinase |
| CDDP 22 | MST1R | macrophage stimulating 1 receptor | Plasma Membrane | kinase |
| CDDP 22 | MYLK2 | myosin light chain kinase 2 | Cytoplasm | kinase |
| CDDP 22 | NEK2 | NIMA related kinase 2 | Cytoplasm | kinase |
| CDDP 22 | NEK6 | NIMA related kinase 6 | Nucleus | kinase |
| CDDP 22 | NLK | nemo like kinase | Nucleus | kinase |
| CDDP 22 | NTRK3 | neurotrophic receptor tyrosine kinase 3 | Plasma Membrane | kinase |
| CDDP 22 | OXSR1 | oxidative stress responsive kinase 1 | Nucleus | kinase |
| CDDP 22 | PAK2 | p21 (RAC1) activated kinase 2 | Cytoplasm | kinase |
| CDDP 22 | PAK5 | p21 (RAC1) activated kinase 5 | Nucleus | kinase |
| CDDP 22 | PASK | PAS domain containing serine/threonine kinase | Cytoplasm | kinase |
| CDDP 22 | PBK | PDZ binding kinase | Cytoplasm | kinase |
| CDDP 22 | CDK17 | cyclin dependent kinase 17 | Cytoplasm | kinase |
| CDDP 22 | PHKG1 | phosphorylase kinase catalytic subunit gamma 1 | Cytoplasm | kinase |
| CDDP 22 | PHKG2 | phosphorylase kinase catalytic subunit gamma 2 | Cytoplasm | kinase |
| CDDP 22 | PIM1 | Pim-1 proto-oncogene, serine/threonine kinase | Cytoplasm | kinase |
| CDDP 22 | PIM2 | Pim-2 proto-oncogene, serine/threonine kinase | Nucleus | kinase |
| CDDP 22 | PIM3 | Pim-3 proto-oncogene, serine/threonine kinase | Cytoplasm | kinase |
| CDDP 22 | PKD2 | polycystin 2, transient receptor potential cation channel | Plasma Membrane | kinase |
| CDDP 22 | PKD3 | polycystic Kidney Disease 3 (Autosomal Dominant) | Plasma Membrane | kinase |
| CDDP 22 | PKN2 | protein kinase N2 | Cytoplasm | kinase |
| CDDP 22 | PLK2 | polo like kinase 2 | Nucleus | kinase |
| CDDP 22 | PLK4 | polo like kinase 4 | Cytoplasm | kinase |
| CDDP 22 | PRKAA1 | protein kinase AMP-activated catalytic subunit alpha 1 | Cytoplasm | kinase |
| CDDP 22 | PRKX | protein kinase X-linked | Cytoplasm | kinase |
| CDDP 22 | SIK3 | SIK family kinase 3 | Cytoplasm | kinase |
| CDDP 22 | RIPK1 | receptor interacting serine/threonine kinase 1 | Plasma Membrane | kinase |
| CDDP 22 | ROS1 | ROS proto-oncogene 1, receptor tyrosine kinase | Plasma Membrane | kinase |
| CDDP 22 | SGK1 | serum/glucocorticoid regulated kinase 1 | Cytoplasm | kinase |
| CDDP 22 | SIK2 | salt inducible kinase 2 | Cytoplasm | kinase |
| CDDP 22 | SRPK1 | SRSF protein kinase 1 | Nucleus | kinase |
| CDDP 22 | STK11 | serine/threonine kinase 11 | Cytoplasm | kinase |
| CDDP 22 | STK16 | serine/threonine kinase 16 | Cytoplasm | kinase |
| CDDP 22 | STK17A | serine/threonine kinase 17a | Nucleus | kinase |
| CDDP 22 | STK17B | serine/threonine kinase 17b | Nucleus | kinase |
| CDDP 22 | STK24 | serine/threonine kinase 24 | Cytoplasm | kinase |
| CDDP 22 | STK25 | serine/threonine kinase 25 | Cytoplasm | kinase |
| CDDP 22 | STK33 | serine/threonine kinase 33 | Cytoplasm | kinase |
| CDDP 22 | STK35 | serine/threonine kinase 35 | Cytoplasm | kinase |
| CDDP 22 | STK38L | serine/threonine kinase 38 like | Cytoplasm | kinase |
| CDDP 22 | STK39 | serine/threonine kinase 39 | Nucleus | kinase |
| CDDP 22 | STK4 | serine/threonine kinase 4 | Cytoplasm | kinase |
| CDDP 22 | SYK | spleen associated tyrosine kinase | Cytoplasm | kinase |
| CDDP 22 | SBK3 | SH3 domain binding kinase family member 3 | Other | other |
| CDDP 22 | TAOK1 | TAO kinase 1 | Cytoplasm | kinase |
| CDDP 22 | TBK1 | TANK binding kinase 1 | Cytoplasm | kinase |
| CDDP 22 | TESK1 | testis associated actin remodelling kinase 1 | Nucleus | kinase |
| CDDP 22 | TNK2 | tyrosine kinase non receptor 2 | Cytoplasm | kinase |
| CDDP 22 | TSSK1B | testis specific serine kinase 1B | Cytoplasm | kinase |
| CDDP 22 | TSSK2 | testis specific serine kinase 2 | Cytoplasm | kinase |
| CDDP 22 | TXK | TXK tyrosine kinase | Cytoplasm | kinase |
| CDDP 22 | TYK2 | tyrosine kinase 2 | Plasma Membrane | kinase |
| CDDP 22 | TYRO3 | TYRO3 protein tyrosine kinase | Plasma Membrane | kinase |
| CDDP 22 | ULK1 | unc-51 like autophagy activating kinase 1 | Cytoplasm | kinase |
| CDDP 22 | ULK2 | unc-51 like autophagy activating kinase 2 | Cytoplasm | kinase |
| CDDP 22 | ULK3 | unc-51 like kinase 3 | Cytoplasm | kinase |
| CDDP 22 | YES1 | YES proto-oncogene 1, Src family tyrosine kinase | Cytoplasm | kinase |
| CDDP 23 | CDC7 | cell division cycle 7 | Nucleus | kinase |
| CDDP 23 | CDKL3 | cyclin dependent kinase like 3 | Cytoplasm | kinase |
| CDDP 23 | CLK1 | CDC like kinase 1 | Nucleus | kinase |
| CDDP 23 | CSNK2A1 | casein kinase 2 alpha 1 | Nucleus | kinase |
| CDDP 23 | CSNK2A2 | casein kinase 2 alpha 2 | Cytoplasm | kinase |
| CDDP 23 | CHEK1 | checkpoint kinase 1 | Nucleus | kinase |
| CDDP 23 | DAPK3 | death associated protein kinase 3 | Cytoplasm | kinase |
| CDDP 23 | EPHB6 | EPH receptor B6 | Plasma Membrane | kinase |
| CDDP 23 | FES | FES proto-oncogene, tyrosine kinase | Cytoplasm | kinase |
| CDDP 23 | GAK | cyclin G associated kinase | Nucleus | kinase |
| CDDP 23 | HIPK2 | homeodomain interacting protein kinase 2 | Nucleus | kinase |
| CDDP 23 | HIPK3 | homeodomain interacting protein kinase 3 | Nucleus | kinase |
| CDDP 23 | EIF2AK1 | eukaryotic translation initiation factor 2 alpha kinase 1 | Cytoplasm | kinase |
| CDDP 23 | MAPK10 | mitogen-activated protein kinase 10 | Cytoplasm | kinase |
| CDDP 23 | MAP3K15 | mitogen-activated protein kinase kinase kinase 15 | Other | other |
| CDDP 23 | MAP4K3 | mitogen-activated protein kinase kinase kinase kinase 3 | Other | kinase |
| CDDP 23 | MAPKAPK5 | MAPK activated protein kinase 5 | Cytoplasm | kinase |
| CDDP 23 | MARK4 | microtubule affinity regulating kinase 4 | Cytoplasm | kinase |
| CDDP 23 | MAST1 | microtubule associated serine/threonine kinase 1 | Cytoplasm | kinase |
| CDDP 23 | MKNK1 | MAPK interacting serine/threonine kinase 1 | Cytoplasm | kinase |
| CDDP 23 | MYLK | myosin light chain kinase | Cytoplasm | kinase |
| CDDP 23 | NLK | nemo like kinase | Nucleus | kinase |
| CDDP 23 | PDGFRA | platelet derived growth factor receptor alpha | Plasma Membrane | kinase |
| CDDP 23 | PIM2 | Pim-2 proto-oncogene, serine/threonine kinase | Nucleus | kinase |
| CDDP 23 | PIM3 | Pim-3 proto-oncogene, serine/threonine kinase | Cytoplasm | kinase |
| CDDP 23 | RET | ret proto-oncogene | Plasma Membrane | kinase |
| CDDP 23 | RIPK1 | receptor interacting serine/threonine kinase 1 | Plasma Membrane | kinase |
| CDDP 23 | TGFBR1 | transforming growth factor beta receptor 1 | Plasma Membrane | kinase |
| CDDP 24 | CDC7 | cell division cycle 7 | Nucleus | kinase |
| CDDP 24 | CSNK2A1 | casein kinase 2 alpha 1 | Nucleus | kinase |
| CDDP 24 | CSNK2A2 | casein kinase 2 alpha 2 | Cytoplasm | kinase |
| CDDP 24 | DAPK3 | death associated protein kinase 3 | Cytoplasm | kinase |
| CDDP 24 | EPHB6 | EPH receptor B6 | Plasma Membrane | kinase |
| CDDP 24 | GRK1 | G protein-coupled receptor kinase 1 | Plasma Membrane | kinase |
| CDDP 24 | HIPK3 | homeodomain interacting protein kinase 3 | Nucleus | kinase |
| CDDP 24 | EIF2AK1 | eukaryotic translation initiation factor 2 alpha kinase 1 | Cytoplasm | kinase |
| CDDP 24 | MAPKAPK5 | MAPK activated protein kinase 5 | Cytoplasm | kinase |
| CDDP 24 | MAST1 | microtubule associated serine/threonine kinase 1 | Cytoplasm | kinase |
| CDDP 24 | MKNK1 | MAPK interacting serine/threonine kinase 1 | Cytoplasm | kinase |
| CDDP 24 | PIM2 | Pim-2 proto-oncogene, serine/threonine kinase | Nucleus | kinase |
| CDDP 24 | PIM3 | Pim-3 proto-oncogene, serine/threonine kinase | Cytoplasm | kinase |
| CDDP 24 | RIPK1 | receptor interacting serine/threonine kinase 1 | Plasma Membrane | kinase |
| CDDP 24 | WEE1 | WEE1 G2 checkpoint kinase | Nucleus | kinase |
| CDDP 25 | AURKC | aurora kinase C | Nucleus | kinase |
| CDDP 25 | BLK | BLK proto-oncogene, Src family tyrosine kinase | Cytoplasm | kinase |
| CDDP 25 | BMPR1B | bone morphogenetic protein receptor type 1B | Plasma Membrane | kinase |
| CDDP 25 | BTK | Bruton tyrosine kinase | Cytoplasm | kinase |
| CDDP 25 | CAMKK1 | calcium/calmodulin dependent protein kinase kinase 1 | Cytoplasm | kinase |
| CDDP 25 | CDK5 | cyclin dependent kinase 5 | Nucleus | kinase |
| CDDP 25 | CDK8 | cyclin dependent kinase 8 | Nucleus | kinase |
| CDDP 25 | CDK9 | cyclin dependent kinase 9 | Nucleus | kinase |
| CDDP 25 | CDKL2 | cyclin dependent kinase like 2 | Nucleus | kinase |
| CDDP 25 | CDKL3 | cyclin dependent kinase like 3 | Cytoplasm | kinase |
| CDDP 25 | CLK4 | CDC like kinase 4 | Nucleus | kinase |
| CDDP 25 | CSNK1A1 | casein kinase 1 alpha 1 | Cytoplasm | kinase |
| CDDP 25 | CSNK1D | casein kinase 1 delta | Cytoplasm | kinase |
| CDDP 25 | CSNK1E | casein kinase 1 epsilon | Cytoplasm | kinase |
| CDDP 25 | CSNK1G1 | casein kinase 1 gamma 1 | Cytoplasm | kinase |
| CDDP 25 | CSNK1G2 | casein kinase 1 gamma 2 | Cytoplasm | kinase |
| CDDP 25 | CSNK1G3 | casein kinase 1 gamma 3 | Cytoplasm | kinase |
| CDDP 25 | DDR1 | discoidin domain receptor tyrosine kinase 1 | Plasma Membrane | kinase |
| CDDP 25 | DYRK1A | dual specificity tyrosine phosphorylation regulated kinase 1A | Nucleus | kinase |
| CDDP 25 | DYRK1B | dual specificity tyrosine phosphorylation regulated kinase 1B | Nucleus | kinase |
| CDDP 25 | DYRK3 | dual specificity tyrosine phosphorylation regulated kinase 3 | Nucleus | kinase |
| CDDP 25 | EGFR | epidermal growth factor receptor | Plasma Membrane | kinase |
| CDDP 25 | EPHA3 | EPH receptor A3 | Plasma Membrane | kinase |
| CDDP 25 | EPHB6 | EPH receptor B6 | Plasma Membrane | kinase |
| CDDP 25 | MAPK7 | mitogen-activated protein kinase 7 | Cytoplasm | kinase |
| CDDP 25 | FGFR1 | fibroblast growth factor receptor 1 | Plasma Membrane | kinase |
| CDDP 25 | GSK3B | glycogen synthase kinase 3 beta | Nucleus | kinase |
| CDDP 25 | ERBB2 | erb-b2 receptor tyrosine kinase 2 | Plasma Membrane | kinase |
| CDDP 25 | ERBB4 | erb-b2 receptor tyrosine kinase 4 | Plasma Membrane | kinase |
| CDDP 25 | HIPK3 | homeodomain interacting protein kinase 3 | Nucleus | kinase |
| CDDP 25 | EIF2AK1 | eukaryotic translation initiation factor 2 alpha kinase 1 | Cytoplasm | kinase |
| CDDP 25 | CILK1 | ciliogenesis Associated Kinase 1 | Nucleus | kinase |
| CDDP 25 | MAPK8 | mitogen-activated protein kinase 8 | Cytoplasm | kinase |
| CDDP 25 | MAPK9 | mitogen-activated protein kinase 9 | Cytoplasm | kinase |
| CDDP 25 | MAPK10 | mitogen-activated protein kinase 10 | Cytoplasm | kinase |
| CDDP 25 | LIMK1 | LIM domain kinase 1 | Cytoplasm | kinase |
| CDDP 25 | LTK | leukocyte receptor tyrosine kinase | Plasma Membrane | kinase |
| CDDP 25 | MAP3K9 | mitogen-activated protein kinase kinase kinase 9 | Cytoplasm | kinase |
| CDDP 25 | MAP4K4 | mitogen-activated protein kinase kinase kinase kinase 4 | Cytoplasm | kinase |
| CDDP 25 | MAP4K5 | mitogen-activated protein kinase kinase kinase kinase 5 | Cytoplasm | kinase |
| CDDP 25 | MAPKAPK5 | MAPK activated protein kinase 5 | Cytoplasm | kinase |
| CDDP 25 | MAST1 | microtubule associated serine/threonine kinase 1 | Cytoplasm | kinase |
| CDDP 25 | MATK | megakaryocyte-associated tyrosine kinase | Cytoplasm | kinase |
| CDDP 25 | MAP2K2 | mitogen-activated protein kinase kinase 2 | Cytoplasm | kinase |
| CDDP 25 | MET | MET proto-oncogene, receptor tyrosine kinase | Plasma Membrane | kinase |
| CDDP 25 | NEK2 | NIMA related kinase 2 | Cytoplasm | kinase |
| CDDP 25 | EIF2AK3 | eukaryotic translation initiation factor 2 alpha kinase 3 | Cytoplasm | kinase |
| CDDP 25 | PIM1 | Pim-1 proto-oncogene, serine/threonine kinase | Cytoplasm | kinase |
| CDDP 25 | PRKCB | protein kinase C beta | Cytoplasm | kinase |
| CDDP 25 | PKD2 | polycystin 2, transient receptor potential cation channel | Plasma Membrane | kinase |
| CDDP 25 | PKD3 | polycystic Kidney Disease 3 (Autosomal Dominant) | Plasma Membrane | kinase |
| CDDP 25 | PLK3 | polo like kinase 3 | Nucleus | kinase |
| CDDP 25 | SYK | spleen associated tyrosine kinase | Cytoplasm | kinase |
| CDDP 25 | TAOK1 | TAO kinase 1 | Cytoplasm | kinase |
| CDDP 25 | TXK | TXK tyrosine kinase | Cytoplasm | kinase |
| CDDP 25 | TYRO3 | TYRO3 protein tyrosine kinase | Plasma Membrane | kinase |
| CDDP 25 | MAP3K20 | mitogen-activated protein kinase kinase kinase 20 | Cytoplasm | kinase |
| CDDP 25 | ZAP70 | zeta chain of T cell receptor associated protein kinase 70 | Plasma Membrane | kinase |
| CDDP 26 | CDK15 | cyclin dependent kinase 15 | Plasma Membrane | kinase |
| CDDP 26 | AXL | AXL receptor tyrosine kinase | Plasma Membrane | kinase |
| CDDP 26 | BLK | BLK proto-oncogene, Src family tyrosine kinase | Cytoplasm | kinase |
| CDDP 26 | BTK | Bruton tyrosine kinase | Cytoplasm | kinase |
| CDDP 26 | CDKL2 | cyclin dependent kinase like 2 | Nucleus | kinase |
| CDDP 26 | CDKL3 | cyclin dependent kinase like 3 | Cytoplasm | kinase |
| CDDP 26 | CSF1R | colony stimulating factor 1 receptor | Plasma Membrane | kinase |
| CDDP 26 | CSNK1E | casein kinase 1 epsilon | Cytoplasm | kinase |
| CDDP 26 | CHEK1 | checkpoint kinase 1 | Nucleus | kinase |
| CDDP 26 | EPHA2 | EPH receptor A2 | Plasma Membrane | kinase |
| CDDP 26 | EPHA4 | EPH receptor A4 | Plasma Membrane | kinase |
| CDDP 26 | EPHB2 | EPH receptor B2 | Plasma Membrane | kinase |
| CDDP 26 | EPHB3 | EPH receptor B3 | Plasma Membrane | kinase |
| CDDP 26 | EPHB6 | EPH receptor B6 | Plasma Membrane | kinase |
| CDDP 26 | FGFR1 | fibroblast growth factor receptor 1 | Plasma Membrane | kinase |
| CDDP 26 | FGFR3 | fibroblast growth factor receptor 3 | Plasma Membrane | kinase |
| CDDP 26 | FRK | fyn related Src family tyrosine kinase | Nucleus | kinase |
| CDDP 26 | GAK | cyclin G associated kinase | Nucleus | kinase |
| CDDP 26 | GRK1 | G protein-coupled receptor kinase 1 | Plasma Membrane | kinase |
| CDDP 26 | HCK | HCK proto-oncogene, Src family tyrosine kinase | Cytoplasm | kinase |
| CDDP 26 | HIPK3 | homeodomain interacting protein kinase 3 | Nucleus | kinase |
| CDDP 26 | EIF2AK1 | eukaryotic translation initiation factor 2 alpha kinase 1 | Cytoplasm | kinase |
| CDDP 26 | IGF1R | insulin like growth factor 1 receptor | Plasma Membrane | transmembrane receptor |
| CDDP 26 | MAPK9 | mitogen-activated protein kinase 9 | Cytoplasm | kinase |
| CDDP 26 | MAPK10 | mitogen-activated protein kinase 10 | Cytoplasm | kinase |
| CDDP 26 | LCK | LCK proto-oncogene, Src family tyrosine kinase | Cytoplasm | kinase |
| CDDP 26 | LIMK1 | LIM domain kinase 1 | Cytoplasm | kinase |
| CDDP 26 | LTK | leukocyte receptor tyrosine kinase | Plasma Membrane | kinase |
| CDDP 26 | LYN | LYN proto-oncogene, Src family tyrosine kinase | Cytoplasm | kinase |
| CDDP 26 | MAP3K15 | mitogen-activated protein kinase kinase kinase 15 | Other | other |
| CDDP 26 | MATK | megakaryocyte-associated tyrosine kinase | Cytoplasm | kinase |
| CDDP 26 | MAP2K1 | mitogen-activated protein kinase kinase 1 | Cytoplasm | kinase |
| CDDP 26 | MELK | maternal embryonic leucine zipper kinase | Cytoplasm | kinase |
| CDDP 26 | NLK | nemo like kinase | Nucleus | kinase |
| CDDP 26 | PDGFRA | platelet derived growth factor receptor alpha | Plasma Membrane | kinase |
| CDDP 26 | RAF1 | Raf-1 proto-oncogene, serine/threonine kinase | Cytoplasm | kinase |
| CDDP 26 | RIPK1 | receptor interacting serine/threonine kinase 1 | Plasma Membrane | kinase |
| CDDP 26 | STK36 | serine/threonine kinase 36 | Cytoplasm | kinase |
| CDDP 26 | TAOK1 | TAO kinase 1 | Cytoplasm | kinase |
| CDDP 26 | TNK2 | tyrosine kinase non receptor 2 | Cytoplasm | kinase |
| CDDP 27 | CSNK1E | casein kinase 1 epsilon | Cytoplasm | kinase |
| CDDP 27 | GRK1 | G protein-coupled receptor kinase 1 | Plasma Membrane | kinase |
| CDDP 27 | EIF2AK1 | eukaryotic translation initiation factor 2 alpha kinase 1 | Cytoplasm | kinase |
| CDDP 27 | INSRR | insulin receptor related receptor | Plasma Membrane | kinase |
| CDDP 27 | MELK | maternal embryonic leucine zipper kinase | Cytoplasm | kinase |
| CDDP 27 | MKNK1 | MAPK interacting serine/threonine kinase 1 | Cytoplasm | kinase |
| CDDP 27 | ROS1 | ROS proto-oncogene 1, receptor tyrosine kinase | Plasma Membrane | kinase |
| CDDP 27 | MAPK14 | mitogen-activated protein kinase 14 | Cytoplasm | kinase |
| CDDP 28 | GRK1 | G protein-coupled receptor kinase 1 | Plasma Membrane | kinase |
| CDDP 28 | EIF2AK1 | eukaryotic translation initiation factor 2 alpha kinase 1 | Cytoplasm | kinase |
| CDDP 28 | MAPK10 | mitogen-activated protein kinase 10 | Cytoplasm | kinase |
| CDDP 28 | LTK | leukocyte receptor tyrosine kinase | Plasma Membrane | kinase |
| CDDP 28 | MELK | maternal embryonic leucine zipper kinase | Cytoplasm | kinase |
| CDDP 28 | PIM2 | Pim-2 proto-oncogene, serine/threonine kinase | Nucleus | kinase |
| CDDP 28 | RIPK1 | receptor interacting serine/threonine kinase 1 | Plasma Membrane | kinase |
| CDDP 29 | GRK1 | G protein-coupled receptor kinase 1 | Plasma Membrane | kinase |
| CDDP 29 | EIF2AK1 | eukaryotic translation initiation factor 2 alpha kinase 1 | Cytoplasm | kinase |
| CDDP 29 | IGF1R | insulin like growth factor 1 receptor | Plasma Membrane | transmembrane receptor |
| CDDP 29 | MAPK14 | mitogen-activated protein kinase 14 | Cytoplasm | kinase |
| CDDP 30 | GRK1 | G protein-coupled receptor kinase 1 | Plasma Membrane | kinase |
| CDDP 30 | EIF2AK1 | eukaryotic translation initiation factor 2 alpha kinase 1 | Cytoplasm | kinase |
| CDDP 30 | IGF1R | insulin like growth factor 1 receptor | Plasma Membrane | transmembrane receptor |
| CDDP 30 | MAPK14 | mitogen-activated protein kinase 14 | Cytoplasm | kinase |
| CDDP 31 | CDK15 | cyclin dependent kinase 15 | Plasma Membrane | kinase |
| CDDP 31 | AXL | AXL receptor tyrosine kinase | Plasma Membrane | kinase |
| CDDP 31 | BLK | BLK proto-oncogene, Src family tyrosine kinase | Cytoplasm | kinase |
| CDDP 31 | BTK | Bruton tyrosine kinase | Cytoplasm | kinase |
| CDDP 31 | CDKL2 | cyclin dependent kinase like 2 | Nucleus | kinase |
| CDDP 31 | CDKL3 | cyclin dependent kinase like 3 | Cytoplasm | kinase |
| CDDP 31 | CSF1R | colony stimulating factor 1 receptor | Plasma Membrane | kinase |
| CDDP 31 | EPHA4 | EPH receptor A4 | Plasma Membrane | kinase |
| CDDP 31 | EPHB6 | EPH receptor B6 | Plasma Membrane | kinase |
| CDDP 31 | FGFR3 | fibroblast growth factor receptor 3 | Plasma Membrane | kinase |
| CDDP 31 | FRK | fyn related Src family tyrosine kinase | Nucleus | kinase |
| CDDP 31 | FYN | FYN proto-oncogene, Src family tyrosine kinase | Plasma Membrane | kinase |
| CDDP 31 | GAK | cyclin G associated kinase | Nucleus | kinase |
| CDDP 31 | GRK1 | G protein-coupled receptor kinase 1 | Plasma Membrane | kinase |
| CDDP 31 | HIPK3 | homeodomain interacting protein kinase 3 | Nucleus | kinase |
| CDDP 31 | EIF2AK1 | eukaryotic translation initiation factor 2 alpha kinase 1 | Cytoplasm | kinase |
| CDDP 31 | MAPK9 | mitogen-activated protein kinase 9 | Cytoplasm | kinase |
| CDDP 31 | MAPK10 | mitogen-activated protein kinase 10 | Cytoplasm | kinase |
| CDDP 31 | LCK | LCK proto-oncogene, Src family tyrosine kinase | Cytoplasm | kinase |
| CDDP 31 | LIMK1 | LIM domain kinase 1 | Cytoplasm | kinase |
| CDDP 31 | LTK | leukocyte receptor tyrosine kinase | Plasma Membrane | kinase |
| CDDP 31 | LYN | LYN proto-oncogene, Src family tyrosine kinase | Cytoplasm | kinase |
| CDDP 31 | MAP3K15 | mitogen-activated protein kinase kinase kinase 15 | Other | other |
| CDDP 31 | MAP2K1 | mitogen-activated protein kinase kinase 1 | Cytoplasm | kinase |
| CDDP 31 | MELK | maternal embryonic leucine zipper kinase | Cytoplasm | kinase |
| CDDP 31 | NLK | nemo like kinase | Nucleus | kinase |
| CDDP 31 | PLK3 | polo like kinase 3 | Nucleus | kinase |
| CDDP 31 | RAF1 | Raf-1 proto-oncogene, serine/threonine kinase | Cytoplasm | kinase |
| CDDP 31 | STK36 | serine/threonine kinase 36 | Cytoplasm | kinase |
| CDDP 31 | TAOK1 | TAO kinase 1 | Cytoplasm | kinase |
| CDDP 31 | TNK2 | tyrosine kinase non receptor 2 | Cytoplasm | kinase |
| CDDP 32 | ALK | ALK receptor tyrosine kinase | Plasma Membrane | kinase |
| CDDP 32 | BLK | BLK proto-oncogene, Src family tyrosine kinase | Cytoplasm | kinase |
| CDDP 32 | BTK | Bruton tyrosine kinase | Cytoplasm | kinase |
| CDDP 32 | CDKL2 | cyclin dependent kinase like 2 | Nucleus | kinase |
| CDDP 32 | CDKL3 | cyclin dependent kinase like 3 | Cytoplasm | kinase |
| CDDP 32 | CSF1R | colony stimulating factor 1 receptor | Plasma Membrane | kinase |
| CDDP 32 | CSK | C-terminal Src kinase | Cytoplasm | kinase |
| CDDP 32 | EPHA4 | EPH receptor A4 | Plasma Membrane | kinase |
| CDDP 32 | EPHB2 | EPH receptor B2 | Plasma Membrane | kinase |
| CDDP 32 | EPHB3 | EPH receptor B3 | Plasma Membrane | kinase |
| CDDP 32 | EPHB6 | EPH receptor B6 | Plasma Membrane | kinase |
| CDDP 32 | FRK | fyn related Src family tyrosine kinase | Nucleus | kinase |
| CDDP 32 | FYN | FYN proto-oncogene, Src family tyrosine kinase | Plasma Membrane | kinase |
| CDDP 32 | GAK | cyclin G associated kinase | Nucleus | kinase |
| CDDP 32 | GRK1 | G protein-coupled receptor kinase 1 | Plasma Membrane | kinase |
| CDDP 32 | HCK | HCK proto-oncogene, Src family tyrosine kinase | Cytoplasm | kinase |
| CDDP 32 | HIPK3 | homeodomain interacting protein kinase 3 | Nucleus | kinase |
| CDDP 32 | EIF2AK1 | eukaryotic translation initiation factor 2 alpha kinase 1 | Cytoplasm | kinase |
| CDDP 32 | IGF1R | insulin like growth factor 1 receptor | Plasma Membrane | transmembrane receptor |
| CDDP 32 | INSR | insulin receptor | Plasma Membrane | kinase |
| CDDP 32 | MAPK9 | mitogen-activated protein kinase 9 | Cytoplasm | kinase |
| CDDP 32 | MAPK10 | mitogen-activated protein kinase 10 | Cytoplasm | kinase |
| CDDP 32 | LCK | LCK proto-oncogene, Src family tyrosine kinase | Cytoplasm | kinase |
| CDDP 32 | LIMK1 | LIM domain kinase 1 | Cytoplasm | kinase |
| CDDP 32 | LTK | leukocyte receptor tyrosine kinase | Plasma Membrane | kinase |
| CDDP 32 | LYN | LYN proto-oncogene, Src family tyrosine kinase | Cytoplasm | kinase |
| CDDP 32 | MAP3K15 | mitogen-activated protein kinase kinase kinase 15 | Other | other |
| CDDP 32 | MAP2K1 | mitogen-activated protein kinase kinase 1 | Cytoplasm | kinase |
| CDDP 32 | MELK | maternal embryonic leucine zipper kinase | Cytoplasm | kinase |
| CDDP 32 | PTK6 | protein tyrosine kinase 6 | Cytoplasm | kinase |
| CDDP 32 | RET | ret proto-oncogene | Plasma Membrane | kinase |
| CDDP 32 | SRC | SRC proto-oncogene, non-receptor tyrosine kinase | Cytoplasm | kinase |
| CDDP 32 | STK36 | serine/threonine kinase 36 | Cytoplasm | kinase |
| CDDP 32 | TAOK1 | TAO kinase 1 | Cytoplasm | kinase |
| CDDP 32 | TNK2 | tyrosine kinase non receptor 2 | Cytoplasm | kinase |
| CDDP 32 | YES1 | YES proto-oncogene 1, Src family tyrosine kinase | Cytoplasm | kinase |
| CDDP 32 | MAPK14 | mitogen-activated protein kinase 14 | Cytoplasm | kinase |
| CDDP 33 | CDK15 | cyclin dependent kinase 15 | Plasma Membrane | kinase |
| CDDP 33 | GRK1 | G protein-coupled receptor kinase 1 | Plasma Membrane | kinase |
| CDDP 33 | EIF2AK1 | eukaryotic translation initiation factor 2 alpha kinase 1 | Cytoplasm | kinase |
| CDDP 33 | INSRR | insulin receptor related receptor | Plasma Membrane | kinase |
| CDDP 33 | MAP3K15 | mitogen-activated protein kinase kinase kinase 15 | Other | other |
| CDDP 33 | MKNK1 | MAPK interacting serine/threonine kinase 1 | Cytoplasm | kinase |
| CDDP 33 | ROS1 | ROS proto-oncogene 1, receptor tyrosine kinase | Plasma Membrane | kinase |
| CDDP 33 | STK36 | serine/threonine kinase 36 | Cytoplasm | kinase |
| CDDP 33 | MAPK14 | mitogen-activated protein kinase 14 | Cytoplasm | kinase |
| CDDP 34 | CDK15 | cyclin dependent kinase 15 | Plasma Membrane | kinase |
| CDDP 34 | GRK1 | G protein-coupled receptor kinase 1 | Plasma Membrane | kinase |
| CDDP 34 | EIF2AK1 | eukaryotic translation initiation factor 2 alpha kinase 1 | Cytoplasm | kinase |
| CDDP 34 | INSRR | insulin receptor related receptor | Plasma Membrane | kinase |
| CDDP 34 | MAP3K15 | mitogen-activated protein kinase kinase kinase 15 | Other | other |
| CDDP 34 | MKNK1 | MAPK interacting serine/threonine kinase 1 | Cytoplasm | kinase |
| CDDP 34 | ROS1 | ROS proto-oncogene 1, receptor tyrosine kinase | Plasma Membrane | kinase |
| CDDP 34 | STK36 | serine/threonine kinase 36 | Cytoplasm | kinase |
| CDDP 34 | MAPK14 | mitogen-activated protein kinase 14 | Cytoplasm | kinase |
| CDDP 35 | CDK15 | cyclin dependent kinase 15 | Plasma Membrane | kinase |
| CDDP 35 | CDKL2 | cyclin dependent kinase like 2 | Nucleus | kinase |
| CDDP 35 | CDKL3 | cyclin dependent kinase like 3 | Cytoplasm | kinase |
| CDDP 35 | CSNK1D | casein kinase 1 delta | Cytoplasm | kinase |
| CDDP 35 | EPHB6 | EPH receptor B6 | Plasma Membrane | kinase |
| CDDP 35 | GAK | cyclin G associated kinase | Nucleus | kinase |
| CDDP 35 | HIPK3 | homeodomain interacting protein kinase 3 | Nucleus | kinase |
| CDDP 35 | EIF2AK1 | eukaryotic translation initiation factor 2 alpha kinase 1 | Cytoplasm | kinase |
| CDDP 35 | MAPKAPK5 | MAPK activated protein kinase 5 | Cytoplasm | kinase |
| CDDP 35 | PRKCA | protein kinase C alpha | Cytoplasm | kinase |
| CDDP 35 | RIPK2 | receptor interacting serine/threonine kinase 2 | Plasma Membrane | kinase |
| CDDP 35 | ROCK2 | Rho associated coiled-coil containing protein kinase 2 | Cytoplasm | kinase |
| CDDP 35 | RPS6KB1 | ribosomal protein S6 kinase B1 | Cytoplasm | kinase |
| CDDP 35 | SIK2 | salt inducible kinase 2 | Cytoplasm | kinase |
| CDDP 35 | SRMS | src-related kinase lacking C-terminal regulatory tyrosine and N-terminal myristylation sites | Cytoplasm | kinase |
| CDDP 36 | ACVR1 | activin A receptor type 1 | Plasma Membrane | kinase |
| CDDP 36 | CDK15 | cyclin dependent kinase 15 | Plasma Membrane | kinase |
| CDDP 36 | AURKA | aurora kinase A | Nucleus | kinase |
| CDDP 36 | AURKB | aurora kinase B | Nucleus | kinase |
| CDDP 36 | AURKC | aurora kinase C | Nucleus | kinase |
| CDDP 36 | AXL | AXL receptor tyrosine kinase | Plasma Membrane | kinase |
| CDDP 36 | BLK | BLK proto-oncogene, Src family tyrosine kinase | Cytoplasm | kinase |
| CDDP 36 | BRSK1 | BR serine/threonine kinase 1 | Cytoplasm | kinase |
| CDDP 36 | BTK | Bruton tyrosine kinase | Cytoplasm | kinase |
| CDDP 36 | CAMK1D | calcium/calmodulin dependent protein kinase ID | Cytoplasm | kinase |
| CDDP 36 | CAMKK1 | calcium/calmodulin dependent protein kinase kinase 1 | Cytoplasm | kinase |
| CDDP 36 | CAMKK2 | calcium/calmodulin dependent protein kinase kinase 2 | Cytoplasm | kinase |
| CDDP 36 | CDC7 | cell division cycle 7 | Nucleus | kinase |
| CDDP 36 | CDK5 | cyclin dependent kinase 5 | Nucleus | kinase |
| CDDP 36 | CDK9 | cyclin dependent kinase 9 | Nucleus | kinase |
| CDDP 36 | CDKL2 | cyclin dependent kinase like 2 | Nucleus | kinase |
| CDDP 36 | CDKL3 | cyclin dependent kinase like 3 | Cytoplasm | kinase |
| CDDP 36 | CSNK2A1 | casein kinase 2 alpha 1 | Nucleus | kinase |
| CDDP 36 | DAPK3 | death associated protein kinase 3 | Cytoplasm | kinase |
| CDDP 36 | DCLK2 | doublecortin like kinase 2 | Cytoplasm | kinase |
| CDDP 36 | DYRK1B | dual specificity tyrosine phosphorylation regulated kinase 1B | Nucleus | kinase |
| CDDP 36 | EEF2K | eukaryotic elongation factor 2 kinase | Cytoplasm | kinase |
| CDDP 36 | EPHA4 | EPH receptor A4 | Plasma Membrane | kinase |
| CDDP 36 | EPHB6 | EPH receptor B6 | Plasma Membrane | kinase |
| CDDP 36 | MAPK12 | mitogen-activated protein kinase 12 | Cytoplasm | kinase |
| CDDP 36 | MAPK7 | mitogen-activated protein kinase 7 | Cytoplasm | kinase |
| CDDP 36 | FER | FER tyrosine kinase | Cytoplasm | kinase |
| CDDP 36 | FES | FES proto-oncogene, tyrosine kinase | Cytoplasm | kinase |
| CDDP 36 | GRK1 | G protein-coupled receptor kinase 1 | Plasma Membrane | kinase |
| CDDP 36 | GRK5 | G protein-coupled receptor kinase 5 | Plasma Membrane | kinase |
| CDDP 36 | HIPK3 | homeodomain interacting protein kinase 3 | Nucleus | kinase |
| CDDP 36 | EIF2AK1 | eukaryotic translation initiation factor 2 alpha kinase 1 | Cytoplasm | kinase |
| CDDP 36 | IGF1R | insulin like growth factor 1 receptor | Plasma Membrane | transmembrane receptor |
| CDDP 36 | IKBKE | inhibitor of nuclear factor kappa B kinase subunit epsilon | Cytoplasm | kinase |
| CDDP 36 | INSR | insulin receptor | Plasma Membrane | kinase |
| CDDP 36 | IRAK1 | interleukin 1 receptor associated kinase 1 | Plasma Membrane | kinase |
| CDDP 36 | JAK2 | Janus kinase 2 | Cytoplasm | kinase |
| CDDP 36 | LIMK1 | LIM domain kinase 1 | Cytoplasm | kinase |
| CDDP 36 | LTK | leukocyte receptor tyrosine kinase | Plasma Membrane | kinase |
| CDDP 36 | MAP3K10 | mitogen-activated protein kinase kinase kinase 10 | Cytoplasm | kinase |
| CDDP 36 | MAP3K15 | mitogen-activated protein kinase kinase kinase 15 | Other | other |
| CDDP 36 | MARK4 | microtubule affinity regulating kinase 4 | Cytoplasm | kinase |
| CDDP 36 | MELK | maternal embryonic leucine zipper kinase | Cytoplasm | kinase |
| CDDP 36 | MET | MET proto-oncogene, receptor tyrosine kinase | Plasma Membrane | kinase |
| CDDP 36 | MKNK1 | MAPK interacting serine/threonine kinase 1 | Cytoplasm | kinase |
| CDDP 36 | MYLK2 | myosin light chain kinase 2 | Cytoplasm | kinase |
| CDDP 36 | NEK6 | NIMA related kinase 6 | Nucleus | kinase |
| CDDP 36 | NLK | nemo like kinase | Nucleus | kinase |
| CDDP 36 | NTRK3 | neurotrophic receptor tyrosine kinase 3 | Plasma Membrane | kinase |
| CDDP 36 | PASK | PAS domain containing serine/threonine kinase | Cytoplasm | kinase |
| CDDP 36 | PIM2 | Pim-2 proto-oncogene, serine/threonine kinase | Nucleus | kinase |
| CDDP 36 | PKD2 | polycystin 2, transient receptor potential cation channel | Plasma Membrane | kinase |
| CDDP 36 | PLK3 | polo like kinase 3 | Nucleus | kinase |
| CDDP 36 | PLK4 | polo like kinase 4 | Cytoplasm | kinase |
| CDDP 36 | RET | ret proto-oncogene | Plasma Membrane | kinase |
| CDDP 36 | RIPK1 | receptor interacting serine/threonine kinase 1 | Plasma Membrane | kinase |
| CDDP 36 | ROS1 | ROS proto-oncogene 1, receptor tyrosine kinase | Plasma Membrane | kinase |
| CDDP 36 | SIK2 | salt inducible kinase 2 | Cytoplasm | kinase |
| CDDP 36 | SRPK1 | SRSF protein kinase 1 | Nucleus | kinase |
| CDDP 36 | STK36 | serine/threonine kinase 36 | Cytoplasm | kinase |
| CDDP 36 | SYK | spleen associated tyrosine kinase | Cytoplasm | kinase |
| CDDP 36 | TBK1 | TANK binding kinase 1 | Cytoplasm | kinase |
| CDDP 36 | TSSK2 | testis specific serine kinase 2 | Cytoplasm | kinase |
| CDDP 36 | TXK | TXK tyrosine kinase | Cytoplasm | kinase |
| CDDP 36 | TYK2 | tyrosine kinase 2 | Plasma Membrane | kinase |
| CDDP 36 | TYRO3 | TYRO3 protein tyrosine kinase | Plasma Membrane | kinase |
| CDDP 37 | CDK15 | cyclin dependent kinase 15 | Plasma Membrane | kinase |
| CDDP 37 | AURKC | aurora kinase C | Nucleus | kinase |
| CDDP 37 | BMPR1A | bone morphogenetic protein receptor type 1A | Plasma Membrane | kinase |
| CDDP 37 | CDC7 | cell division cycle 7 | Nucleus | kinase |
| CDDP 37 | CDK5 | cyclin dependent kinase 5 | Nucleus | kinase |
| CDDP 37 | CDKL2 | cyclin dependent kinase like 2 | Nucleus | kinase |
| CDDP 37 | CDKL3 | cyclin dependent kinase like 3 | Cytoplasm | kinase |
| CDDP 37 | CSNK1D | casein kinase 1 delta | Cytoplasm | kinase |
| CDDP 37 | CSNK2A1 | casein kinase 2 alpha 1 | Nucleus | kinase |
| CDDP 37 | DAPK3 | death associated protein kinase 3 | Cytoplasm | kinase |
| CDDP 37 | EEF2K | eukaryotic elongation factor 2 kinase | Cytoplasm | kinase |
| CDDP 37 | EPHA8 | EPH receptor A8 | Plasma Membrane | kinase |
| CDDP 37 | EPHB6 | EPH receptor B6 | Plasma Membrane | kinase |
| CDDP 37 | FER | FER tyrosine kinase | Cytoplasm | kinase |
| CDDP 37 | GRK1 | G protein-coupled receptor kinase 1 | Plasma Membrane | kinase |
| CDDP 37 | HIPK3 | homeodomain interacting protein kinase 3 | Nucleus | kinase |
| CDDP 37 | EIF2AK1 | eukaryotic translation initiation factor 2 alpha kinase 1 | Cytoplasm | kinase |
| CDDP 37 | MAP3K15 | mitogen-activated protein kinase kinase kinase 15 | Other | other |
| CDDP 37 | MAP2K1 | mitogen-activated protein kinase kinase 1 | Cytoplasm | kinase |
| CDDP 37 | MAP2K7 | mitogen-activated protein kinase kinase 7 | Cytoplasm | kinase |
| CDDP 37 | MKNK1 | MAPK interacting serine/threonine kinase 1 | Cytoplasm | kinase |
| CDDP 37 | MKNK2 | MAPK interacting serine/threonine kinase 2 | Cytoplasm | kinase |
| CDDP 37 | MUSK | muscle associated receptor tyrosine kinase | Plasma Membrane | kinase |
| CDDP 37 | PASK | PAS domain containing serine/threonine kinase | Cytoplasm | kinase |
| CDDP 37 | RIPK1 | receptor interacting serine/threonine kinase 1 | Plasma Membrane | kinase |
| CDDP 37 | ROS1 | ROS proto-oncogene 1, receptor tyrosine kinase | Plasma Membrane | kinase |
| CDDP 37 | SIK2 | salt inducible kinase 2 | Cytoplasm | kinase |
| CDDP 37 | SYK | spleen associated tyrosine kinase | Cytoplasm | kinase |
| CDDP 37 | TGFBR1 | transforming growth factor beta receptor 1 | Plasma Membrane | kinase |
| CDDP 38 | CDK15 | cyclin dependent kinase 15 | Plasma Membrane | kinase |
| CDDP 38 | AURKC | aurora kinase C | Nucleus | kinase |
| CDDP 38 | CAMKK2 | calcium/calmodulin dependent protein kinase kinase 2 | Cytoplasm | kinase |
| CDDP 38 | CDC7 | cell division cycle 7 | Nucleus | kinase |
| CDDP 38 | CDK5 | cyclin dependent kinase 5 | Nucleus | kinase |
| CDDP 38 | CDK9 | cyclin dependent kinase 9 | Nucleus | kinase |
| CDDP 38 | CSNK1E | casein kinase 1 epsilon | Cytoplasm | kinase |
| CDDP 38 | CSNK2A1 | casein kinase 2 alpha 1 | Nucleus | kinase |
| CDDP 38 | CSNK2A2 | casein kinase 2 alpha 2 | Cytoplasm | kinase |
| CDDP 38 | DAPK3 | death associated protein kinase 3 | Cytoplasm | kinase |
| CDDP 38 | DYRK1B | dual specificity tyrosine phosphorylation regulated kinase 1B | Nucleus | kinase |
| CDDP 38 | DYRK3 | dual specificity tyrosine phosphorylation regulated kinase 3 | Nucleus | kinase |
| CDDP 38 | EPHB6 | EPH receptor B6 | Plasma Membrane | kinase |
| CDDP 38 | GRK1 | G protein-coupled receptor kinase 1 | Plasma Membrane | kinase |
| CDDP 38 | HIPK3 | homeodomain interacting protein kinase 3 | Nucleus | kinase |
| CDDP 38 | EIF2AK1 | eukaryotic translation initiation factor 2 alpha kinase 1 | Cytoplasm | kinase |
| CDDP 38 | MAP3K15 | mitogen-activated protein kinase kinase kinase 15 | Other | other |
| CDDP 38 | MAPKAPK5 | MAPK activated protein kinase 5 | Cytoplasm | kinase |
| CDDP 38 | MKNK1 | MAPK interacting serine/threonine kinase 1 | Cytoplasm | kinase |
| CDDP 38 | MKNK2 | MAPK interacting serine/threonine kinase 2 | Cytoplasm | kinase |
| CDDP 38 | PASK | PAS domain containing serine/threonine kinase | Cytoplasm | kinase |
| CDDP 38 | PDGFRA | platelet derived growth factor receptor alpha | Plasma Membrane | kinase |
| CDDP 38 | PIM2 | Pim-2 proto-oncogene, serine/threonine kinase | Nucleus | kinase |
| CDDP 38 | PIM3 | Pim-3 proto-oncogene, serine/threonine kinase | Cytoplasm | kinase |
| CDDP 38 | RIPK1 | receptor interacting serine/threonine kinase 1 | Plasma Membrane | kinase |
| CDDP 38 | ROS1 | ROS proto-oncogene 1, receptor tyrosine kinase | Plasma Membrane | kinase |
| CDDP 38 | SRPK1 | SRSF protein kinase 1 | Nucleus | kinase |
| CDDP 38 | SYK | spleen associated tyrosine kinase | Cytoplasm | kinase |
| CDDP 38 | TYRO3 | TYRO3 protein tyrosine kinase | Plasma Membrane | kinase |
| CDDP 39 | CDK15 | cyclin dependent kinase 15 | Plasma Membrane | kinase |
| CDDP 39 | CAMKK2 | calcium/calmodulin dependent protein kinase kinase 2 | Cytoplasm | kinase |
| CDDP 39 | CDC7 | cell division cycle 7 | Nucleus | kinase |
| CDDP 39 | CDK5 | cyclin dependent kinase 5 | Nucleus | kinase |
| CDDP 39 | CDK9 | cyclin dependent kinase 9 | Nucleus | kinase |
| CDDP 39 | CLK4 | CDC like kinase 4 | Nucleus | kinase |
| CDDP 39 | CSNK1D | casein kinase 1 delta | Cytoplasm | kinase |
| CDDP 39 | CSNK1E | casein kinase 1 epsilon | Cytoplasm | kinase |
| CDDP 39 | CSNK2A1 | casein kinase 2 alpha 1 | Nucleus | kinase |
| CDDP 39 | CSNK2A2 | casein kinase 2 alpha 2 | Cytoplasm | kinase |
| CDDP 39 | DAPK3 | death associated protein kinase 3 | Cytoplasm | kinase |
| CDDP 39 | DYRK1A | dual specificity tyrosine phosphorylation regulated kinase 1A | Nucleus | kinase |
| CDDP 39 | DYRK1B | dual specificity tyrosine phosphorylation regulated kinase 1B | Nucleus | kinase |
| CDDP 39 | DYRK3 | dual specificity tyrosine phosphorylation regulated kinase 3 | Nucleus | kinase |
| CDDP 39 | EPHB6 | EPH receptor B6 | Plasma Membrane | kinase |
| CDDP 39 | GRK1 | G protein-coupled receptor kinase 1 | Plasma Membrane | kinase |
| CDDP 39 | HIPK3 | homeodomain interacting protein kinase 3 | Nucleus | kinase |
| CDDP 39 | EIF2AK1 | eukaryotic translation initiation factor 2 alpha kinase 1 | Cytoplasm | kinase |
| CDDP 39 | MKNK1 | MAPK interacting serine/threonine kinase 1 | Cytoplasm | kinase |
| CDDP 39 | MKNK2 | MAPK interacting serine/threonine kinase 2 | Cytoplasm | kinase |
| CDDP 39 | PIM2 | Pim-2 proto-oncogene, serine/threonine kinase | Nucleus | kinase |
| CDDP 39 | PIM3 | Pim-3 proto-oncogene, serine/threonine kinase | Cytoplasm | kinase |
| CDDP 39 | RIPK1 | receptor interacting serine/threonine kinase 1 | Plasma Membrane | kinase |
| CDDP 39 | ROS1 | ROS proto-oncogene 1, receptor tyrosine kinase | Plasma Membrane | kinase |
| CDDP 39 | SIK2 | salt inducible kinase 2 | Cytoplasm | kinase |
| CDDP 39 | TGFBR1 | transforming growth factor beta receptor 1 | Plasma Membrane | kinase |
| CDDP 40 | BMPR1B | bone morphogenetic protein receptor type 1B | Plasma Membrane | kinase |
| CDDP 40 | CDK8 | cyclin dependent kinase 8 | Nucleus | kinase |
| CDDP 40 | CDKL2 | cyclin dependent kinase like 2 | Nucleus | kinase |
| CDDP 40 | CDKL3 | cyclin dependent kinase like 3 | Cytoplasm | kinase |
| CDDP 40 | CLK4 | CDC like kinase 4 | Nucleus | kinase |
| CDDP 40 | DYRK1A | dual specificity tyrosine phosphorylation regulated kinase 1A | Nucleus | kinase |
| CDDP 40 | EGFR | epidermal growth factor receptor | Plasma Membrane | kinase |
| CDDP 40 | EPHB6 | EPH receptor B6 | Plasma Membrane | kinase |
| CDDP 40 | GRK1 | G protein-coupled receptor kinase 1 | Plasma Membrane | kinase |
| CDDP 40 | GSK3A | glycogen synthase kinase 3 alpha | Nucleus | kinase |
| CDDP 40 | GSK3B | glycogen synthase kinase 3 beta | Nucleus | kinase |
| CDDP 40 | HIPK3 | homeodomain interacting protein kinase 3 | Nucleus | kinase |
| CDDP 40 | EIF2AK1 | eukaryotic translation initiation factor 2 alpha kinase 1 | Cytoplasm | kinase |
| CDDP 40 | MAST1 | microtubule associated serine/threonine kinase 1 | Cytoplasm | kinase |
| CDDP 40 | PRKCA | protein kinase C alpha | Cytoplasm | kinase |

**Table S4. Potential kinase targets of CDDP from known targets and Predicted targets**

| **Gene Symbol** | **Entrez Gene Name** | **Location** | **Type(s)** | **Source** |
| --- | --- | --- | --- | --- |
| ALK | ALK receptor tyrosine kinase | Plasma Membrane | kinase | Known targets |
| AURKB | aurora kinase B | Nucleus | kinase | Known targets |
| AXL | AXL receptor tyrosine kinase | Plasma Membrane | kinase | Known targets |
| CAMK2B | calcium/calmodulin dependent protein kinase II beta | Cytoplasm | kinase | Known targets |
| CDK5 | cyclin dependent kinase 5 | Nucleus | kinase | Known targets |
| CDK6 | cyclin dependent kinase 6 | Nucleus | kinase | Known targets |
| CLK1 | CDC like kinase 1 | Nucleus | kinase | Known targets |
| CSNK1G1 | casein kinase 1 gamma 1 | Cytoplasm | kinase | Known targets |
| CSNK1G2 | casein kinase 1 gamma 2 | Cytoplasm | kinase | Known targets |
| CSNK2A1 | casein kinase 2 alpha 1 | Nucleus | kinase | Known targets |
| CSNK2A2 | casein kinase 2 alpha 2 | Cytoplasm | kinase | Known targets |
| DAPK1 | death associated protein kinase 1 | Cytoplasm | kinase | Known targets |
| EGFR | epidermal growth factor receptor | Plasma Membrane | kinase | Known targets |
| FLT3 | fms related tyrosine kinase 3 | Plasma Membrane | kinase | Known targets |
| FYN | FYN proto-oncogene, Src family tyrosine kinase | Plasma Membrane | kinase | Known targets |
| GSK3A | glycogen synthase kinase 3 alpha | Nucleus | kinase | Known targets |
| GSK3B | glycogen synthase kinase 3 beta | Nucleus | kinase | Known targets |
| IGF1R | insulin like growth factor 1 receptor | Plasma Membrane | transmembrane receptor | Known targets |
| KDR | kinase insert domain receptor | Plasma Membrane | kinase | Known targets |
| LCK | LCK proto-oncogene, Src family tyrosine kinase | Cytoplasm | kinase | Known targets |
| MAP3K5 | mitogen-activated protein kinase kinase kinase 5 | Cytoplasm | kinase | Known targets |
| MAP4K2 | mitogen-activated protein kinase kinase kinase kinase 2 | Cytoplasm | kinase | Known targets |
| MAPK1 | mitogen-activated protein kinase 1 | Cytoplasm | kinase | Known targets |
| MET | MET proto-oncogene, receptor tyrosine kinase | Plasma Membrane | kinase | Known targets |
| NEK2 | NIMA related kinase 2 | Cytoplasm | kinase | Known targets |
| NEK6 | NIMA related kinase 6 | Nucleus | kinase | Known targets |
| PIM1 | Pim-1 proto-oncogene, serine/threonine kinase | Cytoplasm | kinase | Known targets |
| PIM2 | Pim-2 proto-oncogene, serine/threonine kinase | Nucleus | kinase | Known targets |
| PKN1 | protein kinase N1 | Cytoplasm | kinase | Known targets |
| PLK1 | polo like kinase 1 | Nucleus | kinase | Known targets |
| PTK2 | protein tyrosine kinase 2 | Cytoplasm | kinase | Known targets |
| SLK | STE20 like kinase | Nucleus | kinase | Known targets |
| SRC | SRC proto-oncogene, non-receptor tyrosine kinase | Cytoplasm | kinase | Known targets |
| STK16 | serine/threonine kinase 16 | Cytoplasm | kinase | Known targets |
| STK33 | serine/threonine kinase 33 | Cytoplasm | kinase | Known targets |
| SYK | spleen associated tyrosine kinase | Cytoplasm | kinase | Known targets |
| YES1 | YES proto-oncogene 1, Src family tyrosine kinase | Cytoplasm | kinase | Known targets |
| ACVR1 | activin A receptor type 1 | Plasma Membrane | kinase | Predicted targets |
| ALK | ALK receptor tyrosine kinase | Plasma Membrane | kinase | Predicted targets |
| AURKA | aurora kinase A | Nucleus | kinase | Predicted targets |
| AXL | AXL receptor tyrosine kinase | Plasma Membrane | kinase | Predicted targets |
| BLK | BLK proto-oncogene, Src family tyrosine kinase | Cytoplasm | kinase | Predicted targets |
| BRSK1 | BR serine/threonine kinase 1 | Cytoplasm | kinase | Predicted targets |
| CAMK2B | calcium/calmodulin dependent protein kinase II beta | Cytoplasm | kinase | Predicted targets |
| CAMK2D | calcium/calmodulin dependent protein kinase II delta | Cytoplasm | kinase | Predicted targets |
| CAMK2G | calcium/calmodulin dependent protein kinase II gamma | Cytoplasm | kinase | Predicted targets |
| CDK5 | cyclin dependent kinase 5 | Nucleus | kinase | Predicted targets |
| CHEK1 | checkpoint kinase 1 | Nucleus | kinase | Predicted targets |
| CLK1 | CDC like kinase 1 | Nucleus | kinase | Predicted targets |
| CSF1R | colony stimulating factor 1 receptor | Plasma Membrane | kinase | Predicted targets |
| CSNK1A1 | casein kinase 1 alpha 1 | Cytoplasm | kinase | Predicted targets |
| CSNK1D | casein kinase 1 delta | Cytoplasm | kinase | Predicted targets |
| CSNK1G1 | casein kinase 1 gamma 1 | Cytoplasm | kinase | Predicted targets |
| CSNK1G2 | casein kinase 1 gamma 2 | Cytoplasm | kinase | Predicted targets |
| CSNK1G3 | casein kinase 1 gamma 3 | Cytoplasm | kinase | Predicted targets |
| CSNK2A1 | casein kinase 2 alpha 1 | Nucleus | kinase | Predicted targets |
| DAPK3 | death associated protein kinase 3 | Cytoplasm | kinase | Predicted targets |
| DYRK1A | dual specificity tyrosine phosphorylation regulated kinase 1A | Nucleus | kinase | Predicted targets |
| DYRK1B | dual specificity tyrosine phosphorylation regulated kinase 1B | Nucleus | kinase | Predicted targets |
| DYRK2 | dual specificity tyrosine phosphorylation regulated kinase 2 | Cytoplasm | kinase | Predicted targets |
| DYRK3 | dual specificity tyrosine phosphorylation regulated kinase 3 | Nucleus | kinase | Predicted targets |
| EGFR | epidermal growth factor receptor | Plasma Membrane | kinase | Predicted targets |
| FER | FER tyrosine kinase | Cytoplasm | kinase | Predicted targets |
| FGFR1 | fibroblast growth factor receptor 1 | Plasma Membrane | kinase | Predicted targets |
| FGFR3 | fibroblast growth factor receptor 3 | Plasma Membrane | kinase | Predicted targets |
| FLT1 | fms related tyrosine kinase 1 | Plasma Membrane | kinase | Predicted targets |
| FLT3 | fms related tyrosine kinase 3 | Plasma Membrane | kinase | Predicted targets |
| FLT4 | fms related tyrosine kinase 4 | Plasma Membrane | transmembrane receptor | Predicted targets |
| FRK | fyn related Src family tyrosine kinase | Nucleus | kinase | Predicted targets |
| FYN | FYN proto-oncogene, Src family tyrosine kinase | Plasma Membrane | kinase | Predicted targets |
| GRK5 | G protein-coupled receptor kinase 5 | Plasma Membrane | kinase | Predicted targets |
| GSK3A | glycogen synthase kinase 3 alpha | Nucleus | kinase | Predicted targets |
| GSK3B | glycogen synthase kinase 3 beta | Nucleus | kinase | Predicted targets |
| HCK | HCK proto-oncogene, Src family tyrosine kinase | Cytoplasm | kinase | Predicted targets |
| HIPK2 | homeodomain interacting protein kinase 2 | Nucleus | kinase | Predicted targets |
| HIPK4 | homeodomain interacting protein kinase 4 | Cytoplasm | kinase | Predicted targets |
| IKBKE | inhibitor of nuclear factor kappa B kinase subunit epsilon | Cytoplasm | kinase | Predicted targets |
| INSR | insulin receptor | Plasma Membrane | kinase | Predicted targets |
| IRAK1 | interleukin 1 receptor associated kinase 1 | Plasma Membrane | kinase | Predicted targets |
| IRAK4 | interleukin 1 receptor associated kinase 4 | Cytoplasm | kinase | Predicted targets |
| KIT | KIT proto-oncogene, receptor tyrosine kinase | Plasma Membrane | transmembrane receptor | Predicted targets |
| LCK | LCK proto-oncogene, Src family tyrosine kinase | Cytoplasm | kinase | Predicted targets |
| LIMK1 | LIM domain kinase 1 | Cytoplasm | kinase | Predicted targets |
| LTK | leukocyte receptor tyrosine kinase | Plasma Membrane | kinase | Predicted targets |
| MAP3K10 | mitogen-activated protein kinase kinase kinase 10 | Cytoplasm | kinase | Predicted targets |
| MAP3K5 | mitogen-activated protein kinase kinase kinase 5 | Cytoplasm | kinase | Predicted targets |
| MAP4K2 | mitogen-activated protein kinase kinase kinase kinase 2 | Cytoplasm | kinase | Predicted targets |
| MAP4K4 | mitogen-activated protein kinase kinase kinase kinase 4 | Cytoplasm | kinase | Predicted targets |
| MAP4K5 | mitogen-activated protein kinase kinase kinase kinase 5 | Cytoplasm | kinase | Predicted targets |
| MAPK1 | mitogen-activated protein kinase 1 | Cytoplasm | kinase | Predicted targets |
| MAPK12 | mitogen-activated protein kinase 12 | Cytoplasm | kinase | Predicted targets |
| MARK2 | microtubule affinity regulating kinase 2 | Cytoplasm | kinase | Predicted targets |
| MARK3 | microtubule affinity regulating kinase 3 | Cytoplasm | kinase | Predicted targets |
| MARK4 | microtubule affinity regulating kinase 4 | Cytoplasm | kinase | Predicted targets |
| MET | MET proto-oncogene, receptor tyrosine kinase | Plasma Membrane | kinase | Predicted targets |
| MINK1 | misshapen like kinase 1 | Cytoplasm | kinase | Predicted targets |
| MYLK | myosin light chain kinase | Cytoplasm | kinase | Predicted targets |
| NEK4 | NIMA related kinase 4 | Nucleus | kinase | Predicted targets |
| NTRK3 | neurotrophic receptor tyrosine kinase 3 | Plasma Membrane | kinase | Predicted targets |
| PDGFRA | platelet derived growth factor receptor alpha | Plasma Membrane | kinase | Predicted targets |
| PDPK1 | 3-phosphoinositide dependent protein kinase 1 | Cytoplasm | kinase | Predicted targets |
| PHKG2 | phosphorylase kinase catalytic subunit gamma 2 | Cytoplasm | kinase | Predicted targets |
| PIM1 | Pim-1 proto-oncogene, serine/threonine kinase | Cytoplasm | kinase | Predicted targets |
| PIM3 | Pim-3 proto-oncogene, serine/threonine kinase | Cytoplasm | kinase | Predicted targets |
| PKN2 | protein kinase N2 | Cytoplasm | kinase | Predicted targets |
| PLK3 | polo like kinase 3 | Nucleus | kinase | Predicted targets |
| PLK4 | polo like kinase 4 | Cytoplasm | kinase | Predicted targets |
| PRKCA | protein kinase C alpha | Cytoplasm | kinase | Predicted targets |
| PRKCB | protein kinase C beta | Cytoplasm | kinase | Predicted targets |
| PRKCD | protein kinase C delta | Cytoplasm | kinase | Predicted targets |
| PRKG1 | protein kinase cGMP-dependent 1 | Cytoplasm | kinase | Predicted targets |
| PRKX | protein kinase X-linked | Cytoplasm | kinase | Predicted targets |
| PTK2B | protein tyrosine kinase 2 beta | Cytoplasm | kinase | Predicted targets |
| RET | ret proto-oncogene | Plasma Membrane | kinase | Predicted targets |
| ROCK1 | Rho associated coiled-coil containing protein kinase 1 | Cytoplasm | kinase | Predicted targets |
| ROS1 | ROS proto-oncogene 1, receptor tyrosine kinase | Plasma Membrane | kinase | Predicted targets |
| RPS6KA3 | ribosomal protein S6 kinase A3 | Cytoplasm | kinase | Predicted targets |
| SIK2 | salt inducible kinase 2 | Cytoplasm | kinase | Predicted targets |
| SLK | STE20 like kinase | Nucleus | kinase | Predicted targets |
| SRPK1 | SRSF protein kinase 1 | Nucleus | kinase | Predicted targets |
| STK17A | serine/threonine kinase 17a | Nucleus | kinase | Predicted targets |
| STK17B | serine/threonine kinase 17b | Nucleus | kinase | Predicted targets |
| STK3 | serine/threonine kinase 3 | Cytoplasm | kinase | Predicted targets |
| TAOK1 | TAO kinase 1 | Cytoplasm | kinase | Predicted targets |
| TBK1 | TANK binding kinase 1 | Cytoplasm | kinase | Predicted targets |
| TYRO3 | TYRO3 protein tyrosine kinase | Plasma Membrane | kinase | Predicted targets |
| WEE1 | WEE1 G2 checkpoint kinase | Nucleus | kinase | Predicted targets |
| CDK8 | cyclin dependent kinase 8 | Nucleus | kinase | Predicted targets |
| DYRK4 | dual specificity tyrosine phosphorylation regulated kinase 4 | Nucleus | kinase | Predicted targets |

**Table S5. Expression of pAURKB, pMET, pPIM1, pSYK, AURKB, MET, PIM1, and SYK protein levels in four cell lines**

| **Cell lines** | **Group** | **N** | **AURKB** | **pAURKB** | **MET** | **pMET** | **PIM1** | **pPIM1** | **SYK** | **pSYK** |
| --- | --- | --- | --- | --- | --- | --- | --- | --- | --- | --- |
| MCF7 | Control | 3 | 0.930±0.011 | 0.907±0.011 | 0.952±0.011 | 0.930±0.011 | 0.833±0.011 | 0.877±0.011 | 0.793±0.011 | 0.296±0.011 |
| MCF7 | CDDP (0.25 mg/mL) | 3 | 0.888±0.011** | 0.439±0.011**** | 0.937±0.011 | 0.511±0.011**** | 0.766±0.011** | 0.772±0.011*** | 0.770±0.011 | 0.657±0.011**** |
| MCF7 | CDDP (0.5 mg/mL) | 3 | 0.866±0.011** | 0.611±0.011**** | 0.898±0.011** | 0.745±0.011**** | 0.779±0.011** | 0.633±0.011**** | 0.758±0.011* | 0.796±0.011**** |
| MCF7 | CDDP (1.0 mg/mL) | 3 | 0.951±0.011 | 0.545±0.011**** | 0.837±0.011*** | 0.581±0.011**** | 0.762±0.011** | 0.673±0.011**** | 0.822±0.011* | 3.506±0.011**** |
| T47D | Control | 3 | 0.606±0.010 | 1.077±0.020 | 0.739±0.035 | 0.850±0.020 | 0.856±0.025 | 0.866±0.030 | 1.016±0.030 | 0.639±0.026 |
| T47D | CDDP (0.25 mg/mL) | 3 | 0.518±0.016** | 0.863±0.026*** | 0.641±0.020* | 0.617±0.020*** | 0.700±0.020** | 0.363±0.015**** | 0.856±0.020** | 0.692±0.025**** |
| T47D | CDDP (0.5 mg/mL) | 3 | 0.520±0.020** | 0.237±0.020**** | 0.716±0.010 | 0.291±0.020**** | 0.750±0.020** | 0.396±0.020**** | 0.690±0.020**** | 0.299±0.020**** |
| T47D | CDDP (1.0 mg/mL) | 3 | 0.601±0.041 | 0.266±0.020**** | 0.653±0.020* | 0.561±0.020**** | 0.826±0.020 | 0.426±0.020**** | 0.525±0.020**** | 0.269±0.020*** |
| BCPAP | Control | 3 | 0.666±0.014 | 0.908±0.018 | 0.876±0.018 | 0.797±0.018 | 0.823±0.018 | 0.695±0.018 | 0.982±0.014 | 0.562±0.008 |
| BCPAP | CDDP (0.25 mg/mL) | 3 | 0.723±0.018* | 0.821±0.018** | 0.792±0.016** | 0.738±0.018* | 0.746±0.018** | 0.476±0.018*** | 0.721±0.018**** | 0.616±0.018** |
| BCPAP | CDDP (0.5 mg/mL) | 3 | 0.877±0.018**** | 0.898±0.016 | 0.861±0.018 | 0.456±0.018**** | 0.914±0.018** | 0.370±0.018**** | 0.605±0.018**** | 0.821±0.018**** |
| BCPAP | CDDP (1.0 mg/mL) | 3 | 0.907±0.028*** | 0.621±0.008**** | 0.880±0.018 | 0.483±0.018**** | 0.997±0.018*** | 0.318±0.018**** | 0.494±0.018**** | 0.874±0.018**** |
| TPC1 | Control | 3 | 0.637±0.018 | 0.852±0.018 | 0.776±0.018 | 0.616±0.018 | 0.790±0.018 | 0.748±0.018 | 0.935±0.018 | 0.184±0.018 |
| TPC1 | CDDP (0.25 mg/mL) | 3 | 0.601±0.024 | 0.817±0.018 | 0.795±0.018 | 0.651±0.018 | 0.763±0.018 | 0.722±0.041 | 0.782±0.018*** | 0.341±0.018*** |
| TPC1 | CDDP (0.5 mg/mL) | 3 | 0.725±0.069 | 0.622±0.098* | 0.848±0.041* | 0.523±0.052* | 0.798±0.018 | 0.499±0.058** | 0.620±0.018**** | 0.467±0.018**** |
| TPC1 | CDDP (1.0 mg/mL) | 3 | 0.719±0.075 | 0.316±0.029**** | 0.762±0.024 | 0.370±0.041*** | 0.814±0.013 | 0.621±0.035** | 0.477±0.018**** | 0.760±0.018**** |
| Notes: α-tubulin was used as a loading control. Numerical data represent the mean ± standard deviation of 3 independent experiments. Statistical significance was determined by a two-tailed, unpaired Student t-test (Compared with control group, *P<0.05, **P<0.01, ***P<0.001, ****P<0.0001). | | | | | | | | | | |
